# Supplementary material for: White Blood Cells and Severe COVID-19: A Mendelian Randomization Study
Source: J Pers Med. 2021 Mar 12;11(3):195. doi: 10.3390/jpm11030195 (PMC8002054; doi:10.3390/jpm11030195)
Supplement: Supplementary file 1 [file jpm-11-00195-s001.zip › jpm-1137550-SI/Supplementary Information. Tables S2-21.pdf]

Title: White blood cells and severe COVID-19: a Mendelian randomization study

Yitang Sun [1], Jingqi Zhou [1,2] and Kaixiong Ye [1,3,*]

[1] Department of Genetics, Franklin College of Arts and Sciences, University of Georgia, Athens, GA, USA; Yitang.Sun@uga.edu (Y.S.); Jingqi.Zhou@uga.edu (J.Z.)
[2] School of Public Health, Shanghai Jiao Tong University School of Medicine, Shanghai, P.R. China;
[3] Institute of Bioinformatics, University of Georgia, Athens, GA, USA
* Correspondence: Kaixiong.Ye@uga.edu; Tel.: 706-542-5898, Fax: 706-542-3910

* **Address for Correspondence:**

Dr. Kaixiong Ye
Department of Genetics
University of Georgia
C220 Davison Life Sciences
120 East Green Street, Athens, GA 30602
Office: 706-542-5898
Fax: 706-542-3910
Email: Kaixiong.Ye@uga.edu

Table S2. 401 SNPs significantly associated with white blood cell count used IVs in forward MR analyses derived from Vuckovic D et al. Chr: Chromosome; EA: Effect allele; NEA: Non-effect allele; EAF: Effect allele frequency.

SNP	Chr	Pos	EA	NEA	EAF	Beta	SE	P-value
rs4908508	1	8800780	C	T	0.206	0.028	0.003	5.3e-27
rs111877086	1	9702591	C	T	0.192	-0.019	0.003	2.5e-12
rs284317	1	10731625	G	A	0.495	0.019	0.002	2e-18
rs10917107	1	22329414	A	G	0.690	-0.015	0.002	1.7e-10
rs60918921	1	23850590	A	C	0.618	-0.020	0.002	5.6e-20
rs71638504	1	27327822	A	G	0.056	-0.030	0.005	3.4e-10
rs3762297	1	31231680	T	C	0.183	0.026	0.003	6e-22
rs3917932	1	36943916	G	C	0.577	-0.044	0.002	1.6e-95
rs11800162	1	36970512	T	C	0.020	0.053	0.008	4.6e-12
rs3754224	1	43423622	C	T	0.269	-0.016	0.002	4.2e-11
rs9429088	1	46497500	A	T	0.435	-0.022	0.002	3.4e-25
rs12121236	1	56617911	A	C	0.352	0.015	0.002	7.6e-12
rs11208712	1	66148652	C	T	0.356	-0.044	0.002	4.6e-89
rs61780038	1	67487376	A	C	0.559	-0.018	0.002	1.2e-14
rs41313381	1	79411968	A	C	0.031	0.050	0.006	6.8e-17
rs150649461	1	92925654	C	G	0.015	0.065	0.009	4.9e-13
rs56674564	1	101097718	C	T	0.354	-0.016	0.002	1.8e-12
rs6696259	1	101221482	C	G	0.410	0.020	0.002	7.7e-21
rs9429767	1	110496087	A	G	0.198	0.018	0.003	6.5e-11
rs2476601	1	114377568	G	A	0.899	0.052	0.004	8.1e-50
rs4970996	1	150506589	C	G	0.751	0.015	0.002	2.7e-09
rs568035	1	156110167	T	C	0.064	-0.032	0.004	1.8e-13
rs34599082	1	159175494	T	C	0.014	-0.165	0.009	2.6e-73
rs11587213	1	161184875	G	A	0.183	-0.019	0.003	1.5e-12
rs192123799	1	161608301	C	T	0.125	-0.034	0.003	1.4e-24
rs4657690	1	167601061	A	G	0.576	0.013	0.002	5e-10
rs3795503	1	180905694	T	C	0.315	-0.016	0.002	2.1e-12
rs1779807	1	182164250	T	C	0.333	0.014	0.002	1.9e-10
rs41272536	1	183440531	G	A	0.046	-0.033	0.005	1.2e-10
rs10494783	1	198663661	A	G	0.051	-0.047	0.005	3.6e-22
rs1060286	1	207250300	A	G	0.611	0.013	0.002	6.9e-10
rs4844390	1	207934849	G	A	0.222	-0.025	0.003	1.2e-23
rs10916617	1	224637070	T	C	0.212	0.026	0.003	3.5e-23
rs2615061	1	225895806	A	G	0.116	-0.029	0.003	5.8e-18
rs7552783	1	227174145	C	T	0.512	-0.017	0.002	9.2e-14
rs533483	1	234765256	A	G	0.246	-0.017	0.002	3.3e-11
rs9970896	1	236104981	T	A	0.888	-0.060	0.003	8.2e-71
rs34298354	1	247588053	T	C	0.122	-0.025	0.003	6.8e-15
rs56188865	1	247606276	C	T	0.372	-0.026	0.002	1.1e-32
rs1469217	2	12950124	C	T	0.493	0.019	0.002	3.1e-19
rs4632345	2	16702654	A	G	0.678	-0.015	0.002	1.7e-10
rs377110161	2	23963462	G	A	0.128	0.036	0.003	2.9e-29
rs1260326	2	27730940	C	T	0.606	-0.033	0.002	3.1e-53
rs1509396	2	28612213	C	G	0.507	0.013	0.002	7.8e-10
rs935655	2	46067445	G	T	0.762	0.016	0.002	1.7e-10
rs75475627	2	54787592	G	C	0.076	0.035	0.004	6.5e-18
rs2421200	2	61711815	T	G	0.487	-0.022	0.002	3e-26
rs7569257	2	65656976	A	G	0.409	0.026	0.002	3e-33
rs4599108	2	85543222	T	C	0.486	-0.018	0.002	1.3e-16
rs7575217	2	101776932	G	A	0.673	0.023	0.002	9.2e-24
rs7370196	2	102297387	C	T	0.685	0.016	0.002	4.9e-12

rs13011687	2	102535941	C	A	0.257	0.014	0.002	2.3e-09
rs35898589	2	112915809	G	A	0.048	0.031	0.005	4.1e-10
rs55709272	2	113867288	C	T	0.437	0.035	0.002	6.7e-61
rs77524493	2	128618594	T	A	0.029	0.041	0.007	1.7e-10
rs79716587	2	143886819	A	G	0.126	-0.036	0.003	1.7e-29
rs3856364	2	145477217	G	C	0.681	0.015	0.002	1.3e-10
rs62173240	2	160352482	A	G	0.023	-0.054	0.007	7.4e-14
rs2729707	2	160687231	G	A	0.832	-0.032	0.003	1.5e-29
rs2068330	2	163237390	G	C	0.358	0.019	0.002	3.3e-17
rs2632372	2	169717541	C	T	0.504	-0.021	0.002	8e-24
rs17270868	2	182214669	C	T	0.241	0.033	0.002	1.7e-40
rs7573465	2	182315885	T	G	0.557	0.036	0.002	2.4e-62
rs12987470	2	188323311	T	A	0.295	-0.017	0.002	1.5e-13
rs13392977	2	192514856	A	G	0.054	0.033	0.005	1.3e-12
rs6760993	2	202110917	A	G	0.530	0.016	0.002	1.1e-14
rs1047891	2	211540507	A	C	0.316	-0.020	0.002	1.6e-18
rs114050631	2	219020958	T	C	0.011	-0.139	0.011	6.4e-38
rs72951772	2	219990997	A	C	0.127	-0.020	0.003	7.4e-10
rs6755895	2	232579795	C	T	0.227	-0.024	0.003	3.9e-21
rs10164769	2	237779229	T	C	0.740	0.027	0.002	7.2e-28
rs1822534	3	12266804	G	A	0.392	-0.029	0.002	1.7e-39
rs479404	3	27363064	C	T	0.269	-0.015	0.002	5e-10
rs2371108	3	27757018	T	G	0.387	0.021	0.002	3e-22
rs3732378	3	39307162	A	G	0.173	0.023	0.003	7.3e-16
rs9872570	3	42896515	A	T	0.385	-0.026	0.002	1.6e-32
rs4682844	3	46982737	C	T	0.419	-0.027	0.002	4.1e-33
rs6779340	3	58033701	G	C	0.336	-0.020	0.002	7.6e-19
rs832187	3	63833050	T	C	0.634	-0.014	0.002	6.1e-11
rs6445424	3	64717575	A	C	0.238	-0.016	0.002	2.5e-10
rs17737336	3	70764744	C	T	0.237	-0.017	0.003	3.2e-11
rs7639292	3	107295665	T	C	0.170	-0.026	0.003	7.1e-20
rs34198449	3	128381270	A	G	0.270	-0.029	0.002	2.9e-33
rs10935013	3	132227935	A	G	0.113	0.022	0.003	6.5e-11
rs11712552	3	140957762	G	A	0.127	0.030	0.003	1.3e-20
rs9819371	3	141206800	T	C	0.065	-0.048	0.004	6.4e-29
rs6440050	3	141622749	C	G	0.501	-0.013	0.002	2.4e-10
rs6440732	3	150990510	A	C	0.825	0.023	0.003	1.8e-16
rs55730982	3	156797208	G	T	0.401	0.014	0.002	7.6e-11
rs12494724	3	169320998	T	C	0.634	-0.014	0.002	5.2e-11
rs4074672	3	183730295	T	C	0.369	0.019	0.002	1.6e-18
rs9867398	3	185912816	T	C	0.094	0.022	0.004	2.3e-09
rs4490348	3	186204424	A	G	0.410	0.013	0.002	1.9e-09
rs2089979	3	196501413	G	A	0.416	-0.035	0.002	2.5e-59
rs56011263	4	702972	C	T	0.388	0.022	0.002	3.1e-24
rs73191188	4	3105200	A	G	0.353	-0.016	0.002	1e-12
rs6831368	4	6969919	G	A	0.362	-0.020	0.002	4.1e-20
rs28530750	4	36312542	A	G	0.043	0.058	0.005	6.8e-28
rs987121	4	38329783	T	A	0.603	0.015	0.002	1.4e-11
rs13132853	4	38680015	G	A	0.361	0.024	0.002	3.5e-27
rs218264	4	55408875	T	A	0.249	0.029	0.002	8.8e-32
rs723585	4	55503194	G	A	0.486	-0.019	0.002	8.5e-20
rs2412771	4	57761417	C	T	0.417	-0.020	0.002	9.2e-20
rs11723621	4	72615362	G	A	0.292	-0.031	0.002	3.8e-39
rs16850073	4	74703999	T	C	0.375	0.043	0.002	2.6e-83
rs11725704	4	74959996	G	A	0.376	0.065	0.002	7.5e-194
rs116746683	4	83471084	T	C	0.062	-0.032	0.004	4.1e-13

rs28378092	4	90228807	T	C	0.458	0.013	0.002	3.4e-09
rs113473633	4	103449131	G	A	0.026	-0.050	0.007	1e-12
rs144317085	4	105806108	T	A	0.034	0.049	0.006	2.6e-16
rs74693727	4	120330155	T	C	0.328	-0.017	0.002	1.5e-14
rs11735662	4	145026126	T	C	0.034	0.053	0.006	5.7e-20
rs2290846	4	151199080	A	G	0.287	0.018	0.002	7.6e-14
rs6817881	4	152223904	T	C	0.500	0.014	0.002	1.3e-10
rs4535497	5	1107428	A	C	0.570	-0.013	0.002	4.3e-10
rs7705526	5	1285974	A	C	0.326	0.030	0.002	1.2e-38
rs464609	5	34654477	A	G	0.544	0.014	0.002	1.7e-10
rs3822733	5	35875134	C	T	0.259	0.022	0.002	3e-20
rs13155659	5	54987585	C	T	0.699	-0.014	0.002	7.8e-10
rs11744663	5	57315635	A	G	0.192	-0.020	0.003	8.2e-14
rs2964199	5	57532775	C	T	0.682	-0.016	0.002	7.8e-13
rs11741775	5	68590395	T	C	0.443	-0.022	0.002	1.8e-24
rs4703890	5	71748624	A	G	0.882	-0.039	0.003	7.2e-32
rs6876611	5	96243598	T	A	0.486	-0.013	0.002	2.8e-09
rs141543178	5	108133865	T	A	0.026	0.044	0.007	6.7e-11
rs257063	5	114806819	T	C	0.743	-0.015	0.002	7e-10
rs79272926	5	118726637	A	C	0.220	-0.025	0.003	4.4e-22
rs4836224	5	122092875	C	A	0.426	0.014	0.002	1.9e-11
rs2631367	5	131705458	G	C	0.522	0.034	0.002	3.4e-58
rs79237520	5	134712566	T	C	0.023	0.043	0.007	1.5e-09
rs6580229	5	141510754	G	A	0.617	0.025	0.002	8.6e-31
rs2082382	5	148200553	A	G	0.548	-0.021	0.002	1.9e-22
rs4704826	5	156392082	A	C	0.635	-0.016	0.002	4e-13
rs10053217	5	173102585	G	A	0.355	0.014	0.002	8.1e-10
rs2594836	5	173205318	A	G	0.722	-0.036	0.002	1.4e-50
rs13180726	5	179126457	A	G	0.827	-0.022	0.003	1.5e-14
rs12203592	6	396321	T	C	0.220	0.024	0.003	4.2e-20
rs1761678	6	7090640	T	G	0.873	-0.021	0.003	9.3e-11
rs1285886	6	7140831	A	G	0.197	0.029	0.003	1.2e-27
rs1334576	6	7211818	A	G	0.425	-0.013	0.002	1.3e-09
rs1087924	6	10523826	C	G	0.529	-0.013	0.002	4.3e-10
rs1144700	6	16744687	T	C	0.178	-0.031	0.003	3.3e-29
rs9350354	6	21393645	G	A	0.362	0.016	0.002	1.5e-13
rs62392759	6	25804685	A	C	0.125	0.021	0.003	4.9e-11
rs73740434	6	27144980	A	C	0.082	-0.066	0.005	2.8e-37
rs6902400	6	30425276	T	C	0.179	0.034	0.003	4.5e-34
rs2905757	6	31022828	T	C	0.324	0.069	0.002	1e-200
rs9265546	6	31298202	A	G	0.223	-0.067	0.003	2.3e-112
rs200801362	6	31555480	C	T	0.161	-0.090	0.004	1e-116
rs74973088	6	32457874	C	T	0.465	0.077	0.002	1e-200
rs9274305	6	32632000	T	C	0.205	-0.052	0.003	2.7e-75
rs149066177	6	34219560	A	T	0.112	-0.021	0.003	3.4e-10
rs6902672	6	41165840	C	T	0.207	-0.019	0.003	4.4e-13
rs11965885	6	43693094	T	G	0.582	-0.016	0.002	5.5e-14
rs6937438	6	43815364	A	G	0.707	0.016	0.002	7.8e-12
rs900403	6	44591850	A	G	0.425	-0.013	0.002	3.6e-10
rs2012011	6	46893355	A	G	0.135	-0.027	0.003	2.5e-18
rs71548708	6	87837599	T	C	0.497	-0.018	0.002	3.7e-17
rs4707609	6	90946479	C	T	0.362	-0.022	0.002	4.1e-24
rs364663	6	105443189	A	T	0.557	-0.014	0.002	1.3e-10
rs6927569	6	109621494	C	T	0.523	0.023	0.002	2.3e-28
rs1741820	6	122721510	G	A	0.545	0.021	0.002	4.6e-22
rs35786788	6	135419042	A	G	0.253	-0.050	0.002	2.4e-90

rs9402702	6	135634006	C	T	0.492	0.020	0.002	4.9e-21
rs6924387	6	137082948	G	A	0.410	0.017	0.002	1.2e-14
rs72978754	6	138053203	C	T	0.066	-0.029	0.004	1.2e-11
rs9390461	6	147701217	G	A	0.540	0.016	0.002	8.5e-14
rs1738074	6	159465977	C	T	0.565	-0.020	0.002	3.7e-21
rs71536939	6	170486950	G	A	0.785	0.017	0.003	1.2e-09
rs798540	7	2764130	A	G	0.297	0.019	0.002	8.3e-16
rs2462661	7	6702311	G	T	0.576	0.017	0.002	1.1e-15
rs56195338	7	8107922	A	G	0.058	-0.032	0.005	1.1e-12
rs7776857	7	22754768	T	G	0.655	-0.019	0.002	1.4e-17
rs2158799	7	28277107	G	C	0.610	0.043	0.002	5e-88
rs56388170	7	28724374	T	G	0.292	0.060	0.002	7.6e-144
rs2710804	7	36084529	C	T	0.377	0.023	0.002	7.1e-26
rs3735485	7	45009341	G	A	0.845	0.040	0.003	1.2e-42
rs865031	7	47448640	C	A	0.431	-0.013	0.002	3.9e-10
rs6583435	7	50293405	A	C	0.676	0.019	0.002	1.5e-16
rs4689	7	56067016	T	G	0.760	-0.018	0.002	1.5e-12
rs4276561	7	66120952	C	T	0.046	-0.037	0.005	2e-13
rs33951980	7	73029437	T	C	0.130	-0.025	0.003	7.2e-16
rs79864016	7	75655838	G	A	0.166	-0.018	0.003	7.6e-10
rs8179	7	92236164	C	T	0.791	-0.038	0.003	4.4e-48
rs445	7	92408370	T	C	0.096	-0.100	0.004	8.6e-169
rs2107717	7	98005398	C	T	0.679	-0.014	0.002	1.3e-09
rs342293	7	106372219	G	C	0.459	-0.017	0.002	2.4e-16
rs11768743	7	129632081	T	A	0.214	0.016	0.003	3.9e-09
rs9656395	7	130575884	G	A	0.095	-0.030	0.004	1.5e-16
rs7803075	7	130742066	G	A	0.739	-0.026	0.002	5e-27
rs13231262	7	148886058	T	C	0.656	-0.016	0.002	5.9e-13
rs7464263	8	11434176	T	A	0.529	0.025	0.002	9.5e-31
rs12550612	8	22966769	A	G	0.824	-0.034	0.003	9.2e-34
rs2322586	8	27323182	G	C	0.857	-0.019	0.003	3.2e-10
rs2979489	8	30280833	A	G	0.742	0.018	0.002	2.5e-14
rs28571765	8	55449281	C	T	0.209	-0.022	0.003	3.9e-17
rs12676105	8	56795439	T	C	0.505	-0.017	0.002	1.9e-16
rs7822169	8	61417284	G	A	0.371	0.021	0.002	5.4e-22
rs7846314	8	61650831	T	A	0.187	0.049	0.003	4.4e-71
rs4623401	8	68797804	G	A	0.467	-0.019	0.002	1.8e-19
rs1947897	8	78946874	C	G	0.332	-0.016	0.002	3.4e-13
rs62510269	8	79012343	G	A	0.147	-0.037	0.003	1.4e-34
rs10808538	8	87169063	A	G	0.297	0.014	0.002	9.1e-10
rs10104995	8	103901234	T	C	0.231	-0.022	0.003	2e-18
rs2957421	8	106275376	A	G	0.243	0.016	0.002	1.8e-10
rs4734879	8	106583124	G	A	0.275	-0.022	0.002	5.3e-20
rs71510325	8	126298640	T	C	0.598	0.014	0.002	7.1e-11
rs4733823	8	129014697	T	C	0.456	0.021	0.002	4.9e-23
rs59697075	8	130618150	T	C	0.588	-0.038	0.002	3.2e-69
rs145209947	8	130694185	A	C	0.015	-0.075	0.010	1.4e-14
rs7005996	8	142241681	T	C	0.906	0.026	0.004	5.1e-13
rs34634548	8	142329460	A	C	0.358	-0.028	0.002	3.9e-37
rs2992836	9	276053	T	G	0.188	0.024	0.003	1e-18
rs2219143	9	2622278	A	G	0.393	0.020	0.002	4.8e-19
rs385893	9	4763176	C	T	0.524	0.020	0.002	7.1e-22
rs10758669	9	4981602	A	C	0.659	-0.014	0.002	2.8e-10
rs7036656	9	21990457	T	C	0.722	0.029	0.002	4.2e-35
rs10757287	9	22143570	T	A	0.162	-0.035	0.003	1.1e-33
rs3793537	9	35687556	C	G	0.293	0.021	0.002	1.2e-18

rs12343705	9	38197187	T	A	0.490	-0.014	0.002	2e-10
rs611461	9	79325615	G	A	0.759	-0.018	0.002	1.5e-13
rs696825	9	86583076	T	C	0.252	-0.025	0.002	4.1e-25
rs1288649	9	91426717	G	T	0.613	-0.021	0.002	3.7e-21
rs10992433	9	95483503	G	A	0.231	0.017	0.003	4.6e-11
rs1930249	9	112735377	G	A	0.172	0.022	0.003	3e-15
rs1411424	9	113892963	A	G	0.523	-0.028	0.002	1.7e-38
rs7851073	9	114654460	T	C	0.309	0.017	0.002	4.1e-13
rs113702392	9	116039908	T	C	0.226	-0.017	0.003	7.5e-11
rs10760127	9	123692940	C	G	0.569	0.014	0.002	1.2e-10
rs10986338	9	127191232	A	G	0.651	-0.018	0.002	2.7e-16
rs2519093	9	136141870	T	C	0.184	-0.044	0.003	6.7e-57
rs2905064	9	136949558	T	A	0.484	0.015	0.002	1.2e-12
rs12555241	9	139387676	A	G	0.278	0.032	0.002	1.8e-41
rs9329341	10	13532447	A	G	0.580	0.017	0.002	1.3e-15
rs10828725	10	25218243	T	G	0.369	-0.050	0.002	1.8e-113
rs61838426	10	26735804	T	G	0.323	-0.016	0.002	5.2e-13
rs2807742	10	28781367	A	G	0.770	0.034	0.003	1.2e-41
rs2091084	10	30492267	C	T	0.653	0.017	0.002	1.3e-13
rs72790861	10	44880207	G	C	0.308	-0.026	0.002	1.1e-28
rs61850684	10	50252936	A	G	0.235	-0.024	0.003	2.3e-21
rs4746522	10	64437684	A	G	0.277	0.015	0.002	1.2e-10
rs7082470	10	65277026	A	G	0.471	-0.029	0.002	3.4e-41
rs10997821	10	69570691	G	A	0.104	-0.023	0.003	2.3e-11
rs3747869	10	73520632	C	A	0.901	0.038	0.004	2e-27
rs7098414	10	82214586	C	A	0.732	0.015	0.002	5.8e-10
rs1412445	10	91002804	T	C	0.338	0.019	0.002	9.1e-17
rs11187838	10	96038686	A	G	0.434	-0.019	0.002	9.8e-20
rs61863767	10	99084426	T	C	0.597	0.046	0.002	4e-100
rs11593526	10	104228017	A	C	0.539	0.016	0.002	2.3e-14
rs2803606	10	113902827	T	C	0.246	-0.015	0.002	5e-10
rs180943	10	115719472	G	C	0.587	-0.019	0.002	4.3e-18
rs12784071	10	115854445	T	C	0.230	0.017	0.003	4.6e-12
rs3781454	10	126348565	A	G	0.681	0.025	0.002	2.3e-28
rs14408	11	308314	C	T	0.368	0.044	0.002	9.7e-89
rs1468102	11	3004526	G	C	0.306	0.017	0.002	1.2e-13
rs7949917	11	10475370	A	G	0.596	0.014	0.002	1.6e-10
rs11022177	11	12133478	G	C	0.310	-0.017	0.002	1.5e-13
rs2049045	11	27694241	C	G	0.186	0.017	0.003	5.9e-10
rs1227996	11	47965189	G	C	0.661	0.027	0.002	1.9e-32
rs174549	11	61571382	A	G	0.309	-0.027	0.002	1.7e-31
rs10896064	11	65641033	C	G	0.532	0.019	0.002	2.3e-18
rs4980661	11	69306579	A	G	0.527	0.017	0.002	3.5e-15
rs12363256	11	100520680	A	T	0.327	0.018	0.002	5.6e-15
rs228606	11	108087847	T	G	0.411	0.018	0.002	2.3e-16
rs238914	11	113984109	A	C	0.396	0.029	0.002	5.3e-41
rs148713124	11	118225200	T	G	0.009	0.107	0.011	9.9e-21
rs71468750	11	120205560	A	G	0.413	0.014	0.002	2e-10
rs8705	11	128328913	A	G	0.318	-0.028	0.002	7.3e-35
rs632887	12	3392351	G	A	0.410	0.014	0.002	3.9e-11
rs7955734	12	4333159	G	C	0.210	-0.028	0.003	2.1e-27
rs2286599	12	6499533	A	G	0.142	0.037	0.003	3e-33
rs2024385	12	12888438	A	T	0.428	0.017	0.002	1.1e-14
rs7134738	12	26358170	C	T	0.351	-0.014	0.002	1e-10
rs739842	12	48202046	C	T	0.635	-0.016	0.002	3.3e-12
rs7978263	12	49118291	T	C	0.314	-0.016	0.002	9.3e-12

rs11169302	12	50578705	G	T	0.394	0.021	0.002	1.2e-22
rs2452793	12	51539508	A	G	0.880	-0.020	0.003	4.8e-10
rs706809	12	52294257	C	T	0.775	0.022	0.003	4.3e-18
rs1245035	12	64976049	A	C	0.629	0.015	0.002	3.4e-12
rs971546	12	68591919	G	T	0.704	0.016	0.002	2e-11
rs4761234	12	69732105	C	T	0.485	0.015	0.002	8.8e-13
rs12424773	12	80033612	T	C	0.691	-0.015	0.002	1.1e-10
rs7487314	12	88836215	T	G	0.701	-0.025	0.002	1.1e-27
rs17041439	12	101873240	C	A	0.056	0.041	0.005	1.2e-18
rs11835066	12	109844678	C	A	0.050	0.029	0.005	2.9e-09
rs10774624	12	111833788	A	G	0.515	-0.067	0.002	1e-200
rs75468472	12	112363239	C	T	0.015	0.071	0.011	4.8e-10
rs11064881	12	120146925	A	G	0.073	-0.034	0.004	1.8e-16
rs34944476	12	124006269	T	C	0.219	0.017	0.003	2.2e-11
rs9863	12	124421453	C	T	0.327	-0.020	0.002	9e-18
rs76428106	13	28604007	C	T	0.013	0.167	0.010	2.2e-65
rs138028125	13	28712689	G	C	0.035	0.056	0.006	5.6e-21
rs9508005	13	28789794	G	T	0.095	-0.023	0.004	1.6e-09
rs7326825	13	50113450	A	G	0.707	0.022	0.002	8.7e-21
rs706606	13	50833707	C	T	0.531	0.017	0.002	4.2e-15
rs3812849	13	74701736	C	A	0.264	0.021	0.002	1.1e-17
rs17785077	13	99635611	A	G	0.103	0.023	0.003	7.5e-11
rs12874404	13	108993494	G	A	0.053	-0.037	0.005	2.8e-15
rs2260302	13	114150850	A	G	0.258	0.023	0.002	1.3e-21
rs9590390	13	114920867	A	G	0.294	0.016	0.002	8.1e-12
rs7159281	14	23756604	G	A	0.669	-0.014	0.002	5.7e-10
rs2038700	14	25461989	C	T	0.394	0.035	0.002	1.7e-56
rs17386684	14	35602087	G	T	0.178	0.024	0.003	2.4e-17
rs696	14	35871093	T	C	0.363	0.017	0.002	4.8e-14
rs10138752	14	69179971	T	C	0.078	-0.040	0.004	2.8e-24
rs175714	14	75981856	C	T	0.577	0.013	0.002	1.1e-09
rs2205190	14	77886723	G	T	0.521	-0.014	0.002	2.6e-11
rs9806027	14	81668707	C	G	0.554	-0.018	0.002	6e-17
rs117068593	14	93118229	T	C	0.189	-0.029	0.003	1.6e-26
rs36084521	14	93516398	G	T	0.111	0.023	0.003	9.2e-12
rs10146962	14	101170540	C	T	0.335	-0.016	0.002	1.1e-12
rs56227024	14	103861706	G	A	0.283	-0.019	0.002	2.3e-16
rs4924450	15	40597229	A	G	0.704	0.016	0.002	1.5e-11
rs72726027	15	42248826	C	T	0.111	-0.045	0.003	4e-41
rs60695341	15	51010271	T	C	0.197	-0.030	0.003	5.6e-29
rs398474	15	62799035	A	G	0.273	0.016	0.002	1.9e-11
rs7180079	15	64629873	G	A	0.879	0.036	0.003	9.2e-28
rs743580	15	74328116	G	A	0.490	0.014	0.002	1.3e-10
rs67538199	15	75361650	C	T	0.161	-0.020	0.003	1.2e-11
rs8024737	15	77249703	C	T	0.542	0.013	0.002	3.9e-09
rs2562751	15	80251294	A	G	0.760	0.015	0.003	1.8e-09
rs7177338	15	91428636	A	G	0.525	-0.017	0.002	4.2e-15
rs28576226	15	101713668	A	G	0.124	0.034	0.003	2.8e-26
rs75893715	16	1360091	T	C	0.271	0.018	0.002	1.2e-13
rs35929659	16	2165630	C	T	0.180	-0.016	0.003	3.7e-09
rs6500550	16	3746241	T	C	0.302	-0.022	0.002	4.1e-22
rs17199009	16	3867081	G	A	0.070	0.027	0.004	9.4e-11
rs61739285	16	27480797	T	C	0.034	-0.035	0.006	1.3e-09
rs11574938	16	30485393	C	G	0.517	0.040	0.002	1.5e-79
rs28853644	16	30801027	T	C	0.270	-0.019	0.002	7.6e-16
rs2080506	16	50164672	T	C	0.156	-0.018	0.003	5.9e-10

rs11644125	16	57058974	T	C	0.600	-0.018	0.002	8.4e-17
rs12918121	16	67187795	T	C	0.018	-0.051	0.008	3.2e-10
rs117784871	16	68138091	T	G	0.057	0.029	0.005	1.7e-09
rs11642655	16	81610480	A	G	0.565	0.015	0.002	8.2e-13
rs247826	16	84582965	T	C	0.222	0.035	0.003	2.9e-41
rs9939427	16	86016091	A	G	0.231	0.022	0.003	7.7e-18
rs59404182	16	88852866	G	A	0.199	-0.020	0.003	8.7e-14
rs2229075	17	1359363	G	T	0.722	0.016	0.002	2e-11
rs7225843	17	2001825	C	T	0.203	-0.033	0.003	7.1e-37
rs62089694	17	2723868	G	C	0.161	0.021	0.003	5.2e-13
rs8071086	17	4612866	G	A	0.294	0.015	0.002	5.9e-11
rs9896414	17	5015895	T	C	0.509	-0.018	0.002	7.1e-17
rs1029809	17	16156235	G	A	0.475	-0.026	0.002	1.6e-33
rs55767800	17	17674967	C	T	0.635	-0.020	0.002	2.2e-19
rs8070966	17	27196748	C	T	0.844	-0.021	0.003	2.3e-13
rs2259855	17	28097860	C	T	0.526	-0.026	0.002	1.4e-33
rs79730542	17	37298789	C	T	0.031	-0.037	0.006	4.4e-09
rs11651596	17	38056116	C	T	0.468	-0.065	0.002	1e-200
rs576566496	17	38140269	A	G	0.027	0.085	0.007	1.1e-34
rs74725931	17	38196327	C	T	0.038	0.058	0.006	3.6e-24
rs563136944	17	38470429	G	T	0.356	-0.023	0.003	1e-19
rs4796657	17	40589978	A	C	0.573	-0.014	0.002	4.3e-11
rs138284624	17	56385573	T	C	0.013	0.110	0.009	3.4e-31
rs8081395	17	57801761	A	G	0.545	0.038	0.002	3e-70
rs2084312	17	72695211	T	C	0.800	0.029	0.003	6.8e-27
rs12939873	17	74460148	C	T	0.657	-0.013	0.002	3.9e-09
rs8079218	17	76247744	G	A	0.498	-0.015	0.002	2.3e-12
rs9747839	17	81084522	G	C	0.478	0.020	0.002	2.8e-17
rs4468717	18	3457606	T	C	0.077	-0.024	0.004	1.7e-09
rs303753	18	21074922	A	G	0.346	-0.021	0.002	4.7e-20
rs16978075	18	42113151	C	T	0.110	0.027	0.003	4.4e-15
rs55874505	18	43816804	G	T	0.584	-0.019	0.002	3.4e-18
rs9947760	18	48148741	C	T	0.372	0.021	0.002	1.6e-21
rs527544	18	51762866	T	G	0.424	0.016	0.002	4.4e-13
rs72933640	18	60054077	C	G	0.107	-0.021	0.003	1.8e-09
rs17758695	18	60920854	T	C	0.029	-0.062	0.006	6.6e-23
rs763361	18	67531642	C	T	0.527	-0.013	0.002	3.2e-10
rs72973711	18	74072245	T	A	0.067	-0.033	0.004	1.7e-14
rs4807440	19	1026477	T	G	0.638	0.026	0.002	3.4e-30
rs873289	19	1175396	G	A	0.474	-0.013	0.002	2.7e-10
rs2358581	19	10391611	G	T	0.734	-0.029	0.002	1.2e-33
rs895819	19	13947292	C	T	0.337	0.020	0.002	6.7e-18
rs59326120	19	16504352	C	T	0.156	0.054	0.003	1.5e-75
rs57070010	19	17650608	C	G	0.112	0.022	0.003	3.2e-11
rs4805881	19	33896432	C	A	0.665	-0.016	0.002	4.3e-13
rs2194067	19	35464727	T	C	0.417	0.014	0.002	9.8e-11
rs4760	19	44153100	G	A	0.156	-0.070	0.003	5.8e-127
rs11673093	19	45742094	A	G	0.260	-0.054	0.002	1.4e-108
rs177918	19	47634313	G	T	0.894	-0.032	0.003	2e-20
rs35112940	19	51738917	A	G	0.218	-0.024	0.003	1.8e-20
rs6045615	20	1931582	C	A	0.328	-0.018	0.002	3.4e-15
rs2294256	20	8174058	T	A	0.482	-0.015	0.002	4.3e-13
rs6086540	20	8603950	C	G	0.507	-0.023	0.002	1e-27
rs34952318	20	11177055	A	G	0.049	-0.034	0.005	5e-12
rs2185558	20	36905894	C	T	0.140	0.019	0.003	1.5e-09
rs6029234	20	39259278	C	G	0.626	0.025	0.002	2.5e-29

rs1800961	20	43042364	T	C	0.031	-0.042	0.006	5.1e-12
rs143003731	20	47936696	T	C	0.008	-0.074	0.013	4e-09
rs1060402	20	49205095	A	G	0.518	-0.017	0.002	5.1e-16
rs1997577	21	16371102	T	A	0.155	-0.019	0.003	2.5e-10
rs9977672	21	40463283	A	G	0.258	-0.021	0.002	2.5e-18
rs73377344	21	47804358	C	T	0.554	-0.017	0.002	1.8e-14
rs740422	22	17619292	T	C	0.218	0.016	0.003	1.3e-09
rs5746451	22	18126020	C	T	0.504	0.020	0.002	7.3e-22
rs2240717	22	19969106	G	A	0.350	0.022	0.002	3.6e-22
rs13055027	22	24641953	T	G	0.276	-0.019	0.002	3.1e-15
rs62227042	22	29632882	T	A	0.416	-0.019	0.002	1.1e-17
rs9680589	22	31853484	A	G	0.051	-0.029	0.005	2.2e-09
rs139402	22	39546145	C	T	0.438	-0.022	0.002	1.3e-24
rs47340	22	43562829	T	G	0.396	0.015	0.002	1.5e-12

Table S3. 367 SNPs significantly associated with eosinophil count used IVs in forward MR analyses derived from Vuckovic D et al. Chr: Chromosome; EA: Effect allele; NEA: Non-effect allele; EAF: Effect allele frequency.

SNP	Chr	Pos	EA	NEA	EAF	Beta	SE	P-value
rs12097268	1	2477358	A	T	0.482	-0.013	0.002	3.2e-10
rs301802	1	8497307	A	T	0.584	0.019	0.002	2.5e-18
rs1294028	1	9364634	G	A	0.610	0.015	0.002	8.2e-12
rs9430574	1	9709072	A	G	0.325	-0.021	0.002	4.1e-20
rs35249183	1	12099345	G	A	0.100	0.040	0.004	5.1e-29
rs2502994	1	24199363	C	T	0.569	0.021	0.002	1.9e-22
rs10794666	1	25250830	T	C	0.573	0.017	0.002	3e-16
rs6699190	1	26531789	T	G	0.663	-0.014	0.002	3.1e-10
rs79516637	1	28608721	A	G	0.067	-0.032	0.004	4.1e-14
rs1004870	1	42370787	T	C	0.589	0.026	0.002	4e-34
rs567910619	1	65210790	T	C	0.011	-0.088	0.011	4.9e-16
rs12408934	1	65423447	A	G	0.103	-0.042	0.003	4.8e-33
rs41313381	1	79411968	A	C	0.031	0.049	0.006	3e-16
rs6672038	1	87751670	T	C	0.119	0.037	0.003	3.2e-29
rs1088318	1	93733057	T	C	0.379	-0.023	0.002	7e-26
rs74431709	1	101653125	G	T	0.111	-0.042	0.003	7.4e-35
rs1494316	1	111319655	C	G	0.235	-0.016	0.002	1.1e-10
rs2793925	1	120652158	A	T	0.684	0.017	0.002	3.7e-13
rs11204682	1	150595537	T	G	0.223	-0.042	0.003	6.4e-60
rs111548294	1	150911337	T	C	0.013	0.088	0.010	2e-20
rs34448954	1	161168189	T	C	0.106	-0.028	0.003	6.5e-16
rs6672031	1	172796505	G	C	0.487	0.019	0.002	4.3e-19
rs12137747	1	173213790	A	C	0.469	0.018	0.002	8.3e-17
rs6658698	1	181060791	A	G	0.618	-0.015	0.002	1.6e-11
rs17849502	1	183532580	T	G	0.052	-0.029	0.005	1e-09
rs2296618	1	198666232	G	A	0.136	-0.039	0.003	9.3e-37
rs1036332	1	199012478	C	A	0.738	-0.032	0.002	8e-41
rs10793767	1	204549564	C	A	0.675	0.014	0.002	6.6e-10
rs41298997	1	206655331	T	C	0.235	0.016	0.002	4.8e-11
rs59150958	1	221106304	T	C	0.153	-0.027	0.003	1.2e-19
rs12142635	1	226976332	A	C	0.057	-0.030	0.005	2e-10
rs11810108	1	229668671	C	T	0.229	-0.017	0.003	1.8e-11
rs72755295	1	242034263	G	A	0.033	-0.037	0.006	3.2e-10
rs10165200	2	8439629	A	G	0.371	-0.025	0.002	5.2e-30
rs62105489	2	8757326	T	C	0.053	-0.032	0.005	1.3e-11
rs11127154	2	28685967	T	A	0.492	-0.024	0.002	9.8e-28
rs11890306	2	28691488	T	C	0.423	0.013	0.002	2.3e-09
rs17682575	2	43152304	T	C	0.202	-0.019	0.003	2.2e-12
rs113542380	2	43464818	A	G	0.075	-0.028	0.004	6.2e-12
rs778756	2	61781994	G	A	0.580	-0.016	0.002	8.4e-14
rs7569084	2	65656969	T	C	0.585	0.019	0.002	1.7e-18
rs12622600	2	71279940	T	C	0.270	-0.016	0.002	4.6e-11
rs11126424	2	74262353	C	T	0.306	-0.015	0.002	3.4e-11
rs2366640	2	85810734	C	A	0.319	-0.016	0.002	1.6e-12
rs2579505	2	97167962	C	T	0.638	0.044	0.002	1.5e-90
rs13418548	2	102917239	T	C	0.138	-0.093	0.003	1e-200
rs1465641	2	106438936	T	C	0.385	0.015	0.002	1.3e-11
rs72836346	2	111876613	C	G	0.078	0.071	0.004	1.2e-68
rs4848139	2	112418002	T	C	0.525	0.018	0.002	3.5e-18
rs11678022	2	136809033	T	A	0.731	-0.019	0.002	1.4e-14
rs79716587	2	143886819	A	G	0.126	-0.025	0.003	5.5e-15

rs1427499	2	145400317	G	A	0.710	0.019	0.002	1.1e-15
rs10195713	2	158520905	T	C	0.864	0.028	0.003	1.5e-18
rs10930337	2	169695031	T	C	0.285	0.014	0.002	4e-09
rs6731125	2	182308836	C	T	0.565	0.018	0.002	1.9e-16
rs10174238	2	191973034	A	G	0.766	0.015	0.003	3.5e-09
rs1519602	2	197027792	G	T	0.649	0.015	0.002	5.8e-11
rs13021247	2	207926818	A	G	0.315	-0.014	0.002	1.4e-09
rs715	2	211543055	C	T	0.311	0.018	0.002	4.3e-15
rs12619285	2	213824045	G	A	0.265	-0.064	0.002	1.2e-156
rs35409523	2	213908457	A	G	0.076	0.052	0.004	7.4e-38
rs7423615	2	231116874	T	C	0.187	0.023	0.003	5.5e-18
rs1057258	2	234115629	T	C	0.178	-0.032	0.003	5.4e-31
rs34290285	2	242698640	A	G	0.256	-0.046	0.002	8.8e-80
rs76474320	2	242806022	C	T	0.078	0.031	0.004	3.5e-15
rs1695315	3	3153339	A	G	0.605	-0.036	0.002	9.3e-60
rs4684037	3	10228040	G	C	0.143	-0.020	0.003	6.3e-11
rs310747	3	12259545	G	T	0.625	-0.030	0.002	9.9e-42
rs76643909	3	12441367	G	T	0.150	0.027	0.003	5.4e-20
rs13073683	3	25393837	C	T	0.399	0.016	0.002	3.6e-13
rs1353286	3	27772014	G	T	0.453	0.020	0.002	2.1e-21
rs7646283	3	33046480	T	C	0.367	0.026	0.002	8.2e-32
rs2228467	3	42906116	C	T	0.061	0.062	0.004	1.7e-45
rs4072859	3	49032205	C	G	0.659	0.027	0.002	9.3e-30
rs4677079	3	72125863	T	G	0.498	0.014	0.002	1.7e-11
rs7649812	3	98449191	G	C	0.242	0.017	0.002	3.1e-12
rs4618204	3	101281534	C	T	0.444	0.018	0.002	4.3e-17
rs528288236	3	111807413	A	G	0.019	-0.047	0.008	2.3e-09
rs9868582	3	112052927	T	G	0.452	-0.024	0.002	2e-29
rs2399441	3	112587012	C	T	0.350	-0.019	0.002	2.8e-18
rs6806253	3	128282697	G	A	0.164	-0.088	0.003	1e-200
rs142062847	3	128395208	C	T	0.437	0.018	0.002	6.3e-14
rs2712399	3	128432953	G	C	0.409	-0.025	0.002	6e-30
rs73238201	3	142204728	T	C	0.179	-0.017	0.003	3.9e-10
rs1516527	3	148609704	C	T	0.951	-0.033	0.005	2.6e-11
rs4680250	3	150941492	G	C	0.694	-0.016	0.002	1.9e-11
rs76830965	3	159637678	A	C	0.118	-0.023	0.003	7.3e-12
rs191674933	3	169488691	T	A	0.240	0.025	0.002	1.3e-23
rs4464488	3	171498211	T	C	0.414	-0.014	0.002	8.9e-11
rs4074672	3	183730295	T	C	0.369	0.015	0.002	2.8e-12
rs9815874	3	188441161	T	C	0.299	0.029	0.002	3.7e-35
rs9818987	3	194402430	T	C	0.306	0.014	0.002	2.9e-09
rs12152276	3	196368501	G	A	0.081	-0.035	0.004	4.8e-20
rs4916555	3	196538648	T	C	0.475	0.018	0.002	9.3e-18
rs1828803	4	2689449	A	C	0.393	0.013	0.002	1.2e-09
rs6448432	4	26098810	A	G	0.306	0.014	0.002	1.8e-09
rs73232881	4	38664131	C	T	0.214	0.069	0.003	1.3e-156
rs6855379	4	39037026	G	C	0.584	-0.016	0.002	6e-13
rs2412771	4	57761417	C	T	0.417	-0.013	0.002	1.3e-09
rs17005891	4	83547862	A	G	0.184	-0.055	0.003	2.9e-90
rs13105682	4	102702364	G	T	0.059	-0.029	0.005	3.4e-10
rs113473633	4	103449131	G	A	0.026	-0.063	0.007	1.5e-19
rs62324212	4	123560939	A	C	0.437	0.021	0.002	5.4e-22
rs6836610	4	123601697	A	G	0.303	0.019	0.002	6.3e-17
rs13120371	4	139092719	G	A	0.327	0.016	0.002	3.4e-13
rs11931711	4	153291513	T	C	0.282	0.021	0.002	1.6e-18
rs10062687	5	10624866	G	T	0.233	0.025	0.003	2e-22

rs16903574	5	14610309	G	C	0.076	0.030	0.004	1.7e-13
rs4594881	5	35846815	T	G	0.341	-0.036	0.002	8.1e-58
rs4703589	5	72097351	C	T	0.531	0.013	0.002	2.1e-10
rs4703730	5	76549688	T	C	0.517	-0.017	0.002	4.6e-16
rs34495	5	98265807	T	G	0.304	-0.019	0.002	6e-17
rs10455025	5	110404999	C	A	0.355	0.041	0.002	8.6e-75
rs35936927	5	110768362	G	A	0.522	-0.018	0.002	2.4e-14
rs17516457	5	131590387	C	T	0.417	-0.065	0.002	1e-200
rs375509059	5	131960939	T	C	0.046	0.045	0.005	2.2e-18
rs256869	5	132135372	C	T	0.085	0.023	0.004	1.8e-09
rs17653687	5	133468000	G	A	0.179	-0.019	0.003	1.3e-11
rs10062349	5	141509597	A	G	0.617	0.040	0.002	1.9e-76
rs55977204	5	142501095	C	T	0.113	0.021	0.003	3.5e-10
rs56330463	5	148200011	C	T	0.552	0.041	0.002	3.3e-81
rs6869605	5	150452866	C	A	0.123	-0.024	0.003	3.6e-14
rs2431097	5	159890885	T	C	0.485	0.014	0.002	5.9e-11
rs62385501	5	171950231	A	T	0.308	-0.015	0.002	1.5e-10
rs6556313	5	176792491	G	A	0.332	0.020	0.002	3.8e-18
rs2666969	6	336410	G	A	0.254	-0.042	0.003	2.5e-38
rs9504361	6	577820	G	A	0.446	-0.032	0.002	9.4e-49
rs186984319	6	16702251	C	T	0.317	-0.014	0.002	4.2e-09
rs3132682	6	30044388	C	G	0.588	-0.055	0.002	5.1e-144
rs9266321	6	31330360	C	G	0.184	0.048	0.003	9.6e-69
rs111936749	6	31523012	T	C	0.252	0.041	0.003	1.7e-34
rs62395789	6	31572801	A	G	0.044	0.042	0.005	8.2e-16
rs529454848	6	32525948	A	T	0.146	-0.036	0.004	4.7e-22
rs28383314	6	32587213	C	T	0.625	0.063	0.002	5e-184
rs9462094	6	35513603	T	C	0.431	-0.023	0.002	3.4e-20
rs11751949	6	42219296	A	G	0.071	-0.031	0.004	1e-13
rs76236447	6	42225993	C	G	0.072	0.039	0.004	5.1e-21
rs9395112	6	45682806	G	A	0.156	0.020	0.003	7.9e-12
rs1095671	6	88016300	A	G	0.571	-0.015	0.002	6.1e-13
rs62408224	6	90955995	G	A	0.351	-0.041	0.002	1.3e-77
rs62420764	6	106799970	C	T	0.140	0.019	0.003	8.2e-10
rs12208103	6	107442431	T	C	0.376	-0.031	0.002	1.8e-44
rs1915834	6	109597624	A	C	0.480	0.014	0.002	3.6e-11
rs9389268	6	135419631	G	A	0.260	-0.047	0.002	1.1e-82
rs6924387	6	137082948	G	A	0.410	0.017	0.002	1.8e-15
rs113496608	6	138161666	A	G	0.030	-0.043	0.006	6.7e-12
rs13207791	6	143249749	G	A	0.089	0.024	0.004	2.6e-10
rs149110519	6	144385777	T	C	0.036	0.042	0.006	4.7e-13
rs577734383	7	2881732	A	G	0.236	0.016	0.003	4.3e-10
rs6971710	7	3140173	A	G	0.201	0.030	0.003	3.1e-29
rs13245462	7	8074608	T	C	0.102	-0.021	0.004	3e-09
rs10950642	7	17016646	A	G	0.363	-0.019	0.002	3.5e-18
rs57834782	7	20502828	A	T	0.245	-0.059	0.002	2.6e-125
rs12537614	7	22789551	C	G	0.592	-0.028	0.002	1.4e-37
rs60600003	7	37382465	G	T	0.101	0.041	0.004	1.3e-31
rs150640087	7	50444152	T	G	0.016	0.099	0.009	5e-31
rs17450294	7	50763459	C	T	0.086	-0.031	0.004	5e-16
rs13226583	7	75454152	T	A	0.116	-0.062	0.003	5.8e-78
rs55879743	7	75470364	T	C	0.066	0.074	0.004	3.6e-65
rs62473720	7	77254547	G	A	0.318	0.014	0.002	6.3e-10
rs4272	7	92236829	G	A	0.211	0.028	0.003	8.7e-28
rs445	7	92408370	T	C	0.096	-0.053	0.004	7.5e-49
rs56072276	7	98778222	A	G	0.688	-0.016	0.002	5.6e-12

rs12538988	7	101810359	A	C	0.291	0.018	0.002	4.2e-14
rs12705849	7	112782556	A	G	0.407	-0.021	0.002	4.7e-23
rs3807307	7	128579202	C	T	0.464	-0.016	0.002	1e-14
rs56179563	7	129685597	A	G	0.389	0.018	0.002	6.7e-16
rs3110791	7	135367828	C	T	0.624	0.015	0.002	7.6e-12
rs12530946	7	148887942	G	A	0.614	0.043	0.002	6.1e-85
rs4871849	8	22964719	A	G	0.708	-0.033	0.002	1.7e-45
rs17377462	8	27281912	A	G	0.248	-0.017	0.002	1.5e-11
rs2133480	8	47923945	A	C	0.094	0.029	0.004	3.8e-15
rs45577137	8	48651633	G	A	0.045	-0.061	0.005	5.2e-29
rs7846314	8	61650831	T	A	0.187	-0.033	0.003	1.4e-34
rs6989099	8	66898262	C	T	0.317	-0.018	0.002	5.6e-15
rs4739140	8	79753010	T	C	0.610	-0.016	0.002	2.6e-13
rs10957978	8	81285139	T	G	0.647	-0.016	0.002	1e-12
rs2011566	8	95971921	A	G	0.512	0.013	0.002	1.1e-09
rs12681644	8	119972974	T	C	0.211	-0.021	0.003	3.3e-15
rs4870977	8	127526842	C	G	0.871	-0.019	0.003	8.9e-10
rs11786536	8	129000416	A	G	0.164	-0.028	0.003	1.9e-22
rs7840212	8	130599247	T	C	0.336	-0.040	0.002	1.8e-70
rs4236746	8	130699861	G	A	0.975	0.056	0.007	2.3e-16
rs117961539	8	144989466	A	G	0.039	-0.033	0.006	3.9e-09
rs34173062	8	145158607	A	G	0.072	0.054	0.004	5.8e-35
rs460631	9	4851440	G	A	0.885	0.027	0.003	6.6e-16
rs62541534	9	5028921	G	C	0.259	0.032	0.002	1.5e-40
rs4142528	9	6172296	A	T	0.672	-0.076	0.002	1e-200
rs1547258	9	6523056	C	T	0.708	-0.020	0.002	1.3e-17
rs10962679	9	16905441	C	T	0.755	-0.015	0.002	4.1e-10
rs7036656	9	21990457	T	C	0.722	0.018	0.002	9.3e-15
rs9410887	9	86465395	C	T	0.253	0.022	0.002	3e-19
rs1331691	9	94129137	C	T	0.184	-0.030	0.003	2.7e-27
rs496475	9	113638236	G	T	0.387	0.035	0.002	1.5e-58
rs7040707	9	117680732	G	A	0.048	-0.034	0.005	5.6e-10
rs911603	9	117697584	A	C	0.403	-0.025	0.002	2.9e-29
rs2773818	9	135859519	A	C	0.447	0.014	0.002	5.2e-11
rs74612091	9	135877278	A	T	0.063	0.060	0.004	4.9e-43
rs115478735	9	136149711	T	A	0.184	-0.028	0.003	1.5e-24
rs72766638	9	136931778	A	C	0.164	-0.025	0.003	2.8e-18
rs72775768	9	139324574	T	C	0.289	0.022	0.002	2.5e-20
rs11253517	10	970524	A	G	0.238	0.038	0.002	1.2e-51
rs1323650	10	6033049	T	G	0.681	-0.014	0.002	1.8e-09
rs10905281	10	8109583	A	C	0.176	0.021	0.003	2.9e-14
rs2646438	10	8565675	A	G	0.566	-0.021	0.002	3.6e-22
rs1444782	10	9058671	A	G	0.423	-0.040	0.002	2.1e-79
rs2992335	10	26727934	G	C	0.598	-0.035	0.002	1.1e-57
rs2807742	10	28781367	A	G	0.770	0.038	0.003	8.7e-51
rs17482472	10	44859618	A	G	0.099	-0.034	0.004	3.5e-21
rs16917546	10	64397538	C	T	0.369	-0.045	0.002	5.5e-93
rs72834751	10	64559465	T	C	0.013	-0.069	0.010	6.2e-12
rs10998535	10	70805373	T	C	0.215	0.016	0.003	1.2e-09
rs564443	10	89807917	A	G	0.163	0.024	0.003	1.2e-16
rs7897422	10	90849704	C	T	0.191	0.021	0.003	7.1e-15
rs7918084	10	94429467	T	C	0.550	0.030	0.002	4.7e-45
rs11591571	10	104342804	A	G	0.339	0.016	0.002	4.1e-13
rs17126931	10	111754633	C	T	0.129	-0.022	0.003	1e-11
rs7080536	10	115348046	A	G	0.043	-0.046	0.005	4.4e-18
rs1059091	11	309127	G	A	0.321	0.034	0.002	9.8e-51

rs9666598	11	325386	G	C	0.843	-0.021	0.003	4.2e-13
rs58833930	11	2325997	T	C	0.113	-0.024	0.003	2.5e-12
rs34439695	11	33901483	T	C	0.035	-0.041	0.006	3.4e-12
rs3824867	11	47468569	G	A	0.712	0.018	0.002	1.2e-14
rs3016175	11	60830367	G	A	0.881	0.021	0.003	4.7e-10
rs174548	11	61571348	G	C	0.314	-0.023	0.002	9e-24
rs689274	11	65665988	G	A	0.543	0.033	0.002	4e-54
rs7936323	11	76293758	A	G	0.478	0.041	0.002	3.2e-83
rs11236813	11	76343427	C	G	0.101	-0.031	0.004	5.6e-18
rs4409785	11	95311422	C	T	0.172	0.018	0.003	6.2e-11
rs637064	11	108140909	T	C	0.555	0.025	0.002	4.4e-32
rs964184	11	116648917	C	G	0.867	0.028	0.003	7.5e-20
rs7123726	11	118694547	C	T	0.210	0.019	0.003	2.2e-13
rs10893844	11	128185850	C	G	0.502	0.024	0.002	4.5e-30
rs605093	11	128604232	T	G	0.473	-0.017	0.002	5.9e-16
rs12820863	12	4318723	T	C	0.351	0.022	0.002	1.2e-22
rs1800692	12	6442346	G	A	0.587	-0.019	0.002	8e-19
rs3759332	12	6491078	C	T	0.391	-0.013	0.002	3.8e-09
rs7302975	12	32138924	C	T	0.779	-0.023	0.003	7.3e-19
rs12581511	12	46573788	G	C	0.172	-0.018	0.003	1.6e-10
rs10876550	12	54712308	A	G	0.559	0.014	0.002	1.4e-11
rs1689510	12	56396768	C	G	0.338	0.027	0.002	6.7e-34
rs3024971	12	57493727	G	T	0.107	-0.039	0.003	6.7e-30
rs10859277	12	92521198	T	C	0.536	-0.024	0.002	1.4e-28
rs12313790	12	94632933	A	C	0.085	-0.025	0.004	1.1e-10
rs1530161	12	96804649	G	A	0.433	0.013	0.002	1.1e-09
rs73191842	12	110986507	G	T	0.022	0.057	0.007	2.3e-15
rs1265564	12	111708458	C	A	0.441	0.082	0.002	1e-200
rs182686482	12	112350744	C	T	0.015	0.104	0.011	1.9e-21
rs373582220	12	112847812	A	G	0.372	-0.041	0.003	7e-51
rs1557865	12	113342598	G	T	0.817	-0.017	0.003	1.8e-09
rs1169286	12	121419056	C	T	0.426	-0.013	0.002	7.2e-10
rs28866344	12	123885484	G	A	0.907	-0.028	0.004	1.2e-14
rs12861824	13	31079184	C	T	0.273	-0.020	0.003	3.2e-15
rs7986796	13	40351064	T	G	0.627	0.025	0.002	5e-30
rs34373380	13	41244217	A	T	0.305	0.037	0.002	1.3e-58
rs9533117	13	43046812	T	A	0.239	-0.019	0.002	1.8e-14
rs201798	13	50954721	A	G	0.615	0.021	0.002	6.6e-22
rs7327960	13	74682892	C	T	0.826	0.017	0.003	6.7e-10
rs9557201	13	99989538	G	A	0.272	0.019	0.002	2.6e-15
rs912131	13	100032346	G	A	0.702	0.027	0.002	4.3e-32
rs9590475	13	114961483	A	G	0.413	-0.016	0.002	1.4e-13
rs10137343	14	23382640	T	A	0.604	0.013	0.002	3.4e-09
rs2239633	14	23589057	A	G	0.484	0.035	0.002	7.9e-61
rs2004925	14	25445362	A	G	0.393	0.016	0.002	2.1e-13
rs8020739	14	35882492	T	G	0.643	0.025	0.002	2.6e-28
rs12432697	14	38053334	T	G	0.254	-0.016	0.002	1.3e-10
rs6573020	14	55871452	T	C	0.434	0.021	0.002	1.3e-22
rs7152277	14	65535391	C	G	0.399	0.016	0.002	3.8e-13
rs1296535	14	69297986	T	C	0.779	-0.018	0.003	1.7e-12
rs1861160	14	73365712	A	G	0.317	0.015	0.002	2e-11
rs175705	14	75975648	G	C	0.719	0.039	0.002	8.7e-61
rs67856193	14	93024616	G	C	0.308	0.024	0.002	9.7e-26
rs117068593	14	93118229	T	C	0.189	-0.025	0.003	3e-20
rs11555542	14	94417531	C	T	0.063	0.066	0.004	4.5e-51
rs11628569	14	103190362	G	A	0.290	-0.018	0.002	1.9e-14

rs4906317	14	103814500	T	G	0.504	-0.014	0.002	6.4e-10
rs62006172	15	38904527	A	G	0.042	-0.038	0.005	3.9e-13
rs7173571	15	41712807	C	T	0.526	0.014	0.002	1.5e-10
rs34212866	15	43702964	G	A	0.221	0.022	0.003	1e-17
rs11638256	15	58772807	C	T	0.120	0.020	0.003	2.6e-09
rs11637887	15	60765216	T	C	0.193	-0.016	0.003	4.7e-09
rs16943117	15	61078836	T	C	0.192	-0.018	0.003	5.9e-11
rs3101486	15	67346220	T	G	0.765	0.018	0.003	2.8e-13
rs17293632	15	67442596	T	C	0.236	0.030	0.003	3e-33
rs7498094	15	68486097	T	C	0.519	-0.013	0.002	1.6e-09
rs6494871	15	70767978	T	C	0.595	0.015	0.002	3.1e-12
rs2115535	15	80195199	A	G	0.568	0.034	0.002	4.3e-56
rs1138358	15	80263345	C	A	0.264	-0.030	0.002	1.7e-36
rs13511	15	86284817	C	T	0.577	-0.017	0.002	1.7e-14
rs5011652	15	91166608	C	G	0.759	0.020	0.002	2.9e-15
rs4785903	16	2838274	C	T	0.755	-0.034	0.002	7.1e-43
rs9934736	16	3519252	G	A	0.047	0.031	0.005	6.8e-10
rs7186106	16	11210831	A	G	0.354	-0.047	0.002	5.2e-97
rs2072130	16	27356398	T	C	0.365	0.030	0.002	3.7e-41
rs7201518	16	30082508	T	G	0.480	-0.030	0.002	5.3e-45
rs17881535	16	31108709	G	C	0.240	-0.017	0.002	2.5e-12
rs9924483	16	48646258	T	C	0.310	-0.023	0.002	7.3e-24
rs1684578	16	57069650	T	G	0.416	0.017	0.002	1.5e-14
rs2161647	16	57503330	A	C	0.037	-0.041	0.006	2.1e-13
rs12918121	16	67187795	T	C	0.018	-0.047	0.008	4.4e-09
rs570765844	16	67618243	C	T	0.080	0.026	0.004	8.4e-11
rs1170439	16	68608511	C	T	0.779	0.025	0.003	4.4e-23
rs13339407	16	78156295	G	A	0.306	-0.014	0.002	2.1e-09
rs301161	16	85810349	A	G	0.850	0.027	0.003	7e-20
rs12932970	16	86004110	C	T	0.352	0.016	0.002	2.6e-13
rs17175830	16	88558164	A	G	0.237	0.033	0.003	2.8e-39
rs112036266	17	2834143	T	C	0.176	0.017	0.003	4.4e-10
rs34210653	17	4535314	A	G	0.021	-0.176	0.007	1.6e-124
rs74480102	17	7742601	A	G	0.043	-0.084	0.005	1.1e-57
rs12941068	17	17737857	A	G	0.285	-0.015	0.002	2e-10
rs58745116	17	33803592	A	G	0.392	-0.015	0.002	2.1e-11
rs79989390	17	37962845	A	G	0.034	-0.072	0.006	4.4e-35
rs72836561	17	41926126	T	C	0.031	-0.054	0.006	4.2e-19
rs1971374	17	43659738	C	T	0.213	-0.033	0.003	6.4e-38
rs12952581	17	47448346	A	G	0.362	0.029	0.002	8.1e-39
rs4968392	17	57799204	G	A	0.841	0.020	0.003	9.4e-12
rs7215391	17	64232022	T	C	0.257	-0.014	0.002	4.9e-09
rs62086903	17	66016006	C	T	0.231	0.030	0.003	1.7e-32
rs180506	17	68274205	A	G	0.776	-0.023	0.003	4e-20
rs78057960	17	73779075	T	C	0.296	-0.018	0.002	8.7e-15
rs12946146	17	81079703	T	C	0.486	0.016	0.002	1.2e-12
rs2847266	18	12773338	T	C	0.717	-0.015	0.002	5.9e-10
rs12968697	18	20625431	T	C	0.627	0.017	0.002	2.4e-15
rs9955853	18	43829655	T	C	0.559	-0.013	0.002	4.9e-09
rs57633475	18	46469650	G	A	0.122	-0.026	0.003	1.1e-15
rs637716	18	48481308	A	G	0.589	-0.013	0.002	8.9e-10
rs73963711	18	60874413	T	C	0.210	0.023	0.003	9.4e-18
rs954954	18	60902328	C	A	0.105	-0.035	0.003	1.7e-23
rs17758695	18	60920854	T	C	0.029	-0.121	0.006	1.7e-82
rs1395269	18	61377644	G	T	0.301	-0.024	0.002	1.1e-25
rs3848640	19	1037986	G	A	0.329	-0.016	0.002	1.3e-11

rs36084354	19	1079959	A	G	0.092	-0.044	0.004	1.1e-33
rs3746165	19	1102211	G	A	0.469	-0.014	0.002	5.4e-11
rs61731111	19	3179517	T	C	0.011	-0.131	0.010	1.5e-37
rs34466956	19	3353622	T	C	0.568	-0.013	0.002	2.2e-09
rs410867	19	16427111	G	A	0.216	-0.061	0.003	2.2e-122
rs3786586	19	16495287	G	A	0.155	0.032	0.003	6.9e-27
rs8108623	19	18408519	A	C	0.626	0.018	0.002	4.9e-14
rs2043293	19	19751456	A	C	0.826	-0.017	0.003	2.3e-09
rs117710327	19	33726578	A	C	0.067	-0.036	0.004	9.1e-17
rs412884	19	40219449	C	T	0.672	0.057	0.002	1.1e-140
rs62117160	19	45232161	A	G	0.045	-0.048	0.005	1.1e-20
rs73036520	19	45749484	C	G	0.254	-0.031	0.002	7.9e-36
rs113577760	19	46365177	C	T	0.091	-0.040	0.004	3.3e-27
rs6077868	20	10649761	T	C	0.206	-0.016	0.003	5.9e-10
rs3790315	20	17630045	T	C	0.580	-0.018	0.002	9e-17
rs80054178	20	30294682	C	T	0.023	-0.084	0.007	1e-31
rs6141755	20	31163565	T	G	0.236	-0.016	0.003	8e-11
rs6017247	20	42657841	C	T	0.729	-0.025	0.002	1.5e-24
rs6013210	20	50165634	G	C	0.118	-0.023	0.003	1.9e-12
rs7264802	20	62692440	G	A	0.748	-0.016	0.002	2.9e-10
rs2223043	21	16438793	G	A	0.305	0.020	0.002	9.1e-17
rs11701443	21	16931215	C	T	0.202	0.017	0.003	3.7e-10
rs1000005	21	34433051	C	G	0.591	-0.020	0.002	3.1e-20
rs34872427	21	36294044	C	T	0.415	-0.020	0.002	7.7e-21
rs2242886	21	36387806	T	C	0.071	-0.102	0.004	1.4e-132
rs9979383	21	36715761	T	C	0.630	0.030	0.002	1.1e-42
rs150229150	21	45641853	T	C	0.125	-0.021	0.003	5.1e-11
rs11703539	22	17594755	A	G	0.102	-0.022	0.003	4.3e-10
rs5747308	22	18133500	C	A	0.505	0.015	0.002	1.2e-12
rs7288670	22	24621826	G	A	0.306	-0.025	0.002	2.6e-28
rs140074	22	31725634	G	C	0.486	0.023	0.002	2.4e-26
rs41280005	22	37316133	A	G	0.167	-0.029	0.003	1e-23
rs228951	22	37529798	G	A	0.363	-0.022	0.002	3.7e-22
rs2413631	22	41359786	A	G	0.250	0.015	0.002	1.6e-09
rs34780507	22	41405753	G	A	0.066	-0.059	0.004	1.4e-43
rs78023664	22	46647844	A	G	0.092	-0.027	0.004	5.3e-13

Table S4. 388 SNPs significantly associated with eosinophil count used IVs in forward MR analyses derived from Chen MH et al. Chr: Chromosome; EA: Effect allele; NEA: Non-effect allele; EAF: Effect allele frequency.

SNP	Chr	Pos	EA	NEA	EAF	Beta	SE	P-value
rs2419313	10	111750851	A	G	0.143	-0.021	0.003	5.21e-13
rs7080536	10	115348046	A	G	0.043	-0.044	0.005	1.17e-18
rs2992333	10	26727454	A	G	0.599	-0.034	0.002	1.35e-63
rs2807740	10	28784483	T	C	0.770	0.035	0.002	1.05e-50
rs17482472	10	44859618	A	G	0.099	-0.031	0.003	5.34e-21
rs1323650	10	6033049	T	G	0.681	-0.014	0.002	2.53e-10
rs10995240	10	64388631	C	G	0.368	-0.044	0.002	1.55e-101
rs72834751	10	64559465	T	C	0.013	-0.072	0.009	1.5e-14
rs3747869	10	73520632	C	A	0.901	0.020	0.003	2.31e-09
rs4746153	10	75598282	C	G	0.185	0.016	0.003	8.58e-10
rs11255507	10	8109615	G	T	0.178	0.020	0.003	1.05e-14
rs2646438	10	8565675	A	G	0.566	-0.021	0.002	1.2e-24
rs495149	10	89795523	T	C	0.164	0.024	0.003	1.15e-19
rs962993	10	9053132	T	C	0.422	-0.040	0.002	4.92e-90
rs7897422	10	90849704	C	T	0.192	0.021	0.003	1.85e-16
rs2497318	10	94432000	T	C	0.443	-0.030	0.002	1.96e-49
rs1539174	10	974870	G	C	0.237	0.038	0.002	1.12e-59
rs637064	11	108140909	T	C	0.556	0.024	0.002	5.43e-34
rs964184	11	116648917	C	G	0.867	0.029	0.003	4.39e-23
rs10893844	11	128185850	C	G	0.502	0.025	0.002	3.49e-35
rs668248	11	128586262	C	G	0.616	-0.017	0.002	5.49e-16
rs214080	11	17299762	G	A	0.580	0.013	0.002	1.37e-10
rs58833930	11	2325997	T	C	0.113	-0.023	0.003	1.03e-13
rs1059091	11	309127	G	A	0.321	0.034	0.002	3.08e-56
rs9666598	11	325386	G	C	0.843	-0.020	0.003	1.36e-12
rs34439695	11	33901483	T	C	0.035	-0.038	0.005	3.67e-12
rs80116434	11	57110622	A	G	0.142	-0.020	0.003	9.09e-12
rs174548	11	61571348	G	C	0.314	-0.023	0.002	4.08e-27
rs11231727	11	64011854	T	C	0.431	-0.012	0.002	1.34e-09
rs634534	11	65665256	G	A	0.542	0.033	0.002	5.83e-61
rs7936434	11	76293805	C	G	0.478	0.043	0.002	4.08e-104
rs11236814	11	76343428	T	A	0.102	-0.030	0.003	3.46e-20
rs4409785	11	95311422	C	T	0.172	0.016	0.003	4.25e-10
rs11571404	12	1041450	T	C	0.203	0.015	0.002	5.57e-10
rs73202462	12	110266430	A	G	0.020	0.057	0.008	2.14e-13
rs11065822	12	111600134	T	G	0.354	0.065	0.002	1e-200
rs115647629	12	111811903	A	G	0.026	-0.067	0.007	6.69e-25
rs6490291	12	112177775	A	T	0.964	-0.066	0.006	1.11e-29
rs146730870	12	112332996	A	G	0.010	-0.087	0.010	4.95e-17
rs148219449	12	113339873	A	G	0.014	0.064	0.010	6.02e-11
rs28532037	12	123883406	A	G	0.907	-0.030	0.003	4.19e-18
rs4931002	12	32143169	A	C	0.779	-0.022	0.002	1.73e-19
rs12820863	12	4318723	T	C	0.352	0.022	0.002	1.07e-25
rs12581511	12	46573788	G	C	0.173	-0.018	0.003	2.07e-11
rs1689510	12	56396768	C	G	0.338	0.027	0.002	1.06e-36
rs3024971	12	57493727	G	T	0.107	-0.040	0.003	5.04e-36
rs1800692	12	6442346	G	A	0.588	-0.018	0.002	8.92e-19
rs10777378	12	92518680	A	G	0.536	-0.022	0.002	1.28e-29
rs10745763	12	96811386	T	G	0.423	0.013	0.002	2.41e-11
rs12100034	13	114956773	A	G	0.359	-0.017	0.002	2.6e-16
rs12861824	13	31079184	C	T	0.275	-0.021	0.002	2.59e-18

rs7986796	13	40351064	T	G	0.628	0.024	0.002	4.91e-32
rs6563842	13	41244297	T	G	0.306	0.036	0.002	3.39e-61
rs71429414	13	42945821	A	G	0.196	-0.019	0.002	1.03e-13
rs201798	13	50954721	A	G	0.615	0.020	0.002	5.71e-23
rs7327960	13	74682892	C	T	0.827	0.018	0.003	1.91e-11
rs2182885	13	99855124	A	G	0.605	0.026	0.002	2.05e-38
rs11628569	14	103190362	G	A	0.290	-0.017	0.002	4.7e-14
rs2239633	14	23589057	A	G	0.484	0.035	0.002	1.5e-68
rs7141943	14	25438250	G	A	0.395	0.016	0.002	7e-15
rs8020739	14	35882492	T	G	0.644	0.025	0.002	2.57e-32
rs8012643	14	37692864	T	C	0.286	0.015	0.002	1.91e-12
rs6573020	14	55871452	T	C	0.433	0.021	0.002	2.31e-24
rs8011585	14	65522223	A	T	0.397	0.016	0.002	1.5e-14
rs1296535	14	69297986	T	C	0.777	-0.019	0.002	3.35e-15
rs12878610	14	73389048	C	T	0.476	-0.013	0.002	3.95e-11
rs175705	14	75975648	G	C	0.719	0.038	0.002	8.8e-67
rs67856193	14	93024616	G	C	0.308	0.024	0.002	1.63e-27
rs117068593	14	93118229	T	C	0.190	-0.026	0.003	1.17e-24
rs11555542	14	94417531	C	T	0.063	0.064	0.004	2.12e-54
rs62006172	15	38904527	A	G	0.042	-0.038	0.005	4.62e-14
rs34212866	15	43702964	G	A	0.221	0.020	0.002	5.74e-17
rs11071559	15	61069988	T	C	0.131	-0.022	0.003	4.94e-14
rs62011287	15	63791228	G	A	0.344	-0.013	0.002	1.32e-09
rs17293632	15	67442596	T	C	0.236	0.030	0.002	8.92e-37
rs7257	15	80191343	A	G	0.567	0.034	0.002	3.6e-63
rs8026803	15	80260014	C	T	0.261	-0.031	0.002	1.67e-43
rs13511	15	86284817	C	T	0.577	-0.016	0.002	4.13e-16
rs725613	16	11169683	G	T	0.356	-0.047	0.002	4.78e-116
rs121564	16	20717360	T	G	0.270	-0.017	0.002	1.26e-13
rs3785356	16	27349168	T	C	0.298	0.030	0.002	1.7e-43
rs4280242	16	2830482	T	C	0.754	-0.033	0.002	1.03e-45
rs9939774	16	30068354	T	C	0.405	-0.029	0.002	1.98e-47
rs1039341	16	48574869	T	C	0.311	-0.024	0.002	8.79e-29
rs1684578	16	57069650	T	G	0.416	0.016	0.002	5.15e-16
rs16956811	16	57444002	G	T	0.081	-0.029	0.004	1.75e-15
rs8050508	16	67303358	T	C	0.031	0.035	0.006	1.01e-09
rs1170439	16	68608511	C	T	0.779	0.023	0.002	7.86e-21
rs8044920	16	69838676	T	C	0.376	0.012	0.002	3.59e-09
rs3850107	16	78150341	G	C	0.294	-0.014	0.002	3.94e-10
rs17689159	16	79742390	C	T	0.315	-0.014	0.002	1.4e-11
rs301162	16	85810647	G	A	0.853	0.026	0.003	1.16e-20
rs17175830	16	88558164	A	G	0.238	0.032	0.002	4.13e-42
rs61426394	16	9053519	C	G	0.067	-0.025	0.004	6.99e-10
rs12941068	17	17737857	A	G	0.286	-0.014	0.002	9.59e-10
rs112036266	17	2834143	T	C	0.176	0.017	0.003	1.48e-10
rs58745116	17	33803592	A	G	0.391	-0.015	0.002	8.21e-14
rs79989390	17	37962845	A	G	0.035	-0.069	0.005	3.28e-37
rs145947882	17	41809207	C	A	0.026	-0.060	0.006	2.98e-21
rs34210653	17	4535314	A	G	0.021	-0.176	0.007	8.11e-140
rs9889262	17	47398070	A	T	0.364	0.028	0.002	4.18e-43
rs72831272	17	57448441	A	G	0.029	0.035	0.006	3.16e-09
rs11869228	17	57800358	T	C	0.841	0.021	0.003	3.45e-14
rs62086903	17	66016006	C	T	0.232	0.030	0.002	2.27e-37
rs180506	17	68274205	A	G	0.776	-0.023	0.002	5.82e-22
rs55868524	17	7170665	A	G	0.606	0.013	0.002	9.91e-11
rs8076052	17	73779198	C	A	0.296	-0.018	0.002	4.33e-17

rs74480102	17	7742601	A	G	0.043	-0.083	0.005	2.52e-63
rs9709216	17	81081455	G	T	0.487	0.018	0.002	1.21e-16
rs397187	17	8784759	C	T	0.430	-0.012	0.002	1.28e-09
rs2847266	18	12773338	T	C	0.717	-0.014	0.002	1.22e-10
rs9675999	18	20627691	A	G	0.627	0.016	0.002	1.59e-15
rs57633475	18	46469650	G	A	0.122	-0.025	0.003	6.36e-16
rs2850542	18	48403560	T	G	0.556	-0.013	0.002	6.5e-11
rs73963711	18	60874413	T	C	0.211	0.022	0.002	3.28e-19
rs954954	18	60902328	C	A	0.105	-0.035	0.003	3.57e-27
rs17758695	18	60920854	T	C	0.030	-0.122	0.006	1.32e-93
rs7241187	18	61375974	C	T	0.301	-0.024	0.002	9.38e-28
rs36084354	19	1079959	A	G	0.092	-0.045	0.003	1.17e-39
rs4588110	19	1102139	G	A	0.469	-0.015	0.002	3.37e-13
rs410867	19	16427111	G	A	0.217	-0.059	0.002	4.12e-134
rs3786586	19	16495287	G	A	0.155	0.032	0.003	4.42e-31
rs8108623	19	18408519	A	C	0.625	0.018	0.002	1.91e-16
rs1529745	19	19517325	G	C	0.175	0.016	0.003	2.9e-09
rs61731111	19	3179517	T	C	0.011	-0.132	0.010	7.57e-43
rs34466956	19	3353622	T	C	0.568	-0.014	0.002	2.6e-11
rs118013485	19	33726577	A	G	0.067	-0.034	0.004	1.54e-16
rs412884	19	40219449	C	T	0.672	0.058	0.002	2.22e-164
rs62117160	19	45232161	A	G	0.045	-0.048	0.005	2.15e-23
rs117955557	19	45755368	T	G	0.256	-0.030	0.002	1.44e-40
rs76793172	19	46359794	T	C	0.092	-0.042	0.003	8.07e-34
rs2967592	19	8598280	C	T	0.122	-0.018	0.003	3.29e-09
rs111759324	1	101652522	T	C	0.111	-0.043	0.003	3.56e-42
rs1160413	1	111315694	G	A	0.240	-0.015	0.002	2.97e-10
rs35249183	1	12099345	G	A	0.100	0.040	0.003	1.46e-32
rs11204682	1	150595537	T	G	0.223	-0.041	0.002	4.89e-67
rs71628184	1	160596935	T	C	0.094	0.021	0.003	8.32e-10
rs34448954	1	161168189	T	C	0.106	-0.025	0.003	3.87e-15
rs6672031	1	172796505	G	C	0.486	0.020	0.002	1.9e-23
rs1099448	1	173231898	T	C	0.466	0.018	0.002	8.32e-19
rs4652560	1	181057188	T	A	0.618	-0.015	0.002	5.23e-14
rs17849502	1	183532580	T	G	0.052	-0.027	0.004	1.29e-09
rs17668272	1	198623842	T	G	0.118	-0.042	0.003	7.48e-42
rs1036332	1	199012478	C	A	0.737	-0.033	0.002	1.61e-47
rs1414517	1	221092838	G	C	0.187	-0.025	0.003	1.36e-22
rs708776	1	226923505	T	G	0.941	0.027	0.004	2.91e-10
rs4148757	1	229693846	C	A	0.210	-0.017	0.002	2.24e-12
rs3218148	1	23851787	A	G	0.540	-0.018	0.002	7.08e-19
rs2502995	1	24199290	C	T	0.570	0.022	0.002	1.06e-27
rs4149909	1	242023898	G	A	0.033	-0.033	0.006	2.07e-09
rs12097268	1	2477358	A	T	0.482	-0.013	0.002	3.23e-11
rs113105190	1	28614481	C	T	0.068	-0.033	0.004	5.19e-17
rs4310436	1	39489221	A	G	0.120	0.019	0.003	9.15e-10
rs1004870	1	42370787	T	C	0.589	0.026	0.002	1.27e-38
rs77625297	1	65362766	C	G	0.035	-0.046	0.006	2.79e-16
rs12408934	1	65423447	A	G	0.103	-0.041	0.003	2.36e-36
rs41313381	1	79411968	A	C	0.031	0.050	0.006	7.05e-19
rs159963	1	8504421	A	C	0.582	0.019	0.002	9.88e-22
rs6684992	1	87752000	T	A	0.119	0.038	0.003	6.06e-35
rs4908835	1	9357460	C	T	0.161	0.019	0.003	1.8e-12
rs661126	1	93678968	G	T	0.624	0.023	0.002	9.02e-29
rs4529711	1	9709735	C	T	0.320	-0.020	0.002	3.7e-20
rs3790163	20	10647951	G	A	0.792	-0.015	0.003	1.35e-09

rs6080761	20	17629162	A	G	0.419	0.018	0.002	4.75e-20
rs80054178	20	30294682	C	T	0.022	-0.082	0.007	8.57e-34
rs6141755	20	31163565	T	G	0.236	-0.017	0.002	8.3e-13
rs6103572	20	42657862	C	T	0.729	-0.024	0.002	5.96e-27
rs3746420	20	50140627	C	G	0.061	-0.032	0.004	2.26e-14
rs2223043	21	16438793	G	A	0.307	0.019	0.002	3.23e-17
rs11701475	21	16931347	C	T	0.223	0.015	0.002	1.06e-09
rs11088236	21	34416187	T	C	0.453	0.018	0.002	5.17e-20
rs28421324	21	36286938	T	A	0.102	0.036	0.003	1.57e-27
rs56117721	21	36398586	A	T	0.072	-0.104	0.004	6.64e-160
rs114152720	21	36434894	A	G	0.030	-0.036	0.006	8.08e-10
rs9979383	21	36715761	T	C	0.630	0.028	0.002	8e-43
rs2838317	21	44991791	C	A	0.550	0.013	0.002	1.2e-10
rs7354779	21	45670770	C	T	0.271	0.018	0.002	2.71e-15
rs11703539	22	17594755	A	G	0.102	-0.022	0.003	2.82e-11
rs5747308	22	18133500	C	A	0.505	0.016	0.002	4.82e-15
rs7288670	22	24621826	G	A	0.306	-0.026	0.002	4.51e-34
rs739427	22	31659101	C	G	0.512	-0.022	0.002	1.06e-29
rs60175411	22	37311858	A	G	0.167	-0.030	0.003	1.25e-28
rs743002	22	41404939	C	T	0.066	-0.059	0.004	2.9e-48
rs73176685	22	41781094	G	C	0.243	0.024	0.002	1.07e-24
rs8142080	22	46647429	T	G	0.102	-0.024	0.003	5.72e-13
rs72998585	2	102858490	T	A	0.134	-0.091	0.003	1e-200
rs6736701	2	106390440	T	G	0.409	0.013	0.002	1.98e-10
rs72836346	2	111876613	C	G	0.079	0.070	0.004	4.38e-76
rs4848139	2	112418002	T	C	0.525	0.019	0.002	4.82e-21
rs925966	2	136806959	G	C	0.759	-0.018	0.002	1.32e-14
rs79716587	2	143886819	A	G	0.127	-0.024	0.003	3.99e-16
rs1427499	2	145400317	G	A	0.710	0.019	0.002	1.3e-17
rs1406449	2	148495285	C	T	0.462	-0.012	0.002	8.69e-10
rs10195713	2	158520905	T	C	0.864	0.025	0.003	2.74e-17
rs10930337	2	169695031	T	C	0.285	0.015	0.002	2.03e-11
rs6731125	2	182308836	C	T	0.565	0.017	0.002	1.04e-17
rs1519602	2	197027792	G	T	0.648	0.013	0.002	7.43e-10
rs62183994	2	204614872	T	C	0.051	0.027	0.005	2.65e-09
rs715	2	211543055	C	T	0.311	0.018	0.002	3.1e-16
rs6750754	2	213830187	G	T	0.264	-0.064	0.002	8.35e-179
rs35409523	2	213908457	A	G	0.076	0.050	0.004	1.43e-40
rs2713548	2	227156662	T	C	0.633	0.013	0.002	1.02e-09
rs7423615	2	231116874	T	C	0.187	0.023	0.003	5.1e-20
rs1057258	2	234115629	T	C	0.178	-0.032	0.003	2.52e-35
rs34290285	2	242698640	A	G	0.257	-0.046	0.002	4.73e-91
rs76474320	2	242806022	C	T	0.077	0.028	0.004	5.76e-14
rs80066203	2	26220710	T	C	0.068	-0.024	0.004	8.53e-10
rs11695281	2	28686568	T	C	0.514	-0.023	0.002	4.09e-31
rs6753015	2	28875673	T	A	0.315	-0.013	0.002	2.42e-09
rs10165678	2	30446568	A	G	0.758	-0.015	0.002	1.9e-10
rs17682575	2	43152304	T	C	0.201	-0.018	0.002	1.33e-12
rs149290349	2	43451957	A	G	0.075	-0.027	0.004	1.18e-12
rs778756	2	61781994	G	A	0.580	-0.016	0.002	1.57e-15
rs7569084	2	65656969	T	C	0.584	0.019	0.002	1.19e-20
rs346835	2	8438693	T	C	0.328	-0.027	0.002	7.55e-37
rs62105489	2	8757326	T	C	0.053	-0.030	0.004	1.13e-11
rs56268488	2	95902245	A	G	0.086	-0.021	0.004	4.14e-09
rs2579505	2	97167962	C	T	0.638	0.042	0.002	5.79e-94
rs4618204	3	101281534	C	T	0.444	0.018	0.002	1.09e-19

rs4684037	3	10228040	G	C	0.144	-0.021	0.003	4.24e-13
rs111413886	3	111821047	A	G	0.031	-0.040	0.006	3.16e-12
rs1131199	3	112059768	G	C	0.530	-0.025	0.002	9.55e-36
rs2399441	3	112587012	C	T	0.351	-0.019	0.002	8.08e-20
rs10222469	3	121977125	C	A	0.819	0.017	0.003	4.95e-11
rs310747	3	12259545	G	T	0.625	-0.029	0.002	1.65e-44
rs6802327	3	12410892	T	G	0.146	0.027	0.003	2.43e-21
rs62270951	3	128225277	G	A	0.037	-0.043	0.005	1.92e-16
rs73238201	3	142204728	T	C	0.179	-0.016	0.003	1.52e-10
rs1516527	3	148609704	C	T	0.951	-0.032	0.005	2.25e-12
rs4680250	3	150941492	G	C	0.693	-0.016	0.002	4.85e-13
rs76830965	3	159637678	A	C	0.118	-0.023	0.003	3.27e-14
rs4464488	3	171498211	T	C	0.415	-0.014	0.002	1.99e-12
rs4074672	3	183730295	T	C	0.369	0.015	0.002	1.48e-12
rs9815874	3	188441161	T	C	0.300	0.028	0.002	1.93e-39
rs9818987	3	194402430	T	C	0.306	0.014	0.002	2.6e-10
rs7636495	3	196367936	A	G	0.181	-0.024	0.003	1.84e-20
rs2089979	3	196501413	G	A	0.416	-0.015	0.002	1.25e-13
rs13073683	3	25393837	C	T	0.399	0.016	0.002	1.32e-14
rs1353286	3	27772014	G	T	0.453	0.020	0.002	3.09e-24
rs1695315	3	3153339	A	G	0.605	-0.034	0.002	1.56e-61
rs7646283	3	33046480	T	C	0.368	0.026	0.002	2.33e-36
rs2228467	3	42906116	C	T	0.062	0.062	0.004	8.83e-52
rs13316620	3	49045355	C	T	0.659	0.027	0.002	1.16e-33
rs12487980	3	71760041	A	C	0.634	0.013	0.002	1.5e-09
rs7646695	3	72172181	T	C	0.180	0.018	0.003	1.82e-11
rs7649812	3	98449191	G	C	0.243	0.016	0.002	3.74e-12
rs13105682	4	102702364	G	T	0.060	-0.027	0.004	8.22e-10
rs113473633	4	103449131	G	A	0.026	-0.063	0.007	3.47e-22
rs6827756	4	123184411	C	T	0.638	-0.020	0.002	5.39e-23
rs2390353	4	123602097	C	T	0.232	0.023	0.002	3.04e-22
rs13120371	4	139092719	G	A	0.327	0.015	0.002	3e-13
rs11931711	4	153291513	T	C	0.282	0.019	0.002	3.48e-18
rs7441808	4	26090375	G	A	0.301	0.014	0.002	1.22e-10
rs1828803	4	2689449	A	C	0.392	0.013	0.002	1.28e-10
rs73232881	4	38664131	C	T	0.213	0.067	0.002	8.22e-171
rs2566133	4	39022515	C	T	0.584	-0.014	0.002	1.06e-12
rs62308111	4	57738491	T	G	0.221	0.015	0.002	1.5e-10
rs7687708	4	6906076	G	T	0.220	-0.015	0.002	1.56e-09
rs13138355	4	83545976	T	C	0.186	-0.055	0.003	2.35e-102
rs10062687	5	10624866	G	T	0.233	0.024	0.002	9.47e-25
rs139640694	5	109938754	G	A	0.093	0.025	0.003	3.54e-13
rs79881201	5	110427795	T	C	0.360	0.040	0.002	4.72e-85
rs10059018	5	110501604	T	G	0.201	-0.023	0.002	3.38e-21
rs17516457	5	131590387	C	T	0.417	-0.064	0.002	1e-200
rs137906075	5	131972916	G	A	0.046	0.043	0.005	1.39e-18
rs17653687	5	133468000	G	A	0.179	-0.018	0.003	4.06e-12
rs7700687	5	141491985	T	C	0.617	0.040	0.002	4.82e-86
rs55977204	5	142501095	C	T	0.113	0.020	0.003	3.6e-10
rs16903574	5	14610309	G	C	0.078	0.029	0.004	1.17e-13
rs56330463	5	148200011	C	T	0.553	0.039	0.002	5.38e-86
rs73272842	5	150453888	A	G	0.123	-0.025	0.003	4.84e-17
rs2431097	5	159890885	T	C	0.486	0.015	0.002	5.95e-14
rs62385501	5	171950231	A	T	0.308	-0.014	0.002	1.68e-11
rs6556313	5	176792491	G	A	0.332	0.019	0.002	2.16e-20
rs1961220	5	35844125	A	G	0.340	-0.035	0.002	2.64e-63

rs4703589	5	72097351	C	T	0.533	0.014	0.002	3.26e-13
rs4703730	5	76549688	T	C	0.517	-0.016	0.002	6.91e-16
rs34495	5	98265807	T	G	0.304	-0.020	0.002	3.67e-20
rs62420764	6	106799970	C	T	0.140	0.019	0.003	4.21e-11
rs12208103	6	107442431	T	C	0.378	-0.031	0.002	6.83e-50
rs783646	6	117286220	C	G	0.817	-0.019	0.003	2.41e-13
rs9389268	6	135419631	G	A	0.256	-0.045	0.002	3.69e-87
rs6924387	6	137082948	G	A	0.410	0.017	0.002	2.19e-16
rs113496608	6	138161666	A	G	0.030	-0.045	0.006	1.13e-14
rs13207791	6	143249749	G	A	0.089	0.026	0.004	4.3e-13
rs149110519	6	144385777	T	C	0.036	0.038	0.005	1.39e-12
rs3093023	6	167534290	A	G	0.434	0.013	0.002	2.27e-10
rs41546314	6	29910378	T	C	0.290	0.060	0.002	1.29e-166
rs6929022	6	33562447	G	A	0.282	-0.029	0.002	1.39e-40
rs10456428	6	35012302	T	C	0.235	-0.023	0.002	1.17e-22
rs56987668	6	35319253	C	T	0.037	-0.045	0.005	1.21e-17
rs3793084	6	42142244	C	T	0.183	0.015	0.003	2.89e-09
rs9349322	6	45634092	C	A	0.147	0.022	0.003	4.04e-15
rs9504361	6	577820	G	A	0.447	-0.032	0.002	7.98e-56
rs138305038	6	88014416	G	C	0.474	-0.016	0.002	2.07e-15
rs62408224	6	90955995	G	A	0.350	-0.042	0.002	5.69e-92
rs73187850	7	101867824	T	A	0.278	0.018	0.002	8.69e-17
rs12705849	7	112782556	A	G	0.407	-0.021	0.002	2.55e-24
rs7797428	7	124886883	T	C	0.469	0.012	0.002	5.85e-10
rs3823536	7	128579666	A	G	0.467	-0.018	0.002	3.16e-19
rs56179563	7	129685597	A	G	0.389	0.018	0.002	3.87e-18
rs3110791	7	135367828	C	T	0.623	0.015	0.002	7.49e-14
rs12530946	7	148887942	G	A	0.613	0.043	0.002	3.99e-97
rs12540285	7	150312474	G	A	0.221	0.015	0.002	1.01e-09
rs6979947	7	157005863	G	A	0.266	0.013	0.002	3.75e-09
rs10950642	7	17016646	A	G	0.363	-0.019	0.002	4.15e-20
rs61210140	7	20503007	A	G	0.245	-0.058	0.002	7.12e-141
rs4722171	7	22785717	G	A	0.592	-0.027	0.002	1.83e-41
rs6971710	7	3140173	A	G	0.201	0.028	0.002	2.75e-30
rs60600003	7	37382465	G	T	0.100	0.043	0.003	3.05e-38
rs1037674	7	50217850	T	G	0.292	0.013	0.002	1.63e-09
rs13226583	7	75454152	T	A	0.116	-0.062	0.003	1.59e-88
rs55879743	7	75470364	T	C	0.066	0.078	0.004	7.07e-82
rs62473720	7	77254547	G	A	0.317	0.014	0.002	2.64e-11
rs56195338	7	8107922	A	G	0.058	-0.030	0.004	1.88e-12
rs4272	7	92236829	G	A	0.212	0.028	0.002	2.68e-30
rs445	7	92408370	T	C	0.097	-0.053	0.003	2.84e-56
rs12681644	8	119972974	T	C	0.212	-0.019	0.002	1.23e-15
rs13251643	8	126240768	T	A	0.044	0.030	0.005	3.54e-10
rs4870977	8	127526842	C	G	0.871	-0.019	0.003	2.4e-10
rs11786536	8	129000416	A	G	0.165	-0.027	0.003	5.11e-23
rs7840212	8	130599247	T	C	0.337	-0.040	0.002	7.11e-81
rs4236746	8	130699861	G	A	0.975	0.057	0.006	6.78e-19
rs117961539	8	144989466	A	G	0.040	-0.031	0.005	2.63e-09
rs34173062	8	145158607	A	G	0.073	0.055	0.004	1.55e-40
rs3208305	8	19823648	T	A	0.287	0.013	0.002	8.64e-10
rs4871849	8	22964719	A	G	0.707	-0.033	0.002	1.06e-51
rs10088702	8	27263107	T	C	0.264	-0.017	0.002	2.1e-13
rs45577137	8	48651633	G	A	0.045	-0.060	0.005	5.1e-31
rs62539154	8	48677930	A	G	0.097	0.027	0.003	7.2e-16
rs7846314	8	61650831	T	A	0.187	-0.033	0.003	3.92e-38

rs6989099	8	66898262	C	T	0.317	-0.018	0.002	8.58e-17
rs2887502	8	79756591	T	C	0.590	-0.014	0.002	1.74e-11
rs6986109	8	81286298	T	G	0.704	-0.016	0.002	4.24e-14
rs4240624	8	9184231	A	G	0.909	-0.020	0.003	3.62e-09
rs574183	8	95973816	G	A	0.389	-0.014	0.002	1.2e-11
rs7026022	9	102562216	C	A	0.378	0.012	0.002	3.38e-09
rs496475	9	113638236	G	T	0.387	0.035	0.002	1.83e-66
rs911603	9	117697584	A	C	0.404	-0.025	0.002	1.42e-33
rs74612091	9	135877278	A	T	0.063	0.061	0.004	1.23e-49
rs460631	9	4851440	G	A	0.885	0.026	0.003	1.88e-16
rs7868130	9	4998401	T	C	0.259	0.032	0.002	3.35e-46
rs4142528	9	6172296	A	T	0.672	-0.075	0.002	1e-200
rs1547258	9	6523056	C	T	0.708	-0.019	0.002	1.49e-18
rs295273	9	86466968	A	G	0.256	0.021	0.002	1.5e-19
rs6479336	9	94110288	A	T	0.185	-0.029	0.003	3.44e-30
rs78691875	12	113070264	C	A	0.979	0.051	0.007	1.69e-13
rs12928503	16	67377216	C	T	0.980	0.049	0.007	1.92e-11
rs151036504	6	31409578	C	T	0.985	-0.062	0.008	2.5e-14
rs150640087	7	50444152	G	T	0.984	-0.100	0.008	1.8e-35
rs10892296	11	118718729	T	C	0.195	0.018	0.003	2.63e-12
rs12577643	11	47467172	T	A	0.326	0.017	0.002	4.31e-15
rs11559982	12	54711574	G	A	0.556	0.014	0.002	1.92e-12
rs4906317	14	103814500	T	G	0.505	-0.015	0.002	9.44e-12
rs8010804	14	23396480	G	T	0.614	0.013	0.002	6.51e-10
rs4924544	15	41693455	T	G	0.525	0.015	0.002	4.26e-13
rs76758258	15	91190935	A	T	0.240	-0.020	0.002	2.4e-16
rs7184899	16	4472087	A	T	0.704	-0.014	0.002	1.16e-09
rs9906974	17	43822772	C	T	0.220	-0.034	0.002	8.37e-44
rs4789797	17	80527895	A	G	0.587	0.012	0.002	2.67e-09
rs3848640	19	1037986	G	A	0.329	-0.016	0.002	9.78e-13
rs2793925	1	120652158	A	T	0.684	0.017	0.002	7.93e-14
rs111548294	1	150911337	T	C	0.013	0.087	0.009	2.61e-21
rs12116645	1	204524474	T	G	0.675	0.016	0.002	6.31e-13
rs192397873	1	28002091	C	T	0.053	0.030	0.005	1.56e-10
rs2422631	20	1556758	C	T	0.739	-0.014	0.002	2.04e-09
rs7264802	20	62692440	G	A	0.749	-0.015	0.002	1.07e-10
rs12622600	2	71279940	T	C	0.270	-0.016	0.002	9.11e-12
rs11126424	2	74262353	C	T	0.306	-0.016	0.002	1.84e-12
rs2366640	2	85810734	C	A	0.319	-0.017	0.002	5.08e-14
rs112924476	3	128265131	A	G	0.119	-0.098	0.003	1e-200
rs6765218	3	128380332	G	A	0.254	0.045	0.002	2.65e-80
rs191674933	3	169488691	T	A	0.240	0.025	0.002	4.26e-25
rs186984319	6	16702251	C	T	0.318	-0.013	0.002	4.37e-09
rs9270464	6	32558914	G	A	0.345	-0.035	0.002	5.57e-52
rs9272400	6	32604898	A	G	0.655	0.055	0.002	5.11e-130
rs2666969	6	336410	G	A	0.256	-0.040	0.003	7.32e-38
rs77396612	7	138737519	C	A	0.025	0.040	0.007	4.33e-09
rs572698961	7	50802158	A	C	0.086	-0.031	0.004	4.86e-17
rs56072276	7	98778222	A	G	0.688	-0.016	0.002	6.88e-13
rs7040707	9	117680732	G	A	0.048	-0.031	0.005	1.69e-09
rs115478735	9	136149711	T	A	0.183	-0.028	0.003	1.1e-25
rs146671954	9	136934203	A	G	0.164	-0.025	0.003	2.1e-20
rs68142670	9	139324573	C	T	0.278	0.022	0.002	3.07e-21
rs10962679	9	16905441	C	T	0.754	-0.014	0.002	1.26e-09
rs7874405	9	21980944	T	C	0.712	0.018	0.002	9.15e-16
rs12339911	9	34845984	A	T	0.613	-0.013	0.002	6.18e-10

rs193101979	5	129836370	I	D	0.160	0.017	0.003	3e-09

Table S5. 146 SNPs significantly associated with basophil count used IVs in forward MR analyses derived from Vuckovic D et al. Chr: Chromosome; EA: Effect allele; NEA: Non-effect allele; EAF: Effect allele frequency.

SNP	Chr	Pos	EA	NEA	EAF	Beta	SE	P-value
rs35076930	1	17677636	T	C	0.293	0.021	0.003	1.3e-13
rs12097415	1	21506276	A	T	0.547	-0.021	0.002	9.3e-21
rs3917932	1	36943916	G	C	0.577	-0.015	0.002	1.3e-11
rs17613339	1	118139540	T	C	0.128	-0.022	0.003	6.7e-11
rs12143614	1	150932696	A	T	0.117	-0.023	0.003	5e-11
rs12075	1	159175354	A	G	0.579	0.031	0.002	2.5e-43
rs2926468	1	161612578	C	T	0.468	-0.019	0.002	5e-17
rs10798014	1	185394834	G	A	0.321	-0.014	0.002	4.7e-09
rs12123922	1	205140436	A	G	0.441	-0.031	0.002	1.5e-44
rs1086893	1	212563114	C	T	0.344	0.032	0.002	3.2e-43
rs10927074	1	236103965	C	T	0.892	0.088	0.004	4.7e-135
rs62105478	2	8735869	A	G	0.052	-0.031	0.005	8.9e-10
rs12470883	2	65651851	A	G	0.409	0.022	0.002	8.1e-23
rs4851592	2	103087641	T	C	0.303	0.017	0.002	1.5e-12
rs1427499	2	145400317	G	A	0.710	0.017	0.002	6.3e-12
rs78862952	2	146506667	C	T	0.092	0.026	0.004	2.4e-11
rs1424951	2	148533708	C	T	0.334	0.015	0.002	2.4e-10
rs4602187	2	181834018	T	C	0.683	0.015	0.002	2.2e-10
rs4667282	2	182312809	A	T	0.561	0.016	0.002	3e-13
rs73987603	2	213844822	A	G	0.166	0.024	0.003	1.2e-15
rs10716631	2	219138170	G	T	0.536	0.017	0.002	6e-14
rs4324460	3	3120542	G	T	0.219	0.017	0.003	8.3e-11
rs1669340	3	3198380	T	G	0.839	-0.025	0.003	1.1e-16
rs6766037	3	16908186	G	A	0.218	-0.041	0.003	1.1e-52
rs12497690	3	27795397	C	A	0.371	0.016	0.002	6.7e-12
rs3181077	3	46250652	T	C	0.718	-0.043	0.002	1.2e-69
rs13063578	3	47087837	A	T	0.401	0.017	0.002	2.4e-13
rs6782812	3	128317997	A	G	0.893	0.069	0.004	9.2e-82
rs9819371	3	141206800	T	C	0.065	-0.033	0.004	3.5e-13
rs1973791	3	187416634	A	C	0.617	-0.018	0.002	7.4e-15
rs13434265	3	196489422	T	C	0.415	-0.013	0.002	4.9e-09
rs11930388	4	9985884	T	C	0.211	-0.016	0.003	4.9e-09
rs10023310	4	79629863	C	T	0.601	0.017	0.002	3.3e-13
rs906149	4	83537777	G	C	0.221	-0.017	0.003	1.4e-10
rs55913164	4	87898930	C	T	0.190	-0.031	0.003	8.9e-29
rs374187209	4	103410817	A	T	0.453	-0.016	0.002	1e-11
rs10214237	5	35883734	C	T	0.275	-0.019	0.002	4.1e-14
rs34500	5	98294885	A	G	0.084	-0.028	0.004	1.7e-12
rs2271352	5	126091428	C	G	0.226	0.046	0.003	2.8e-66
rs6579875	5	150782574	T	C	0.389	-0.016	0.002	4.8e-12
rs2594836	5	173205318	A	G	0.722	-0.017	0.002	6.4e-12
rs62393575	6	7058516	C	G	0.053	-0.030	0.005	3.1e-09
rs1100575	6	22373710	A	G	0.545	0.022	0.002	9.3e-23
rs2524079	6	31242174	A	G	0.417	0.031	0.002	1.3e-44
rs9267893	6	32202221	T	A	0.210	-0.026	0.003	3.9e-21
rs141378803	6	32490016	A	G	0.233	0.027	0.003	6.4e-18
rs915125	6	82463376	T	C	0.279	-0.034	0.002	3.3e-43
rs72895231	6	82679396	A	C	0.596	-0.017	0.002	9e-14
rs6927569	6	109621494	C	T	0.523	0.019	0.002	1.3e-18
rs9376098	6	135499460	A	T	0.349	0.026	0.002	2e-28
rs73049252	7	8016602	A	G	0.059	-0.044	0.005	1.2e-20

rs2158799	7	28277107	G	C	0.610	0.016	0.002	2.2e-12
rs56388170	7	28724374	T	G	0.292	0.034	0.002	5.5e-44
rs149007767	7	50370254	T	C	0.162	0.031	0.003	4.4e-24
rs1186222	7	75247329	T	C	0.462	-0.027	0.002	1.9e-33
rs11974227	7	80222408	T	C	0.064	0.028	0.005	5.4e-10
rs8179	7	92236164	C	T	0.792	-0.034	0.003	3.5e-36
rs445	7	92408370	T	C	0.096	-0.087	0.004	2.5e-119
rs11772895	7	143081942	C	G	0.277	0.040	0.002	3.3e-58
rs9314614	8	6697731	G	C	0.533	0.033	0.002	7.1e-49
rs2738104	8	6792257	A	G	0.673	0.021	0.002	1.2e-18
rs10086568	8	6900336	A	G	0.321	-0.041	0.002	6.8e-67
rs877116	8	10712945	T	G	0.589	0.017	0.002	3.2e-14
rs9644063	8	22974450	C	T	0.823	-0.027	0.003	1.6e-20
rs45577137	8	48651633	G	A	0.045	-0.052	0.006	1.3e-19
rs16923637	8	59567885	T	G	0.286	0.014	0.002	3.9e-09
rs7818415	8	70727663	A	T	0.485	-0.014	0.002	1e-09
rs4541868	8	106590705	A	C	0.275	-0.018	0.002	3.9e-13
rs10956401	8	129002419	A	G	0.343	-0.017	0.002	2.7e-13
rs3731211	9	21986847	A	T	0.722	0.017	0.002	4.2e-12
rs12376511	9	22142756	C	T	0.162	-0.019	0.003	1.2e-10
rs2150052	9	113945067	T	A	0.505	-0.014	0.002	5.7e-10
rs11253511	10	964832	T	C	0.238	0.052	0.003	1.4e-87
rs11595895	10	26734518	A	C	0.403	0.024	0.002	2.7e-26
rs2998286	10	28780373	C	T	0.769	0.025	0.003	4.4e-21
rs1897191	10	77143467	G	A	0.617	-0.019	0.002	2.3e-16
rs56014906	10	102103508	G	A	0.368	-0.018	0.002	3.9e-15
rs11195959	10	114199287	A	G	0.096	0.023	0.004	1.8e-09
rs3781452	10	126355129	T	C	0.633	0.022	0.002	7.1e-21
rs1058900	11	308290	C	T	0.568	0.018	0.002	8.2e-15
rs12278324	11	3862058	A	C	0.619	0.016	0.002	2.3e-12
rs742631	11	32077472	T	C	0.282	-0.016	0.002	6.6e-11
rs531612	11	65705432	T	C	0.509	0.013	0.002	4.4e-09
rs74472890	11	72946279	C	T	0.049	0.119	0.005	4.7e-120
rs4753251	11	89654536	A	T	0.730	-0.016	0.003	9.2e-11
rs2606724	11	113957880	A	G	0.455	0.017	0.002	3.2e-13
rs10893845	11	128186882	G	T	0.502	-0.014	0.002	8.5e-11
rs2286599	12	6499533	A	G	0.142	0.020	0.003	3.4e-10
rs146970669	12	27103449	A	G	0.093	0.026	0.004	9.9e-12
rs117053853	12	51720047	A	G	0.011	0.124	0.011	2.8e-31
rs2118140	12	66698461	G	A	0.528	0.015	0.002	2.6e-11
rs75084335	12	76981753	G	C	0.069	0.035	0.004	3e-15
rs3184504	12	111884608	C	T	0.519	-0.029	0.002	3.1e-39
rs11064881	12	120146925	A	G	0.073	-0.033	0.004	5.5e-15
rs4475963	12	129302255	G	T	0.370	-0.017	0.002	2.4e-13
rs76428106	13	28604007	C	T	0.013	0.082	0.010	8.8e-16
rs149391212	14	23544999	T	C	0.016	0.069	0.009	1.8e-14
rs2239630	14	23589349	G	A	0.558	0.028	0.002	8e-37
rs114917114	14	35366971	G	A	0.353	0.014	0.002	2.7e-09
rs11158159	14	57857162	C	G	0.773	0.031	0.003	9.6e-30
rs72721631	14	75807762	A	C	0.238	-0.021	0.003	4.3e-16
rs2289511	14	88454910	A	G	0.582	-0.017	0.002	6.9e-15
rs79429038	15	42677739	A	G	0.133	0.023	0.003	3.3e-12
rs2070596	15	50545159	A	T	0.211	0.020	0.003	6.5e-13
rs3848148	15	64637536	C	T	0.943	0.035	0.005	4.1e-13
rs1847271	15	69632367	A	G	0.624	0.016	0.002	1.2e-12
rs7166645	15	83740277	A	G	0.387	0.015	0.002	1.1e-10

rs2074585	15	91009484	A	G	0.514	-0.036	0.002	2.8e-60
rs8026614	15	100051136	T	C	0.472	-0.014	0.002	9.5e-10
rs67175901	15	101748227	T	C	0.108	0.029	0.004	1.4e-16
rs875740	16	16123048	A	C	0.666	-0.019	0.002	6.7e-16
rs7196129	16	30471109	C	T	0.528	0.019	0.002	1.2e-16
rs12927351	16	74596618	A	C	0.198	0.017	0.003	8.8e-10
rs247830	16	84581925	C	T	0.304	0.015	0.002	1.6e-10
rs4782302	16	88524389	C	A	0.436	0.018	0.002	4.4e-16
rs7503461	17	2883320	T	C	0.319	-0.015	0.002	7.3e-10
rs12453682	17	37770005	T	C	0.695	-0.019	0.002	1.4e-14
rs12941811	17	38159335	C	T	0.578	-0.048	0.002	6.9e-102
rs8178414	17	56345363	T	C	0.013	0.088	0.010	3.9e-19
rs34097845	17	56358429	T	C	0.057	0.049	0.005	1.9e-23
rs1295927	17	57929535	G	A	0.447	-0.027	0.002	1.1e-33
rs2384952	17	72744512	C	T	0.539	-0.017	0.002	3.6e-13
rs479032	18	9850581	A	G	0.656	-0.016	0.002	1.1e-11
rs17758695	18	60920854	T	C	0.029	-0.104	0.007	1.6e-57
rs62132278	19	836654	T	C	0.256	-0.031	0.003	1.2e-32
rs2336068	19	5813519	C	A	0.276	0.015	0.003	7.4e-10
rs2967595	19	8566299	T	C	0.168	-0.030	0.003	3.3e-23
rs873636	19	16514771	G	A	0.308	0.024	0.002	2.6e-23
rs55990904	19	18302239	C	T	0.274	-0.016	0.002	1.4e-10
rs8113682	19	19743730	G	T	0.748	-0.023	0.003	1.4e-19
rs78744187	19	33754548	T	C	0.081	-0.088	0.004	8.3e-105
rs34158728	19	38903032	A	G	0.029	0.105	0.007	1.8e-56
rs431329	19	40221107	G	C	0.663	-0.017	0.002	6.9e-14
rs12459419	19	51728477	T	C	0.324	-0.016	0.002	3.1e-12
rs7250849	19	52158316	T	G	0.107	0.021	0.004	3.5e-09
rs6045612	20	1931001	T	C	0.263	-0.016	0.003	1.1e-10
rs4911102	20	31179500	T	C	0.234	-0.017	0.003	2.9e-10
rs6141333	20	31263324	A	C	0.147	0.025	0.003	1.6e-15
rs4931	20	43530234	C	A	0.271	-0.024	0.002	1.6e-21
rs6091176	20	49150510	T	C	0.261	0.015	0.003	2.1e-09
rs310631	20	62196253	A	C	0.411	0.014	0.002	1.1e-09
rs11702209	21	38808428	G	C	0.118	-0.031	0.003	6.3e-20
rs2836154	21	39485842	T	C	0.336	0.017	0.002	8.8e-14
rs5756822	22	38196474	T	C	0.556	-0.014	0.002	4.6e-10
rs761702	22	41404970	A	G	0.065	0.038	0.004	2.5e-17
rs5766582	22	45632528	T	C	0.475	-0.018	0.002	1.4e-16

Table S6. 166 SNPs significantly associated with basophil count used IVs in forward MR analyses derived from Chen MH et al. Chr: Chromosome; EA: Effect allele; NEA: Non-effect allele; EAF: Effect allele frequency.

SNP	Chr	Pos	EA	NEA	EAF	Beta	SE	P-value
rs61872688	10	101273546	A	G	0.381	-0.013	0.002	1.04e-09
rs10883782	10	104583932	G	A	0.169	0.018	0.003	1.28e-10
rs3781452	10	126355129	T	C	0.632	0.020	0.002	1.34e-20
rs11595895	10	26734518	A	C	0.403	0.024	0.002	1.23e-29
rs2998286	10	28780373	C	T	0.770	0.021	0.002	1.31e-17
rs7078507	10	77140864	G	A	0.609	-0.016	0.002	2.54e-14
rs2606724	11	113957880	A	G	0.453	0.016	0.002	1.33e-13
rs10893844	11	128185850	C	G	0.501	-0.015	0.002	4.22e-14
rs695113	11	128562098	T	C	0.696	0.017	0.002	3.93e-14
rs734095	11	2323198	G	C	0.113	-0.028	0.003	2.04e-17
rs6421984	11	305619	C	T	0.514	0.018	0.002	6.97e-19
rs742631	11	32077472	T	C	0.284	-0.017	0.002	4.83e-13
rs10835333	11	3957766	G	A	0.349	0.015	0.002	1.68e-11
rs10896064	11	65641033	C	G	0.532	0.013	0.002	8.01e-11
rs74472890	11	72946279	C	T	0.049	0.103	0.005	1.02e-103
rs4753251	11	89654536	A	T	0.728	-0.022	0.002	3.42e-20
rs3184504	12	111884608	C	T	0.519	-0.028	0.002	5.08e-43
rs11064881	12	120146925	A	G	0.074	-0.032	0.004	2.05e-16
rs4475963	12	129302255	G	T	0.370	-0.014	0.002	2.11e-10
rs146970669	12	27103449	A	G	0.094	0.022	0.004	2.43e-10
rs17860282	12	51740350	T	C	0.011	0.105	0.010	2.62e-26
rs2286599	12	6499533	A	G	0.142	0.023	0.003	1.15e-14
rs2118140	12	66698461	G	A	0.528	0.018	0.002	4.03e-19
rs75084335	12	76981753	G	C	0.069	0.028	0.004	3.75e-12
rs76428106	13	28604007	C	T	0.013	0.082	0.009	2.78e-18
rs2701863	13	41170140	T	C	0.306	0.052	0.007	1.74e-13
rs2239635	14	23588731	C	G	0.704	0.034	0.002	1.89e-52
rs11158159	14	57857162	C	G	0.770	0.028	0.003	4.59e-28
rs72721631	14	75807762	A	C	0.237	-0.019	0.002	1.6e-15
rs2289511	14	88454910	A	G	0.582	-0.016	0.002	3.3e-15
rs72697295	14	93069980	C	G	0.181	-0.018	0.003	8e-11
rs67175901	15	101748227	T	C	0.108	0.026	0.003	8.24e-15
rs79429038	15	42677739	A	G	0.132	0.021	0.003	1.33e-11
rs2070596	15	50545159	A	T	0.211	0.023	0.003	3.3e-19
rs62021606	15	64548734	G	T	0.051	-0.034	0.005	2e-13
rs7496362	15	65758874	G	C	0.366	-0.013	0.002	2.71e-09
rs12443468	15	81888088	G	A	0.226	0.015	0.002	3.04e-09
rs2074585	15	91009484	A	G	0.515	-0.031	0.002	2.71e-51
rs875740	16	16123048	A	C	0.665	-0.018	0.002	1.52e-16
rs7196129	16	30471109	C	T	0.529	0.017	0.002	7.19e-16
rs9928015	16	57570561	T	G	0.302	-0.015	0.002	2.01e-11
rs247833	16	84581684	A	G	0.249	0.016	0.002	1.42e-11
rs12447180	16	88517722	C	T	0.324	0.023	0.002	2.06e-26
rs7503461	17	2883320	T	C	0.319	-0.013	0.002	9.6e-10
rs12453682	17	37770005	T	C	0.695	-0.018	0.002	3.6e-16
rs12941811	17	38159335	C	T	0.578	-0.046	0.002	7.71e-111
rs8178414	17	56345363	T	C	0.013	0.074	0.009	4.48e-16
rs34097845	17	56358429	T	C	0.057	0.043	0.005	4.58e-21
rs1295927	17	57929535	G	A	0.448	-0.025	0.002	5.83e-34
rs2959356	18	23590203	G	A	0.298	0.014	0.002	4.14e-10
rs17758695	18	60920854	T	C	0.030	-0.125	0.006	1.47e-91

rs561102	18	9851388	T	C	0.655	-0.014	0.002	8.12e-11
rs2607278	19	16568197	T	C	0.306	0.023	0.002	1.79e-24
rs8113682	19	19743730	G	T	0.747	-0.022	0.002	6.41e-20
rs78744187	19	33754548	T	C	0.082	-0.130	0.004	1e-200
rs34158728	19	38903032	A	G	0.028	0.095	0.006	3.24e-53
rs12459419	19	51728477	T	C	0.323	-0.017	0.002	2.44e-14
rs7250849	19	52158316	T	G	0.107	0.022	0.003	1.01e-10
rs76427287	19	837190	C	T	0.254	-0.029	0.002	2.07e-31
rs12143614	1	150932696	A	T	0.117	-0.023	0.003	4.72e-13
rs12075	1	159175354	A	G	0.580	0.028	0.002	6.02e-41
rs17625587	1	198990494	A	G	0.264	0.015	0.002	1.25e-10
rs1086893	1	212563114	C	T	0.343	0.030	0.002	2.41e-43
rs4060971	1	21506930	T	C	0.551	-0.021	0.002	1.85e-23
rs10927074	1	236103965	C	T	0.892	0.079	0.003	3e-127
rs56043070	1	247719769	A	G	0.071	-0.026	0.004	6.04e-11
rs7515985	1	28997021	G	A	0.416	0.014	0.002	6.05e-11
rs1537061	1	87739219	C	T	0.099	-0.025	0.003	2.55e-13
rs6045612	20	1931001	T	C	0.264	-0.017	0.002	7.18e-13
rs4911102	20	31179500	T	C	0.234	-0.022	0.002	6.46e-19
rs6141781	20	31263342	T	C	0.147	0.033	0.003	4.12e-29
rs6029234	20	39259278	C	G	0.626	0.013	0.002	3.46e-09
rs16989483	20	43537200	C	T	0.274	-0.021	0.002	3.15e-20
rs6091176	20	49150510	T	C	0.261	0.014	0.002	4.8e-09
rs310631	20	62196253	A	C	0.412	0.012	0.002	4.42e-09
rs2834670	21	36280376	G	A	0.172	0.028	0.003	1.69e-18
rs138595256	21	36789420	G	C	0.026	0.064	0.007	1.82e-20
rs7285377	22	19987202	T	G	0.284	0.014	0.002	1.86e-10
rs34288539	22	24642009	T	C	0.274	-0.015	0.002	6.34e-11
rs5756822	22	38196474	T	C	0.556	-0.012	0.002	3.79e-09
rs34780507	22	41405753	G	A	0.066	0.035	0.004	2.96e-17
rs5766582	22	45632528	T	C	0.475	-0.017	0.002	1.77e-16
rs6543144	2	103092575	G	A	0.313	0.014	0.002	7.43e-10
rs62160676	2	112167931	C	T	0.296	-0.017	0.002	3.57e-13
rs1427499	2	145400317	G	A	0.710	0.021	0.002	1.57e-20
rs77785849	2	146580195	A	C	0.033	0.036	0.006	4.94e-10
rs1598207	2	148757870	G	A	0.303	0.020	0.002	1.41e-19
rs4602187	2	181834018	T	C	0.683	0.014	0.002	2.53e-10
rs1449263	2	182319301	T	C	0.556	0.016	0.002	5.15e-14
rs73987603	2	213844822	A	G	0.166	0.024	0.003	2.64e-18
rs13419763	2	219134950	T	C	0.562	0.016	0.002	4.43e-15
rs79140637	2	65084123	A	G	0.054	-0.027	0.005	2.09e-09
rs2028900	2	85767735	T	C	0.449	-0.014	0.002	1.25e-11
rs62105478	2	8735869	A	G	0.052	-0.032	0.005	1.09e-11
rs74535412	3	128297689	A	G	0.035	0.038	0.006	2.31e-11
rs2335235	3	128315725	C	G	0.108	-0.088	0.003	4.63e-157
rs9819371	3	141206800	T	C	0.065	-0.030	0.004	7.05e-13
rs7613595	3	16908518	C	A	0.218	-0.036	0.002	1.55e-48
rs6780544	3	187424334	A	G	0.623	-0.016	0.002	9.32e-15
rs2089979	3	196501413	G	A	0.416	-0.014	0.002	2.59e-11
rs12497690	3	27795397	C	A	0.371	0.015	0.002	6.48e-12
rs4324460	3	3120542	G	T	0.219	0.024	0.003	6.15e-22
rs1669340	3	3198380	T	G	0.839	-0.034	0.003	3.09e-34
rs3181077	3	46250652	T	C	0.718	-0.037	0.002	2.23e-59
rs11097787	4	103407342	T	C	0.403	-0.015	0.002	3.83e-12
rs906149	4	83537777	G	C	0.221	-0.019	0.002	5.16e-14
rs56406125	4	87940205	T	G	0.190	-0.035	0.003	6.04e-41

rs2271352	5	126091428	C	G	0.226	0.042	0.002	1.44e-65
rs449454	5	141533062	G	A	0.617	0.013	0.002	1.56e-09
rs2594836	5	173205318	A	G	0.722	-0.018	0.002	1.87e-14
rs13188960	5	35853319	T	G	0.279	-0.019	0.002	5.08e-17
rs9376098	6	135499460	A	T	0.349	0.023	0.002	4.15e-27
rs1205896	6	22341469	A	G	0.504	0.024	0.002	4.46e-32
rs1029239	6	30138162	G	C	0.468	-0.014	0.002	2.17e-12
rs2308557	6	31239417	T	C	0.351	0.031	0.002	1.34e-46
rs1883638	6	34830754	T	C	0.162	-0.017	0.003	1.43e-09
rs62393575	6	7058516	C	G	0.053	-0.031	0.005	1.33e-11
rs915125	6	82463376	T	C	0.281	-0.029	0.002	2.18e-37
rs905670	6	90958502	A	G	0.350	-0.014	0.002	2.49e-11
rs56179563	7	129685597	A	G	0.389	0.015	0.002	6.1e-12
rs11772895	7	143081942	C	G	0.278	0.034	0.002	9.23e-50
rs2158799	7	28277107	G	C	0.610	0.014	0.002	2.57e-11
rs56388170	7	28724374	T	G	0.293	0.031	0.002	3.34e-43
rs149007767	7	50370254	T	C	0.162	0.027	0.003	3.48e-20
rs11768817	7	80223839	G	A	0.064	0.025	0.004	1.22e-09
rs8179	7	92236164	C	T	0.791	-0.028	0.003	2.75e-29
rs445	7	92408370	T	C	0.097	-0.076	0.003	2.69e-105
rs4541868	8	106590705	A	C	0.276	-0.019	0.002	2.61e-16
rs7819602	8	10726842	G	C	0.613	0.016	0.002	1.98e-14
rs4876400	8	119114110	G	A	0.626	-0.017	0.002	6e-16
rs7832357	8	126516197	G	A	0.342	-0.015	0.002	8.9e-13
rs9644063	8	22974450	C	T	0.822	-0.024	0.003	1.49e-18
rs45577137	8	48651633	G	A	0.045	-0.045	0.005	2.08e-17
rs16923637	8	59567885	T	G	0.286	0.014	0.002	4.79e-10
rs2977799	8	6696927	G	A	0.509	0.027	0.002	1.17e-40
rs2738104	8	6792257	A	G	0.672	0.018	0.002	4.01e-17
rs55690609	8	6902043	A	G	0.323	-0.035	0.002	8.93e-57
rs2150052	9	113945067	T	A	0.506	-0.013	0.002	8.76e-11
rs1633768	9	135879138	T	C	0.275	-0.017	0.002	7.02e-13
rs4503179	9	21950879	A	G	0.724	0.017	0.002	3.77e-13
rs12376511	9	22142756	C	T	0.163	-0.021	0.003	1.03e-13
rs7044519	9	82233842	G	T	0.161	0.017	0.003	2.37e-09
rs2548992	5	131808668	A	G	0.267	0.014	0.002	4.65e-09
rs2148204	10	102096354	A	C	0.364	-0.017	0.002	2.86e-14
rs12762973	10	960761	G	C	0.237	0.048	0.002	3.85e-83
rs33931987	16	87903382	G	A	0.208	0.018	0.003	4.73e-11
rs2384952	17	72744512	C	T	0.539	-0.015	0.002	5.14e-12
rs7249415	19	40223579	A	C	0.667	-0.015	0.002	2.02e-11
rs2967602	19	8569064	C	T	0.166	-0.028	0.003	9.21e-23
rs2926468	1	161612578	C	T	0.462	-0.018	0.002	2.54e-16
rs35076930	1	17677636	T	C	0.296	0.017	0.003	6.51e-11
rs6692170	1	205074019	A	G	0.477	-0.028	0.002	1.45e-41
rs2009069	21	39596365	T	C	0.415	0.015	0.002	1.94e-12
rs7576126	2	65658369	A	G	0.407	0.020	0.002	3.01e-21
rs10212483	3	3160214	A	G	0.043	-0.033	0.005	3.63e-10
rs13063578	3	47087837	A	T	0.401	0.017	0.002	1.32e-14
rs10023310	4	79629863	C	T	0.601	0.017	0.002	4.19e-15
rs4481234	4	9956821	C	T	0.210	-0.017	0.003	1.56e-10
rs181548	5	150847783	A	G	0.386	-0.014	0.002	3.99e-11
rs111536497	5	98187510	A	G	0.091	-0.027	0.004	6.8e-13
rs9487032	6	109605323	T	G	0.484	0.022	0.002	2.61e-26
rs111596698	6	32487123	G	T	0.262	-0.025	0.003	2.35e-22
rs72895231	6	82679396	A	C	0.596	-0.016	0.002	2.99e-13

rs2705789	7	75270073	A	T	0.459	-0.024	0.002	2.9e-28
rs140289677	7	8013293	G	A	0.058	-0.039	0.005	9.58e-18
rs74883958	10	1104030	I	D	0.644	0.014	0.002	5.1e-10

Table S7. 350 SNPs significantly associated with neutrophil count used IVs in forward MR analyses derived from Vuckovic D et al. Chr: Chromosome; EA: Effect allele; NEA: Non-effect allele; EAF: Effect allele frequency.

SNP	Chr	Pos	EA	NEA	EAF	Beta	SE	P-value
rs301819	1	8501786	G	A	0.588	-0.014	0.002	3.5e-10
rs6577536	1	8910110	G	A	0.509	0.025	0.002	1e-30
rs284317	1	10731625	G	A	0.495	0.014	0.002	6.6e-11
rs10917107	1	22329414	A	G	0.690	-0.019	0.002	1.3e-16
rs60918921	1	23850590	A	C	0.618	-0.017	0.002	1.1e-14
rs79567479	1	27011533	C	T	0.080	-0.034	0.004	2.3e-18
rs3762297	1	31231680	T	C	0.183	0.026	0.003	4.8e-21
rs3917932	1	36943916	G	C	0.577	-0.051	0.002	7e-121
rs12139676	1	42201847	A	T	0.371	-0.016	0.002	6.7e-13
rs3754224	1	43423622	C	T	0.269	-0.017	0.002	2.7e-12
rs9429088	1	46497500	A	T	0.435	-0.022	0.002	9.1e-24
rs12121236	1	56617911	A	C	0.352	0.016	0.002	1.7e-12
rs74076327	1	56979431	C	T	0.045	-0.036	0.005	3.1e-12
rs34293785	1	66137192	C	T	0.364	-0.046	0.002	5.4e-93
rs41313381	1	79411968	A	C	0.031	0.061	0.006	4.3e-24
rs115490375	1	93427987	G	A	0.025	0.042	0.007	6.4e-10
rs694180	1	111726213	G	A	0.658	0.018	0.002	1.5e-15
rs6679677	1	114303808	A	C	0.101	-0.037	0.004	1.1e-25
rs140441527	1	150026632	G	A	0.093	-0.025	0.004	2.3e-11
rs501791	1	156089873	T	C	0.044	-0.037	0.005	3.2e-12
rs34599082	1	159175494	T	C	0.014	-0.181	0.009	2.3e-86
rs150041157	1	161566312	G	C	0.526	0.017	0.002	1.1e-13
rs55970405	1	161617820	C	G	0.268	-0.022	0.002	1.5e-18
rs10918721	1	167568952	G	T	0.510	0.013	0.002	6.6e-10
rs60124939	1	174075302	T	C	0.174	-0.018	0.003	1.1e-10
rs41272536	1	183440531	G	A	0.046	-0.035	0.005	1.5e-11
rs16843346	1	198543027	T	C	0.029	-0.038	0.006	3.4e-09
rs200639793	1	200304877	A	G	0.712	0.014	0.002	2.3e-09
rs896319	1	205205651	T	G	0.912	0.027	0.004	1.4e-12
rs12141901	1	208031234	A	G	0.242	-0.023	0.002	1.5e-19
rs1855829	1	221018832	T	G	0.316	0.018	0.002	2.7e-14
rs10916617	1	224637070	T	C	0.212	0.026	0.003	2.4e-23
rs7552783	1	227174145	C	T	0.512	-0.024	0.002	1.6e-25
rs486650	1	234819885	T	C	0.471	-0.013	0.002	3.3e-10
rs2065919	1	234928045	G	C	0.562	0.021	0.002	2.6e-20
rs9970896	1	236104981	T	A	0.888	-0.058	0.003	1.6e-65
rs61838753	1	247569300	C	A	0.672	-0.019	0.002	6.3e-17
rs56188865	1	247606276	C	T	0.372	-0.027	0.002	3.7e-34
rs2113818	2	12890860	C	T	0.499	0.013	0.002	1.6e-09
rs36101491	2	24387532	T	C	0.297	0.022	0.002	2.1e-20
rs1866560	2	27328553	G	T	0.556	-0.014	0.002	3.6e-10
rs1260326	2	27730940	C	T	0.606	-0.033	0.002	2.7e-52
rs116447416	2	37501547	G	A	0.035	-0.035	0.006	1.7e-09
rs17030394	2	43354280	G	A	0.385	-0.015	0.002	1.6e-11
rs77552263	2	43786818	A	G	0.078	0.047	0.004	5.6e-32
rs75475627	2	54787592	G	C	0.076	0.034	0.004	1.2e-16
rs2421200	2	61711815	T	G	0.487	-0.020	0.002	2.7e-20
rs2028150	2	65655012	G	C	0.409	0.026	0.002	7.9e-32
rs4599108	2	85543222	T	C	0.486	-0.020	0.002	1.2e-20
rs2309996	2	101735838	G	T	0.627	0.023	0.002	3.1e-26
rs35789178	2	102603925	G	T	0.187	0.018	0.003	2.7e-11

rs10181102	2	111835489	C	T	0.274	0.016	0.002	4.3e-11
rs35898589	2	112915809	G	A	0.048	0.031	0.005	1.1e-09
rs55709272	2	113867288	C	T	0.437	0.039	0.002	2.5e-73
rs7604081	2	127866535	C	T	0.056	0.031	0.005	6e-11
rs354703	2	143883952	C	T	0.593	0.015	0.002	2.3e-11
rs3856364	2	145477217	G	C	0.681	0.016	0.002	2.6e-12
rs188653407	2	160671563	G	C	0.023	-0.056	0.007	4.5e-15
rs12692567	2	160678144	A	G	0.833	-0.037	0.003	8.4e-39
rs2632372	2	169717541	C	T	0.504	-0.021	0.002	9.1e-23
rs7573465	2	182315885	T	G	0.557	0.022	0.002	1.5e-24
rs13013264	2	192521279	T	C	0.072	0.027	0.004	1.5e-10
rs79710932	2	218947121	T	G	0.077	-0.032	0.004	1e-15
rs114050631	2	219020958	T	C	0.011	-0.159	0.011	2.2e-48
rs1521134	2	219995186	T	G	0.643	0.013	0.002	3.4e-09
rs79047930	2	220165527	T	C	0.015	0.053	0.009	2.6e-09
rs6755895	2	232579795	C	T	0.227	-0.018	0.003	8.7e-13
rs10164769	2	237779229	T	C	0.740	0.027	0.002	7.1e-29
rs56217149	3	12962914	A	G	0.151	0.018	0.003	2e-09
rs4682867	3	42905465	T	C	0.383	-0.022	0.002	1.5e-23
rs11920354	3	47262246	A	C	0.388	-0.016	0.002	4.5e-12
rs6779340	3	58033701	G	C	0.336	-0.017	0.002	3.5e-13
rs9842724	3	63804761	C	T	0.803	-0.016	0.003	3.9e-09
rs12487658	3	71528943	T	C	0.802	0.019	0.003	1.2e-12
rs4504118	3	98422588	C	G	0.556	0.013	0.002	1.3e-09
rs191832862	3	107303068	G	A	0.169	-0.025	0.003	1.1e-17
rs3732360	3	119536581	T	C	0.747	0.017	0.002	4.1e-12
rs6782812	3	128317997	A	G	0.893	-0.065	0.003	2.7e-77
rs6764912	3	128372487	A	G	0.067	-0.039	0.004	1.7e-18
rs113799399	3	140926791	T	C	0.126	0.034	0.003	8.1e-25
rs9819371	3	141206800	T	C	0.065	-0.046	0.004	1.8e-26
rs55730982	3	156797208	G	T	0.401	0.014	0.002	1.2e-10
rs10936588	3	169319801	A	G	0.634	-0.013	0.002	1.6e-09
rs4074672	3	183730295	T	C	0.369	0.018	0.002	7.5e-16
rs789858	3	194405966	T	C	0.403	0.015	0.002	3.5e-11
rs7626444	3	196504902	C	G	0.423	-0.022	0.002	1.9e-23
rs35734242	4	706700	C	T	0.428	0.024	0.002	6.6e-29
rs73191188	4	3105200	A	G	0.353	-0.016	0.002	1.5e-12
rs6831368	4	6969919	G	A	0.362	-0.023	0.002	4.6e-24
rs28530750	4	36312542	A	G	0.043	0.061	0.005	7.3e-30
rs218264	4	55408875	T	A	0.249	0.034	0.002	8.5e-43
rs723585	4	55503194	G	A	0.486	-0.022	0.002	2e-25
rs2412771	4	57761417	C	T	0.417	-0.015	0.002	3.8e-12
rs2201124	4	72597009	T	C	0.332	-0.022	0.002	1.4e-21
rs16850073	4	74703999	T	C	0.375	0.053	0.002	8.9e-124
rs146149115	4	74768492	G	A	0.010	-0.090	0.011	1.3e-15
rs16850408	4	74932807	A	C	0.369	0.069	0.002	1e-200
rs17049930	4	105579471	C	T	0.028	0.044	0.007	1.5e-11
rs7679673	4	106061534	A	C	0.376	-0.025	0.002	1.3e-28
rs4145952	4	120155806	A	C	0.401	-0.014	0.002	1.2e-10
rs11735662	4	145026126	T	C	0.034	0.049	0.006	1.2e-16
rs2290846	4	151199080	A	G	0.287	0.018	0.002	8.2e-15
rs6817881	4	152223904	T	C	0.500	0.013	0.002	1.5e-09
rs4535497	5	1107428	A	C	0.570	-0.015	0.002	4.7e-12
rs7705526	5	1285974	A	C	0.326	0.035	0.002	2.4e-50
rs11744663	5	57315635	A	G	0.192	-0.017	0.003	3.7e-10
rs11741775	5	68590395	T	C	0.443	-0.026	0.002	1.8e-33

rs71576926	5	68828803	A	T	0.784	0.016	0.003	3.3e-09
rs4703890	5	71748624	A	G	0.882	-0.044	0.003	1.2e-40
rs716848	5	96265000	A	C	0.436	0.014	0.002	2.2e-11
rs141543178	5	108133865	T	A	0.026	0.041	0.007	3.6e-09
rs257063	5	114806819	T	C	0.743	-0.015	0.002	2.5e-09
rs4836224	5	122092875	C	A	0.426	0.014	0.002	2.3e-10
rs2631367	5	131705458	G	C	0.522	0.024	0.002	4.7e-30
rs329117	5	133860101	C	T	0.582	0.014	0.002	8.9e-11
rs6580229	5	141510754	G	A	0.617	0.026	0.002	9.9e-32
rs2082382	5	148200553	A	G	0.548	-0.031	0.002	1.4e-47
rs284440	5	156170215	G	T	0.837	-0.021	0.003	4.1e-13
rs2561758	5	173205282	G	A	0.723	-0.033	0.002	8.9e-43
rs13190036	5	176737720	G	A	0.892	-0.022	0.003	1.6e-10
rs1134924	5	179126090	T	C	0.506	-0.017	0.002	4.3e-15
rs155784	5	179354966	G	A	0.353	0.014	0.002	2.8e-10
rs61025394	6	7085817	A	G	0.211	-0.022	0.003	7.9e-17
rs144721194	6	7223858	G	T	0.023	-0.043	0.007	2.6e-09
rs3777755	6	12159699	T	C	0.310	0.015	0.002	1.8e-10
rs1144700	6	16744687	T	C	0.179	-0.033	0.003	7.1e-32
rs4712614	6	21382765	G	T	0.622	-0.022	0.002	3.2e-23
rs141928748	6	28296315	G	C	0.045	-0.032	0.005	3.7e-10
rs9261145	6	29984865	T	C	0.098	0.028	0.004	3.8e-15
rs144626001	6	31374671	T	C	0.019	-0.049	0.008	7.6e-10
rs4713470	6	31472821	C	T	0.312	0.046	0.002	2e-83
rs200801362	6	31555480	C	T	0.161	-0.060	0.004	6.3e-51
rs138408209	6	32537509	T	A	0.090	-0.039	0.005	6.5e-16
rs9271578	6	32590635	T	A	0.502	0.063	0.002	5.6e-190
rs9274305	6	32632000	T	C	0.205	-0.038	0.003	6.7e-40
rs16895923	6	42633368	G	A	0.167	0.026	0.003	3.2e-19
rs729761	6	43804571	G	T	0.711	0.017	0.002	2.5e-12
rs915125	6	82463376	T	C	0.279	-0.017	0.002	6.3e-13
rs9362415	6	87968565	G	A	0.490	-0.027	0.002	6.1e-36
rs207253	6	90808423	A	G	0.408	-0.014	0.002	2.1e-09
rs364663	6	105443189	A	T	0.557	-0.013	0.002	3.3e-09
rs6927569	6	109621494	C	T	0.523	0.027	0.002	2.7e-37
rs1490384	6	126851160	T	C	0.500	-0.015	0.002	1.6e-12
rs35786788	6	135419042	A	G	0.253	-0.036	0.003	6e-47
rs62431534	6	135832523	G	C	0.544	0.020	0.002	1.1e-19
rs6924387	6	137082948	G	A	0.410	0.017	0.002	1.2e-14
rs72978754	6	138053203	C	T	0.066	-0.027	0.004	6.2e-10
rs9390461	6	147701217	G	A	0.540	0.017	0.002	5.1e-16
rs212409	6	159470058	A	G	0.553	-0.022	0.002	1.6e-23
rs4724795	7	6377836	A	G	0.291	-0.015	0.002	5.2e-10
rs6954012	7	6755724	C	A	0.112	0.026	0.003	5.2e-14
rs56195338	7	8107922	A	G	0.058	-0.039	0.005	3e-17
rs7776857	7	22754768	T	G	0.655	-0.015	0.002	1.4e-11
rs2158799	7	28277107	G	C	0.610	0.052	0.002	8.7e-125
rs56388170	7	28724374	T	G	0.292	0.071	0.002	5.3e-195
rs2710804	7	36084529	C	T	0.377	0.021	0.002	4.2e-22
rs1557934	7	45022257	C	T	0.843	0.026	0.003	8.9e-19
rs10252457	7	47337530	G	A	0.425	-0.013	0.002	3.9e-09
rs1456896	7	50304461	T	C	0.667	0.024	0.002	2.1e-26
rs149007767	7	50370254	T	C	0.162	0.031	0.003	7.4e-25
rs1540652	7	65650277	C	T	0.890	-0.027	0.004	3.6e-14
rs4718353	7	65941448	A	G	0.045	-0.034	0.005	3e-11
rs62466318	7	73042085	T	C	0.203	-0.024	0.003	5.3e-19

rs42033	7	92237533	T	A	0.208	0.041	0.003	1.3e-54
rs445	7	92408370	T	C	0.096	-0.097	0.004	2e-156
rs6465668	7	97961469	C	T	0.482	-0.015	0.002	1e-12
rs342242	7	106338989	C	T	0.454	-0.016	0.002	3.2e-14
rs11556924	7	129663496	T	C	0.389	0.016	0.002	3e-13
rs2015210	7	130737982	G	T	0.751	-0.021	0.002	1.8e-17
rs13231262	7	148886058	T	C	0.656	-0.020	0.002	2.1e-19
rs2977806	8	6692968	T	C	0.531	0.013	0.002	5.1e-10
rs55690609	8	6902043	A	G	0.322	-0.014	0.002	3.4e-10
rs2126260	8	9185081	C	T	0.798	0.017	0.003	1.1e-10
rs11781592	8	10511121	A	G	0.572	0.025	0.002	4.6e-30
rs12550612	8	22966769	A	G	0.824	-0.032	0.003	1.3e-29
rs2979489	8	30280833	A	G	0.742	0.021	0.002	9.1e-18
rs45577137	8	48651633	G	A	0.045	0.037	0.006	1.3e-11
rs28571765	8	55449281	C	T	0.209	-0.022	0.003	1.4e-16
rs7816785	8	56797418	T	C	0.606	-0.021	0.002	1.3e-20
rs609264	8	61548546	G	T	0.528	0.013	0.002	1.7e-09
rs7846314	8	61650831	T	A	0.187	0.067	0.003	6.5e-130
rs34685450	8	61916962	A	T	0.253	-0.015	0.003	4.1e-09
rs57039640	8	68864171	T	C	0.334	-0.019	0.002	2.8e-16
rs62510269	8	79012343	G	A	0.147	-0.029	0.003	6.9e-21
rs10808538	8	87169063	A	G	0.297	0.014	0.002	2.7e-09
rs2679741	8	103881011	G	C	0.366	-0.020	0.002	1.3e-19
rs4734879	8	106583124	G	A	0.275	-0.021	0.002	7.2e-18
rs2980888	8	126507308	C	T	0.699	0.023	0.002	1.7e-23
rs59697075	8	130618150	T	C	0.588	-0.038	0.002	1.6e-66
rs145209947	8	130694185	A	C	0.015	-0.069	0.010	2e-12
rs7005996	8	142241681	T	C	0.906	0.024	0.004	6.3e-11
rs34634548	8	142329460	A	C	0.358	-0.028	0.002	1.1e-35
rs2992836	9	276053	T	G	0.188	0.027	0.003	3.8e-22
rs2219143	9	2622278	A	G	0.393	0.020	0.002	3.3e-19
rs385893	9	4763176	C	T	0.524	0.027	0.002	5.3e-36
rs10758669	9	4981602	A	C	0.659	-0.015	0.002	2.9e-11
rs680775	9	79322674	G	A	0.759	-0.020	0.003	1.7e-15
rs696825	9	86583076	T	C	0.253	-0.022	0.002	4.5e-19
rs116852611	9	112745176	T	A	0.135	0.020	0.003	2e-10
rs7866863	9	114657707	A	G	0.343	0.014	0.002	3e-10
rs78610102	9	116039668	T	G	0.225	-0.020	0.003	3.6e-15
rs10986338	9	127191232	A	G	0.651	-0.015	0.002	2.6e-11
rs2519093	9	136141870	T	C	0.184	-0.041	0.003	1.4e-49
rs2810489	9	136922700	A	G	0.258	0.021	0.002	8.7e-18
rs11145986	9	139319847	G	A	0.290	0.033	0.002	1.8e-45
rs9329341	10	13532447	A	G	0.580	0.019	0.002	7.9e-18
rs56278466	10	17875857	G	T	0.662	-0.014	0.002	2e-09
rs692594	10	18265893	C	G	0.491	0.015	0.002	8.1e-12
rs10828722	10	25199951	G	A	0.370	-0.040	0.002	4.3e-73
rs2807742	10	28781367	A	G	0.770	0.034	0.003	8e-41
rs72790861	10	44880207	G	C	0.308	-0.020	0.002	6.8e-18
rs2393969	10	65140440	C	A	0.471	-0.029	0.002	1.1e-41
rs10997821	10	69570691	G	A	0.104	-0.024	0.004	4.4e-12
rs3747869	10	73520632	C	A	0.901	0.032	0.004	1.4e-19
rs2785072	10	89765388	T	A	0.168	-0.022	0.003	1.2e-12
rs1412445	10	91002804	T	C	0.338	0.021	0.002	1.1e-20
rs9633675	10	96210400	C	G	0.478	-0.019	0.002	3e-18
rs10882895	10	99075300	G	A	0.597	0.044	0.002	3.1e-90
rs4556473	10	104454700	C	T	0.384	-0.014	0.002	9.2e-10

rs76863268	10	111995452	A	T	0.344	-0.016	0.002	3.9e-12
rs151595	10	115719765	C	T	0.628	-0.014	0.002	3.9e-10
rs3781454	10	126348565	A	G	0.681	0.024	0.002	2.1e-25
rs14408	11	308314	C	T	0.368	0.050	0.002	1.9e-110
rs1468102	11	3004526	G	C	0.306	0.018	0.002	2.5e-14
rs899013	11	10476689	G	A	0.596	0.013	0.002	4.2e-09
rs1159649	11	12135604	G	C	0.316	-0.014	0.002	1.9e-09
rs10765999	11	12859917	T	C	0.677	-0.014	0.002	3.6e-09
rs55876153	11	47416636	A	G	0.323	0.020	0.002	2.2e-18
rs7396753	11	60021508	C	T	0.592	0.015	0.002	2.2e-12
rs174544	11	61567753	A	C	0.308	-0.026	0.002	9.3e-30
rs545500	11	65629934	C	G	0.667	0.017	0.002	1.9e-13
rs55904328	11	76267477	A	G	0.314	-0.014	0.002	2.4e-09
rs7129527	11	108044995	G	A	0.411	0.017	0.002	4.1e-15
rs1791807	11	113954234	T	C	0.415	-0.018	0.002	3e-16
rs238914	11	113984109	A	C	0.396	0.029	0.002	3.9e-39
rs1715429	11	118083664	G	A	0.753	0.022	0.002	4.6e-18
rs1783921	11	128094803	C	T	0.205	0.018	0.003	1.7e-11
rs8705	11	128328913	A	G	0.318	-0.020	0.002	7.9e-18
rs632887	12	3392351	G	A	0.409	0.016	0.002	1.4e-12
rs11611647	12	4333919	C	T	0.210	-0.024	0.003	8.7e-20
rs2280503	12	51138687	C	A	0.344	0.019	0.002	8e-18
rs1700159	12	52305786	T	C	0.772	0.021	0.003	5.5e-16
rs1245035	12	64976049	A	C	0.629	0.017	0.002	6.7e-14
rs7968902	12	66363070	G	T	0.570	0.014	0.002	9e-10
rs4761234	12	69732105	C	T	0.485	0.023	0.002	6.4e-26
rs7487314	12	88836215	T	G	0.701	-0.025	0.002	6.3e-27
rs17041439	12	101873240	C	A	0.056	0.027	0.005	4.3e-09
rs3184504	12	111884608	C	T	0.519	-0.031	0.002	3.1e-47
rs11064881	12	120146925	A	G	0.073	-0.035	0.004	8.3e-18
rs610578	12	121194565	G	A	0.662	-0.014	0.002	2.3e-09
rs61955089	12	123851372	C	T	0.026	0.045	0.007	1.2e-11
rs117808697	13	28513538	T	A	0.073	0.034	0.005	1.2e-13
rs2504235	13	28612886	G	A	0.638	0.026	0.002	1.6e-30
rs17521426	13	42868208	A	C	0.238	0.022	0.003	3.5e-18
rs7326825	13	50113450	A	G	0.707	0.021	0.002	2e-19
rs11841945	13	73630059	C	G	0.579	0.013	0.002	1.1e-09
rs150861794	13	109003805	T	C	0.018	-0.053	0.009	7e-10
rs2260766	13	114186800	G	A	0.281	0.024	0.002	3.1e-24
rs2038700	14	25461989	C	T	0.394	0.034	0.002	6.5e-54
rs72664840	14	35596323	T	C	0.178	0.024	0.003	2.2e-17
rs72731564	14	69280158	T	C	0.186	0.020	0.003	1.4e-12
rs12884741	14	69870944	G	A	0.065	0.044	0.004	9.6e-24
rs2205190	14	77886723	G	T	0.521	-0.016	0.002	5.4e-13
rs9806027	14	81668707	C	G	0.554	-0.018	0.002	4.3e-17
rs10498635	14	93103309	T	C	0.184	-0.029	0.003	1.9e-25
rs12588718	14	101149605	C	G	0.672	0.015	0.002	4e-11
rs2494748	14	105258892	T	C	0.616	0.013	0.002	1.8e-09
rs4983590	14	105646179	A	G	0.609	-0.017	0.002	3.7e-14
rs4924450	15	40597229	A	G	0.704	0.015	0.002	6.6e-10
rs7183879	15	42260456	G	A	0.111	-0.045	0.003	8e-39
rs2663553	15	51207818	C	G	0.540	0.020	0.002	3.4e-21
rs7180079	15	64629873	G	A	0.879	0.032	0.003	2e-22
rs7496362	15	65758874	G	C	0.366	0.017	0.002	1.7e-14
rs67538199	15	75361650	C	T	0.161	-0.022	0.003	1.1e-13
rs8024737	15	77249703	C	T	0.542	0.013	0.002	5e-10

rs72756503	15	86062567	T	C	0.147	-0.018	0.003	3.7e-09
rs7183988	15	91428589	G	T	0.526	-0.017	0.002	9.2e-16
rs67175901	15	101748227	T	C	0.108	0.045	0.003	4.1e-39
rs11647740	16	1347862	C	T	0.593	-0.018	0.002	8.3e-16
rs35929659	16	2165630	C	T	0.180	-0.019	0.003	2.3e-11
rs6500550	16	3746241	T	C	0.302	-0.024	0.002	9e-24
rs16958642	16	11864032	A	G	0.083	0.025	0.004	1.8e-10
rs61739285	16	27480797	T	C	0.034	-0.042	0.006	1.1e-12
rs12716977	16	30482803	C	T	0.517	0.020	0.002	3.7e-21
rs1362623	16	49885785	T	C	0.210	0.019	0.003	2.5e-12
rs74250734	16	50212589	G	T	0.095	-0.025	0.004	2.6e-11
rs11648664	16	53122486	A	G	0.404	-0.014	0.002	8.6e-11
rs36026517	16	74597758	C	G	0.198	0.023	0.003	2.8e-17
rs8045100	16	81603400	T	C	0.541	0.020	0.002	7.7e-21
rs305082	16	85936978	C	T	0.171	0.037	0.003	3.1e-38
rs72803323	16	89373324	T	C	0.040	0.034	0.005	3.2e-10
rs9905106	17	1373518	C	T	0.735	0.022	0.002	1e-19
rs7225843	17	2001825	C	T	0.203	-0.023	0.003	1.4e-17
rs3213878	17	5039138	T	C	0.106	0.022	0.003	4e-10
rs12936529	17	16168784	T	C	0.476	-0.037	0.002	4.3e-66
rs11653826	17	27653016	T	C	0.099	0.026	0.004	9e-13
rs2617884	17	28120430	C	G	0.497	-0.020	0.002	3.1e-20
rs11651596	17	38056116	C	T	0.468	-0.066	0.002	1e-200
rs576566496	17	38140269	A	G	0.027	0.091	0.007	6.1e-39
rs74725931	17	38196327	C	T	0.038	0.063	0.006	1e-27
rs563136944	17	38470429	G	T	0.356	-0.028	0.003	6.6e-27
rs184482331	17	40702760	T	C	0.079	0.024	0.004	3.5e-09
rs11656151	17	44068492	G	A	0.239	-0.016	0.003	1.4e-10
rs138284624	17	56385573	T	C	0.013	0.130	0.010	4.7e-42
rs529080407	17	57252243	C	A	0.043	0.045	0.007	1.8e-11
rs11650106	17	57850251	T	C	0.545	0.031	0.002	4.7e-48
rs9916458	17	72688028	A	G	0.782	0.018	0.003	2.3e-12
rs2384952	17	72744512	C	T	0.539	-0.017	0.002	1.2e-14
rs2240775	17	74710128	C	T	0.471	-0.015	0.002	2.3e-12
rs72901753	17	76244283	C	G	0.326	-0.015	0.002	1.5e-10
rs4468717	18	3457606	T	C	0.077	-0.028	0.004	3.2e-12
rs7235882	18	19684119	T	C	0.092	0.024	0.004	5.8e-11
rs303753	18	21074922	A	G	0.346	-0.023	0.002	1.1e-23
rs78285907	18	21622003	T	A	0.089	0.023	0.004	4.1e-09
rs9965539	18	41973779	A	G	0.133	0.033	0.003	1.4e-25
rs8093990	18	42122473	C	A	0.432	0.014	0.002	1.8e-10
rs55874505	18	43816804	G	T	0.584	-0.019	0.002	1.5e-16
rs8098724	18	48125899	A	G	0.212	-0.018	0.003	9.3e-12
rs66723169	18	57808978	A	C	0.230	-0.015	0.003	2.2e-09
rs1108167	18	60163025	G	A	0.286	-0.018	0.002	7.7e-14
rs72973714	18	74076020	T	C	0.067	-0.039	0.004	1.4e-19
rs930232	19	1036018	A	G	0.460	0.022	0.002	3.1e-24
rs539088769	19	3228641	G	T	0.065	-0.039	0.005	1.4e-13
rs263067	19	5028094	C	A	0.664	0.017	0.002	2.8e-13
rs571497	19	7827830	A	G	0.153	-0.039	0.003	8.5e-39
rs2116942	19	10334663	G	T	0.604	-0.022	0.002	3.4e-23
rs11085767	19	11370547	T	C	0.164	0.018	0.003	1.4e-09
rs1985157	19	18513594	C	T	0.411	0.026	0.002	1.5e-33
rs58434384	19	19786099	G	A	0.085	0.032	0.004	6.8e-17
rs4805881	19	33896432	C	A	0.665	-0.014	0.002	8.9e-10
rs4760	19	44153100	G	A	0.156	-0.075	0.003	3.2e-142

rs11673093	19	45742094	A	G	0.260	-0.047	0.002	7.3e-83
rs386243	19	49127490	T	C	0.240	-0.026	0.003	8.1e-26
rs2243602	20	1546742	A	T	0.563	-0.014	0.002	7.3e-11
rs633284	20	2904143	T	A	0.516	0.016	0.002	4.9e-13
rs579111	20	3763872	C	T	0.636	-0.013	0.002	4.2e-09
rs2294256	20	8174058	T	A	0.482	-0.016	0.002	2.5e-13
rs6029234	20	39259278	C	G	0.626	0.017	0.002	1.3e-14
rs1800961	20	43042364	T	C	0.031	-0.056	0.006	2e-19
rs143003731	20	47936696	T	C	0.008	-0.086	0.013	9.3e-12
rs6011066	20	62364180	A	G	0.674	0.015	0.002	1.5e-11
rs62239920	21	18944889	A	G	0.203	-0.017	0.003	3.1e-10
rs9977672	21	40463283	A	G	0.258	-0.024	0.002	4.2e-22
rs35990176	21	44472118	C	A	0.449	0.013	0.002	3.9e-09
rs73377344	21	47804358	C	T	0.554	-0.014	0.002	1.7e-10
rs5994158	22	17567898	G	A	0.807	-0.017	0.003	8.6e-10
rs5746451	22	18126020	C	T	0.504	0.022	0.002	2.2e-25
rs5746638	22	18907997	T	C	0.285	-0.014	0.002	2.9e-09
rs35955747	22	31813058	T	A	0.539	0.019	0.002	8.7e-18
rs139402	22	39546145	C	T	0.438	-0.014	0.002	3.3e-10

Table S8. 383 SNPs significantly associated with neutrophil count used IVs in forward MR analyses derived from Chen MH et al. Chr: Chromosome; EA: Effect allele; NEA: Non-effect allele; EAF: Effect allele frequency.

SNP	Chr	Pos	EA	NEA	EAF	Beta	SE	P-value
rs7917772	10	104487443	A	G	0.631	0.016	0.002	5.41e-16
rs10509912	10	112029405	A	T	0.135	-0.021	0.003	7.02e-14
rs72836628	10	113902454	T	C	0.248	-0.015	0.002	4.07e-12
rs180941	10	115720674	A	G	0.625	-0.013	0.002	8.33e-11
rs3781454	10	126348565	A	G	0.678	0.024	0.002	6.26e-31
rs9419387	10	133758358	A	G	0.405	-0.012	0.002	2.66e-10
rs10906393	10	13536512	T	A	0.578	0.017	0.002	9.66e-19
rs56278466	10	17875857	G	T	0.637	-0.014	0.002	1.53e-10
rs692594	10	18265893	C	G	0.490	0.012	0.002	8.23e-11
rs9804265	10	25214050	C	A	0.367	-0.040	0.002	2.79e-89
rs2807742	10	28781367	A	G	0.771	0.033	0.002	1.16e-45
rs1571956	10	30504708	A	G	0.646	0.014	0.002	1.43e-11
rs72790862	10	44880260	C	T	0.307	-0.021	0.002	6.71e-24
rs10995477	10	65010672	C	T	0.474	-0.030	0.002	2.86e-56
rs3747869	10	73520632	C	A	0.901	0.033	0.003	1.62e-22
rs2802372	10	81047575	C	A	0.435	-0.014	0.002	7.44e-12
rs1412445	10	91002804	T	C	0.338	0.021	0.002	2.64e-25
rs1977289	10	96301907	C	T	0.479	-0.018	0.002	2.14e-21
rs10786325	10	99068738	G	C	0.596	0.042	0.002	6.06e-104
rs4909932	11	10475967	G	A	0.598	0.013	0.002	8.36e-12
rs7934719	11	108341864	T	C	0.413	0.018	0.002	2.82e-20
rs61904448	11	113958121	C	T	0.284	-0.015	0.002	2.37e-11
rs73000965	11	113982321	A	T	0.316	0.029	0.002	6.63e-45
rs8705	11	128328913	A	G	0.317	-0.018	0.002	1.97e-18
rs4757955	11	12876492	A	C	0.680	-0.015	0.002	5.28e-13
rs1840595	11	18162482	T	C	0.230	0.015	0.002	1.29e-10
rs10833024	11	3010390	T	C	0.310	0.016	0.002	4.51e-15
rs7929589	11	59975078	T	C	0.407	-0.015	0.002	1.79e-14
rs7947929	11	65625191	A	G	0.666	0.016	0.002	1.38e-14
rs2282611	11	76154846	G	T	0.320	-0.013	0.002	1.03e-09
rs7131178	11	93462607	T	A	0.812	0.014	0.002	4.79e-09
rs28588142	12	10097609	T	C	0.188	0.015	0.002	6.6e-10
rs3184504	12	111884608	C	T	0.517	-0.029	0.002	1.9e-52
rs11064881	12	120146925	A	G	0.073	-0.034	0.004	2.37e-20
rs610578	12	121194565	G	A	0.663	-0.015	0.002	6.27e-13
rs1168669	12	122228694	C	T	0.814	-0.015	0.002	3.87e-09
rs632887	12	3392351	G	A	0.411	0.014	0.002	1.06e-12
rs10849020	12	4332009	G	C	0.210	-0.024	0.002	2.34e-24
rs12426444	12	50686512	A	G	0.358	0.018	0.002	6.66e-19
rs1700159	12	52305786	T	C	0.774	0.022	0.002	1.92e-20
rs1245035	12	64976049	A	C	0.628	0.016	0.002	3.39e-16
rs1042725	12	66358347	T	C	0.494	0.013	0.002	3.33e-11
rs4761234	12	69732105	C	T	0.484	0.022	0.002	4.25e-29
rs11104881	12	88843474	C	T	0.702	-0.026	0.002	7.84e-35
rs2260766	13	114186800	G	A	0.282	0.023	0.002	5.22e-27
rs76603681	13	28496468	A	G	0.032	0.041	0.006	2.65e-13
rs76428106	13	28604007	C	T	0.013	0.104	0.009	6.25e-32
rs78738581	13	42843630	A	G	0.232	0.021	0.002	1.13e-20
rs7325851	13	50113215	G	A	0.741	0.016	0.002	6.33e-13
rs2296028	13	52345637	C	G	0.175	-0.015	0.003	2.16e-09
rs9543219	13	73638478	T	C	0.574	0.013	0.002	5.06e-11

rs12588718	14	101149605	C	G	0.666	0.015	0.002	4.29e-12
rs2494748	14	105258892	T	C	0.616	0.013	0.002	1.5e-10
rs11625865	14	105644421	A	G	0.610	-0.015	0.002	1.26e-13
rs2038700	14	25461989	C	T	0.394	0.033	0.002	1.25e-64
rs72664840	14	35596323	T	C	0.179	0.021	0.003	9.21e-17
rs72731564	14	69280158	T	C	0.187	0.020	0.002	2.95e-16
rs34765661	14	69852940	C	T	0.065	0.042	0.004	8.58e-27
rs4903580	14	77850978	T	C	0.456	0.017	0.002	1.16e-17
rs10498635	14	93103309	T	C	0.184	-0.030	0.002	8.73e-34
rs67175901	15	101748227	T	C	0.107	0.044	0.003	1.99e-43
rs4924450	15	40597229	A	G	0.705	0.014	0.002	3.4e-10
rs72726027	15	42248826	C	T	0.112	-0.043	0.003	1.71e-45
rs2663553	15	51207818	C	G	0.541	0.021	0.002	4.92e-27
rs780142	15	62797964	G	T	0.274	0.013	0.002	3.84e-09
rs2062250	15	64672002	A	G	0.939	0.047	0.004	9.62e-31
rs7496362	15	65758874	G	C	0.366	0.018	0.002	4.26e-18
rs11072516	15	75224810	A	T	0.448	0.015	0.002	6.36e-15
rs4886830	15	77094418	T	A	0.490	0.012	0.002	1.61e-09
rs4843073	15	86124419	T	C	0.645	0.014	0.002	1.01e-11
rs7183988	15	91428589	G	T	0.527	-0.017	0.002	6.26e-18
rs16958642	16	11864032	A	G	0.083	0.022	0.004	2.77e-10
rs4984803	16	1349929	A	G	0.596	-0.018	0.002	1.34e-17
rs35929659	16	2165630	C	T	0.180	-0.020	0.003	1.21e-13
rs61739285	16	27480797	T	C	0.033	-0.040	0.005	1.15e-13
rs11574938	16	30485393	C	G	0.520	0.020	0.002	1.57e-25
rs6500550	16	3746241	T	C	0.304	-0.022	0.002	5.53e-26
rs1362623	16	49885785	T	C	0.208	0.018	0.002	1.49e-14
rs11648664	16	53122486	A	G	0.402	-0.014	0.002	5.43e-13
rs75974417	16	57063494	T	C	0.211	-0.017	0.002	2.32e-12
rs12927351	16	74596618	A	C	0.199	0.022	0.002	2.83e-19
rs12930850	16	81602212	G	A	0.537	0.019	0.002	1.29e-22
rs72832055	16	81869387	A	G	0.313	-0.012	0.002	3.09e-09
rs305082	16	85936978	C	T	0.172	0.037	0.003	3.04e-45
rs72803323	16	89373324	T	C	0.040	0.034	0.005	3.56e-12
rs9905106	17	1373518	C	T	0.734	0.023	0.002	1.62e-24
rs7210990	17	16170764	A	C	0.478	-0.037	0.002	9.18e-83
rs16961474	17	17146619	A	G	0.137	-0.017	0.003	1.11e-09
rs7225843	17	2001825	C	T	0.203	-0.022	0.002	2.69e-20
rs4795474	17	27228198	A	G	0.124	0.019	0.003	3.23e-11
rs7214252	17	27486673	A	G	0.211	0.015	0.002	2.11e-10
rs3115086	17	28025949	T	C	0.503	0.020	0.002	1.73e-24
rs79730542	17	37298789	C	T	0.030	-0.043	0.006	3.32e-13
rs12946510	17	37912377	T	C	0.473	-0.059	0.002	1e-200
rs74725931	17	38196327	C	T	0.037	0.067	0.005	9.03e-37
rs111975659	17	38570168	T	C	0.027	-0.041	0.006	1.96e-11
rs11656151	17	44068492	G	A	0.238	-0.016	0.002	1.08e-11
rs78813154	17	5267002	T	G	0.144	0.019	0.003	7.13e-12
rs56378716	17	56356502	G	A	0.013	0.129	0.009	3.72e-50
rs2665405	17	57875292	A	G	0.548	0.030	0.002	6.74e-53
rs749780	17	72699384	A	C	0.732	0.019	0.002	4.94e-18
rs2001613	17	75383679	T	C	0.472	0.012	0.002	2.12e-09
rs72901753	17	76244283	C	G	0.327	-0.015	0.002	1.23e-11
rs7235882	18	19684119	T	C	0.093	0.023	0.003	2.4e-12
rs303753	18	21074922	A	G	0.345	-0.023	0.002	9.57e-29
rs78285907	18	21622003	T	A	0.089	0.022	0.003	3.11e-10
rs4468717	18	3457606	T	C	0.078	-0.025	0.004	4.59e-12

rs9965539	18	41973779	A	G	0.134	0.031	0.003	1.35e-27
rs8093990	18	42122473	C	A	0.430	0.012	0.002	1.63e-10
rs17742008	18	48124763	A	C	0.212	-0.018	0.002	4.1e-14
rs656306	18	51705700	A	C	0.359	0.015	0.002	8.72e-14
rs763362	18	67531797	G	A	0.399	0.016	0.002	2.23e-15
rs72973711	18	74072245	T	A	0.067	-0.036	0.004	5.53e-21
rs10409243	19	10332988	T	C	0.601	-0.022	0.002	5.55e-26
rs930232	19	1036018	A	G	0.460	0.021	0.002	2.91e-25
rs4808683	19	17862925	G	C	0.458	-0.012	0.002	3.67e-10
rs1985157	19	18513594	C	T	0.410	0.026	0.002	1.29e-38
rs56408111	19	19793545	C	T	0.084	0.031	0.004	1.11e-18
rs4805881	19	33896432	C	A	0.665	-0.014	0.002	3.05e-12
rs4760	19	44153100	G	A	0.153	-0.075	0.003	1.42e-171
rs11673093	19	45742094	A	G	0.260	-0.049	0.002	1.45e-109
rs386243	19	49127490	T	C	0.250	-0.026	0.002	1.03e-30
rs28540102	19	4975763	C	T	0.659	0.017	0.002	8.51e-17
rs571497	19	7827830	A	G	0.155	-0.038	0.003	9.76e-45
rs284317	1	10731625	G	A	0.496	0.014	0.002	1.6e-11
rs694180	1	111726213	G	A	0.656	0.019	0.002	1.24e-20
rs2476601	1	114377568	G	A	0.900	0.038	0.003	5.82e-33
rs7555995	1	150212707	G	C	0.285	-0.015	0.002	2.86e-12
rs114427170	1	159240076	A	G	0.037	0.032	0.005	2.81e-09
rs2312675	1	166922533	C	T	0.212	-0.020	0.002	2.61e-17
rs60124939	1	174075302	T	C	0.176	-0.018	0.003	1.52e-12
rs41272536	1	183440531	G	A	0.046	-0.036	0.005	2.33e-13
rs16843350	1	198543095	T	A	0.030	-0.034	0.006	2.85e-09
rs2808519	1	200361914	T	G	0.738	0.016	0.002	1.5e-13
rs4844622	1	208034329	T	C	0.240	-0.023	0.002	1.44e-25
rs35915186	1	224654623	C	T	0.215	0.024	0.002	3.67e-25
rs11580229	1	227210820	A	G	0.462	-0.023	0.002	2.76e-30
rs533483	1	234765256	A	G	0.244	-0.017	0.002	6.13e-14
rs4626924	1	234909298	T	C	0.551	0.019	0.002	4.99e-23
rs1886654	1	236105910	C	T	0.892	-0.058	0.003	3.08e-80
rs35020138	1	247570221	C	T	0.675	-0.018	0.002	1.42e-17
rs56188865	1	247606276	C	T	0.374	-0.027	0.002	1.93e-43
rs3762297	1	31231680	T	C	0.183	0.024	0.003	2.83e-21
rs3917932	1	36943916	G	C	0.577	-0.052	0.002	3e-154
rs3754224	1	43423622	C	T	0.270	-0.017	0.002	2.7e-14
rs74076327	1	56979431	C	T	0.045	-0.039	0.005	7.85e-17
rs6678033	1	66077624	A	G	0.368	-0.043	0.002	1.46e-102
rs41313381	1	79411968	A	C	0.031	0.059	0.005	1.16e-26
rs301819	1	8501786	G	A	0.584	-0.014	0.002	6.96e-13
rs10864368	1	8918313	C	T	0.503	0.024	0.002	4.01e-35
rs150649461	1	92925654	C	G	0.015	0.053	0.008	1.9e-10
rs116631966	1	93229909	A	G	0.025	0.040	0.006	1.02e-10
rs2254458	20	1551485	T	C	0.645	-0.013	0.002	1.76e-10
rs156338	20	1854678	T	C	0.436	-0.012	0.002	3.32e-09
rs633284	20	2904143	T	A	0.516	0.015	0.002	1.74e-13
rs4812447	20	39272620	G	A	0.440	0.017	0.002	1.49e-18
rs1800961	20	43042364	T	C	0.031	-0.052	0.006	8.99e-21
rs1555275	20	55969270	G	A	0.650	-0.014	0.002	2.3e-11
rs2315008	20	62343956	G	T	0.667	0.016	0.002	1.64e-15
rs12481262	20	8126920	C	T	0.460	-0.015	0.002	4.79e-13
rs9977672	21	40463283	A	G	0.259	-0.022	0.002	7.34e-24
rs1788493	21	44469063	T	C	0.500	0.013	0.002	1.88e-11
rs2839166	21	47656712	C	T	0.398	-0.013	0.002	1.91e-11

rs41433144	22	17569993	A	T	0.043	-0.035	0.005	1.45e-12
rs5747308	22	18133500	C	A	0.504	0.022	0.002	2.3e-30
rs5746638	22	18907997	T	C	0.286	-0.014	0.002	9.41e-10
rs47341	22	43560763	T	C	0.396	0.013	0.002	2.26e-10
rs738408	22	44324730	T	C	0.217	-0.016	0.002	7.74e-12
rs35789178	2	102603925	G	T	0.186	0.018	0.002	3.41e-13
rs11691193	2	111854524	T	C	0.218	0.015	0.002	2.52e-10
rs75726191	2	112914684	T	C	0.135	0.023	0.003	1.29e-15
rs6734238	2	113841030	G	A	0.403	0.039	0.002	1.58e-90
rs11683933	2	127886955	A	C	0.055	0.031	0.004	5.72e-13
rs6432335	2	12928971	G	T	0.452	0.012	0.002	2.42e-10
rs354703	2	143883952	C	T	0.593	0.015	0.002	4.08e-14
rs3856364	2	145477217	G	C	0.680	0.014	0.002	5.28e-12
rs188653407	2	160671563	G	C	0.023	-0.051	0.006	2.58e-15
rs2729707	2	160687231	G	A	0.831	-0.036	0.003	2.68e-44
rs11689257	2	162958353	A	G	0.070	-0.025	0.004	2.51e-10
rs4632345	2	16702654	A	G	0.678	-0.012	0.002	3.24e-09
rs2632372	2	169717541	C	T	0.504	-0.021	0.002	6.68e-28
rs62189859	2	182254087	C	T	0.244	0.014	0.002	2.96e-10
rs6740847	2	182308352	G	A	0.562	0.022	0.002	3.95e-29
rs13392977	2	192514856	A	G	0.054	0.028	0.004	3.73e-11
rs1047891	2	211540507	A	C	0.315	-0.019	0.002	8.79e-21
rs114050631	2	219020958	T	C	0.011	-0.163	0.010	8.74e-59
rs11695689	2	220018091	C	A	0.640	0.012	0.002	2.88e-09
rs79047930	2	220165527	T	C	0.015	0.053	0.008	1.64e-10
rs55729107	2	232582085	G	A	0.226	-0.018	0.002	7.87e-14
rs1057258	2	234115629	T	C	0.178	0.017	0.003	2.83e-10
rs4074882	2	237780727	C	T	0.742	0.028	0.002	5.61e-37
rs36101491	2	24387532	T	C	0.294	0.021	0.002	1.41e-23
rs1260326	2	27730940	C	T	0.602	-0.033	0.002	6.91e-62
rs116447416	2	37501547	G	A	0.035	-0.037	0.005	1.47e-12
rs17030394	2	43354280	G	A	0.386	-0.016	0.002	6.29e-16
rs77552263	2	43786818	A	G	0.078	0.049	0.004	7.19e-42
rs75475627	2	54787592	G	C	0.077	0.033	0.004	3.76e-19
rs2421200	2	61711815	T	G	0.487	-0.021	0.002	1.03e-26
rs6731993	2	65642097	T	A	0.408	0.023	0.002	2.45e-31
rs7639292	3	107295665	T	C	0.169	-0.022	0.003	4.83e-18
rs58605236	3	119697032	A	T	0.373	-0.014	0.002	2.53e-12
rs2734031	3	128301390	C	T	0.908	-0.068	0.003	4.2e-91
rs6764912	3	128372487	A	G	0.065	-0.037	0.004	3.14e-20
rs56217149	3	12962914	A	G	0.151	0.017	0.003	4.45e-10
rs11712552	3	140957762	G	A	0.126	0.030	0.003	6.45e-26
rs9819371	3	141206800	T	C	0.065	-0.043	0.004	3.64e-28
rs2046934	3	151057642	A	G	0.823	0.015	0.002	1.66e-09
rs56082403	3	156797225	C	T	0.404	0.015	0.002	5.08e-15
rs11716015	3	169320058	T	C	0.636	-0.013	0.002	1.16e-10
rs3749440	3	183702089	G	A	0.372	0.017	0.002	2.2e-18
rs56174170	3	184091881	C	T	0.139	0.017	0.003	2.04e-09
rs34834004	3	185991986	G	T	0.506	-0.013	0.002	6.77e-11
rs789858	3	194405966	T	C	0.404	0.014	0.002	1.07e-12
rs7626444	3	196504902	C	G	0.422	-0.021	0.002	1.27e-26
rs1366045	3	42909050	C	T	0.385	-0.022	0.002	1.2e-28
rs6442061	3	47203783	T	G	0.596	0.017	0.002	3.68e-17
rs6779340	3	58033701	G	C	0.337	-0.015	0.002	8.69e-14
rs12487658	3	71528943	T	C	0.802	0.017	0.002	2.06e-12
rs11721064	3	98408826	T	G	0.609	0.014	0.002	3.22e-13

rs17213043	4	105546979	G	C	0.028	0.046	0.006	1.97e-15
rs7679673	4	106061534	A	C	0.380	-0.024	0.002	2.15e-33
rs4145952	4	120155806	A	C	0.399	-0.013	0.002	1.06e-11
rs11735662	4	145026126	T	C	0.034	0.048	0.005	5.59e-19
rs2290846	4	151199080	A	G	0.284	0.019	0.002	9.92e-19
rs6817881	4	152223904	T	C	0.499	0.014	0.002	1.35e-13
rs6855981	4	3148276	A	G	0.373	-0.016	0.002	1.85e-16
rs28530750	4	36312542	A	G	0.043	0.056	0.005	2.36e-31
rs218264	4	55408875	T	A	0.251	0.032	0.002	5.59e-46
rs723585	4	55503194	G	A	0.484	-0.022	0.002	2.97e-31
rs7684253	4	57727311	T	C	0.550	0.014	0.002	3.29e-13
rs6831368	4	6969919	G	A	0.362	-0.022	0.002	3.65e-27
rs35734242	4	706700	C	T	0.428	0.023	0.002	5.42e-31
rs11723621	4	72615362	G	A	0.291	-0.027	0.002	2.67e-37
rs16850073	4	74703999	T	C	0.374	0.052	0.002	2.12e-149
rs370655	4	74903134	C	T	0.394	0.062	0.002	1e-200
rs1445171	5	100046075	C	T	0.517	0.014	0.002	1.15e-13
rs4535497	5	1107428	A	C	0.570	-0.014	0.002	1.75e-12
rs257063	5	114806819	T	C	0.741	-0.015	0.002	1.54e-11
rs1966479	5	118627271	G	A	0.660	0.014	0.002	1.21e-12
rs6878780	5	122093740	C	T	0.426	0.013	0.002	6.01e-12
rs7705526	5	1285974	A	C	0.327	0.034	0.002	9.19e-54
rs2522051	5	131797578	C	T	0.454	0.024	0.002	1.88e-35
rs6860961	5	133849687	C	T	0.583	0.012	0.002	2.3e-10
rs6881942	5	141518653	T	C	0.556	0.021	0.002	2.76e-28
rs114363252	5	142539162	A	G	0.074	0.023	0.004	9.53e-10
rs284440	5	156170215	G	T	0.838	-0.020	0.003	6.16e-15
rs2561758	5	173205282	G	A	0.723	-0.034	0.002	3.49e-55
rs13190036	5	176737720	G	A	0.891	-0.022	0.003	3.63e-12
rs10447304	5	179172098	A	G	0.506	-0.017	0.002	5.58e-18
rs152232	5	179353282	C	T	0.384	0.014	0.002	4.45e-12
rs62360185	5	57274612	G	A	0.191	-0.016	0.002	8.05e-11
rs12658947	5	57552464	G	A	0.257	0.013	0.002	3.01e-09
rs6859727	5	71742622	C	T	0.878	-0.040	0.003	4.29e-43
rs2432142	5	96275201	A	G	0.435	0.015	0.002	1.89e-14
rs6927569	6	109621494	C	T	0.523	0.026	0.002	2.4e-41
rs3777755	6	12159699	T	C	0.311	0.014	0.002	4.78e-11
rs62429983	6	121862273	A	G	0.145	0.016	0.003	1.77e-09
rs1490384	6	126851160	T	C	0.501	-0.015	0.002	2.19e-14
rs9402685	6	135419688	C	T	0.256	-0.034	0.002	1.05e-52
rs12214269	6	135846518	A	G	0.545	0.019	0.002	6.6e-24
rs6924387	6	137082948	G	A	0.412	0.017	0.002	1.73e-18
rs72978754	6	138053203	C	T	0.066	-0.026	0.004	5.38e-11
rs9390461	6	147701217	G	A	0.539	0.018	0.002	3.49e-20
rs10945542	6	158752931	T	C	0.521	0.012	0.002	4.82e-10
rs212409	6	159470058	A	G	0.554	-0.021	0.002	4.71e-28
rs6915310	6	16758002	T	C	0.174	-0.032	0.003	1.71e-36
rs4712614	6	21382765	G	T	0.620	-0.020	0.002	1.04e-23
rs4712850	6	24805574	T	G	0.508	0.014	0.002	3.35e-13
rs72843538	6	25808742	A	T	0.126	0.019	0.003	1.41e-10
rs3828914	6	31465819	T	C	0.305	0.043	0.002	1.04e-94
rs12194148	6	32444198	T	G	0.469	0.059	0.002	1e-200
rs2395618	6	35299507	T	C	0.686	0.016	0.002	8.98e-15
rs16895923	6	42633368	G	A	0.166	0.025	0.003	1.62e-21
rs6905891	6	43759189	A	G	0.081	-0.028	0.004	3.26e-15
rs1089946	6	52310094	A	T	0.614	0.013	0.002	8.29e-11

rs6936191	6	7201660	C	T	0.608	0.018	0.002	9.87e-20
rs915125	6	82463376	T	C	0.282	-0.018	0.002	1.92e-16
rs6454596	6	87980735	C	G	0.490	-0.025	0.002	1.38e-39
rs7753008	6	90809639	C	T	0.387	-0.013	0.002	1.08e-10
rs342242	7	106338989	C	T	0.453	-0.016	0.002	3.62e-16
rs35759345	7	116388021	T	C	0.450	0.013	0.002	6.19e-11
rs9656395	7	130575884	G	A	0.095	-0.023	0.003	4.08e-12
rs7803075	7	130742066	G	A	0.735	-0.019	0.002	2.19e-18
rs13231262	7	148886058	T	C	0.656	-0.020	0.002	1.34e-23
rs12530608	7	18023617	G	A	0.146	-0.016	0.003	3.63e-09
rs7776857	7	22754768	T	G	0.657	-0.015	0.002	3.16e-13
rs798547	7	2761177	C	T	0.296	0.014	0.002	1.46e-10
rs2158799	7	28277107	G	C	0.611	0.052	0.002	6.53e-152
rs56388170	7	28724374	T	G	0.294	0.070	0.002	1e-200
rs2710804	7	36084529	C	T	0.376	0.020	0.002	3.76e-23
rs3735485	7	45009341	G	A	0.845	0.029	0.003	4.2e-28
rs10252457	7	47337530	G	A	0.426	-0.014	0.002	1.82e-12
rs7341532	7	48135306	T	G	0.522	0.011	0.002	3.46e-09
rs876036	7	50307710	C	T	0.321	0.021	0.002	6.88e-25
rs778732	7	65822360	T	C	0.107	0.027	0.003	6.53e-18
rs62465399	7	65941570	T	C	0.045	-0.033	0.005	1.66e-12
rs1474419	7	6692605	C	T	0.579	0.018	0.002	2.55e-19
rs33951980	7	73029437	T	C	0.129	-0.028	0.003	6.63e-22
rs73049276	7	8022016	A	G	0.059	-0.040	0.004	5.13e-22
rs42033	7	92237533	T	A	0.209	0.038	0.002	5.51e-59
rs445	7	92408370	T	C	0.098	-0.091	0.003	1.36e-175
rs7790229	7	97919338	A	G	0.493	-0.014	0.002	1.45e-12
rs4734879	8	106583124	G	A	0.276	-0.021	0.002	2.63e-23
rs1991651	8	10706411	G	C	0.618	0.023	0.002	7.4e-31
rs2954038	8	126507389	A	C	0.697	0.021	0.002	2.64e-23
rs145209947	8	130694185	A	C	0.014	-0.069	0.009	1.96e-14
rs7005996	8	142241681	T	C	0.906	0.023	0.003	3.33e-11
rs6985508	8	142337734	A	G	0.358	-0.027	0.002	3.36e-39
rs34215892	8	21767240	A	G	0.028	0.038	0.006	2.3e-10
rs12550612	8	22966769	A	G	0.822	-0.031	0.003	1.09e-33
rs6468341	8	30279355	C	T	0.743	0.019	0.002	7.1e-18
rs45577137	8	48651633	G	A	0.045	0.036	0.005	2.63e-12
rs28571765	8	55449281	C	T	0.206	-0.022	0.002	3.8e-21
rs7816785	8	56797418	T	C	0.606	-0.021	0.002	4.31e-27
rs609264	8	61548546	G	T	0.528	0.013	0.002	7.93e-11
rs7846314	8	61650831	T	A	0.187	0.064	0.002	4.7e-149
rs4276676	8	68795618	C	T	0.470	-0.018	0.002	1.16e-20
rs12716647	8	6901304	C	G	0.637	0.014	0.002	1.03e-12
rs16939607	8	79013333	A	G	0.146	-0.028	0.003	1.99e-25
rs7815046	8	87054885	C	A	0.303	-0.013	0.002	4.65e-10
rs6998846	8	9193341	A	G	0.783	0.015	0.002	4.54e-11
rs117468663	9	112745175	T	A	0.134	0.019	0.003	8.74e-12
rs1411424	9	113892963	A	G	0.523	-0.011	0.002	2.51e-09
rs7866863	9	114657707	A	G	0.341	0.013	0.002	1e-10
rs17831500	9	116047472	C	A	0.224	-0.020	0.002	5.64e-18
rs12378064	9	127191540	T	C	0.650	-0.014	0.002	1.99e-11
rs2519093	9	136141870	T	C	0.185	-0.040	0.002	9.83e-59
rs2157770	9	136921464	G	A	0.287	0.021	0.002	2.67e-21
rs4413892	9	139330158	A	G	0.278	0.034	0.002	1.65e-51
rs9411293	9	139929015	G	C	0.337	-0.014	0.002	4.19e-11
rs2219143	9	2622278	A	G	0.394	0.019	0.002	1.3e-20

rs2992836	9	276053	T	G	0.186	0.028	0.002	6.65e-29
rs3739873	9	34978431	A	G	0.220	-0.014	0.002	3.45e-09
rs3793537	9	35687556	C	G	0.291	0.013	0.002	2.55e-09
rs385893	9	4763176	C	T	0.521	0.023	0.002	7.7e-34
rs10758669	9	4981602	A	C	0.658	-0.014	0.002	1.85e-12
rs626416	9	79326680	C	G	0.762	-0.020	0.002	5.77e-18
rs796007	9	86577541	A	G	0.253	-0.021	0.002	9.41e-21
rs10992394	9	95433830	A	G	0.231	0.014	0.002	1.14e-09
rs150861794	13	109003805	C	T	0.980	0.053	0.008	2.47e-12
rs34599082	1	159175494	C	T	0.987	0.173	0.008	7.27e-94
rs138645114	10	69586392	G	T	0.056	-0.035	0.005	2.04e-14
rs74149356	10	93785201	T	A	0.169	0.016	0.003	2.06e-09
rs1715460	11	118122052	G	C	0.741	0.021	0.002	4.32e-19
rs14408	11	308314	C	T	0.368	0.049	0.002	1.21e-115
rs11823949	11	47723512	A	G	0.347	0.019	0.002	1.56e-20
rs174557	11	61581368	G	A	0.307	-0.025	0.002	3.19e-31
rs35375443	11	9729432	T	A	0.285	0.015	0.002	9.14e-12
rs370591186	12	123885930	T	C	0.026	0.048	0.006	1.89e-13
rs549435091	12	27393975	G	T	0.067	-0.028	0.004	6.2e-11
rs149859245	14	81710883	C	T	0.782	-0.019	0.002	1.41e-14
rs576566496	17	38140269	A	G	0.026	0.092	0.006	9.87e-46
rs79135050	17	38215203	C	A	0.030	0.080	0.006	2.29e-37
rs113686433	17	56803943	G	A	0.135	0.022	0.003	8.3e-13
rs55874505	18	43816804	G	T	0.584	-0.018	0.002	4.06e-19
rs12232734	18	74253960	A	T	0.186	0.016	0.003	9.43e-10
rs150665764	19	14499614	C	G	0.017	0.047	0.008	3.75e-09
rs61801809	1	161557512	T	C	0.355	-0.013	0.002	1.79e-09
rs140884047	1	161610372	C	A	0.848	0.027	0.003	1.28e-21
rs6593925	1	205100663	C	G	0.908	0.024	0.003	9.24e-13
rs12037562	1	248047350	C	T	0.660	-0.013	0.002	2.56e-10
rs11800162	1	36970512	T	C	0.020	0.064	0.007	5.07e-19
rs140958972	1	46428213	G	A	0.435	-0.020	0.002	1.45e-23
rs12136896	1	56617956	A	G	0.358	0.015	0.002	8.71e-14
rs62239920	21	18944889	A	G	0.203	-0.016	0.003	1.56e-10
rs143188161	22	24403541	T	C	0.522	0.013	0.002	3.59e-10
rs35955747	22	31813058	T	A	0.539	0.019	0.002	1.34e-21
rs2309996	2	101735838	G	T	0.625	0.023	0.002	4.27e-30
rs4954387	2	136784344	G	A	0.248	0.014	0.002	3.67e-09
rs2891550	2	27334624	T	C	0.554	-0.013	0.002	1.91e-10
rs4599108	2	85543222	T	C	0.488	-0.019	0.002	6.58e-22
rs3733120	3	63850299	C	G	0.788	-0.015	0.002	1.63e-09
rs547380175	4	26290470	T	A	0.043	-0.030	0.005	2.55e-09
rs10479004	5	131842977	T	G	0.512	0.014	0.002	7.07e-13
rs11960649	5	148203144	C	A	0.553	-0.031	0.002	1.96e-53
rs66514959	5	68611583	A	G	0.444	-0.026	0.002	2.48e-38
rs9260150	6	29911029	T	G	0.092	0.032	0.004	1.73e-19
rs9274305	6	32632000	T	C	0.192	-0.038	0.003	2.66e-43
rs62454712	7	6498057	C	T	0.239	-0.018	0.002	2.64e-14
rs10104995	8	103901234	T	C	0.231	-0.023	0.002	3.36e-22
rs59697075	8	130618150	T	C	0.587	-0.038	0.002	6.4e-80
rs6502497	17	16102530	D	I	0.464	0.033	0.002	2.67e-54
rs534234053	6	32490048	G	A	0.074	0.053	0.006	5.15e-21

Table S9. 421 SNPs significantly associated with monocyte count used IVs in forward MR analyses derived from Vuckovic D et al. Chr: Chromosome; EA: Effect allele; NEA: Non-effect allele; EAF: Effect allele frequency.

SNP	Chr	Pos	EA	NEA	EAF	Beta	SE	P-value
rs4240895	1	9713386	T	C	0.388	-0.023	0.002	2.8e-27
rs620405	1	10554794	C	T	0.684	0.015	0.002	1.6e-11
rs284324	1	10724236	A	G	0.494	0.022	0.002	8e-25
rs11247913	1	26648551	C	G	0.762	0.028	0.002	5.8e-31
rs141459612	1	28215835	T	A	0.011	0.077	0.010	4.1e-14
rs35351292	1	31208042	T	C	0.267	0.015	0.002	6.7e-11
rs77046911	1	41330726	T	C	0.112	0.029	0.003	1.5e-18
rs80200046	1	43423932	A	G	0.172	-0.027	0.003	1.2e-22
rs11211176	1	46223086	A	G	0.532	-0.016	0.002	1.8e-14
rs10788942	1	53210521	T	A	0.470	-0.017	0.002	5.2e-17
rs12117663	1	54892079	T	C	0.432	-0.017	0.002	5.5e-16
rs72675573	1	56636881	T	C	0.367	0.017	0.002	2e-14
rs1933295	1	62107021	G	A	0.776	-0.024	0.003	1.1e-21
rs72683129	1	66069781	G	A	0.148	0.017	0.003	4.7e-09
rs12562761	1	67396739	T	C	0.650	-0.018	0.002	1.2e-16
rs41313381	1	79411968	A	C	0.031	0.042	0.006	1.6e-12
rs528433	1	89987940	G	A	0.524	-0.015	0.002	2.6e-13
rs191524	1	91584916	G	A	0.658	-0.014	0.002	5.6e-11
rs11466597	1	92185351	C	T	0.012	0.291	0.010	1e-200
rs564161800	1	92423062	T	A	0.035	0.084	0.007	2.2e-33
rs115340020	1	92766438	A	G	0.028	-0.097	0.006	7.7e-54
rs12138486	1	93865391	T	C	0.781	0.018	0.003	4.4e-13
rs28549287	1	110230138	A	G	0.774	-0.016	0.003	1.2e-09
rs333947	1	110470764	A	G	0.150	0.037	0.003	1.8e-36
rs11102144	1	111195493	G	A	0.169	-0.017	0.003	5.9e-10
rs67224956	1	118154575	C	T	0.169	0.026	0.003	1.9e-21
rs11204682	1	150595537	T	G	0.223	-0.075	0.003	4.4e-195
rs3806232	1	153364130	C	T	0.118	0.029	0.003	8.3e-20
rs142676169	1	156243941	T	C	0.020	-0.048	0.008	1.3e-09
rs3026940	1	159130696	T	A	0.008	-0.099	0.013	4.2e-14
rs3027012	1	159174123	T	C	0.182	0.028	0.003	1e-24
rs10908820	1	160999479	G	A	0.796	0.017	0.003	1.5e-10
rs35902866	1	169544387	T	C	0.399	-0.018	0.002	4e-18
rs10489182	1	169710669	G	A	0.189	0.026	0.003	4e-22
rs3795503	1	180905694	T	C	0.315	-0.019	0.002	4.2e-18
rs7364556	1	185401229	C	T	0.679	0.026	0.002	1.5e-30
rs9787298	1	204276040	C	A	0.211	-0.016	0.003	4.5e-10
rs2018104	1	205309382	G	A	0.299	0.013	0.002	4.2e-09
rs9438393	1	205782718	G	A	0.414	0.019	0.002	2.4e-19
rs7522307	1	207998783	C	G	0.125	-0.022	0.003	1.7e-12
rs6702347	1	212612911	A	C	0.298	0.023	0.002	1.3e-24
rs1414519	1	221134350	A	G	0.572	0.017	0.002	1.8e-15
rs113292043	1	223231657	T	C	0.067	-0.027	0.004	1.1e-10
rs6696074	1	225962690	T	C	0.565	0.018	0.002	2.4e-16
rs4626924	1	234909298	T	C	0.551	-0.021	0.002	9.5e-23
rs4269828	1	235257838	G	A	0.559	0.013	0.002	1.1e-09
rs6429432	1	236107241	C	A	0.892	-0.092	0.003	3.8e-167
rs6429439	1	243993530	T	A	0.868	0.021	0.003	2.6e-11
rs61165644	1	244487940	G	A	0.141	-0.027	0.003	9.2e-19
rs4335411	1	249191706	A	G	0.762	-0.019	0.003	2.1e-13
rs3111414	2	8443859	G	C	0.796	0.018	0.003	4.5e-12

rs7572278	2	8563029	A	T	0.203	0.022	0.003	1.1e-17
rs2059427	2	12893977	T	C	0.372	0.021	0.002	6.4e-22
rs6753534	2	27752871	T	C	0.565	0.014	0.002	1e-10
rs649729	2	31464385	A	T	0.697	0.019	0.002	1e-16
rs28498283	2	43360065	T	A	0.256	0.022	0.002	3.5e-19
rs6706095	2	46075677	G	T	0.760	0.020	0.002	4.9e-16
rs75475627	2	54787592	G	C	0.076	0.024	0.004	2.5e-09
rs778752	2	61776902	G	C	0.613	-0.015	0.002	1.3e-12
rs13026184	2	64930786	G	C	0.243	0.017	0.002	1.4e-12
rs79709502	2	65087232	G	C	0.222	0.015	0.003	2.1e-09
rs13417564	2	65665273	T	G	0.582	0.029	0.002	6e-44
rs58448550	2	96880147	T	G	0.327	0.016	0.002	3.2e-12
rs6543084	2	102294664	C	T	0.579	0.013	0.002	2.4e-09
rs150449635	2	111752151	C	T	0.022	0.149	0.007	1.1e-90
rs72836307	2	111776154	T	C	0.157	-0.041	0.003	4.3e-47
rs72836346	2	111876613	C	G	0.078	0.034	0.004	1.1e-17
rs6735269	2	136885734	A	G	0.735	-0.041	0.002	6.8e-67
rs34005843	2	148548683	G	C	0.294	0.020	0.002	8.9e-18
rs1549387	2	160417540	G	A	0.512	0.018	0.002	8e-19
rs3931	2	169721381	G	A	0.321	0.022	0.002	4.7e-22
rs4374330	2	182058432	T	C	0.747	-0.032	0.002	1.1e-41
rs6740847	2	182308352	G	A	0.564	0.129	0.002	1e-200
rs34743120	2	182425330	A	G	0.019	-0.046	0.008	1.2e-09
rs145447411	2	182846767	C	T	0.024	0.043	0.007	6.7e-10
rs4416233	2	196992495	T	C	0.720	0.014	0.002	3.3e-09
rs10931936	2	202143928	C	T	0.732	0.014	0.002	3.2e-09
rs6736362	2	219115108	T	C	0.562	0.021	0.002	1.3e-23
rs6436124	2	220041433	A	C	0.609	0.015	0.002	7.6e-12
rs2304336	2	225750861	T	C	0.157	-0.042	0.003	4e-49
rs4470337	2	228293381	G	A	0.598	-0.013	0.002	1.9e-09
rs13002735	2	232268884	C	A	0.238	-0.015	0.002	2.5e-09
rs34236350	2	241568326	T	C	0.183	-0.033	0.003	2.9e-35
rs1822534	3	12266804	G	A	0.392	-0.032	0.002	5.5e-52
rs869785	3	24347800	C	T	0.671	-0.018	0.002	2.8e-16
rs73058593	3	39281374	C	G	0.217	0.037	0.003	2.2e-49
rs200623415	3	42831059	A	C	0.088	0.030	0.004	1.4e-15
rs2228467	3	42906116	C	T	0.061	0.163	0.004	1e-200
rs875890	3	45945287	A	T	0.260	-0.017	0.002	3.7e-13
rs2213290	3	46406367	T	C	0.409	0.037	0.002	7.2e-66
rs374231748	3	46813660	A	G	0.054	-0.033	0.005	1.3e-11
rs13094390	3	71817778	G	A	0.116	-0.021	0.003	6.9e-11
rs9809116	3	72397279	G	A	0.408	0.019	0.002	3.6e-18
rs62276455	3	98417481	A	T	0.443	0.024	0.002	2.8e-30
rs167924	3	107379837	G	A	0.622	0.014	0.002	2.4e-10
rs62263808	3	123086587	A	G	0.217	-0.023	0.003	2.3e-19
rs2291079	3	124483089	G	C	0.220	-0.015	0.003	3.8e-09
rs11713343	3	128185399	A	G	0.196	-0.025	0.003	3.8e-22
rs2245626	3	128287687	G	A	0.674	0.075	0.002	1e-200
rs2669833	3	129604169	A	G	0.157	-0.018	0.003	2.2e-10
rs62410436	3	136597549	A	C	0.681	0.016	0.002	1.1e-12
rs4683611	3	141290903	C	T	0.201	-0.017	0.003	3.4e-09
rs6440013	3	141304571	G	A	0.401	-0.024	0.002	5.4e-30
rs10804681	3	141660675	T	A	0.842	-0.020	0.003	2.2e-12
rs16831133	3	159923576	C	T	0.398	-0.014	0.002	4.3e-11
rs7633965	3	168864643	C	A	0.930	0.027	0.004	3.6e-11
rs231988	3	172276979	C	T	0.868	0.027	0.003	5.8e-18

rs9815073	3	188115682	A	C	0.343	-0.023	0.002	1.4e-23
rs7626444	3	196504902	C	G	0.423	-0.029	0.002	2e-43
rs2269486	4	2267566	T	A	0.161	0.021	0.003	1.5e-13
rs6815294	4	7042349	A	G	0.572	0.039	0.002	1e-73
rs73809166	4	36305937	C	T	0.023	0.045	0.007	2e-10
rs2711981	4	39039258	T	C	0.663	-0.023	0.002	1.4e-25
rs723585	4	55503194	G	A	0.486	-0.016	0.002	5.9e-15
rs9637714	4	57731446	C	T	0.289	-0.016	0.002	3.6e-12
rs2282679	4	72608383	G	T	0.291	-0.014	0.002	3.7e-09
rs113172748	4	79639615	G	A	0.670	0.017	0.002	9.2e-14
rs6832609	4	83556571	G	A	0.185	-0.049	0.003	2.3e-73
rs4566648	4	84159219	G	T	0.342	-0.025	0.002	8.4e-31
rs141936164	4	103401723	G	A	0.354	-0.019	0.002	2.1e-18
rs144317085	4	105806108	T	A	0.034	0.058	0.006	3.7e-23
rs6533483	4	110887989	A	G	0.403	-0.019	0.002	9.4e-20
rs9307475	4	120368556	A	G	0.327	-0.014	0.002	3.6e-10
rs4565031	4	145044759	A	G	0.550	-0.023	0.002	1.6e-27
rs72720206	4	146105777	A	G	0.325	-0.017	0.002	4.4e-14
rs7687559	4	154385925	C	G	0.399	0.015	0.002	3.6e-12
rs113862443	4	157725371	G	C	0.083	-0.025	0.004	4.5e-11
rs964751	5	17399882	T	C	0.531	-0.013	0.002	1e-09
rs116619972	5	32214314	A	G	0.027	-0.046	0.007	2.5e-12
rs4865956	5	54882505	A	T	0.696	-0.019	0.002	2e-17
rs28722705	5	55453942	T	A	0.148	0.036	0.003	1.2e-34
rs74735005	5	57202727	C	T	0.207	-0.016	0.003	4.9e-10
rs6452473	5	71741094	G	T	0.877	-0.032	0.003	3.5e-23
rs31243	5	75594360	G	A	0.095	-0.022	0.004	1.7e-09
rs165944	5	88110363	T	C	0.765	-0.016	0.002	1.4e-10
rs1902801	5	110982340	A	G	0.280	-0.017	0.002	7.4e-14
rs2631367	5	131705458	G	C	0.522	0.022	0.002	3.6e-26
rs12332674	5	132322852	G	T	0.152	-0.017	0.003	2.2e-09
rs329125	5	133871101	A	C	0.209	-0.017	0.003	1.2e-10
rs4362932	5	149483906	T	C	0.267	0.034	0.002	3.1e-47
rs17656204	5	149501803	T	C	0.323	-0.056	0.002	3.8e-135
rs360017	5	173207353	G	A	0.775	-0.023	0.003	7.4e-20
rs6883116	5	179230321	C	T	0.417	-0.017	0.002	1.3e-15
rs55693514	6	6708847	T	A	0.703	0.015	0.002	7.2e-11
rs9505057	6	7171407	G	T	0.203	0.030	0.003	4.6e-31
rs707794	6	10519890	A	C	0.530	-0.031	0.002	5.4e-48
rs707846	6	16711874	G	A	0.509	-0.017	0.002	1.4e-15
rs28505913	6	21826915	T	C	0.141	0.019	0.003	5.6e-10
rs261945	6	30272417	G	C	0.612	-0.027	0.002	3.3e-37
rs2523562	6	31330618	T	C	0.248	-0.069	0.002	3e-176
rs200801362	6	31555480	C	T	0.160	-0.074	0.004	1e-81
rs28361033	6	31973863	T	C	0.440	0.023	0.002	5.9e-26
rs41552812	6	32632689	T	C	0.070	-0.076	0.005	3.2e-46
rs56007794	6	41990827	T	A	0.243	0.042	0.002	4.8e-67
rs79894332	6	44591721	A	G	0.101	-0.049	0.003	1.3e-44
rs115202835	6	45533239	A	G	0.068	0.028	0.004	1e-11
rs17470494	6	46883834	G	A	0.102	-0.033	0.003	7.9e-22
rs632497	6	53443926	G	A	0.198	-0.020	0.003	1.7e-14
rs915125	6	82463376	T	C	0.279	0.042	0.002	3.2e-74
rs9480737	6	107442277	G	A	0.322	-0.022	0.002	1.2e-23
rs77791617	6	114083374	T	C	0.060	0.040	0.004	9e-20
rs509596	6	122799541	A	G	0.554	0.018	0.002	7.6e-18
rs34164109	6	135421176	T	C	0.261	-0.038	0.002	4.6e-58

rs9389287	6	135684975	T	C	0.278	0.017	0.002	8.2e-12
rs9494571	6	137111121	T	C	0.593	-0.016	0.002	2.3e-13
rs2797670	6	137571613	A	G	0.648	-0.013	0.002	4.2e-09
rs6920282	6	140013130	A	G	0.326	-0.014	0.002	1.1e-09
rs149110519	6	144385777	T	C	0.036	0.082	0.006	7.5e-48
rs9390460	6	147694334	C	T	0.540	0.013	0.002	1.6e-09
rs2817441	6	156938699	T	C	0.280	-0.021	0.002	6.7e-19
rs3012415	6	170489464	T	C	0.822	0.021	0.003	4e-15
rs2273215	6	170586082	A	G	0.459	-0.018	0.002	2.4e-18
rs2895	7	2568770	C	T	0.099	0.023	0.004	1.2e-10
rs798531	7	2770067	C	G	0.428	0.016	0.002	1.1e-14
rs6796	7	6502367	C	T	0.278	0.047	0.002	2.1e-90
rs75814517	7	17145526	A	G	0.056	-0.033	0.005	8.4e-13
rs17437411	7	27198086	T	C	0.069	-0.036	0.004	2.7e-18
rs17156536	7	28279488	A	C	0.158	0.025	0.003	6.4e-18
rs10238435	7	28724491	T	C	0.239	-0.024	0.003	8.8e-22
rs55959091	7	44771671	A	G	0.131	0.022	0.003	1.9e-12
rs4385425	7	50307334	G	A	0.323	-0.062	0.002	6.9e-168
rs149007767	7	50370254	T	C	0.162	-0.075	0.003	4.1e-142
rs11766800	7	50435617	T	A	0.322	0.020	0.002	1.1e-19
rs41430449	7	50798525	G	C	0.067	-0.032	0.004	3.4e-14
rs3981841	7	65184339	C	G	0.567	-0.014	0.002	1.9e-11
rs178405	7	73513045	T	G	0.786	-0.016	0.003	2.8e-10
rs445	7	92408370	T	C	0.096	-0.093	0.004	2.3e-152
rs6465668	7	97961469	C	T	0.482	-0.017	0.002	1.4e-15
rs142473518	7	99894971	G	A	0.190	0.018	0.003	6.4e-12
rs4727673	7	107021301	A	C	0.526	0.014	0.002	2.2e-11
rs71243006	7	114633041	G	T	0.104	-0.021	0.003	6.7e-10
rs1973325	7	137879428	G	A	0.692	-0.028	0.002	1.3e-34
rs28744543	7	138705803	A	G	0.509	-0.013	0.002	1.9e-09
rs71552657	7	143082534	A	G	0.211	0.021	0.003	2.5e-16
rs4840638	8	6698248	G	T	0.536	-0.024	0.002	9.6e-30
rs17078436	8	6827165	C	T	0.016	0.076	0.008	2.5e-19
rs7826487	8	6880925	G	A	0.116	-0.050	0.003	8.5e-52
rs6988319	8	6915673	G	C	0.608	0.015	0.002	1e-11
rs7460395	8	11835375	A	G	0.512	-0.016	0.002	1.1e-14
rs62501136	8	23089429	A	G	0.222	0.021	0.003	8e-17
rs3735759	8	27203039	T	C	0.867	-0.019	0.003	5.5e-10
rs62505144	8	30355038	T	G	0.230	-0.019	0.002	8.7e-15
rs4737009	8	41630405	A	G	0.236	0.017	0.002	1.6e-11
rs45577137	8	48651633	G	A	0.045	-0.068	0.005	3e-36
rs333613	8	56785685	T	C	0.611	-0.018	0.002	2e-17
rs7824937	8	61392724	G	A	0.367	0.018	0.002	1.4e-16
rs144345073	8	68811043	A	T	0.399	-0.024	0.002	1.3e-28
rs62510269	8	79012343	G	A	0.147	-0.025	0.003	2e-17
rs1863651	8	82066998	T	A	0.699	-0.017	0.002	1.1e-13
rs72673751	8	106578940	C	T	0.190	-0.017	0.003	3.4e-10
rs1954735	8	108239325	C	T	0.169	0.022	0.003	1.2e-15
rs3808461	8	116597716	T	C	0.566	-0.031	0.002	1e-47
rs12334935	8	126617990	A	G	0.472	-0.015	0.002	8.5e-13
rs7843207	8	130570063	A	C	0.506	-0.072	0.002	1e-200
rs10101101	8	130691767	A	G	0.139	-0.041	0.003	3.5e-40
rs4397371	8	130701417	T	C	0.733	-0.018	0.002	9e-14
rs837227	8	130964654	T	C	0.744	-0.017	0.002	4.2e-13
rs10099546	8	144638734	G	A	0.142	-0.021	0.003	1.8e-12
rs34881325	9	2622134	T	C	0.376	0.018	0.002	1.1e-16

rs7036656	9	21990457	T	C	0.722	0.029	0.002	4.2e-35
rs10757287	9	22143570	T	A	0.162	-0.051	0.003	2e-71
rs11557154	9	34107505	T	C	0.127	0.033	0.003	2.1e-25
rs679787	9	35040449	C	T	0.717	0.018	0.002	1.7e-14
rs10758481	9	38195756	C	T	0.504	-0.015	0.002	3.2e-13
rs10780209	9	91472127	A	G	0.477	0.045	0.002	2.5e-105
rs9410425	9	91562311	A	G	0.323	-0.029	0.002	1e-37
rs575107	9	113583908	A	G	0.070	-0.034	0.004	5.7e-17
rs1008158	9	113828811	G	A	0.342	-0.071	0.002	1e-200
rs13296236	9	113877078	G	T	0.362	-0.071	0.002	1e-200
rs13296201	9	114341971	T	C	0.192	0.050	0.003	2.6e-78
rs2181148	9	114467450	C	T	0.652	0.027	0.002	6.5e-32
rs72759267	9	126971204	T	C	0.157	0.024	0.003	5.1e-16
rs3824527	9	130702642	T	C	0.099	0.025	0.003	7.6e-13
rs687289	9	136137106	A	G	0.318	-0.047	0.002	9e-97
rs71477838	10	3827920	T	C	0.148	-0.020	0.003	1.3e-11
rs7918233	10	3919071	A	G	0.489	0.013	0.002	1.4e-10
rs7919913	10	5926216	A	G	0.346	0.020	0.002	6.9e-19
rs2646420	10	8467037	T	C	0.380	-0.026	0.002	2.8e-33
rs112955547	10	25217783	A	G	0.369	-0.046	0.002	4.2e-100
rs475616	10	30496905	G	A	0.656	0.015	0.002	5.1e-12
rs72790861	10	44880207	G	C	0.308	-0.021	0.002	1.1e-20
rs61850681	10	50248924	A	G	0.237	-0.023	0.002	5.7e-21
rs2790180	10	60036246	G	T	0.112	-0.021	0.003	2.6e-10
rs2393573	10	61657500	C	G	0.327	0.016	0.002	2.6e-12
rs224101	10	64543777	A	T	0.605	0.022	0.002	1.4e-24
rs7919533	10	73525525	C	T	0.486	0.020	0.002	2.8e-22
rs1870148	10	82271341	A	G	0.789	0.021	0.003	8.6e-17
rs11189167	10	99125722	G	A	0.255	0.054	0.002	1.4e-112
rs11190141	10	101292390	T	C	0.372	-0.044	0.002	2.7e-91
rs185575325	10	104665997	G	C	0.075	-0.026	0.004	4.9e-11
rs10885396	10	114711755	T	C	0.541	-0.016	0.002	6.9e-14
rs4980325	11	234451	T	G	0.543	0.014	0.002	6.2e-11
rs11246065	11	324170	G	A	0.339	-0.023	0.002	3.3e-26
rs907612	11	1874221	T	C	0.380	-0.035	0.002	4.3e-57
rs9783374	11	2340619	C	T	0.851	0.021	0.003	1.1e-12
rs7120300	11	8823493	T	C	0.737	-0.016	0.002	4.7e-12
rs1885523	11	33903814	T	C	0.507	0.014	0.002	1.3e-10
rs2596397	11	47247621	A	G	0.808	-0.022	0.003	7.9e-17
rs534903026	11	57309610	A	C	0.123	0.020	0.003	3.1e-09
rs6591578	11	60158649	A	G	0.630	0.023	0.002	3.2e-25
rs11227332	11	65623739	G	A	0.193	-0.016	0.003	3.1e-09
rs4441044	11	69500363	A	G	0.645	-0.019	0.002	2.3e-17
rs4944832	11	72949172	A	G	0.414	-0.016	0.002	2.7e-14
rs17756204	11	88083639	A	G	0.093	-0.029	0.004	7.3e-16
rs61890767	11	100645477	C	A	0.262	0.017	0.002	9e-13
rs17684670	11	101676821	A	G	0.065	0.035	0.004	2.6e-16
rs227043	11	108231309	A	C	0.553	0.017	0.002	4.4e-16
rs3782004	11	114070250	C	T	0.188	-0.017	0.003	5.9e-10
rs7110984	11	118442234	A	G	0.047	0.034	0.005	4.4e-12
rs3794073	11	119146129	C	T	0.273	-0.018	0.002	1.3e-14
rs12577260	11	122506129	G	C	0.124	0.065	0.003	1.1e-92
rs7108562	11	128350653	C	T	0.334	0.021	0.002	1.4e-20
rs74400777	11	128634760	T	C	0.156	-0.019	0.003	2e-09
rs2535393	12	711662	C	T	0.600	-0.017	0.002	1.4e-15
rs7955734	12	4333159	G	C	0.210	-0.024	0.003	6.7e-21

rs10849448	12	6493351	G	A	0.753	-0.054	0.002	5.5e-106
rs78326895	12	10584254	C	T	0.144	0.044	0.003	1.3e-46
rs4082413	12	30783184	G	C	0.511	0.017	0.002	2.1e-15
rs2651369	12	32552769	G	C	0.323	0.014	0.002	1.2e-09
rs1700159	12	52305786	T	C	0.772	0.019	0.002	7.6e-15
rs11170652	12	54099076	A	G	0.194	-0.019	0.003	2.3e-13
rs35979828	12	54685880	T	C	0.070	-0.044	0.004	4.3e-27
rs73120022	12	57613780	A	G	0.248	-0.014	0.002	3.2e-09
rs1800973	12	69744014	A	C	0.061	0.126	0.004	1.4e-182
rs7487314	12	88836215	T	G	0.701	-0.015	0.002	5.8e-11
rs4254123	12	89908218	A	C	0.707	0.019	0.002	4.5e-16
rs147780246	12	101884399	T	G	0.019	0.045	0.008	4.5e-09
rs12372532	12	108726376	T	C	0.079	0.023	0.004	2.8e-09
rs7314538	12	110050110	G	A	0.441	0.017	0.002	3.9e-15
rs3184504	12	111884608	C	T	0.519	-0.047	0.002	3.4e-112
rs28432783	12	124857823	C	T	0.606	0.014	0.002	8.9e-11
rs10846740	12	125303282	C	T	0.858	-0.023	0.003	4.6e-14
rs2504209	13	28546766	G	A	0.811	0.019	0.003	6.6e-13
rs188175496	13	28595940	T	C	0.024	-0.099	0.007	7.2e-41
rs76428106	13	28604007	C	T	0.013	0.555	0.010	1e-200
rs17086242	13	28611221	C	T	0.041	-0.070	0.005	2e-40
rs138028125	13	28712689	G	C	0.035	0.139	0.006	9.9e-122
rs1892548	13	41002641	C	T	0.657	-0.064	0.002	3.1e-182
rs9532580	13	41244260	C	T	0.264	-0.030	0.002	1.4e-37
rs9316470	13	50203763	A	G	0.126	-0.020	0.003	6.1e-10
rs809845	13	50724055	T	C	0.443	0.017	0.002	1.6e-16
rs73217470	13	72513420	G	A	0.047	0.064	0.005	5.1e-38
rs1146933	13	78404049	C	T	0.785	-0.015	0.003	1.4e-09
rs60699901	13	92009193	C	T	0.117	0.024	0.003	7.4e-13
rs374039502	13	108960385	A	T	0.021	-0.113	0.008	3.5e-48
rs9514999	13	109827299	T	C	0.444	0.013	0.002	7.2e-10
rs9555596	13	110011839	C	T	0.385	0.023	0.002	1.2e-25
rs7983334	13	110303919	A	G	0.872	-0.019	0.003	3.5e-09
rs58814158	13	110820255	G	T	0.156	-0.024	0.003	1.4e-16
rs2239630	14	23589349	G	A	0.558	0.046	0.002	1.4e-106
rs1052484	14	25281444	C	G	0.217	0.015	0.003	1.8e-09
rs2038700	14	25461989	C	T	0.394	0.040	0.002	1.3e-77
rs1400745	14	35355300	G	A	0.528	0.019	0.002	1.1e-18
rs113670117	14	36099366	T	C	0.088	-0.024	0.004	1.2e-10
rs12147629	14	75307588	G	A	0.311	0.019	0.002	2.6e-17
rs754388	14	93115410	C	G	0.811	0.029	0.003	9.2e-27
rs4905043	14	93550009	A	G	0.399	0.018	0.002	7.1e-17
rs62007660	14	103843344	A	G	0.737	0.064	0.002	3.9e-159
rs11856829	15	39277781	T	C	0.438	0.014	0.002	1.2e-10
rs72726037	15	42263979	A	G	0.111	-0.056	0.003	5.4e-62
rs3101851	15	50846896	T	C	0.214	-0.017	0.003	1.8e-11
rs8034610	15	64628917	A	G	0.879	0.061	0.003	2.6e-79
rs2572207	15	66070693	T	C	0.240	-0.025	0.002	4e-24
rs8037798	15	75240030	G	T	0.231	0.023	0.003	1.8e-20
rs1077965	15	80065337	G	A	0.425	0.017	0.002	1.3e-15
rs2562756	15	80226326	G	A	0.329	-0.026	0.002	2.9e-32
rs8033923	15	80257747	G	A	0.210	-0.078	0.003	1e-200
rs2061822	15	86122779	C	T	0.673	-0.016	0.002	3.8e-12
rs3803478	15	99090692	A	C	0.198	0.021	0.003	3.1e-15
rs1967309	16	4065583	G	A	0.605	-0.023	0.002	2.1e-27
rs8049116	16	4150530	T	C	0.117	0.025	0.003	3.3e-14

rs11641185	16	10986596	T	C	0.733	-0.016	0.002	4.3e-12
rs10871483	16	29592725	A	T	0.517	-0.015	0.002	3.2e-11
rs7196129	16	30471109	C	T	0.528	0.027	0.002	2.2e-36
rs35675346	16	30936081	A	G	0.240	-0.019	0.002	5.3e-15
rs11644125	16	57058974	T	C	0.600	-0.020	0.002	8.7e-21
rs34746889	16	67221444	C	G	0.018	-0.055	0.008	6e-12
rs12924604	16	74601486	A	G	0.198	-0.018	0.003	4.3e-12
rs11640701	16	84603083	A	G	0.303	0.015	0.002	1.3e-10
rs428578	16	85917944	G	A	0.348	-0.071	0.002	1e-200
rs305071	16	85949271	A	G	0.120	-0.083	0.003	1.9e-141
rs2176777	16	86012521	C	G	0.813	0.056	0.003	8.1e-98
rs4843885	16	86033186	G	T	0.183	-0.023	0.003	7.4e-17
rs16940328	16	86076108	A	G	0.113	0.039	0.003	4e-31
rs58912472	17	1626018	G	A	0.527	-0.012	0.002	3.1e-09
rs10459917	17	2009752	C	A	0.203	-0.027	0.003	1.9e-24
rs9898876	17	7526962	T	G	0.199	0.019	0.003	2.4e-13
rs118083884	17	16522922	A	G	0.016	-0.096	0.009	6.4e-26
rs4925114	17	17711270	G	A	0.654	-0.019	0.002	1.9e-18
rs62057793	17	25856325	A	G	0.111	-0.021	0.003	7.8e-10
rs880749	17	28059970	C	T	0.527	-0.034	0.002	8.4e-59
rs7223589	17	29728284	C	T	0.615	0.015	0.002	1.7e-11
rs138899494	17	32540836	A	G	0.139	0.022	0.003	1.2e-12
rs1016680	17	35874385	C	A	0.635	-0.027	0.002	2.8e-34
rs2302774	17	38183090	T	G	0.382	-0.020	0.002	2.5e-20
rs2074163	17	40270957	A	G	0.176	0.019	0.003	9.9e-12
rs62054807	17	43915497	T	C	0.293	0.022	0.002	6.1e-22
rs34097845	17	56358429	T	C	0.057	-0.114	0.005	4.3e-133
rs7212018	17	56411460	A	G	0.131	0.025	0.003	4e-16
rs2526359	17	57933508	G	A	0.436	-0.039	0.002	2.4e-75
rs236513	17	68169458	C	T	0.118	-0.021	0.003	1.1e-10
rs2084312	17	72695211	T	C	0.800	0.036	0.003	4.2e-44
rs16978176	17	72761242	C	T	0.469	-0.025	0.002	8.3e-34
rs745570	17	77781725	G	A	0.514	0.015	0.002	1.7e-12
rs9330536	17	81083854	C	T	0.487	0.064	0.002	1.9e-172
rs238135	18	3448979	C	T	0.290	-0.015	0.002	1.5e-10
rs4006418	18	19710333	T	C	0.084	0.027	0.004	7.4e-13
rs12327253	18	20719164	G	A	0.288	0.020	0.002	4.4e-18
rs718515	18	43856297	A	G	0.553	-0.022	0.002	2.2e-25
rs8084255	18	48144754	T	C	0.373	0.015	0.002	1.3e-11
rs954954	18	60902328	C	A	0.105	-0.030	0.003	2.8e-18
rs17758695	18	60920854	T	C	0.029	-0.113	0.006	6e-74
rs10460159	18	60996360	C	T	0.429	-0.016	0.002	8.1e-15
rs3177609	18	74071078	C	T	0.077	0.031	0.004	1.2e-15
rs2007483	18	77476131	A	T	0.382	-0.025	0.002	4.4e-30
rs61242663	19	836059	T	C	0.258	0.027	0.002	3.7e-28
rs113772652	19	1031550	T	C	0.459	0.021	0.002	2.2e-22
rs2873040	19	3172494	T	C	0.022	-0.050	0.007	5.4e-12
rs12980774	19	3410135	C	T	0.222	-0.017	0.003	4.9e-11
rs62107586	19	6468331	C	T	0.448	-0.014	0.002	6.3e-12
rs413141	19	6675989	G	A	0.863	0.045	0.003	3.8e-48
rs4239552	19	7848679	T	C	0.219	-0.021	0.003	5.4e-17
rs35928002	19	18116144	G	A	0.306	-0.034	0.002	3.7e-49
rs10406080	19	18402142	G	C	0.616	0.029	0.002	7.9e-42
rs79802726	19	18654587	T	G	0.187	0.020	0.003	4.1e-13
rs7249692	19	19670688	C	T	0.671	-0.018	0.002	1.3e-15
rs62126615	19	33749710	T	C	0.167	0.023	0.003	3.6e-16

rs12151289	19	33751852	C	G	0.030	-0.088	0.006	1.3e-45
rs8111664	19	44295092	A	G	0.424	-0.041	0.002	2.9e-82
rs13344267	19	45137012	C	A	0.200	0.016	0.003	1.8e-09
rs439401	19	45414451	C	T	0.638	-0.016	0.002	1.9e-13
rs344812	19	45791838	C	A	0.306	-0.051	0.002	9e-111
rs11669500	19	52144572	C	T	0.383	-0.019	0.002	1.3e-16
rs62143206	19	54326212	T	G	0.211	-0.073	0.003	2.1e-179
rs11666987	19	55698477	T	G	0.161	0.018	0.003	8.2e-10
rs34600126	20	1597433	T	G	0.219	0.023	0.003	2.1e-19
rs4813619	20	2815715	T	G	0.511	0.017	0.002	1.5e-15
rs628977	20	3649721	C	T	0.629	-0.014	0.002	4e-10
rs1883932	20	8609588	T	A	0.508	-0.029	0.002	3.7e-44
rs192009275	20	30250942	T	C	0.255	-0.021	0.002	1.9e-17
rs4911236	20	31082056	A	G	0.308	-0.032	0.002	4.4e-42
rs61114499	20	31192357	A	G	0.259	0.055	0.002	6.4e-115
rs4142441	20	42839620	G	A	0.151	0.035	0.003	1.2e-31
rs6125961	20	48884124	A	C	0.200	-0.072	0.003	2.6e-167
rs4350814	20	48969177	A	G	0.176	-0.047	0.003	9e-65
rs6091164	20	49073733	T	G	0.063	-0.036	0.004	1.8e-16
rs3787513	20	61573736	A	G	0.209	-0.017	0.003	5.2e-11
rs1997577	21	16371102	T	A	0.155	-0.033	0.003	7.9e-30
rs11088296	21	36237546	T	C	0.263	0.017	0.002	1.1e-12
rs10460712	21	36292228	C	A	0.898	-0.024	0.003	5.3e-12
rs34825969	21	39846142	A	G	0.135	-0.026	0.003	5.4e-17
rs41409548	22	17579495	A	G	0.033	-0.103	0.006	3.1e-60
rs5748937	22	17675324	T	C	0.054	-0.069	0.005	2.1e-44
rs138632530	22	17676901	T	C	0.108	0.025	0.004	1.9e-12
rs2019180	22	17712142	A	G	0.092	-0.035	0.004	2.7e-20
rs35720801	22	24639878	A	G	0.275	-0.029	0.002	1e-33
rs9625746	22	29637658	C	G	0.416	-0.021	0.002	2.5e-23
rs3091374	22	39480245	T	C	0.558	0.016	0.002	5.8e-15
rs5758364	22	41855912	C	A	0.776	0.016	0.003	1.6e-09
rs138918	22	43559451	A	G	0.392	0.031	0.002	5.8e-47
rs739239	22	50050468	A	G	0.613	0.018	0.002	1.4e-17

Table S10. 469 SNPs significantly associated with monocyte count used IVs in forward MR analyses derived from Chen MH et al. Chr: Chromosome; EA: Effect allele; NEA: Non-effect allele; EAF: Effect allele frequency.

SNP	Chr	Pos	EA	NEA	EAF	Beta	SE	P-value
rs11190141	10	101292390	T	C	0.372	-0.044	0.002	4.02e-113
rs11191514	10	104773364	T	C	0.080	-0.028	0.003	1.55e-15
rs7095778	10	111929369	G	A	0.091	-0.020	0.003	3.56e-10
rs10885396	10	114711755	T	C	0.542	-0.014	0.002	2.71e-14
rs10828725	10	25218243	T	G	0.368	-0.047	0.002	3.56e-121
rs475616	10	30496905	G	A	0.656	0.016	0.002	2.97e-15
rs1043009	10	3819714	T	C	0.377	-0.013	0.002	5.81e-12
rs7918233	10	3919071	A	G	0.489	0.012	0.002	1.54e-10
rs72790862	10	44880260	C	T	0.307	-0.020	0.002	1.92e-23
rs17011726	10	50264204	G	C	0.233	-0.023	0.002	1.23e-24
rs10795595	10	5895923	T	A	0.425	0.020	0.002	3.17e-25
rs1180658	10	61645945	A	C	0.634	-0.013	0.002	1.19e-10
rs224111	10	64552010	A	G	0.390	-0.021	0.002	1.16e-27
rs7919533	10	73525525	C	T	0.488	0.020	0.002	1.55e-26
rs77637782	10	80924258	T	G	0.433	-0.012	0.002	7.39e-10
rs2646421	10	8467155	C	G	0.378	-0.026	0.002	3.28e-41
rs2184697	10	89851815	G	T	0.293	-0.015	0.002	1.23e-12
rs11189154	10	99108922	A	G	0.256	0.051	0.002	1.25e-122
rs71482156	11	118734516	T	A	0.016	0.056	0.008	4.71e-12
rs10892342	11	119135814	C	T	0.276	-0.018	0.002	7.02e-18
rs662333	11	121578945	A	G	0.780	-0.014	0.002	1.05e-09
rs11602323	11	122519281	G	T	0.123	0.065	0.003	9.22e-115
rs695113	11	128562098	T	C	0.697	0.021	0.002	1.04e-24
rs907612	11	1874221	T	C	0.380	-0.033	0.002	5.19e-60
rs4980325	11	234451	T	G	0.541	0.014	0.002	5.73e-13
rs11246065	11	324170	G	A	0.339	-0.022	0.002	1.6e-26
rs1885525	11	33904180	A	G	0.506	0.013	0.002	7.62e-12
rs2957873	11	47249294	A	G	0.809	-0.021	0.002	9.44e-19
rs573790	11	59855385	C	T	0.614	-0.016	0.002	1.48e-16
rs6591578	11	60158649	A	G	0.634	0.022	0.002	2.03e-29
rs10796828	11	69490346	G	T	0.635	-0.018	0.002	3.26e-20
rs11235689	11	72949747	T	C	0.414	-0.016	0.002	4.79e-16
rs72966841	11	88070110	T	A	0.094	-0.027	0.003	1.61e-16
rs7120300	11	8823493	T	C	0.735	-0.017	0.002	6.41e-16
rs7975680	12	101864287	C	A	0.604	0.013	0.002	2.12e-10
rs2734442	12	10600368	A	G	0.876	-0.042	0.003	3e-49
rs10744732	12	1078076	T	C	0.378	0.012	0.002	2.49e-09
rs4365101	12	108707583	A	G	0.068	-0.027	0.004	1.14e-12
rs9943753	12	109840940	G	A	0.628	0.014	0.002	4.79e-12
rs3184504	12	111884608	C	T	0.517	-0.047	0.002	1.58e-139
rs73201961	12	116830960	C	A	0.089	0.027	0.003	1.44e-16
rs7308348	12	122225420	C	T	0.182	0.015	0.002	4.78e-10
rs11057841	12	125316743	T	C	0.144	0.020	0.003	8.16e-14
rs4082413	12	30783184	G	C	0.512	0.015	0.002	2.06e-16
rs2651369	12	32552769	G	C	0.326	0.014	0.002	1.45e-12
rs10849020	12	4332009	G	C	0.210	-0.024	0.002	1.28e-25
rs1700159	12	52305786	T	C	0.774	0.020	0.002	1.83e-19
rs12309572	12	54074686	T	C	0.168	0.019	0.003	2.44e-14
rs35979828	12	54685880	T	C	0.071	-0.046	0.004	1.28e-35
rs7485577	12	57616061	A	G	0.269	-0.015	0.002	9.09e-12
rs10849448	12	6493351	G	A	0.753	-0.050	0.002	2.77e-111

rs1800973	12	69744014	A	C	0.062	0.113	0.004	1.64e-187
rs11104881	12	88843474	C	T	0.702	-0.016	0.002	3.35e-14
rs12306790	12	89864047	T	C	0.715	0.019	0.002	2.22e-20
rs9555596	13	110011839	C	T	0.383	0.023	0.002	1.55e-32
rs7983334	13	110303919	A	G	0.872	-0.019	0.003	3.38e-11
rs58814158	13	110820255	G	T	0.157	-0.024	0.003	3.24e-19
rs9583493	13	111091632	A	C	0.427	0.011	0.002	3.12e-09
rs2183246	13	114901188	T	C	0.683	-0.014	0.002	1.94e-11
rs2504209	13	28546766	G	A	0.811	0.018	0.002	3.58e-14
rs188175496	13	28595940	T	C	0.024	-0.093	0.007	2.93e-43
rs76428106	13	28604007	C	T	0.013	0.536	0.009	1e-200
rs17086242	13	28611221	C	T	0.041	-0.069	0.005	1.71e-47
rs138028125	13	28712689	G	C	0.035	0.135	0.006	6.19e-131
rs1892548	13	41002641	C	T	0.655	-0.063	0.002	1e-200
rs9532580	13	41244260	C	T	0.264	-0.028	0.002	9.72e-40
rs9316470	13	50203763	A	G	0.127	-0.017	0.003	1.72e-09
rs809845	13	50724055	T	C	0.443	0.015	0.002	3.57e-16
rs73217470	13	72513420	G	A	0.047	0.065	0.004	2.11e-47
rs1146933	13	78404049	C	T	0.786	-0.014	0.002	8.88e-10
rs60699901	13	92009193	C	T	0.118	0.022	0.003	2.02e-13
rs8016326	14	103846716	A	G	0.734	0.061	0.002	3.81e-178
rs4983387	14	105268228	A	G	0.899	0.019	0.003	2.2e-09
rs2239630	14	23589349	G	A	0.556	0.049	0.002	4.68e-146
rs1052484	14	25281444	C	G	0.216	0.014	0.002	1.28e-09
rs2038700	14	25461989	C	T	0.394	0.040	0.002	2.81e-96
rs754388	14	93115410	C	G	0.811	0.030	0.002	6.43e-36
rs4905043	14	93550009	A	G	0.400	0.019	0.002	1.31e-20
rs62018159	15	40397421	A	G	0.209	0.015	0.002	1.42e-10
rs1002774	15	42261781	A	G	0.112	-0.054	0.003	4.48e-73
rs60695341	15	51010271	T	C	0.197	-0.018	0.002	1.14e-14
rs6493575	15	53013475	T	C	0.396	-0.012	0.002	1.86e-10
rs62011334	15	63837491	G	A	0.355	0.012	0.002	2.1e-09
rs7180079	15	64629873	G	A	0.877	0.059	0.003	3.41e-95
rs11857609	15	66095270	C	T	0.760	0.023	0.002	3.99e-26
rs1081230	15	80095646	G	A	0.577	-0.020	0.002	8.43e-25
rs2061822	15	86122779	C	T	0.674	-0.014	0.002	8.67e-12
rs73467599	15	99088453	C	T	0.199	0.018	0.002	5.95e-15
rs11641185	16	10986596	T	C	0.732	-0.015	0.002	5.06e-13
rs7196129	16	30471109	C	T	0.530	0.025	0.002	2.45e-39
rs7185007	16	30927509	T	C	0.240	-0.017	0.002	2.4e-14
rs1967309	16	4065583	G	A	0.603	-0.022	0.002	1.43e-30
rs11508026	16	56999328	T	C	0.431	-0.012	0.002	2.7e-10
rs11644125	16	57058974	T	C	0.598	-0.019	0.002	4.73e-24
rs9937847	16	85917551	C	T	0.071	-0.112	0.004	1e-200
rs366078	16	85964563	C	T	0.158	0.107	0.003	1e-200
rs76209847	16	85967249	T	C	0.037	-0.075	0.005	7.35e-49
rs2885363	16	86009261	C	G	0.079	0.117	0.004	1e-200
rs140634734	16	86018831	T	C	0.028	0.047	0.006	4.27e-15
rs79252394	16	86021968	T	G	0.030	-0.043	0.006	2.91e-14
rs12942576	17	1312461	C	T	0.683	0.012	0.002	2.19e-09
rs118083884	17	16522922	A	G	0.017	-0.097	0.009	1.01e-29
rs12941356	17	17716531	G	A	0.588	-0.017	0.002	2.45e-18
rs9915112	17	2007826	G	A	0.203	-0.027	0.002	4.21e-30
rs62057793	17	25856325	A	G	0.112	-0.019	0.003	8e-11
rs71368117	17	27329227	C	T	0.087	-0.024	0.003	1.58e-11
rs2729450	17	28088459	C	T	0.524	-0.031	0.002	1.36e-59

rs17614093	17	32509256	G	C	0.430	-0.015	0.002	7.17e-15
rs1016680	17	35874385	C	A	0.637	-0.026	0.002	4.31e-39
rs2302774	17	38183090	T	G	0.383	-0.024	0.002	5.68e-36
rs2074163	17	40270957	A	G	0.177	0.019	0.002	4.21e-14
rs62054807	17	43915497	T	C	0.295	0.020	0.002	2.45e-22
rs34097845	17	56358429	T	C	0.056	-0.101	0.004	2.99e-124
rs11656162	17	56404625	C	G	0.034	0.035	0.005	7.65e-11
rs2665405	17	57875292	A	G	0.548	0.038	0.002	5.37e-93
rs236513	17	68169458	C	T	0.118	-0.020	0.003	6.64e-12
rs2084312	17	72695211	T	C	0.800	0.038	0.002	8.97e-58
rs16978176	17	72761242	C	T	0.465	-0.025	0.002	2.89e-40
rs9898876	17	7526962	T	G	0.197	0.018	0.002	9.89e-14
rs745570	17	77781725	G	A	0.514	0.014	0.002	5.38e-13
rs9674881	17	81078568	T	C	0.486	0.063	0.002	2.16e-189
rs238136	18	3448755	G	A	0.250	-0.015	0.002	1.68e-11
rs11082397	18	42094299	G	T	0.129	0.019	0.003	1.36e-11
rs718515	18	43856297	A	G	0.552	-0.020	0.002	5.66e-26
rs9963693	18	45599257	C	T	0.261	0.013	0.002	5.39e-10
rs745822	18	48142822	G	T	0.376	0.016	0.002	3.16e-17
rs954954	18	60902328	C	A	0.105	-0.029	0.003	4.2e-21
rs141390096	18	60911544	C	G	0.037	-0.033	0.005	4.2e-11
rs17758695	18	60920854	T	C	0.029	-0.109	0.006	3.89e-80
rs10460159	18	60996360	C	T	0.429	-0.016	0.002	9.88e-17
rs3177609	18	74071078	C	T	0.078	0.031	0.004	8.26e-19
rs2007483	18	77476131	A	T	0.382	-0.024	0.002	1.63e-34
rs10403909	19	1008358	G	A	0.724	0.019	0.002	1.95e-17
rs11086102	19	18398628	C	G	0.633	0.031	0.002	2.26e-54
rs3859570	19	18510925	C	T	0.425	-0.028	0.002	3.97e-44
rs7249692	19	19670688	C	T	0.671	-0.017	0.002	8.83e-18
rs72987040	19	3251116	A	C	0.124	-0.025	0.003	2.71e-18
rs62126615	19	33749710	T	C	0.168	0.020	0.003	3.69e-16
rs74259566	19	42735177	G	C	0.101	0.029	0.003	7.51e-20
rs56344893	19	44282529	A	C	0.373	-0.044	0.002	2.12e-114
rs10425556	19	45259871	T	G	0.062	0.026	0.004	1.94e-10
rs11669910	19	45741333	T	A	0.258	-0.067	0.002	1e-200
rs33978622	19	51736383	C	G	0.329	-0.018	0.002	7.51e-19
rs3803904	19	55699646	T	C	0.134	0.017	0.003	1.96e-09
rs413141	19	6675989	G	A	0.863	0.043	0.003	1.42e-54
rs571497	19	7827830	A	G	0.155	-0.028	0.003	8.94e-27
rs6687430	1	10633245	A	G	0.543	0.012	0.002	2.41e-10
rs284324	1	10724236	A	G	0.495	0.021	0.002	5.94e-27
rs333947	1	110470764	A	G	0.150	0.037	0.003	2.21e-44
rs11102144	1	111195493	G	A	0.166	-0.016	0.003	1.43e-10
rs67224956	1	118154575	C	T	0.170	0.027	0.002	2.01e-27
rs6665912	1	150544093	C	T	0.223	-0.072	0.002	1e-200
rs60752752	1	153339782	G	A	0.118	0.025	0.003	2.95e-17
rs3027012	1	159174123	T	C	0.183	0.028	0.002	4.37e-31
rs4987353	1	169666987	A	G	0.310	-0.022	0.002	1.93e-27
rs3795503	1	180905694	T	C	0.315	-0.019	0.002	1.68e-20
rs11590380	1	185400939	C	T	0.679	0.025	0.002	1.69e-32
rs12747432	1	198566554	T	G	0.067	-0.024	0.004	1.31e-10
rs9787298	1	204276040	C	A	0.211	-0.016	0.002	2.66e-12
rs1772143	1	205799987	A	T	0.413	0.020	0.002	8.11e-26
rs7522307	1	207998783	C	G	0.124	-0.023	0.003	4.36e-16
rs701905	1	212553111	C	G	0.352	0.022	0.002	1.63e-28
rs2784250	1	221060393	G	T	0.140	-0.017	0.003	3.11e-10

rs1335929	1	221136787	C	T	0.571	0.017	0.002	1.77e-18
rs113292043	1	223231657	T	C	0.066	-0.025	0.004	5.45e-11
rs6696074	1	225962690	T	C	0.563	0.018	0.002	1.49e-21
rs4626924	1	234909298	T	C	0.551	-0.018	0.002	8.2e-22
rs4269828	1	235257838	G	A	0.560	0.012	0.002	1.89e-10
rs6429432	1	236107241	C	A	0.892	-0.080	0.003	6.29e-156
rs1212036	1	23690216	T	C	0.114	-0.018	0.003	1.72e-09
rs61165644	1	244487940	G	A	0.141	-0.026	0.003	2.59e-22
rs4335411	1	249191706	A	G	0.762	-0.017	0.002	5.51e-13
rs11247908	1	26638222	A	G	0.173	-0.029	0.002	1.92e-32
rs188393352	1	28254136	G	C	0.010	0.087	0.010	2.36e-17
rs35351292	1	31208042	T	C	0.265	0.014	0.002	6.97e-11
rs41268099	1	41328644	A	G	0.111	0.029	0.003	2.47e-22
rs2786487	1	42368339	C	G	0.559	0.015	0.002	3.33e-15
rs17387886	1	43419705	C	G	0.174	-0.028	0.003	1.45e-28
rs55684236	1	44080009	A	G	0.348	0.012	0.002	3.89e-09
rs2274664	1	54704629	T	C	0.580	-0.015	0.002	2.75e-15
rs12738019	1	54876199	G	A	0.556	-0.013	0.002	2.21e-12
rs72675573	1	56636881	T	C	0.366	0.016	0.002	1.76e-16
rs1933295	1	62107021	G	A	0.777	-0.024	0.002	1.74e-25
rs6664626	1	66128239	T	G	0.143	0.019	0.003	4.38e-11
rs41313381	1	79411968	A	C	0.030	0.043	0.005	8.85e-16
rs7532966	1	89955188	C	T	0.499	0.017	0.002	1.6e-19
rs12144117	1	9182141	T	C	0.215	-0.014	0.002	7.63e-10
rs114425738	1	92171673	C	G	0.013	0.268	0.008	1e-200
rs115340020	1	92766438	A	G	0.027	-0.094	0.006	2.22e-59
rs7524046	1	9307033	A	G	0.251	0.015	0.002	1.11e-11
rs2433279	1	93907909	G	T	0.879	0.023	0.003	2.08e-15
rs80036648	1	93912771	G	A	0.017	0.155	0.008	1.16e-92
rs7516138	1	9711642	G	A	0.391	-0.023	0.002	1.31e-31
rs34600126	20	1597433	T	G	0.218	0.022	0.002	6.03e-23
rs4813619	20	2815715	T	G	0.511	0.017	0.002	4.24e-18
rs12480462	20	31065178	T	C	0.316	-0.031	0.002	1.44e-52
rs12480732	20	31191015	T	C	0.258	0.054	0.002	8.58e-136
rs628977	20	3649721	C	T	0.631	-0.013	0.002	1.21e-10
rs4142441	20	42839620	G	A	0.152	0.031	0.003	1.22e-31
rs34414028	20	47212868	T	A	0.209	0.014	0.002	2.47e-09
rs932905	20	48796131	A	G	0.282	0.021	0.002	6.11e-24
rs17196752	20	48887268	T	C	0.190	-0.072	0.002	8.1e-199
rs6512627	20	48967591	A	G	0.194	-0.047	0.002	1.83e-88
rs3787513	20	61573736	A	G	0.207	-0.015	0.002	6.32e-11
rs4432538	20	8607393	A	G	0.509	-0.029	0.002	4.75e-54
rs1997577	21	16371102	T	A	0.155	-0.030	0.003	5.8e-30
rs57221391	21	16583936	G	T	0.071	-0.022	0.004	1.51e-09
rs35068491	21	36238307	C	T	0.209	0.015	0.002	5.68e-10
rs2834670	21	36280376	G	A	0.176	0.027	0.003	1.11e-19
rs73203055	21	36407530	G	C	0.072	-0.026	0.004	1.27e-12
rs192498589	22	17516047	G	A	0.017	0.070	0.008	8.24e-18
rs41409548	22	17579495	A	G	0.034	-0.096	0.006	1.49e-64
rs9606614	22	17579956	T	C	0.033	0.034	0.005	1.19e-10
rs2159068	22	17653955	G	C	0.798	0.017	0.002	3.28e-13
rs2019180	22	17712142	A	G	0.093	-0.037	0.003	2.57e-27
rs35477714	22	18202530	C	G	0.505	0.013	0.002	1.39e-11
rs34505104	22	24624609	G	A	0.305	-0.033	0.002	6.38e-57
rs9625746	22	29637658	C	G	0.413	-0.021	0.002	7.42e-28
rs62236123	22	31474019	C	G	0.042	-0.028	0.005	4.75e-09

rs139271	22	39487595	C	T	0.442	-0.015	0.002	1.11e-15
rs47341	22	43560763	T	C	0.396	0.031	0.002	1.46e-54
rs739241	22	50052921	A	G	0.607	0.018	0.002	1.72e-20
rs140763648	22	50767351	G	A	0.412	-0.012	0.002	4.34e-09
rs6745920	2	102145661	C	T	0.619	0.013	0.002	2.02e-10
rs150449635	2	111752151	C	T	0.022	0.140	0.007	4.24e-97
rs72836307	2	111776154	T	C	0.156	-0.041	0.003	1.37e-56
rs3789062	2	111917317	T	C	0.245	-0.020	0.002	3.22e-20
rs4669869	2	12898460	C	T	0.445	0.020	0.002	3.76e-27
rs7574456	2	136890059	T	C	0.740	-0.040	0.002	5.2e-80
rs7608128	2	137007943	T	G	0.181	-0.019	0.002	1.39e-15
rs3856364	2	145477217	G	C	0.680	0.013	0.002	3.45e-10
rs13032786	2	148803672	G	C	0.300	0.020	0.002	2.49e-23
rs1863219	2	160418171	G	C	0.512	0.018	0.002	5.95e-23
rs13032491	2	161080233	T	C	0.139	0.016	0.003	4.72e-09
rs6733162	2	162913498	C	G	0.624	0.012	0.002	1.4e-09
rs3931	2	169721381	G	A	0.320	0.023	0.002	2.02e-30
rs8207	2	170493863	G	A	0.267	0.014	0.002	1.29e-10
rs11680095	2	181825956	T	C	0.592	0.022	0.002	1.35e-31
rs78655702	2	182303729	T	C	0.050	-0.059	0.004	3.58e-43
rs155127	2	182305701	C	G	0.348	0.060	0.002	1e-200
rs155109	2	182352679	G	A	0.321	-0.021	0.002	7.19e-26
rs17860428	2	202151400	A	G	0.180	-0.016	0.002	1.39e-10
rs6736362	2	219115108	T	C	0.559	0.022	0.002	6.45e-32
rs6436124	2	220041433	A	C	0.608	0.015	0.002	8.1e-15
rs10197805	2	225750753	C	T	0.157	-0.042	0.003	4.7e-59
rs4470337	2	228293381	G	A	0.596	-0.012	0.002	4.33e-10
rs13002735	2	232268884	C	A	0.241	-0.014	0.002	3.3e-10
rs30102	2	237817538	C	G	0.317	-0.013	0.002	9.89e-11
rs34236350	2	241568326	T	C	0.183	-0.032	0.003	8.44e-38
rs2950835	2	27750545	G	A	0.557	0.013	0.002	7.45e-12
rs649729	2	31464385	A	T	0.698	0.021	0.002	5.04e-24
rs28498283	2	43360065	T	A	0.258	0.021	0.002	2.41e-22
rs6706095	2	46075677	G	T	0.760	0.019	0.002	9.2e-19
rs75475627	2	54787592	G	C	0.077	0.024	0.004	1.8e-11
rs1562309	2	61770126	A	C	0.615	-0.015	0.002	1.04e-15
rs1968179	2	64929212	T	C	0.243	0.018	0.002	3.81e-17
rs13385171	2	65661843	C	T	0.583	0.028	0.002	5.14e-49
rs3111414	2	8443859	G	C	0.794	0.017	0.002	5.62e-14
rs7572278	2	8563029	A	T	0.207	0.022	0.002	3.44e-20
rs4907230	2	96855241	A	G	0.324	0.016	0.002	7.24e-15
rs62261974	3	107296969	G	A	0.183	-0.017	0.002	7.01e-12
rs1822534	3	12266804	G	A	0.394	-0.031	0.002	4.69e-59
rs62263808	3	123086587	A	G	0.217	-0.022	0.002	2.37e-22
rs2291079	3	124483089	G	C	0.219	-0.015	0.002	1.97e-11
rs11713343	3	128185399	A	G	0.197	-0.026	0.002	5.24e-28
rs2245626	3	128287687	G	A	0.676	0.075	0.002	1e-200
rs2669833	3	129604169	A	G	0.160	-0.019	0.003	2.54e-13
rs7434143	3	136600667	T	G	0.680	0.017	0.002	6.49e-17
rs6800122	3	141249398	T	C	0.400	-0.022	0.002	4.75e-30
rs10804681	3	141660675	T	A	0.843	-0.018	0.003	9.56e-13
rs1472121	3	151036229	T	C	0.776	0.014	0.002	3.65e-10
rs16831132	3	159923303	T	C	0.397	-0.014	0.002	2.07e-12
rs7633965	3	168864643	C	A	0.929	0.025	0.004	8.35e-12
rs231988	3	172276979	C	T	0.868	0.026	0.003	4.8e-19
rs2018092	3	185903842	C	T	0.615	-0.012	0.002	1.97e-09

rs56925909	3	187429202	G	C	0.219	0.014	0.002	1.33e-09
rs9815073	3	188115682	A	C	0.346	-0.022	0.002	4.16e-26
rs7626444	3	196504902	C	G	0.421	-0.029	0.002	5.72e-52
rs869785	3	24347800	C	T	0.670	-0.017	0.002	3.97e-18
rs3732378	3	39307162	A	G	0.173	0.055	0.002	1.07e-108
rs4683345	3	42886642	A	G	0.385	-0.058	0.002	1e-200
rs73083231	3	43172180	G	A	0.045	0.029	0.005	1.04e-10
rs28677778	3	46322171	G	A	0.067	-0.065	0.004	5.53e-68
rs112313229	3	46364860	A	G	0.045	0.057	0.005	6.64e-36
rs7429588	3	46835250	C	T	0.293	0.015	0.002	7.84e-11
rs11130612	3	58029016	T	C	0.334	-0.012	0.002	4.71e-10
rs13079034	3	71813219	T	C	0.113	-0.021	0.003	1.05e-12
rs9809116	3	72397279	G	A	0.409	0.017	0.002	8.21e-20
rs10935473	3	98416900	T	G	0.442	0.025	0.002	2.3e-40
rs141936164	4	103401723	G	A	0.351	-0.019	0.002	2.29e-21
rs144317085	4	105806108	T	A	0.034	0.062	0.005	3.1e-32
rs6533483	4	110887989	A	G	0.403	-0.018	0.002	8.82e-22
rs7692994	4	120427489	A	C	0.330	-0.014	0.002	5.59e-12
rs4240356	4	145042250	G	C	0.550	-0.024	0.002	1.94e-35
rs7687559	4	154385925	C	G	0.398	0.014	0.002	1.58e-12
rs113862443	4	157725371	G	C	0.085	-0.025	0.003	7.94e-14
rs56058420	4	185237116	G	A	0.356	0.012	0.002	2.96e-09
rs2269486	4	2267566	T	A	0.164	0.021	0.003	8.55e-16
rs73809166	4	36305937	C	T	0.023	0.046	0.006	3.78e-13
rs2711981	4	39039258	T	C	0.663	-0.022	0.002	1.59e-27
rs723585	4	55503194	G	A	0.483	-0.017	0.002	9.43e-19
rs9637714	4	57731446	C	T	0.289	-0.016	0.002	5.42e-15
rs871134	4	7044380	T	C	0.571	0.038	0.002	7.06e-89
rs11723621	4	72615362	G	A	0.291	-0.013	0.002	1.48e-10
rs113172748	4	79639615	G	A	0.671	0.016	0.002	4.59e-15
rs17005891	4	83547862	A	G	0.184	-0.051	0.002	1.15e-98
rs4566648	4	84159219	G	T	0.342	-0.025	0.002	8.14e-38
rs2548257	5	100162317	A	C	0.653	0.013	0.002	5.35e-11
rs10478058	5	110962584	G	A	0.197	0.015	0.002	3.01e-10
rs6869021	5	111039017	T	C	0.313	-0.017	0.002	5.47e-17
rs11242109	5	131677047	T	G	0.478	-0.022	0.002	4.75e-33
rs12332674	5	132322852	G	T	0.152	-0.017	0.003	3.33e-10
rs329125	5	133871101	A	C	0.207	-0.017	0.002	1.38e-12
rs6579771	5	149482262	T	C	0.269	0.034	0.002	6.13e-57
rs17656204	5	149501803	T	C	0.322	-0.054	0.002	6.78e-156
rs360017	5	173207353	G	A	0.775	-0.024	0.002	1.62e-27
rs964752	5	17399899	G	C	0.540	-0.012	0.002	9.61e-11
rs6883116	5	179230321	C	T	0.417	-0.018	0.002	8.21e-21
rs116619972	5	32214314	A	G	0.026	-0.040	0.006	6.73e-12
rs445611	5	35396119	G	A	0.845	0.016	0.003	8.58e-10
rs4865956	5	54882505	A	T	0.696	-0.019	0.002	1.97e-20
rs28722705	5	55453942	T	A	0.149	0.034	0.003	1e-38
rs74735005	5	57202727	C	T	0.206	-0.017	0.002	3.14e-13
rs2338021	5	71749094	C	T	0.880	-0.032	0.003	3.64e-28
rs31243	5	75594360	G	A	0.096	-0.023	0.003	1.35e-12
rs244760	5	88111209	G	C	0.764	-0.014	0.002	1.14e-10
rs707794	6	10519890	A	C	0.532	-0.030	0.002	7.79e-59
rs9480737	6	107442277	G	A	0.321	-0.022	0.002	2.74e-27
rs1970364	6	113969527	T	C	0.265	0.019	0.002	1.32e-19
rs9375150	6	122887555	G	T	0.447	-0.018	0.002	1.84e-22
rs761841	6	131114386	C	T	0.577	0.012	0.002	1.31e-10

rs12055642	6	135079040	C	T	0.199	0.018	0.002	1.04e-14
rs7776054	6	135418916	G	A	0.261	-0.038	0.002	6.09e-71
rs55666429	6	137113137	A	G	0.588	-0.016	0.002	1.96e-16
rs2797681	6	137593036	C	G	0.256	-0.015	0.002	1.14e-10
rs149110519	6	144385777	T	C	0.036	0.075	0.005	1.51e-48
rs9390460	6	147694334	C	T	0.538	0.012	0.002	3.01e-10
rs11155787	6	151686905	T	C	0.638	0.014	0.002	3.92e-12
rs2817441	6	156938699	T	C	0.279	-0.022	0.002	4.67e-25
rs73784287	6	160980401	G	A	0.149	0.015	0.003	4.93e-09
rs707845	6	16711855	G	A	0.511	-0.017	0.002	6.09e-19
rs3012415	6	170489464	T	C	0.823	0.020	0.003	1.37e-15
rs2273215	6	170586082	A	G	0.458	-0.018	0.002	2.72e-20
rs6940465	6	21207748	A	G	0.151	0.016	0.003	1.61e-09
rs78912080	6	25616453	G	A	0.075	0.026	0.004	5.55e-13
rs12530071	6	2886067	C	T	0.225	-0.015	0.002	5.78e-11
rs2853999	6	31326074	T	A	0.138	-0.076	0.003	2.93e-171
rs17207748	6	31863168	C	T	0.047	-0.035	0.004	2.3e-15
rs56007794	6	41990827	T	A	0.244	0.039	0.002	4.77e-72
rs11963621	6	44591006	C	T	0.101	-0.048	0.003	6.15e-54
rs115202835	6	45533239	A	G	0.068	0.027	0.004	4.52e-13
rs10948314	6	46911131	G	A	0.103	-0.034	0.003	1.04e-27
rs644492	6	53444319	G	A	0.196	-0.020	0.002	1.84e-17
rs3761986	6	6680510	C	T	0.394	-0.012	0.002	4.88e-10
rs9379077	6	7167170	G	A	0.201	0.029	0.002	1.46e-34
rs915125	6	82463376	T	C	0.282	0.034	0.002	4.34e-59
rs62470670	7	114651694	G	A	0.092	-0.023	0.003	5.3e-13
rs7803075	7	130742066	G	A	0.735	-0.014	0.002	2.28e-11
rs7785014	7	137881690	C	T	0.671	-0.025	0.002	2.04e-36
rs7787179	7	143081833	G	A	0.207	0.021	0.002	3.31e-19
rs62491927	7	149172894	A	C	0.257	-0.013	0.002	1.42e-09
rs17700436	7	17167825	T	C	0.057	-0.028	0.004	1.53e-12
rs34947605	7	2581594	A	G	0.172	0.015	0.002	3.39e-09
rs62454420	7	27191804	G	A	0.070	-0.035	0.004	2.39e-21
rs798563	7	2757867	C	A	0.297	0.018	0.002	3.23e-17
rs17156536	7	28279488	A	C	0.159	0.025	0.003	8.66e-22
rs10238435	7	28724491	T	C	0.240	-0.024	0.002	4.23e-25
rs112248289	7	44770213	C	A	0.129	0.022	0.003	4.83e-15
rs2136769	7	47469152	C	A	0.449	0.011	0.002	2.34e-09
rs4385425	7	50307334	G	A	0.324	-0.060	0.002	1.28e-198
rs149007767	7	50370254	T	C	0.162	-0.072	0.003	1.97e-163
rs11766800	7	50435617	T	A	0.322	0.019	0.002	2.26e-21
rs41430449	7	50798525	G	C	0.066	-0.030	0.004	1.82e-15
rs6796	7	6502367	C	T	0.274	0.046	0.002	1.66e-102
rs7786376	7	73042614	G	A	0.276	0.012	0.002	4.73e-09
rs178405	7	73513045	T	G	0.784	-0.015	0.002	1.6e-10
rs445	7	92408370	T	C	0.098	-0.094	0.003	3.59e-196
rs6465661	7	97885213	T	C	0.492	-0.016	0.002	5.49e-18
rs7792525	7	99972122	G	A	0.186	0.019	0.002	4.21e-15
rs72673751	8	106578940	C	T	0.190	-0.017	0.002	4.35e-12
rs1954735	8	108239325	C	T	0.168	0.021	0.003	1.01e-16
rs13271228	8	116597409	G	T	0.567	-0.031	0.002	4.35e-59
rs12334935	8	126617990	A	G	0.471	-0.015	0.002	2.75e-15
rs7843207	8	130570063	A	C	0.506	-0.069	0.002	1e-200
rs10101101	8	130691767	A	G	0.139	-0.041	0.003	1.44e-50
rs4397371	8	130701417	T	C	0.734	-0.018	0.002	1.17e-17
rs837227	8	130964654	T	C	0.745	-0.015	0.002	1.11e-11

rs10099546	8	144638734	G	A	0.143	-0.020	0.003	4.33e-13
rs4871844	8	22879734	C	T	0.343	0.016	0.002	1.27e-14
rs62501136	8	23089429	A	G	0.221	0.020	0.002	1.72e-19
rs62502392	8	27190945	A	G	0.143	-0.018	0.003	2.08e-11
rs4737009	8	41630405	A	G	0.236	0.015	0.002	3.44e-12
rs113155021	8	43719739	A	G	0.015	-0.048	0.008	2.21e-09
rs145718079	8	48641192	A	G	0.010	-0.080	0.010	4.68e-16
rs45577137	8	48651633	G	A	0.044	-0.065	0.005	9.05e-39
rs113015223	8	57037166	T	C	0.211	0.019	0.002	1.06e-15
rs3808609	8	59465377	C	G	0.318	0.017	0.002	3.86e-17
rs7824937	8	61392724	G	A	0.369	0.018	0.002	1.03e-19
rs7826487	8	6880925	G	A	0.116	-0.041	0.003	2.17e-43
rs12542907	8	68813188	G	C	0.399	-0.024	0.002	1.01e-36
rs13267464	8	6906925	T	C	0.399	-0.020	0.002	8.9e-27
rs16939607	8	79013333	A	G	0.146	-0.025	0.003	2.43e-21
rs1863651	8	82066998	T	A	0.699	-0.017	0.002	4.32e-16
rs13292863	9	113316712	G	C	0.155	-0.016	0.003	5.77e-10
rs1008158	9	113828811	G	A	0.343	-0.067	0.002	1e-200
rs10817147	9	113864075	T	C	0.377	-0.059	0.002	1e-200
rs77155602	9	114153208	T	C	0.180	0.029	0.002	3.25e-33
rs13293713	9	114353573	A	G	0.081	0.052	0.004	1.83e-50
rs72759267	9	126971204	T	C	0.159	0.021	0.003	5.36e-16
rs2491104	9	130313823	A	T	0.242	-0.014	0.002	3.17e-10
rs79086185	9	130666538	C	A	0.039	0.031	0.005	1.17e-09
rs11792030	9	130732588	A	G	0.104	0.022	0.003	1e-12
rs676996	9	136146077	G	T	0.320	-0.044	0.002	5.13e-106
rs3731211	9	21986847	A	T	0.720	0.028	0.002	2.51e-42
rs12376511	9	22142756	C	T	0.164	-0.049	0.003	3.44e-82
rs34881325	9	2622134	T	C	0.379	0.010	0.002	8.15e-11
rs11557154	9	34107505	T	C	0.128	0.032	0.003	2.64e-30
rs10814193	9	35018686	C	A	0.716	0.015	0.002	1.07e-12
rs2236288	9	35749845	C	G	0.229	0.013	0.002	1.78e-09
rs10758481	9	38195756	C	T	0.502	-0.014	0.002	4.63e-14
rs10780209	9	91472127	A	G	0.479	0.045	0.002	5.69e-131
rs9410425	9	91562311	A	G	0.325	-0.030	0.002	3.23e-51
rs290243	9	93571337	A	G	0.215	0.014	0.002	3e-10
rs34746889	16	67221444	G	C	0.983	0.058	0.007	2e-15
rs75726056	1	150859753	A	G	0.988	-0.072	0.008	2.11e-17
rs115240699	6	33743079	A	G	0.993	-0.067	0.011	2.97e-09
rs10909370	10	115847890	T	G	0.464	-0.012	0.002	4.76e-09
rs2343551	10	82267945	C	A	0.795	0.021	0.002	1.29e-18
rs2651780	11	2345261	A	G	0.850	0.022	0.003	5.51e-15
rs11227312	11	65586597	G	C	0.485	0.012	0.002	3.87e-10
rs113681054	12	21402979	C	T	0.161	-0.016	0.003	3.31e-10
rs2535403	12	718885	C	T	0.557	-0.016	0.002	7.7e-16
rs374039502	13	108960385	A	T	0.022	-0.116	0.007	4.16e-61
rs113235072	14	35344040	C	G	0.071	0.036	0.004	2.06e-21
rs138841053	14	59985478	C	T	0.406	0.012	0.002	4.54e-10
rs2300598	14	75359229	C	T	0.313	0.017	0.002	1.29e-16
rs8037798	15	75240030	G	T	0.231	0.022	0.002	5.84e-22
rs2562750	15	80250606	A	T	0.759	0.070	0.002	1e-200
rs10871483	16	29592725	A	T	0.521	-0.015	0.002	1.49e-11
rs8054439	16	4146007	C	G	0.117	0.024	0.003	1.78e-15
rs12924604	16	74601486	A	G	0.198	-0.017	0.002	8.24e-13
rs181791	16	84558889	T	C	0.164	0.026	0.003	2.94e-18
rs7223589	17	29728284	C	T	0.616	0.014	0.002	1.93e-12

rs113232639	18	20715656	A	G	0.489	-0.018	0.002	5.66e-19
rs12151289	19	33751852	C	G	0.031	-0.085	0.006	1.13e-46
rs62113155	19	36752859	T	G	0.201	-0.019	0.002	1.33e-14
rs12972849	19	54338067	A	G	0.652	0.051	0.002	7.41e-95
rs61242663	19	836059	T	C	0.260	0.026	0.002	5.69e-27
rs28549287	1	110230138	A	G	0.761	-0.017	0.003	1.17e-11
rs7512283	1	161000113	G	A	0.795	0.017	0.002	2.51e-12
rs61804163	1	161622861	C	T	0.296	-0.020	0.002	3.69e-19
rs7542018	1	243972240	A	G	0.887	0.022	0.003	1.48e-12
rs7529978	1	53204705	A	G	0.470	-0.017	0.002	5.28e-19
rs2815358	1	67493778	C	T	0.650	-0.018	0.002	9.15e-18
rs192009275	20	30250942	T	C	0.257	-0.021	0.002	1.08e-20
rs6055629	20	8178309	A	C	0.485	-0.012	0.002	7.12e-10
rs180765893	2	182372755	A	T	0.017	0.048	0.008	1.48e-09
rs185880215	2	43549887	T	C	0.069	0.023	0.004	2.72e-09
rs4683611	3	141290903	C	T	0.203	-0.016	0.003	4.7e-09
rs549046531	4	145975794	T	C	0.272	-0.019	0.002	4.49e-16
rs565245327	6	31614543	T	G	0.471	-0.045	0.003	8.78e-70
rs28474755	6	32623919	T	C	0.121	0.042	0.003	2.23e-45
rs6968441	7	107151877	C	G	0.467	0.012	0.002	2.8e-10
rs6950899	7	65286649	A	G	0.370	0.015	0.002	3.74e-12
rs201446427	7	6851361	C	A	0.197	0.019	0.003	1.86e-11
rs71518544	8	11782349	T	G	0.404	0.016	0.002	1.09e-14
rs2978891	8	6697258	A	T	0.505	-0.020	0.002	1.51e-25
rs7020171	9	91776952	G	T	0.248	-0.014	0.002	9.16e-10
rs549758081	1	93691855	D	I	0.433	-0.016	0.002	2.22e-12

Table S11. 393 SNPs significantly associated with lymphocyte count used IVs in forward MR analyses derived from Vuckovic D et al. Chr: Chromosome; EA: Effect allele; NEA: Non-effect allele; EAF: Effect allele frequency.

SNP	Chr	Pos	EA	NEA	EAF	Beta	SE	P-value
rs159963	1	8504421	A	C	0.583	-0.019	0.002	3.8e-19
rs753612	1	19978895	G	A	0.611	0.014	0.002	4.9e-11
rs7517330	1	23830340	A	C	0.152	0.020	0.003	9.1e-11
rs12750971	1	24219590	T	C	0.758	-0.022	0.003	2.4e-16
rs9438860	1	25224957	C	G	0.410	-0.016	0.002	3.4e-14
rs11247712	1	28229681	T	C	0.369	-0.020	0.002	8.8e-21
rs77425607	1	39544876	A	G	0.333	-0.014	0.002	1.5e-09
rs4068540	1	40398941	G	A	0.267	0.017	0.002	1.8e-12
rs5011302	1	56907563	C	T	0.544	-0.020	0.002	2.2e-21
rs3014983	1	65460691	T	C	0.761	-0.019	0.003	6e-14
rs6700896	1	66089782	T	C	0.378	-0.018	0.002	1e-16
rs2755253	1	67470843	T	C	0.707	-0.032	0.002	6.5e-43
rs2635119	1	79352340	C	T	0.688	0.017	0.002	6.5e-14
rs12741113	1	101147149	C	T	0.447	-0.018	0.002	1e-16
rs6696259	1	101221482	C	G	0.410	0.035	0.002	3.5e-59
rs377345022	1	101653213	T	A	0.265	-0.019	0.002	1e-13
rs10875371	1	101762529	T	C	0.420	-0.022	0.002	2.4e-24
rs2800883	1	112143035	C	T	0.378	-0.014	0.002	1.2e-10
rs2476601	1	114377568	G	A	0.899	0.058	0.003	4.4e-62
rs4970926	1	150673684	C	T	0.513	-0.027	0.002	3.1e-38
rs2157691	1	158582838	C	G	0.266	0.032	0.002	3.3e-42
rs12405457	1	160759052	G	A	0.215	0.026	0.003	7.1e-25
rs11746	1	161683136	A	G	0.487	0.019	0.002	7.4e-20
rs10918048	1	164558512	T	A	0.122	-0.020	0.003	7.9e-10
rs1689796	1	182151236	G	T	0.334	0.018	0.002	2.5e-15
rs188626852	1	198498784	A	C	0.013	0.074	0.010	7.4e-14
rs10494783	1	198663661	A	G	0.051	-0.054	0.005	3.9e-29
rs17612648	1	198665917	G	C	0.015	0.061	0.009	5.1e-11
rs3861929	1	201025850	T	C	0.605	-0.016	0.002	3.7e-14
rs4531346	1	204269185	A	G	0.268	-0.020	0.002	3.5e-16
rs7548606	1	212388607	G	A	0.626	0.014	0.002	2.1e-10
rs12745411	1	214405704	T	G	0.599	0.018	0.002	3.2e-16
rs12021809	1	225941782	A	G	0.109	-0.027	0.003	1.3e-15
rs4659611	1	236033115	T	A	0.744	-0.018	0.002	3.5e-13
rs74227709	1	247722588	A	G	0.072	-0.030	0.004	2.8e-13
rs6721663	2	7615061	A	G	0.159	-0.026	0.003	1.2e-19
rs7572278	2	8563029	A	T	0.203	0.023	0.003	1.2e-18
rs56243142	2	11333606	C	T	0.547	0.015	0.002	3.9e-13
rs730126	2	12891476	C	A	0.414	0.015	0.002	1.2e-12
rs72781679	2	24237712	A	G	0.135	0.061	0.003	3.1e-87
rs4407213	2	25541636	C	G	0.032	0.045	0.006	2.2e-13
rs1260326	2	27730940	C	T	0.605	-0.024	0.002	5.8e-28
rs113542380	2	43464818	A	G	0.075	-0.070	0.004	2.6e-66
rs805316	2	54133744	C	T	0.276	-0.015	0.002	1.4e-10
rs2920880	2	55297818	T	C	0.594	0.016	0.002	3.5e-13
rs12991188	2	62554797	G	A	0.599	0.014	0.002	2.3e-10
rs4852777	2	71534161	C	G	0.595	0.013	0.002	1.4e-09
rs6755786	2	103048103	T	C	0.774	0.028	0.003	1.1e-27
rs13019266	2	111604642	C	T	0.679	0.031	0.002	1e-41
rs1976055	2	111787853	A	C	0.157	-0.017	0.003	3.1e-09
rs199898421	2	112193161	G	A	0.059	0.038	0.006	1.1e-09

rs55706446	2	112239082	T	C	0.075	-0.074	0.004	1.7e-66
rs2241978	2	113954006	G	A	0.482	0.021	0.002	1.4e-23
rs11688303	2	128410244	T	C	0.118	0.020	0.003	2e-09
rs71420836	2	129060871	T	C	0.096	0.033	0.004	2.8e-20
rs73961715	2	143803847	C	T	0.129	-0.051	0.003	3.9e-59
rs34030812	2	144248905	C	T	0.367	-0.019	0.002	3.2e-18
rs10173412	2	161330940	C	T	0.201	-0.042	0.003	2.4e-56
rs2075302	2	163076146	C	T	0.453	-0.015	0.002	2.1e-12
rs74401397	2	181944580	G	C	0.042	-0.038	0.005	3.9e-13
rs62180252	2	182175221	C	T	0.250	0.052	0.002	6.6e-101
rs10179705	2	188315234	A	G	0.292	-0.022	0.002	4.2e-21
rs3769818	2	202151163	G	A	0.732	0.033	0.002	1.1e-43
rs11888947	2	204642460	A	T	0.730	-0.015	0.002	1.2e-10
rs715	2	211543055	C	T	0.311	-0.015	0.002	3.3e-11
rs13018173	2	216248606	C	T	0.232	-0.015	0.002	2.9e-09
rs78058190	2	219699999	A	G	0.050	0.048	0.005	1e-18
rs978917	2	223974443	A	G	0.616	0.017	0.002	2.1e-15
rs11676298	2	227291731	G	C	0.192	0.026	0.003	6.5e-22
rs4973318	2	231222675	C	T	0.106	0.022	0.003	9e-11
rs58106596	2	232579379	A	G	0.222	-0.024	0.003	2e-21
rs13016839	2	234357513	G	A	0.106	-0.026	0.003	6.8e-14
rs9826367	3	12294202	G	A	0.449	-0.035	0.002	3.3e-61
rs4261897	3	18764304	C	T	0.296	0.021	0.002	8.9e-19
rs13096529	3	24232035	T	C	0.191	0.020	0.003	1.5e-13
rs2887944	3	27758275	T	G	0.435	0.024	0.002	2e-28
rs75033378	3	28295653	T	C	0.175	0.037	0.003	4.2e-39
rs4676482	3	39237190	T	A	0.190	0.016	0.003	3.8e-09
rs11706384	3	39296881	T	G	0.236	0.026	0.002	4.4e-25
rs13063578	3	47087837	A	T	0.401	0.054	0.002	2.6e-125
rs7637449	3	56628031	A	G	0.529	0.015	0.002	3.4e-13
rs12485738	3	56865776	G	A	0.629	0.017	0.002	5.5e-15
rs35592432	3	71355240	C	G	0.027	0.107	0.007	1.6e-55
rs6809697	3	71414864	A	G	0.135	-0.020	0.003	2.5e-10
rs6782672	3	108506049	G	A	0.659	0.016	0.002	8.4e-13
rs59107033	3	123105643	T	C	0.225	-0.025	0.003	8.6e-24
rs332507	3	124435110	C	T	0.220	-0.018	0.003	2e-12
rs11712335	3	128210550	C	T	0.270	0.016	0.002	2.3e-11
rs9834250	3	136561899	G	C	0.687	0.020	0.002	1.5e-18
rs7625643	3	141150026	G	A	0.446	0.025	0.002	1.9e-31
rs9863821	3	151007175	T	C	0.775	0.026	0.003	9.7e-24
rs2647929	3	159693570	C	T	0.567	0.015	0.002	1e-11
rs2291900	3	170825920	A	G	0.293	0.017	0.002	1.3e-12
rs7627511	3	171178037	C	G	0.217	-0.018	0.003	2.1e-11
rs10937106	3	182738287	A	G	0.713	-0.015	0.002	5.3e-10
rs77265382	3	185546150	T	C	0.045	-0.036	0.005	2.8e-12
rs2089979	3	196501413	G	A	0.416	-0.036	0.002	3.2e-61
rs1344603	4	38109847	C	T	0.675	0.031	0.002	2.6e-42
rs1386623	4	38372043	T	G	0.681	0.068	0.002	8.5e-198
rs13105987	4	38602467	A	G	0.277	0.050	0.002	3.6e-98
rs73142294	4	40301624	T	C	0.249	0.016	0.002	1.7e-10
rs7437961	4	55066758	C	T	0.236	0.015	0.002	1.7e-09
rs7665147	4	57767327	A	T	0.186	-0.023	0.003	1.1e-17
rs2298850	4	72614267	C	G	0.279	-0.016	0.002	1.4e-11
rs7662083	4	80933308	A	G	0.529	-0.031	0.002	1.2e-48
rs17005891	4	83547862	A	G	0.184	-0.047	0.003	7.5e-66
rs17703261	4	88058337	T	A	0.187	0.020	0.003	2.4e-13

rs1431545	4	90220659	T	C	0.654	0.021	0.002	1.6e-20
rs909349	4	103556316	C	T	0.521	0.039	0.002	6.4e-76
rs62332762	4	106143492	T	C	0.399	-0.027	0.002	2.4e-35
rs2237040	4	110838862	T	C	0.404	-0.019	0.002	2.1e-18
rs72684793	4	123024769	A	C	0.300	0.015	0.002	1.7e-10
rs309375	4	123681156	A	C	0.572	0.018	0.002	1.5e-16
rs112737912	4	149366675	T	G	0.360	-0.014	0.002	1.6e-10
rs4696314	4	153005716	C	T	0.512	0.016	0.002	1.3e-13
rs419470	5	16625591	G	A	0.716	0.016	0.002	2.8e-11
rs6859682	5	34668797	G	A	0.331	-0.014	0.002	2.1e-09
rs11567699	5	35859011	G	C	0.260	0.056	0.002	2.8e-117
rs2548499	5	52072544	G	A	0.208	0.020	0.003	3.7e-14
rs2408014	5	54864017	T	A	0.681	-0.016	0.002	7.9e-12
rs151912	5	57607142	T	A	0.596	-0.013	0.002	1.1e-09
rs251398	5	67509801	C	T	0.451	-0.017	0.002	2.5e-16
rs678393	5	72418309	G	T	0.261	-0.017	0.002	5.1e-12
rs2544920	5	100141374	T	A	0.617	0.014	0.002	6.6e-11
rs78270096	5	110517419	A	G	0.070	-0.032	0.004	2.5e-13
rs73239278	5	118695051	A	G	0.202	-0.032	0.003	7.4e-34
rs251390	5	127538346	A	G	0.749	0.021	0.002	4.7e-18
rs58835386	5	131600559	G	A	0.567	-0.023	0.002	4.1e-26
rs75925066	5	134722833	A	C	0.023	0.087	0.007	1.5e-35
rs60580948	5	148340270	G	A	0.139	-0.020	0.003	5.4e-11
rs3815725	5	150518532	G	A	0.519	-0.016	0.002	5.4e-14
rs17055818	5	157969475	C	T	0.254	-0.032	0.002	5.5e-39
rs34066608	5	158360881	C	A	0.098	-0.035	0.004	2e-22
rs6882776	5	172664163	A	G	0.283	0.014	0.002	3.5e-09
rs360017	5	173207353	G	A	0.775	-0.022	0.003	4.7e-18
rs10063803	5	176667327	A	C	0.832	0.025	0.003	3.3e-19
rs872071	6	411064	G	A	0.528	0.025	0.002	2.2e-32
rs9392840	6	6901582	T	C	0.272	0.026	0.002	2.9e-26
rs16870458	6	10545728	A	G	0.221	-0.016	0.003	4.1e-10
rs17119	6	14719496	A	G	0.804	-0.018	0.003	3e-11
rs52484	6	23564366	G	A	0.532	-0.014	0.002	5.3e-11
rs6927418	6	24827912	T	C	0.556	-0.025	0.002	6.4e-32
rs2523855	6	31022158	C	G	0.331	0.071	0.002	1e-200
rs9265552	6	31298296	G	C	0.249	-0.070	0.003	6.3e-142
rs377181093	6	31422893	G	A	0.092	0.051	0.004	5.4e-39
rs200801362	6	31555480	C	T	0.161	-0.081	0.004	2.5e-94
rs9272557	6	32607048	C	T	0.242	-0.057	0.003	1.9e-111
rs9942489	6	35323709	A	T	0.037	-0.076	0.006	5.2e-42
rs9381130	6	42040606	A	G	0.107	0.027	0.003	6.1e-15
rs4711790	6	44573822	T	C	0.341	-0.019	0.002	7.4e-18
rs2294861	6	53136516	T	C	0.294	0.018	0.002	1.7e-14
rs72928038	6	90976768	A	G	0.179	-0.036	0.003	4e-38
rs12526696	6	108053364	A	G	0.161	0.039	0.003	1.4e-41
rs6929208	6	109720405	C	G	0.298	-0.015	0.002	1.1e-10
rs6926219	6	122720806	A	G	0.547	0.025	0.002	1.7e-32
rs9375486	6	127388186	T	C	0.377	-0.013	0.002	1.6e-09
rs2327531	6	135048564	G	A	0.208	0.017	0.003	2.7e-11
rs35786788	6	135419042	A	G	0.253	-0.041	0.002	8.6e-62
rs7763994	6	151912354	T	A	0.290	0.014	0.002	1.5e-09
rs17710008	6	153043035	A	G	0.184	0.020	0.003	1.7e-13
rs41267765	6	159462424	T	C	0.021	-0.048	0.007	3.3e-11
rs2273215	6	170586082	A	G	0.459	-0.016	0.002	6e-14
rs10480059	7	970035	T	C	0.518	-0.016	0.002	1.4e-13

rs4719654	7	2760008	C	G	0.442	-0.015	0.002	1e-11
rs852432	7	5562740	G	A	0.367	0.014	0.002	3.4e-10
rs6796	7	6502367	C	T	0.278	0.028	0.002	2.3e-33
rs35345753	7	22740513	G	C	0.209	-0.027	0.003	5e-25
rs4719922	7	28158058	T	C	0.201	-0.025	0.003	8.4e-21
rs2710804	7	36084529	C	T	0.377	0.015	0.002	1.4e-11
rs73112675	7	37379030	G	A	0.180	0.019	0.003	5.9e-12
rs3735485	7	45009341	G	A	0.845	0.045	0.003	2.8e-54
rs150640087	7	50444152	T	G	0.016	0.061	0.009	1.1e-12
rs10237524	7	56036024	A	G	0.330	-0.019	0.002	6.4e-18
rs4718976	7	70113409	C	T	0.695	-0.016	0.002	5.2e-12
rs2530482	7	74300890	C	A	0.285	0.016	0.002	1e-10
rs7780424	7	77211703	T	A	0.609	-0.014	0.002	4.9e-10
rs10230506	7	92471518	A	T	0.160	0.019	0.003	3.1e-11
rs11551890	7	97845713	A	G	0.489	0.013	0.002	7.8e-10
rs7780328	7	99256844	G	A	0.067	0.040	0.004	1.2e-21
rs9920	7	116200092	C	T	0.105	0.033	0.003	9.5e-22
rs62621812	7	127015083	A	G	0.020	0.058	0.008	5e-14
rs2727487	7	129513820	C	A	0.629	-0.014	0.002	4.8e-10
rs7793008	7	130747306	G	A	0.205	0.022	0.003	2.6e-17
rs62485905	7	138741586	T	C	0.551	-0.018	0.002	2e-17
rs55700285	7	150209636	T	G	0.231	0.025	0.003	8.1e-23
rs9886191	7	156656661	T	C	0.431	-0.014	0.002	7.5e-11
rs28588745	8	10647044	T	A	0.206	0.024	0.003	3.4e-20
rs6586777	8	18702471	A	C	0.436	-0.013	0.002	6.8e-10
rs533703709	8	21736272	T	A	0.042	-0.043	0.005	4.8e-16
rs2313571	8	22789184	T	C	0.348	0.019	0.002	4.2e-17
rs1567124	8	25244326	A	C	0.310	0.015	0.002	5.5e-11
rs6474359	8	41549194	C	T	0.038	-0.048	0.006	3.9e-18
rs4737010	8	41630447	A	G	0.229	0.028	0.003	2.2e-28
rs1976451	8	49456791	G	T	0.133	-0.019	0.003	3.5e-09
rs200523234	8	59501137	C	A	0.296	0.014	0.002	1.6e-09
rs948421	8	61403965	C	T	0.376	0.014	0.002	4.7e-11
rs6982175	8	61812553	C	T	0.879	-0.023	0.003	5.1e-12
rs894885	8	72908283	T	G	0.238	-0.019	0.002	4.4e-14
rs75653581	8	78363128	T	C	0.013	0.068	0.010	2.4e-12
rs117934175	8	79069520	A	G	0.040	-0.043	0.005	3.1e-15
rs1441850	8	79657666	C	T	0.249	0.052	0.002	2.1e-99
rs17424046	8	101372316	T	C	0.135	0.030	0.003	1.4e-21
rs543427	8	102468599	C	G	0.491	0.016	0.002	1.9e-14
rs6995717	8	106229410	C	T	0.449	-0.013	0.002	4.8e-10
rs72680380	8	119932741	A	T	0.466	-0.013	0.002	3.2e-10
rs2954025	8	126484463	T	C	0.649	-0.015	0.002	5.2e-12
rs10956401	8	129002419	A	G	0.343	-0.025	0.002	9.3e-30
rs837223	8	130971463	T	C	0.343	-0.017	0.002	6.1e-14
rs7007986	8	142217115	A	G	0.602	-0.022	0.002	1.1e-24
rs7033677	9	4049942	G	A	0.640	-0.020	0.002	1.5e-18
rs10815097	9	4858114	G	A	0.566	-0.013	0.002	4.8e-10
rs12336678	9	21785894	T	C	0.098	-0.023	0.004	1.1e-10
rs3731211	9	21986847	A	T	0.722	0.046	0.002	4.5e-86
rs2065500	9	22145694	G	A	0.158	-0.051	0.003	1.4e-69
rs11541908	9	35705759	A	G	0.279	0.022	0.002	2.4e-21
rs12343705	9	38197187	T	A	0.490	-0.020	0.002	9.3e-22
rs2807303	9	82187095	A	G	0.346	0.022	0.002	1.2e-22
rs35010780	9	86499923	A	G	0.129	-0.022	0.003	7.7e-12
rs61750929	9	91495135	T	C	0.056	-0.076	0.005	6e-60

rs6477734	9	112728014	C	T	0.490	-0.015	0.002	1e-12
rs59364245	9	113897765	C	T	0.559	-0.013	0.002	3.6e-09
rs3761846	9	123689597	T	C	0.569	0.021	0.002	3.3e-23
rs10985915	9	126018715	G	A	0.112	-0.023	0.003	1.6e-11
rs2791737	9	133632758	G	A	0.398	-0.015	0.002	3.2e-12
rs2427964	9	136931957	C	T	0.302	0.015	0.002	2e-10
rs17250351	9	139801262	A	G	0.011	0.088	0.010	9.1e-18
rs61839660	10	6094697	T	C	0.098	-0.046	0.004	8.5e-39
rs3824667	10	8100125	G	A	0.827	0.027	0.003	3.1e-21
rs11593057	10	14887979	T	C	0.161	-0.018	0.003	1.7e-10
rs10828725	10	25218243	T	G	0.369	-0.033	0.002	1.7e-50
rs2992263	10	26741207	A	G	0.609	-0.020	0.002	2.9e-20
rs2091084	10	30492267	C	T	0.653	0.014	0.002	1.3e-09
rs72790861	10	44880207	G	C	0.308	-0.018	0.002	7e-15
rs61850684	10	50252936	A	G	0.235	-0.034	0.003	6.4e-42
rs4948492	10	63719739	T	C	0.660	-0.024	0.002	5.7e-26
rs224082	10	64566258	T	C	0.377	-0.015	0.002	4.1e-12
rs10762264	10	70976833	A	G	0.685	-0.021	0.002	1.5e-20
rs10823631	10	72625741	G	A	0.326	-0.019	0.002	2.1e-16
rs748113	10	73508791	C	T	0.436	-0.035	0.002	1.3e-61
rs1878036	10	82280137	G	T	0.794	0.018	0.003	2.4e-11
rs11187162	10	94508636	A	G	0.161	0.021	0.003	7.7e-13
rs58667319	10	96524395	T	C	0.123	-0.024	0.003	1.8e-13
rs41317270	10	98369755	T	C	0.177	-0.018	0.003	3.2e-11
rs11189058	10	99001258	A	G	0.661	0.014	0.002	2.7e-10
rs603424	10	102075479	A	G	0.171	0.021	0.003	4.1e-14
rs35896146	10	116024458	A	G	0.159	0.024	0.003	4e-16
rs7071131	10	121026712	G	A	0.495	-0.017	0.002	4.1e-15
rs10794175	10	126358073	T	G	0.415	-0.022	0.002	3.3e-24
rs574185	11	221322	T	G	0.538	0.017	0.002	2.8e-16
rs7945319	11	405197	A	G	0.553	0.014	0.002	1.2e-10
rs61867141	11	1874892	A	G	0.195	-0.020	0.003	1.1e-13
rs4756265	11	36078171	G	A	0.418	0.020	0.002	1.1e-19
rs10838634	11	46929954	G	A	0.902	-0.026	0.004	5.8e-13
rs7123436	11	48013484	A	G	0.201	-0.038	0.003	7e-48
rs547957008	11	60258999	T	A	0.517	0.017	0.002	4.7e-16
rs74679312	11	62194434	G	A	0.058	0.032	0.005	5.4e-12
rs949349	11	86297189	C	T	0.270	0.014	0.002	4.9e-09
rs1111890	11	100501571	G	C	0.368	0.023	0.002	1.7e-25
rs1800057	11	108143456	G	C	0.027	0.040	0.007	1.1e-09
rs58432776	11	122514281	A	C	0.380	0.032	0.002	1.8e-49
rs35871881	11	123356451	G	A	0.225	0.016	0.003	2.1e-10
rs10790962	11	128385169	A	G	0.469	0.023	0.002	2.5e-26
rs34038797	12	740009	G	C	0.479	-0.021	0.002	8.8e-22
rs10466905	12	6502832	A	G	0.190	0.060	0.003	2.7e-110
rs768908	12	10035304	T	C	0.125	-0.020	0.003	2.5e-10
rs34325	12	12877692	C	T	0.524	-0.027	0.002	4.1e-36
rs7134738	12	26358170	C	T	0.351	-0.017	0.002	4.7e-14
rs1900942	12	26831928	G	C	0.839	0.018	0.003	2e-10
rs11168249	12	48208368	C	T	0.461	-0.018	0.002	8e-18
rs73108566	12	49080698	A	T	0.329	-0.019	0.002	1.4e-16
rs1131017	12	56435929	G	C	0.572	0.014	0.002	3.6e-11
rs4913407	12	68560246	T	G	0.705	0.016	0.002	5.2e-12
rs6538189	12	89885923	A	C	0.239	0.016	0.002	7.5e-11
rs17041439	12	101873240	C	A	0.056	0.043	0.005	2e-20
rs4764803	12	101898230	G	C	0.616	0.015	0.002	7.5e-12

rs1265564	12	111708458	C	A	0.441	0.073	0.002	1e-200
rs73207626	12	112686873	G	A	0.043	-0.035	0.005	1.9e-11
rs373582220	12	112847812	A	G	0.372	-0.035	0.003	6e-38
rs1293782	12	113463594	A	C	0.643	-0.015	0.002	1.4e-11
rs6488868	12	123799974	G	A	0.738	-0.027	0.002	5.3e-29
rs67516712	12	124201524	A	G	0.425	0.016	0.002	4.1e-14
rs3768	12	124499839	T	C	0.194	-0.018	0.003	5.2e-11
rs76428106	13	28604007	C	T	0.013	0.063	0.010	7.7e-11
rs9532679	13	41522338	C	A	0.150	-0.032	0.003	1.7e-26
rs9533100	13	42996548	T	G	0.536	-0.021	0.002	9.3e-23
rs2146880	13	46706598	A	C	0.461	0.015	0.002	3.4e-13
rs9316484	13	50645164	C	T	0.220	-0.028	0.003	3.8e-27
rs67483792	13	72503638	T	C	0.043	-0.036	0.005	1.3e-11
rs9592965	13	74615925	C	A	0.327	-0.015	0.002	1.3e-10
rs3812849	13	74701736	C	A	0.264	0.045	0.002	2.7e-76
rs9513573	13	99815898	A	G	0.279	-0.015	0.002	3.2e-10
rs1320472	13	114843162	C	T	0.460	-0.014	0.002	4.2e-11
rs9590390	13	114920867	A	G	0.294	0.030	0.002	1.4e-37
rs45528934	14	23793305	T	C	0.162	-0.019	0.003	7.9e-11
rs2057340	14	35848774	G	A	0.655	0.016	0.002	1.2e-12
rs696	14	35871093	T	C	0.363	0.027	0.002	2.3e-34
rs11848179	14	64977053	G	A	0.318	0.030	0.002	2.1e-38
rs10138752	14	69179971	T	C	0.078	-0.029	0.004	3.2e-13
rs175714	14	75981856	C	T	0.577	0.027	0.002	5.1e-36
rs7157267	14	103350813	C	T	0.221	0.030	0.003	9.5e-33
rs12882130	14	103878774	G	C	0.384	0.017	0.002	5e-15
rs11855923	15	40892601	A	G	0.392	-0.017	0.002	1.5e-15
rs139974673	15	44027885	C	T	0.026	0.054	0.007	1.8e-15
rs12593807	15	48546727	C	T	0.135	-0.022	0.003	6.8e-13
rs7161799	15	58770523	T	C	0.075	0.038	0.004	1.3e-20
rs149453951	15	69583434	T	C	0.024	0.048	0.007	3.6e-11
rs34025077	15	70033578	G	A	0.103	-0.033	0.003	2.1e-21
rs631864	15	70376441	C	T	0.472	0.014	0.002	5.9e-11
rs17507693	15	83793622	T	A	0.222	0.017	0.003	2.6e-11
rs17832339	15	90557494	T	G	0.039	0.050	0.005	1.4e-19
rs8030465	15	90656372	G	C	0.573	-0.013	0.002	2e-09
rs28539372	15	91021412	A	T	0.319	0.040	0.002	1.3e-69
rs447361	16	4148240	G	T	0.669	0.020	0.002	2.7e-18
rs1677490	16	9028541	C	G	0.794	0.016	0.003	1.7e-09
rs17229044	16	11062936	T	C	0.208	-0.019	0.003	7.2e-13
rs11643225	16	24046655	T	G	0.059	-0.033	0.005	2e-13
rs11574938	16	30485393	C	G	0.517	0.057	0.002	1.1e-155
rs28853644	16	30801027	T	C	0.270	-0.025	0.002	2.3e-26
rs9746394	16	31300769	C	T	0.270	-0.016	0.002	2.9e-11
rs7192652	16	57075180	G	A	0.478	0.022	0.002	1.1e-25
rs80180406	16	67435558	A	G	0.023	-0.047	0.007	3.6e-11
rs117556162	16	67680806	A	G	0.059	0.044	0.004	1.4e-22
rs8058619	16	75182354	C	G	0.308	-0.018	0.002	2.7e-14
rs11150084	16	78561820	G	C	0.669	0.015	0.002	1.9e-11
rs247826	16	84582965	T	C	0.222	0.037	0.003	7.9e-48
rs8060375	16	88507538	T	C	0.321	0.014	0.002	6.5e-10
rs8052370	16	88837298	T	C	0.610	-0.023	0.002	3.9e-25
rs7214308	17	1965085	T	C	0.742	-0.015	0.002	3.6e-10
rs6502555	17	2729652	C	T	0.269	0.029	0.002	7.5e-34
rs62091998	17	2881625	G	A	0.308	-0.025	0.002	5.1e-27
rs34230287	17	4613630	T	C	0.239	0.031	0.002	2.5e-36

rs62073635	17	4770197	C	T	0.346	-0.027	0.002	4.3e-33
rs77234976	17	7141140	G	C	0.145	-0.024	0.003	5.3e-15
rs11652760	17	16786819	G	T	0.101	-0.025	0.004	2.1e-12
rs2297508	17	17715317	G	C	0.649	-0.023	0.002	8.7e-26
rs2453582	17	19439066	T	C	0.386	0.018	0.002	6.6e-17
rs3760456	17	27948844	T	C	0.441	-0.016	0.002	9.8e-14
rs2522972	17	35839021	T	C	0.639	-0.022	0.002	7.4e-23
rs4795397	17	38023745	G	A	0.482	-0.031	0.002	2.1e-48
rs3136685	17	38719799	T	C	0.161	-0.021	0.003	8.7e-13
rs72836561	17	41926126	T	C	0.031	-0.037	0.006	7.3e-10
rs1295927	17	57929535	G	A	0.447	-0.022	0.002	1.2e-25
rs9893518	17	62006997	C	T	0.258	-0.014	0.002	3.8e-09
rs16978167	17	72673667	T	A	0.764	0.024	0.003	2.4e-21
rs34891561	17	73027851	T	G	0.333	-0.015	0.002	4.4e-11
rs2665967	17	74016834	C	T	0.732	0.015	0.002	1.2e-09
rs4789299	17	74452010	A	G	0.203	0.030	0.003	2.6e-29
rs8096327	18	12887750	G	A	0.391	-0.013	0.002	2.1e-09
rs3851820	18	20461322	T	A	0.744	-0.016	0.002	1.2e-10
rs77551289	18	60788745	G	A	0.098	-0.028	0.004	9.2e-15
rs4940572	18	60826343	A	G	0.134	0.024	0.003	2.8e-14
rs111304587	18	77271669	G	T	0.085	0.029	0.004	2.5e-14
rs77232119	19	300952	G	T	0.273	0.015	0.002	1.9e-10
rs35140707	19	1078297	T	C	0.100	-0.059	0.004	1.8e-63
rs28841391	19	1193965	G	T	0.759	0.028	0.003	2.8e-27
rs35251378	19	10459969	A	G	0.293	0.044	0.002	2.8e-78
rs56252104	19	13123188	T	C	0.261	0.019	0.002	1.1e-15
rs62124251	19	13915213	T	G	0.348	0.032	0.002	2.6e-47
rs35024802	19	16388636	T	C	0.148	-0.023	0.003	1.9e-14
rs56133626	19	16441973	A	G	0.328	-0.070	0.002	1e-200
rs183685887	19	16598721	C	T	0.039	0.116	0.006	6.7e-97
rs4530278	19	33752994	T	G	0.601	0.019	0.002	4.3e-18
rs4805881	19	33896432	C	A	0.665	-0.015	0.002	3.3e-11
rs75018496	19	40785683	G	C	0.077	0.024	0.004	1.6e-09
rs61387190	19	44260929	T	C	0.157	0.030	0.003	1.1e-25
rs34159938	19	47690811	A	G	0.259	0.031	0.002	4.3e-38
rs11084096	19	52128795	A	G	0.296	-0.018	0.002	1.4e-14
rs2070745	19	52249947	G	C	0.365	-0.017	0.002	6.3e-14
rs78487206	19	55347738	T	G	0.165	-0.019	0.003	2.8e-10
rs4630833	20	1930798	C	T	0.263	-0.045	0.002	5.1e-79
rs1883932	20	8609588	T	A	0.508	-0.028	0.002	6.3e-40
rs34952318	20	11177055	A	G	0.049	-0.037	0.005	1.3e-13
rs1997797	20	31387954	G	C	0.447	0.018	0.002	7.6e-17
rs7273174	20	34350306	C	T	0.110	-0.020	0.003	2.9e-09
rs6072080	20	39260927	T	C	0.585	0.021	0.002	2e-21
rs4812803	20	42850827	T	C	0.285	0.015	0.002	2.9e-10
rs13045492	20	49134535	T	A	0.084	0.031	0.004	1.2e-15
rs34323943	20	52192637	C	T	0.111	0.023	0.003	2e-11
rs343829	20	57553504	C	G	0.929	-0.027	0.004	4.6e-11
rs259981	20	57748369	A	T	0.359	-0.028	0.002	9.2e-36
rs6064762	20	57867578	C	T	0.539	-0.013	0.002	2.9e-10
rs1997577	21	16371102	T	A	0.155	-0.018	0.003	1.5e-09
rs150797	21	43465831	G	C	0.454	-0.015	0.002	8.6e-12
rs1893592	21	43855067	C	A	0.300	0.016	0.002	2.7e-12
rs721131	21	47957767	C	T	0.320	-0.020	0.002	3.9e-19
rs1034564	22	19984013	T	C	0.286	0.022	0.002	3.5e-21
rs4821124	22	21979289	C	T	0.189	-0.043	0.003	6e-56

rs552292854	22	24654627	T	C	0.254	-0.017	0.003	6.4e-11
rs4583569	22	28933106	T	C	0.878	0.033	0.003	7.1e-24
rs6006356	22	30503171	A	G	0.446	0.035	0.002	1e-61
rs139425	22	39559742	C	G	0.451	-0.027	0.002	6.2e-36
rs202638	22	41855458	G	A	0.795	-0.027	0.003	2.5e-24
rs9607869	22	42418110	A	T	0.299	0.015	0.002	6.2e-11

Table S12. 456 SNPs significantly associated with lymphocyte count used IVs in forward MR analyses derived from Chen MH et al. Chr: Chromosome; EA: Effect allele; NEA: Non-effect allele; EAF: Effect allele frequency.

SNP	Chr	Pos	EA	NEA	EAF	Beta	SE	P-value
rs603424	10	102075479	A	G	0.175	0.020	0.002	2.78e-16
rs35896146	10	116024458	A	G	0.159	0.022	0.003	8.15e-17
rs7071131	10	121026712	G	A	0.495	-0.016	0.002	3.85e-16
rs10794175	10	126358073	T	G	0.416	-0.021	0.002	2.5e-26
rs35001362	10	14958758	A	C	0.236	-0.015	0.002	1.52e-11
rs10828725	10	25218243	T	G	0.366	-0.032	0.002	1.29e-59
rs2992263	10	26741207	A	G	0.610	-0.020	0.002	1.58e-25
rs2462017	10	30486133	A	G	0.652	0.014	0.002	2.87e-12
rs7093481	10	44879711	G	A	0.307	-0.017	0.002	6.84e-16
rs61850684	10	50252936	A	G	0.234	-0.032	0.002	2.59e-47
rs3118469	10	6101129	T	A	0.300	0.029	0.002	1.5e-45
rs4948492	10	63719739	T	C	0.662	-0.022	0.002	5.69e-28
rs224082	10	64566258	T	C	0.378	-0.016	0.002	1.79e-16
rs10762264	10	70976833	A	G	0.681	-0.020	0.002	1.06e-23
rs10823631	10	72625741	G	A	0.325	-0.017	0.002	3.11e-17
rs703257	10	73119135	T	C	0.888	-0.021	0.003	3.71e-12
rs748113	10	73508791	C	T	0.436	-0.034	0.002	1.53e-65
rs10748526	10	82273079	C	T	0.794	0.015	0.002	7.88e-11
rs11187162	10	94508636	A	G	0.162	0.020	0.003	2.61e-15
rs58667319	10	96524395	T	C	0.123	-0.023	0.003	9.91e-16
rs41317270	10	98369755	T	C	0.177	-0.018	0.002	9.82e-14
rs11189058	10	99001258	A	G	0.660	0.012	0.002	7.31e-10
rs11224302	11	100456604	T	C	0.099	-0.038	0.003	1.73e-33
rs17116384	11	113981241	G	A	0.317	0.013	0.002	4.5e-10
rs1893033	11	119089055	T	C	0.075	-0.028	0.004	5.7e-15
rs11218725	11	122509237	G	A	0.377	0.033	0.002	1.03e-63
rs79805393	11	128412297	C	T	0.173	0.017	0.003	1.04e-11
rs7127911	11	128492549	T	G	0.266	0.024	0.002	3.44e-29
rs2403246	11	18101078	G	C	0.414	0.012	0.002	4.81e-10
rs61867141	11	1874892	A	G	0.195	-0.017	0.003	1.37e-11
rs11033388	11	36074218	T	A	0.419	0.020	0.002	2.07e-24
rs74045276	11	403484	C	G	0.565	0.013	0.002	2.85e-11
rs10838634	11	46929954	G	A	0.902	-0.026	0.003	3e-16
rs1017875	11	47999218	T	C	0.202	-0.036	0.002	5.01e-53
rs74679312	11	62194434	G	A	0.057	0.031	0.004	2.28e-13
rs34662054	11	68908815	C	G	0.299	0.014	0.002	4.8e-12
rs949349	11	86297189	C	T	0.271	0.014	0.002	3.26e-11
rs768908	12	10035304	T	C	0.125	-0.020	0.003	2.4e-12
rs17041439	12	101873240	C	A	0.057	0.041	0.004	2.11e-23
rs4764803	12	101898230	G	C	0.617	0.014	0.002	4.87e-13
rs1265564	12	111708458	C	A	0.443	0.072	0.002	1e-200
rs73207626	12	112686873	G	A	0.042	-0.036	0.005	9.72e-14
rs1293782	12	113463594	A	C	0.644	-0.015	0.002	3.74e-14
rs28533432	12	123873242	T	C	0.700	-0.025	0.002	7.17e-33
rs67516712	12	124201524	A	G	0.423	0.015	0.002	9.11e-15
rs10846577	12	124400261	C	T	0.442	-0.014	0.002	2.37e-13
rs34327	12	12873748	C	T	0.523	-0.026	0.002	5.91e-42
rs6487543	12	26438189	A	G	0.771	0.020	0.002	6.2e-18
rs11611710	12	4340527	C	A	0.269	-0.013	0.002	2.45e-09
rs11168249	12	48208368	C	T	0.461	-0.019	0.002	6.04e-24
rs73108566	12	49080698	A	T	0.332	-0.017	0.002	1.23e-16

rs4761790	12	51749966	A	G	0.763	0.014	0.002	8.26e-10
rs706819	12	52315923	C	T	0.738	0.014	0.002	3.61e-10
rs1131017	12	56435929	G	C	0.573	0.014	0.002	9.76e-13
rs10466905	12	6502832	A	G	0.190	0.062	0.002	7.09e-140
rs1118866	12	68520751	T	C	0.704	0.015	0.002	1.1e-12
rs34038797	12	740009	G	C	0.481	-0.019	0.002	9.44e-23
rs7308380	12	89851232	C	A	0.751	0.016	0.002	2.47e-13
rs1371076	12	90561450	C	T	0.740	0.015	0.002	4.22e-12
rs374053567	13	114454143	G	A	0.042	0.031	0.005	2.05e-09
rs2001388	13	114843054	C	T	0.461	-0.014	0.002	1.55e-12
rs9590390	13	114920867	A	G	0.292	0.030	0.002	2.51e-46
rs76428106	13	28604007	C	T	0.013	0.063	0.009	5.49e-13
rs9532679	13	41522338	C	A	0.149	-0.032	0.003	6.33e-33
rs9525619	13	42992135	T	C	0.534	-0.020	0.002	1.08e-26
rs9534338	13	46707137	C	T	0.460	0.015	0.002	1.38e-15
rs9568054	13	49179386	A	C	0.323	0.014	0.002	1.41e-11
rs1523178	13	50652456	C	A	0.221	-0.026	0.002	3.86e-29
rs912416	13	50933553	C	A	0.445	-0.012	0.002	3.58e-10
rs67483792	13	72503638	T	C	0.043	-0.035	0.005	8.06e-14
rs9592965	13	74615925	C	A	0.327	-0.014	0.002	8.83e-13
rs3812849	13	74701736	C	A	0.266	0.043	0.002	1.32e-91
rs7157267	14	103350813	C	T	0.222	0.030	0.002	1.99e-40
rs4900575	14	103899169	C	G	0.366	0.018	0.002	2.03e-20
rs45528934	14	23793305	T	C	0.162	-0.021	0.003	1.85e-15
rs2057340	14	35848774	G	A	0.653	0.014	0.002	4.66e-12
rs696	14	35871093	T	C	0.365	0.025	0.002	4.94e-38
rs1966865	14	64975861	A	G	0.317	0.027	0.002	1.36e-40
rs10138752	14	69179971	T	C	0.077	-0.030	0.004	4.89e-17
rs175714	14	75981856	C	T	0.577	0.026	0.002	9.2e-42
rs2180369	14	93516465	C	T	0.111	0.020	0.003	1.47e-10
rs10145277	14	99793449	A	T	0.625	-0.015	0.002	3.19e-14
rs2412544	15	40949526	T	C	0.394	-0.016	0.002	1.13e-16
rs139974673	15	44027885	C	T	0.026	0.054	0.006	1.89e-19
rs12593807	15	48546727	C	T	0.136	-0.021	0.003	7.57e-14
rs7161799	15	58770523	T	C	0.076	0.034	0.004	2.79e-21
rs2439408	15	66925163	C	G	0.561	-0.012	0.002	9.72e-11
rs149453951	15	69583434	T	C	0.024	0.049	0.007	4.04e-13
rs34025077	15	70033578	G	A	0.101	-0.033	0.003	1.25e-25
rs631864	15	70376441	C	T	0.472	0.014	0.002	1.48e-12
rs17507693	15	83793622	T	A	0.223	0.016	0.002	9.86e-13
rs187856913	15	90555181	A	G	0.039	0.048	0.005	6.24e-22
rs11073903	15	90653194	A	G	0.567	-0.013	0.002	4.41e-11
rs28539372	15	91021412	A	T	0.319	0.038	0.002	3.38e-78
rs17229044	16	11062936	T	C	0.210	-0.019	0.002	1.78e-16
rs145719494	16	24044921	G	A	0.059	-0.031	0.004	3.71e-14
rs12598978	16	30482540	G	T	0.520	0.055	0.002	1.58e-181
rs28853644	16	30801027	T	C	0.271	-0.024	0.002	3.77e-30
rs78487935	16	3651579	G	A	0.078	-0.024	0.004	3.2e-11
rs447361	16	4148240	G	T	0.666	0.018	0.002	6.1e-19
rs7192652	16	57075180	G	A	0.481	0.022	0.002	4.32e-31
rs149012671	16	67596482	G	C	0.044	-0.030	0.005	1.89e-10
rs117556162	16	67680806	A	G	0.058	0.044	0.004	3.31e-27
rs9939124	16	75185764	T	C	0.307	-0.017	0.002	3.6e-16
rs247826	16	84582965	T	C	0.221	0.039	0.002	1.19e-63
rs56292801	16	88535341	A	G	0.273	0.014	0.002	1.02e-09
rs8052370	16	88837298	T	C	0.609	-0.021	0.002	5.82e-25

rs1677490	16	9028541	C	G	0.791	0.016	0.002	1.25e-11
rs11652968	17	16813349	T	C	0.190	-0.016	0.002	8.04e-11
rs2297508	17	17715317	G	C	0.648	-0.022	0.002	2.2e-27
rs2453582	17	19439066	T	C	0.391	0.018	0.002	4.88e-19
rs6502555	17	2729652	C	T	0.269	0.029	0.002	9.26e-38
rs3760456	17	27948844	T	C	0.439	-0.015	0.002	1.4e-14
rs62091998	17	2881625	G	A	0.307	-0.022	0.002	1.33e-27
rs2522972	17	35839021	T	C	0.640	-0.020	0.002	6.54e-25
rs4795397	17	38023745	G	A	0.479	-0.030	0.002	3.62e-57
rs2072081	17	42327493	T	G	0.255	-0.017	0.002	2.53e-14
rs35186877	17	4625892	A	G	0.214	0.032	0.002	1.94e-42
rs8075090	17	4969108	C	T	0.524	0.021	0.002	2.01e-28
rs9898958	17	55242242	G	T	0.153	0.016	0.003	3.56e-09
rs1292069	17	57928290	C	T	0.450	-0.023	0.002	3.09e-32
rs11657448	17	62023128	T	C	0.322	0.013	0.002	8.47e-10
rs1215	17	7163350	G	A	0.144	-0.023	0.003	3.67e-17
rs9906320	17	72690829	A	G	0.775	0.025	0.002	8.67e-27
rs1491765	17	73028923	A	G	0.293	-0.017	0.002	1.67e-16
rs2665960	17	74024711	G	A	0.669	0.012	0.002	4.03e-09
rs4789294	17	74419177	G	A	0.219	0.027	0.002	1.05e-30
rs116876036	17	76393762	T	C	0.090	0.021	0.003	9.57e-10
rs8096327	18	12887750	G	A	0.391	-0.013	0.002	1.59e-10
rs3851820	18	20461322	T	A	0.745	-0.018	0.002	7.37e-16
rs4987855	18	60793549	T	C	0.096	-0.024	0.003	1.16e-13
rs4940572	18	60826343	A	G	0.136	0.023	0.003	1.17e-16
rs9676181	18	74769175	A	T	0.574	0.012	0.002	9.73e-10
rs111626441	18	77250669	A	G	0.097	0.028	0.003	8.97e-17
rs36084354	19	1079959	A	G	0.092	-0.061	0.003	6.6e-71
rs3817621	19	12998205	C	G	0.238	-0.017	0.002	1.11e-13
rs345627	19	13917264	T	C	0.349	0.034	0.002	3.13e-60
rs897791	19	16490859	G	A	0.967	-0.096	0.005	3.3e-69
rs138243594	19	16495586	A	G	0.013	-0.076	0.009	4.57e-16
rs148213173	19	16647026	C	A	0.054	0.108	0.004	5.63e-146
rs4530278	19	33752994	T	G	0.598	0.017	0.002	5.24e-18
rs4805881	19	33896432	C	A	0.665	-0.015	0.002	1.41e-13
rs75018496	19	40785683	G	C	0.076	0.025	0.004	1.6e-11
rs60505738	19	4089811	T	C	0.090	0.026	0.004	1.2e-13
rs61387190	19	44260929	T	C	0.157	0.032	0.003	5.81e-34
rs56179616	19	47695751	T	G	0.259	0.031	0.002	6.22e-47
rs3810276	19	50934939	A	G	0.405	-0.012	0.002	1.47e-09
rs11084096	19	52128795	A	G	0.296	-0.018	0.002	6.42e-19
rs2070745	19	52249947	G	C	0.367	-0.016	0.002	3.94e-16
rs12088882	1	101224620	T	C	0.408	0.034	0.002	1.3e-70
rs10875371	1	101762529	T	C	0.418	-0.021	0.002	3.37e-27
rs77954449	1	10242292	G	A	0.028	-0.037	0.006	9.78e-11
rs2476601	1	114377568	G	A	0.900	0.054	0.003	1.47e-67
rs11204702	1	150663507	A	C	0.510	-0.026	0.002	3.37e-42
rs967072	1	156972485	T	C	0.137	-0.017	0.003	3.88e-10
rs12045893	1	158530416	T	C	0.248	0.027	0.002	9.7e-35
rs1633267	1	159010220	G	T	0.865	-0.018	0.003	1.46e-10
rs72634819	1	1596500	T	C	0.264	-0.014	0.002	4.67e-10
rs494091	1	160791892	C	T	0.426	0.027	0.002	6.35e-46
rs60220286	1	161639559	A	G	0.175	-0.021	0.003	8.03e-15
rs6427618	1	161680258	A	G	0.621	0.014	0.002	1.55e-12
rs2489279	1	182144119	G	C	0.334	0.018	0.002	1.28e-18
rs72740550	1	197342380	A	G	0.222	-0.019	0.002	1.58e-16

rs10494783	1	198663661	A	G	0.052	-0.054	0.004	8.95e-37
rs113116201	1	198830942	C	T	0.020	0.041	0.007	1.93e-09
rs2294630	1	19981791	G	A	0.493	0.013	0.002	8.15e-12
rs11240335	1	203714629	T	C	0.067	0.029	0.004	6.49e-13
rs4619033	1	204269242	G	A	0.268	-0.019	0.002	5.73e-19
rs1967115	1	212375359	A	G	0.629	0.017	0.002	1.04e-17
rs12745411	1	214405704	T	G	0.600	0.017	0.002	6.32e-18
rs12021809	1	225941782	A	G	0.110	-0.026	0.003	2.69e-18
rs533483	1	234765256	A	G	0.244	-0.014	0.002	1.58e-10
rs4660128	1	236087992	C	T	0.897	-0.025	0.003	3.12e-15
rs61778219	1	24235932	T	C	0.141	0.023	0.003	2.67e-17
rs4925547	1	247612435	T	A	0.402	0.012	0.002	4.55e-10
rs74227709	1	247722588	A	G	0.072	-0.029	0.004	2.51e-15
rs2282718	1	25241056	A	G	0.372	-0.018	0.002	3.25e-18
rs4274112	1	26746199	G	A	0.373	0.014	0.002	1.51e-12
rs11247712	1	28229681	T	C	0.369	-0.020	0.002	3.38e-24
rs12142033	1	39542129	A	G	0.333	-0.013	0.002	1.73e-10
rs59543294	1	51495673	C	T	0.107	0.019	0.003	3.46e-10
rs4319365	1	56907720	A	G	0.545	-0.019	0.002	3.26e-24
rs1933295	1	62107021	G	A	0.777	-0.014	0.002	1.22e-09
rs12127284	1	65431599	T	C	0.710	-0.017	0.002	1.11e-15
rs6700896	1	66089782	T	C	0.379	-0.017	0.002	3.67e-19
rs2755253	1	67470843	T	C	0.709	-0.031	0.002	2.87e-50
rs2275902	1	79357360	C	G	0.298	-0.016	0.002	5.22e-15
rs12131864	1	8591162	G	A	0.631	-0.019	0.002	3.34e-23
rs462579	1	91511604	T	A	0.109	-0.021	0.003	2.87e-12
rs34952318	20	11177055	A	G	0.050	-0.035	0.004	1.11e-14
rs4411786	20	1930897	C	T	0.265	-0.045	0.002	6.39e-98
rs1997797	20	31387954	G	C	0.446	0.017	0.002	4.44e-19
rs7273174	20	34350306	C	T	0.110	-0.022	0.003	1.17e-12
rs6072080	20	39260927	T	C	0.585	0.020	0.002	1.77e-24
rs6103270	20	42015635	G	A	0.226	-0.014	0.002	1.91e-09
rs968478	20	47339742	G	A	0.397	0.015	0.002	3.11e-14
rs2230604	20	49196284	T	C	0.084	0.030	0.003	3.65e-18
rs6021231	20	50092287	C	T	0.471	-0.011	0.002	2.21e-09
rs6063965	20	52191200	A	G	0.114	0.023	0.003	3.46e-14
rs259981	20	57748369	A	T	0.360	-0.027	0.002	3.91e-42
rs2738783	20	62308612	G	T	0.790	-0.016	0.002	1.24e-10
rs1297265	21	16817051	G	A	0.443	0.012	0.002	7.44e-10
rs150797	21	43465831	G	C	0.447	-0.014	0.002	2.19e-12
rs1893592	21	43855067	C	A	0.296	0.015	0.002	2.13e-12
rs721131	21	47957767	C	T	0.320	-0.019	0.002	1.36e-20
rs9605047	22	20002848	T	G	0.328	0.020	0.002	2.99e-21
rs5998509	22	21917479	T	C	0.188	-0.041	0.002	2.19e-61
rs71318973	22	24658265	T	G	0.241	-0.015	0.002	7.29e-12
rs16986308	22	28717032	A	G	0.116	-0.032	0.003	9.95e-28
rs714027	22	30577771	G	A	0.552	-0.035	0.002	5.96e-75
rs139425	22	39559742	C	G	0.447	-0.027	0.002	1.85e-45
rs2899319	22	39874314	C	A	0.619	0.012	0.002	3.13e-09
rs5758452	22	42144927	A	G	0.793	-0.027	0.002	9.92e-31
rs62241216	22	50744821	G	A	0.492	-0.012	0.002	3.88e-10
rs6755786	2	103048103	T	C	0.773	0.027	0.002	4.2e-32
rs1533298	2	111616304	T	C	0.715	0.031	0.002	1.19e-49
rs1976055	2	111787853	A	C	0.156	-0.017	0.003	4.05e-11
rs144942718	2	112238447	A	G	0.075	-0.072	0.004	3.47e-73
rs17366643	2	11344504	C	T	0.538	0.015	0.002	1.35e-14

rs4849169	2	113953657	C	A	0.482	0.021	0.002	4.14e-28
rs35002856	2	12878229	C	G	0.373	0.012	0.002	4.03e-10
rs71420836	2	129060871	T	C	0.097	0.030	0.003	1.67e-19
rs62160910	2	136919140	C	G	0.058	-0.024	0.004	4.53e-09
rs73961715	2	143803847	C	T	0.128	-0.049	0.003	2.79e-67
rs148601577	2	143847602	G	A	0.020	-0.049	0.007	3.79e-12
rs34030812	2	144248905	C	T	0.367	-0.018	0.002	1.49e-20
rs10173412	2	161330940	C	T	0.201	-0.039	0.002	1.45e-62
rs10180049	2	162993472	A	G	0.385	0.015	0.002	5.21e-14
rs13432797	2	165526587	C	G	0.345	-0.012	0.002	2.12e-09
rs529002	2	169703896	G	T	0.551	-0.011	0.002	4.46e-09
rs11684463	2	182077535	T	A	0.327	-0.047	0.002	1.3e-121
rs62180255	2	182188444	A	T	0.441	0.034	0.002	1.81e-72
rs10179705	2	188315234	A	G	0.294	-0.021	0.002	1.29e-24
rs6743068	2	202153920	G	A	0.730	0.031	0.002	1.69e-49
rs715	2	211543055	C	T	0.311	-0.015	0.002	5.63e-13
rs11897252	2	216250263	T	C	0.234	-0.014	0.002	4.69e-10
rs78058190	2	219699999	A	G	0.052	0.047	0.005	1.36e-22
rs978917	2	223974443	A	G	0.615	0.018	0.002	7.38e-20
rs11676298	2	227291731	G	C	0.191	0.027	0.002	2.86e-29
rs34261309	2	231224638	C	T	0.107	0.022	0.003	8.5e-13
rs58106596	2	232579379	A	G	0.226	-0.024	0.002	3.67e-25
rs13016839	2	234357513	G	A	0.107	-0.026	0.003	1.03e-16
rs55982282	2	24188128	A	G	0.135	0.062	0.003	8.7e-110
rs1260326	2	27730940	C	T	0.602	-0.024	0.002	1.44e-35
rs11688289	2	40698811	C	T	0.189	-0.015	0.002	6.98e-10
rs149290349	2	43451957	A	G	0.075	-0.068	0.004	3.29e-77
rs2542573	2	54046646	C	T	0.331	-0.015	0.002	1.52e-13
rs4671238	2	55324905	T	C	0.560	-0.014	0.002	1.05e-12
rs12991188	2	62554797	G	A	0.598	0.013	0.002	3.77e-11
rs6546881	2	74187420	T	C	0.657	0.013	0.002	2.17e-10
rs6721663	2	7615061	A	G	0.159	-0.026	0.003	9.83e-24
rs7572278	2	8563029	A	T	0.207	0.020	0.002	1.04e-17
rs2100139	3	108504265	C	T	0.658	0.015	0.002	2.32e-14
rs1822534	3	12266804	G	A	0.394	-0.034	0.002	7.22e-71
rs62262391	3	123105119	T	C	0.225	-0.026	0.002	3.52e-30
rs332507	3	124435110	C	T	0.222	-0.015	0.002	3.12e-11
rs11712335	3	128210550	C	T	0.269	0.015	0.002	1.26e-12
rs9835571	3	136523475	G	T	0.690	0.022	0.002	3.49e-27
rs12636926	3	141660742	C	T	0.730	-0.014	0.002	1.92e-10
rs721377	3	14435063	T	C	0.102	0.020	0.003	5.23e-10
rs9864216	3	151007262	A	G	0.776	0.024	0.002	1.57e-25
rs76830965	3	159637678	A	C	0.117	-0.021	0.003	2.13e-12
rs2291900	3	170825920	A	G	0.291	0.017	0.002	1.49e-15
rs7627511	3	171178037	C	G	0.219	-0.017	0.002	2.11e-12
rs2270968	3	182755209	G	T	0.738	-0.013	0.002	2.46e-09
rs11926572	3	183742328	G	A	0.375	0.013	0.002	7.34e-11
rs77265382	3	185546150	T	C	0.045	-0.033	0.005	1.17e-12
rs11505019	3	188094327	G	T	0.411	-0.013	0.002	3.18e-12
rs6808949	3	18883999	C	T	0.111	0.023	0.003	9.16e-14
rs56850780	3	195926731	T	G	0.078	-0.021	0.004	3.55e-09
rs2089979	3	196501413	G	A	0.414	-0.035	0.002	4.1e-72
rs2887944	3	27758275	T	G	0.436	0.019	0.002	1.13e-28
rs74280151	3	28287735	T	C	0.175	0.029	0.002	2.22e-38
rs11706384	3	39296881	T	G	0.236	0.027	0.002	7.44e-34
rs73081851	3	56623617	T	A	0.530	0.016	0.002	2.48e-17

rs12485738	3	56865776	G	A	0.628	0.018	0.002	5.99e-20
rs35592432	3	71355240	C	G	0.027	0.109	0.006	1.68e-68
rs6809697	3	71414864	A	G	0.136	-0.018	0.003	7.75e-11
rs5026470	4	103554722	G	A	0.517	0.037	0.002	1.45e-84
rs2237040	4	110838862	T	C	0.406	-0.020	0.002	3.53e-24
rs7672879	4	122811172	A	T	0.353	0.013	0.002	1.08e-11
rs309375	4	123681156	A	C	0.575	0.019	0.002	7.84e-23
rs7696969	4	143326714	G	T	0.620	-0.012	0.002	7.36e-10
rs4696314	4	153005716	C	T	0.515	0.014	0.002	9.33e-14
rs74408817	4	157801752	A	C	0.081	-0.023	0.003	2.1e-11
rs62334097	4	169301245	G	A	0.113	-0.019	0.003	3.38e-10
rs1344603	4	38109847	C	T	0.676	0.029	0.002	1.19e-47
rs6531629	4	38358752	C	T	0.703	0.064	0.002	1e-200
rs17581460	4	38456404	C	T	0.020	-0.040	0.007	3.76e-09
rs13105987	4	38602467	A	G	0.274	0.046	0.002	3.64e-103
rs73142294	4	40301624	T	C	0.248	0.015	0.002	7.51e-12
rs6554158	4	55066077	A	G	0.231	0.014	0.002	2.34e-10
rs58408429	4	57769824	C	T	0.186	-0.024	0.002	2.42e-22
rs12640189	4	6893181	T	C	0.176	0.016	0.003	3.4e-10
rs2298850	4	72614267	C	G	0.278	-0.014	0.002	1.67e-11
rs10027415	4	80898027	T	C	0.529	-0.016	0.001	6.33e-33
rs17005891	4	83547862	A	G	0.184	-0.024	0.002	4.43e-43
rs1431545	4	90220659	T	C	0.653	0.021	0.002	5.05e-25
rs2548256	5	100148462	T	G	0.620	0.014	0.002	6.49e-13
rs78270096	5	110517419	A	G	0.070	-0.034	0.004	7.79e-19
rs3797727	5	111079024	A	T	0.112	0.020	0.003	4.94e-11
rs73239280	5	118695579	A	G	0.203	-0.032	0.002	4.72e-41
rs58265751	5	124377841	C	T	0.521	-0.012	0.002	1.61e-10
rs251391	5	127538116	T	C	0.747	0.020	0.002	1.21e-19
rs10076701	5	131596419	C	T	0.557	-0.023	0.002	5.52e-33
rs75925066	5	134722833	A	C	0.023	0.085	0.006	1.84e-40
rs1990933	5	147935890	G	A	0.475	-0.012	0.002	4.86e-10
rs3815725	5	150518532	G	A	0.520	-0.017	0.002	3.66e-18
rs11134475	5	156399950	G	A	0.636	-0.014	0.002	4.42e-13
rs17055818	5	157969475	C	T	0.252	-0.030	0.002	2.15e-42
rs55977949	5	158375669	A	G	0.098	-0.030	0.003	1.15e-21
rs360017	5	173207353	G	A	0.775	-0.021	0.002	1.69e-20
rs4976642	5	176673068	A	T	0.831	0.024	0.003	3.44e-22
rs11742873	5	179244302	T	C	0.837	-0.017	0.003	1.75e-10
rs464609	5	34654477	A	G	0.544	0.012	0.002	5.3e-10
rs11567701	5	35859863	T	G	0.262	0.054	0.002	2.4e-141
rs2548496	5	52073844	G	A	0.208	0.019	0.002	3.34e-16
rs2910580	5	57527886	A	T	0.659	-0.014	0.002	3.57e-12
rs251398	5	67509801	C	T	0.447	-0.017	0.002	2.39e-19
rs678393	5	72418309	G	T	0.262	-0.015	0.002	2.32e-12
rs160044	5	88114476	C	T	0.308	0.012	0.002	4.72e-09
rs504117	6	10518350	A	G	0.522	-0.013	0.002	1.34e-11
rs12526696	6	108053364	A	G	0.161	0.037	0.003	6.06e-44
rs6926219	6	122720806	A	G	0.547	0.027	0.002	2.23e-45
rs1415701	6	130345835	A	G	0.267	0.014	0.002	9.71e-11
rs72986491	6	135042596	A	G	0.206	0.017	0.002	1.28e-12
rs7776054	6	135418916	G	A	0.261	-0.040	0.002	9.81e-78
rs17710008	6	153043035	A	G	0.182	0.021	0.002	4.45e-17
rs707827	6	15327500	A	G	0.629	0.015	0.002	9.97e-15
rs41267765	6	159462424	T	C	0.022	-0.046	0.006	1.24e-12
rs2273215	6	170586082	A	G	0.458	-0.015	0.002	5.04e-14

rs73735249	6	21809342	A	G	0.208	0.018	0.002	2.74e-14
rs72835315	6	231878	C	T	0.060	0.035	0.004	7.09e-17
rs2106072	6	30153363	A	G	0.127	-0.087	0.003	1e-200
rs28360986	6	30994248	G	C	0.095	0.051	0.003	3.1e-55
rs9461975	6	34236459	T	C	0.113	-0.031	0.003	4.24e-24
rs1050979	6	410417	G	A	0.525	0.025	0.002	3.97e-38
rs4711790	6	44573822	T	C	0.340	-0.017	0.002	4.88e-18
rs12190136	6	44767072	A	G	0.526	0.012	0.002	3.99e-10
rs2294861	6	53136516	T	C	0.294	0.019	0.002	3.84e-20
rs9328393	6	6902939	A	C	0.668	0.023	0.002	5.74e-31
rs607465	6	7129563	G	A	0.066	0.034	0.004	4.83e-19
rs1334577	6	7211751	A	G	0.235	-0.017	0.002	5.29e-14
rs72928038	6	90976768	A	G	0.177	-0.033	0.002	4.24e-41
rs78976959	7	100311666	A	G	0.041	0.033	0.005	8.41e-12
rs4730737	7	116076443	A	C	0.920	0.023	0.004	9.41e-11
rs9920	7	116200092	C	T	0.105	0.029	0.003	2.15e-21
rs62621812	7	127015083	A	G	0.021	0.056	0.007	2.73e-16
rs2727487	7	129513820	C	A	0.629	-0.013	0.002	1.08e-10
rs4336553	7	130743720	G	A	0.793	-0.022	0.002	1.61e-21
rs10269987	7	138741642	A	G	0.575	-0.017	0.002	4.83e-19
rs35114152	7	150294476	G	A	0.226	0.025	0.002	1.73e-27
rs73165514	7	150527651	A	G	0.086	0.021	0.003	5.47e-10
rs35345753	7	22740513	G	C	0.207	-0.027	0.002	6.95e-30
rs35121828	7	23634985	A	G	0.259	0.013	0.002	1.22e-09
rs10807805	7	2753850	G	A	0.584	-0.012	0.002	4.01e-10
rs9648346	7	28160113	G	C	0.203	-0.024	0.002	2.63e-25
rs6955702	7	3157702	G	A	0.522	-0.022	0.002	3.36e-31
rs2710804	7	36084529	C	T	0.376	0.014	0.002	1.63e-12
rs6966893	7	37385357	A	G	0.179	0.019	0.002	3.91e-14
rs3735485	7	45009341	G	A	0.845	0.044	0.003	3.56e-63
rs10245472	7	55147478	A	G	0.177	-0.016	0.002	1.65e-10
rs2908425	7	5569327	A	G	0.332	0.014	0.002	3.78e-12
rs10237524	7	56036024	A	G	0.330	-0.019	0.002	1.35e-21
rs2347784	7	6524843	G	C	0.268	0.028	0.002	6.95e-39
rs4718976	7	70113409	C	T	0.695	-0.018	0.002	1.1e-17
rs799166	7	73051932	G	C	0.133	-0.017	0.003	7.57e-10
rs10230506	7	92471518	A	T	0.161	0.021	0.003	2.86e-16
rs11984234	7	949067	A	T	0.214	0.017	0.002	2.12e-12
rs57098456	7	98038351	G	A	0.322	-0.013	0.002	9.15e-10
rs881492	8	101484638	G	C	0.467	-0.019	0.002	6.56e-23
rs1264593	8	102471269	T	G	0.495	0.015	0.002	2.15e-14
rs28588745	8	10647044	T	A	0.206	0.022	0.002	1.25e-21
rs13276352	8	11657838	C	T	0.209	0.014	0.002	2.15e-09
rs2055101	8	119886923	T	C	0.527	-0.013	0.002	1.84e-12
rs2980888	8	126507308	C	T	0.697	-0.015	0.002	1.21e-13
rs10956401	8	129002419	A	G	0.345	-0.025	0.002	5.05e-37
rs10107362	8	130986984	G	C	0.361	0.013	0.002	8.36e-11
rs7007986	8	142217115	A	G	0.601	-0.022	0.002	8.5e-28
rs7830045	8	18700282	C	A	0.436	-0.013	0.002	1.69e-11
rs56094005	8	21769432	G	A	0.043	-0.040	0.005	1.61e-17
rs1533065	8	22785768	T	C	0.345	0.017	0.002	4.95e-17
rs113142693	8	30242076	C	T	0.247	-0.017	0.002	1.46e-14
rs2980821	8	40028306	A	C	0.425	-0.013	0.002	4.97e-12
rs6474359	8	41549194	C	T	0.038	-0.051	0.005	1.13e-24
rs4737010	8	41630447	A	G	0.229	0.027	0.002	2.47e-32
rs10503147	8	421777	T	C	0.089	0.021	0.003	7.48e-10

rs7839516	8	61386533	C	T	0.369	0.015	0.002	3.08e-15
rs10109370	8	72909349	A	C	0.238	-0.019	0.002	2.26e-17
rs117934175	8	79069520	A	G	0.041	-0.045	0.005	1.12e-19
rs1441850	8	79657666	C	T	0.252	0.050	0.002	9.8e-117
rs7855586	9	112678121	G	A	0.290	0.015	0.002	1.03e-12
rs1002607	9	113858933	C	T	0.593	-0.013	0.002	1.93e-11
rs3761846	9	123689597	T	C	0.570	0.021	0.002	6.26e-27
rs10985915	9	126018715	G	A	0.111	-0.023	0.003	6.9e-14
rs4880192	9	139927062	G	A	0.721	0.029	0.002	1.16e-40
rs12336678	9	21785894	T	C	0.099	-0.022	0.003	6.82e-12
rs3731211	9	21986847	A	T	0.720	0.044	0.002	5.14e-95
rs2065500	9	22145694	G	A	0.160	-0.048	0.003	3.29e-77
rs1971429	9	33776182	A	C	0.729	0.013	0.002	2.01e-09
rs10814138	9	34723035	A	C	0.314	-0.014	0.002	2.55e-11
rs11541908	9	35705759	A	G	0.277	0.020	0.002	4.93e-22
rs10973700	9	38196117	C	G	0.487	-0.019	0.002	1.11e-23
rs501461	9	4039727	T	G	0.600	-0.018	0.002	6.76e-20
rs2807303	9	82187095	A	G	0.346	0.022	0.002	2.79e-27
rs62565782	9	86600887	A	G	0.131	-0.022	0.003	3.75e-14
rs61750929	9	91495135	T	C	0.056	-0.070	0.004	1.36e-65
rs3739756	9	98775235	C	T	0.036	-0.032	0.005	2.88e-10
rs74911261	11	108357137	G	A	0.974	-0.045	0.006	8.68e-14
rs4407213	2	25541636	G	C	0.968	-0.042	0.006	1.49e-14
rs140763487	3	112686528	C	T	0.986	0.049	0.008	9.26e-10
rs146946728	6	32490363	A	C	0.935	-0.039	0.006	5.08e-10
rs149984502	7	50539036	C	T	0.981	-0.048	0.007	1.18e-11
rs75653581	8	78363128	C	T	0.987	-0.061	0.009	5.45e-12
rs3824667	10	8100125	G	A	0.827	0.027	0.003	1.56e-24
rs28416859	11	196606	C	A	0.376	-0.019	0.002	3.44e-15
rs7933244	11	60267695	C	T	0.525	0.017	0.002	7.19e-19
rs7113428	11	65770230	A	G	0.340	0.013	0.002	5.89e-11
rs373582220	12	112847812	A	G	0.372	-0.035	0.003	4.06e-40
rs9513573	13	99815898	A	G	0.279	-0.015	0.002	2.73e-12
rs11863592	16	31301011	G	A	0.269	-0.016	0.002	2.21e-13
rs2667640	16	78575335	T	C	0.674	0.014	0.002	7.39e-12
rs77017480	17	2064712	A	T	0.100	-0.039	0.003	1.88e-32
rs578102530	17	44364335	A	G	0.187	-0.016	0.003	5.33e-10
rs28841391	19	1193965	G	T	0.758	0.029	0.003	3.03e-31
rs8101897	19	16410422	G	A	0.419	-0.062	0.002	1e-200
rs77232119	19	300952	G	T	0.273	0.016	0.002	1.22e-11
rs12741113	1	101147149	C	T	0.449	-0.018	0.002	2.26e-19
rs377345022	1	101653213	T	A	0.265	-0.018	0.002	2.97e-15
rs327200	1	120550701	G	C	0.106	0.021	0.003	3.83e-11
rs60918921	1	23850590	A	C	0.619	-0.015	0.002	1.39e-12
rs4068540	1	40398941	G	A	0.266	0.017	0.002	1.52e-14
rs1883932	20	8609588	T	A	0.509	-0.027	0.002	1.91e-44
rs5995837	22	40603363	C	G	0.270	0.015	0.002	5.15e-12
rs199898421	2	112193161	G	A	0.060	0.038	0.006	1.59e-10
rs4673265	2	204631738	T	C	0.731	-0.016	0.002	4.15e-13
rs7625643	3	141150026	G	A	0.444	0.025	0.002	2.36e-36
rs13063578	3	47087837	A	T	0.401	0.053	0.002	1.24e-142
rs62332762	4	106143492	T	C	0.396	-0.026	0.002	1.17e-39
rs62638606	5	124234974	A	T	0.504	-0.012	0.002	3.74e-09
rs373718455	5	16634559	A	G	0.249	-0.017	0.002	1.44e-13
rs2408014	5	54864017	T	A	0.681	-0.015	0.002	3.58e-13
rs61076041	6	109741530	G	C	0.298	-0.015	0.002	1.52e-11

rs7763994	6	151912354	T	A	0.291	0.014	0.002	6.72e-11
rs565245327	6	31614543	T	G	0.471	-0.050	0.003	5.89e-84
rs111959275	6	32618796	T	G	0.142	0.085	0.003	2.82e-168
rs55633929	7	156595998	A	G	0.425	-0.014	0.002	8.67e-12
rs192364644	7	77163413	C	G	0.612	-0.013	0.002	3.49e-11
rs200984644	7	98931658	G	A	0.064	0.040	0.004	3.73e-23
rs1976451	8	49456791	G	T	0.135	-0.017	0.003	4.35e-09
rs13277751	8	59415337	G	T	0.642	-0.012	0.002	1.14e-09
rs2791733	9	133623966	A	C	0.400	-0.015	0.002	4.38e-14
rs77866737	10	6075285	D	I	0.200	-0.029	0.003	3.65e-29
rs538811162	2	24613039	I	D	0.580	-0.017	0.002	9.31e-15
rs9494100	6	135195857	I	D	0.253	-0.018	0.002	8.97e-14
rs74835819	6	32455787	A	G	0.071	0.046	0.005	2.74e-20

Table S13. 464 SNPs significantly associated with myeloid white cell count used IVs in forward MR analyses derived from Chen MH et al. Chr: Chromosome; EA: Effect allele; NEA: Non-effect allele; EAF: Effect allele frequency.

SNP	Chr	Pos	EA	NEA	EAF	Beta	SE	P-value
rs7917772	10	104487443	A	G	0.631	0.017	0.002	3.6e-18
rs72836628	10	113902454	T	C	0.249	-0.014	0.002	3.02e-11
rs180941	10	115720674	A	G	0.625	-0.017	0.002	1.63e-18
rs12784071	10	115854445	T	C	0.222	0.018	0.002	4.79e-16
rs7920891	10	120895131	A	C	0.569	-0.012	0.002	1.35e-10
rs3781454	10	126348565	A	G	0.677	0.025	0.002	3.68e-36
rs10906393	10	13536512	T	A	0.576	0.015	0.002	2.44e-16
rs692594	10	18265893	C	G	0.489	0.013	0.002	1.7e-12
rs10828725	10	25218243	T	G	0.365	-0.050	0.002	1.1e-148
rs703005	10	26758576	T	C	0.598	-0.015	0.002	4.55e-16
rs2993986	10	28800023	T	C	0.774	0.030	0.002	4.75e-43
rs2091084	10	30492267	C	T	0.653	0.017	0.002	6.25e-19
rs11252331	10	4129296	G	T	0.837	0.017	0.002	1.8e-11
rs72790862	10	44880260	C	T	0.307	-0.026	0.002	5.05e-38
rs17011726	10	50264204	G	C	0.233	-0.024	0.002	3.58e-27
rs41295055	10	6111622	T	C	0.187	-0.014	0.002	4.95e-09
rs7082470	10	65277026	A	G	0.474	-0.029	0.002	5.75e-56
rs1885474	10	69566751	G	T	0.103	-0.022	0.003	1.11e-12
rs3747869	10	73520632	C	A	0.900	0.039	0.003	1.79e-33
rs11002309	10	79594931	T	C	0.340	0.013	0.002	8.28e-11
rs1880390	10	88334654	A	C	0.292	-0.012	0.002	1.18e-09
rs59085061	10	89681458	G	A	0.040	-0.050	0.005	9e-27
rs9633675	10	96210400	C	G	0.479	-0.019	0.002	9.6e-25
rs61863767	10	99084426	T	C	0.596	0.043	0.002	4.56e-117
rs12363256	11	100520680	A	T	0.325	0.016	0.002	4.54e-16
rs11225017	11	101687730	T	C	0.084	0.020	0.003	1.72e-09
rs4909932	11	10475967	G	A	0.598	0.014	0.002	2.01e-13
rs7934719	11	108341864	T	C	0.414	0.018	0.002	1.55e-21
rs73000965	11	113982321	A	T	0.315	0.029	0.002	1.86e-47
rs672058	11	116764021	T	C	0.880	0.018	0.003	1.81e-10
rs148713124	11	118225200	T	G	0.009	0.096	0.010	1.35e-21
rs11022177	11	12133478	G	C	0.311	-0.018	0.002	3.43e-19
rs8705	11	128328913	A	G	0.317	-0.026	0.002	1.35e-39
rs10831912	11	12856414	C	T	0.604	-0.011	0.002	1.69e-09
rs12792460	11	18076439	G	C	0.271	0.016	0.002	4.13e-14
rs1468102	11	3004526	G	C	0.306	0.015	0.002	9.95e-14
rs1232050	11	30798288	C	G	0.430	-0.012	0.002	2.48e-09
rs934177	11	44612714	G	C	0.436	-0.011	0.002	1.48e-09
rs1228024	11	47951353	A	C	0.661	0.025	0.002	2.32e-38
rs579721	11	59834318	A	G	0.284	-0.012	0.002	1.55e-09
rs174548	11	61571348	G	C	0.314	-0.025	0.002	6.38e-36
rs617791	11	65702523	C	G	0.483	0.019	0.002	1.59e-24
rs7177	11	69466115	A	C	0.529	-0.013	0.002	1.43e-12
rs17041439	12	101873240	C	A	0.057	0.038	0.004	2.96e-21
rs73190675	12	109788891	A	G	0.050	0.029	0.004	1.15e-11
rs10774624	12	111833788	A	G	0.514	-0.065	0.002	1e-200
rs11064881	12	120146925	A	G	0.074	-0.032	0.004	9.92e-20
rs7308123	12	122225225	C	T	0.181	0.019	0.002	2.29e-14
rs9697459	12	123988435	T	C	0.225	0.014	0.002	4e-10
rs9863	12	124421453	C	T	0.329	-0.019	0.002	1.53e-21
rs2024385	12	12888438	A	T	0.427	0.017	0.002	1.2e-19

rs11048425	12	26339986	G	C	0.491	0.013	0.002	1.03e-12
rs1007938	12	26802549	G	A	0.405	-0.011	0.002	3.26e-09
rs12580347	12	3388932	C	T	0.583	-0.012	0.002	3.28e-10
rs7955734	12	4333159	G	C	0.210	-0.029	0.002	2.1e-37
rs73108566	12	49080698	A	T	0.331	-0.013	0.002	1.32e-10
rs2302900	12	50599709	C	T	0.341	0.019	0.002	3.69e-22
rs1700159	12	52305786	T	C	0.774	0.024	0.002	6.3e-28
rs1245035	12	64976049	A	C	0.627	0.014	0.002	4.79e-14
rs2286599	12	6499533	A	G	0.141	0.039	0.003	1.43e-43
rs7968902	12	66363070	G	T	0.571	0.011	0.002	1.65e-09
rs759488	12	68587155	C	T	0.705	0.014	0.002	2.58e-11
rs4761234	12	69732105	C	T	0.482	0.015	0.002	2.03e-16
rs11114149	12	79908743	T	A	0.683	-0.014	0.002	7.3e-12
rs11104881	12	88843474	C	T	0.702	-0.025	0.002	1.59e-34
rs2265146	13	114178222	A	T	0.281	0.022	0.002	4.66e-28
rs3118899	13	114919294	A	G	0.357	0.014	0.002	2.6e-13
rs76428106	13	28604007	C	T	0.013	0.158	0.009	3.45e-77
rs138028125	13	28712689	G	C	0.034	0.054	0.006	2.54e-22
rs9508005	13	28789794	G	T	0.094	-0.023	0.003	4.92e-11
rs7326825	13	50113450	A	G	0.704	0.021	0.002	1.73e-24
rs806316	13	50838230	G	C	0.532	0.016	0.002	4.61e-18
rs9526795	13	52341181	C	T	0.259	-0.014	0.002	2.68e-11
rs3812849	13	74701736	C	A	0.267	0.020	0.002	5.85e-21
rs2439963	13	78638416	G	A	0.737	-0.012	0.002	3.29e-09
rs12429714	13	99649831	T	C	0.424	-0.011	0.002	2.45e-09
rs10146962	14	101170540	C	T	0.338	-0.015	0.002	6.45e-15
rs12898000	14	103835128	C	G	0.701	0.019	0.002	7.85e-20
rs2494748	14	105258892	T	C	0.616	0.013	0.002	6.09e-11
rs7159281	14	23756604	G	A	0.672	-0.013	0.002	1.35e-10
rs2038700	14	25461989	C	T	0.395	0.034	0.002	4.61e-74
rs72664840	14	35596323	T	C	0.179	0.021	0.002	8.44e-18
rs696	14	35871093	T	C	0.366	0.015	0.002	4.53e-15
rs9323285	14	55863829	A	C	0.338	0.012	0.002	8.19e-10
rs10138752	14	69179971	T	C	0.077	-0.040	0.003	1.85e-31
rs4903580	14	77850978	T	C	0.456	0.016	0.002	2.93e-18
rs2241621	14	81737076	C	A	0.588	-0.015	0.002	7.34e-16
rs10498635	14	93103309	T	C	0.183	-0.031	0.002	6.58e-39
rs2180369	14	93516465	C	T	0.111	0.023	0.003	7.65e-14
rs28576226	15	101713668	A	G	0.122	0.031	0.003	6.14e-28
rs4924450	15	40597229	A	G	0.706	0.014	0.002	1.14e-10
rs72726027	15	42248826	C	T	0.112	-0.043	0.003	4.17e-48
rs60695341	15	51010271	T	C	0.197	-0.030	0.002	3.2e-38
rs780142	15	62797964	G	T	0.274	0.015	0.002	2.02e-12
rs2062250	15	64672002	A	G	0.938	0.051	0.004	2.12e-38
rs2469147	15	66910224	G	A	0.158	-0.015	0.003	1.67e-09
rs62007171	15	77306285	C	T	0.291	-0.015	0.002	6.22e-14
rs11854390	15	80224817	T	C	0.562	0.014	0.002	2.13e-13
rs7183988	15	91428589	G	T	0.527	-0.016	0.002	8.74e-17
rs4984768	16	1253907	T	G	0.363	0.012	0.002	3.34e-09
rs9926183	16	1363878	T	C	0.263	0.019	0.002	2.86e-17
rs12929950	16	2144894	A	G	0.077	-0.030	0.004	7.83e-17
rs61739285	16	27480797	T	C	0.033	-0.034	0.005	9.98e-11
rs11574938	16	30485393	C	G	0.521	0.038	0.002	8.13e-91
rs28853644	16	30801027	T	C	0.272	-0.018	0.002	1.64e-17
rs9925985	16	31305593	C	A	0.269	-0.014	0.002	1.09e-11
rs6500550	16	3746241	T	C	0.304	-0.021	0.002	2.65e-24

rs75859969	16	50160737	C	A	0.156	-0.017	0.003	5.94e-11
rs11644125	16	57058974	T	C	0.597	-0.018	0.002	6.36e-21
rs12918121	16	67187795	T	C	0.018	-0.051	0.007	1.08e-12
rs117556162	16	67680806	A	G	0.057	0.027	0.004	1.99e-11
rs7198940	16	81605559	T	C	0.522	0.016	0.002	3.33e-17
rs247826	16	84582965	T	C	0.221	0.034	0.002	9.59e-52
rs9933582	16	86016026	G	T	0.230	0.021	0.002	9.18e-22
rs17232826	16	89842784	T	C	0.102	-0.022	0.003	1.6e-12
rs6502721	17	1362503	T	C	0.678	0.013	0.002	1.28e-10
rs12936529	17	16168784	T	C	0.479	-0.025	0.002	2.74e-42
rs146207734	17	16472850	T	A	0.017	-0.054	0.008	4.58e-11
rs4925108	17	17649423	T	C	0.615	-0.017	0.002	2.67e-18
rs7225843	17	2001825	C	T	0.203	-0.032	0.002	8.58e-43
rs756818	17	2711248	A	G	0.172	0.017	0.002	2.01e-12
rs16964963	17	27603316	G	A	0.173	0.022	0.002	2.54e-19
rs9900730	17	28179066	G	A	0.504	0.023	0.002	1.57e-35
rs1034686	17	35899141	A	T	0.584	-0.013	0.002	4.87e-13
rs12946510	17	37912377	T	C	0.472	-0.059	0.002	1e-200
rs74725931	17	38196327	C	T	0.037	0.061	0.005	3.65e-33
rs111975659	17	38570168	T	C	0.027	-0.036	0.006	1.34e-09
rs34855406	17	40731411	C	G	0.275	0.016	0.002	5.42e-15
rs35592645	17	4616387	A	C	0.249	0.017	0.002	2.38e-16
rs4790752	17	5115877	T	C	0.451	-0.018	0.002	2.11e-21
rs56378716	17	56356502	G	A	0.013	0.108	0.008	1.7e-38
rs2665405	17	57875292	A	G	0.548	0.035	0.002	5.63e-82
rs2084312	17	72695211	T	C	0.800	0.029	0.002	7.67e-36
rs9900613	17	74674857	T	C	0.431	-0.011	0.002	1.3e-09
rs8079218	17	76247744	G	A	0.497	-0.015	0.002	1.11e-14
rs9747839	17	81084522	G	C	0.476	0.019	0.002	4.46e-18
rs11874453	18	20750400	A	G	0.533	-0.012	0.002	1.36e-09
rs303753	18	21074922	A	G	0.345	-0.020	0.002	1.78e-23
rs78285907	18	21622003	T	A	0.089	0.020	0.003	3.1e-09
rs75354229	18	42078782	T	C	0.129	0.024	0.003	6.22e-19
rs8084255	18	48144754	T	C	0.376	0.020	0.002	1.26e-25
rs527544	18	51762866	T	G	0.427	0.014	0.002	8.21e-13
rs17758695	18	60920854	T	C	0.029	-0.056	0.006	1.67e-22
rs12956324	18	67537270	A	C	0.403	0.014	0.002	2.1e-13
rs72973711	18	74072245	T	A	0.067	-0.032	0.004	1.28e-17
rs7246841	19	1030431	C	T	0.731	0.022	0.002	3.78e-24
rs2358581	19	10391611	G	T	0.733	-0.028	0.002	9.36e-38
rs56121005	19	11414706	T	C	0.023	0.040	0.007	3.03e-09
rs2290669	19	16495774	C	A	0.786	-0.048	0.002	1.52e-94
rs4808683	19	17862925	G	C	0.458	-0.013	0.002	8.23e-12
rs4805881	19	33896432	C	A	0.665	-0.016	0.002	1.32e-16
rs2194067	19	35464727	T	C	0.416	0.013	0.002	2.31e-11
rs4760	19	44153100	G	A	0.153	-0.070	0.003	2.21e-161
rs73036517	19	45744842	G	A	0.259	-0.053	0.002	1.08e-137
rs309190	19	47629264	C	T	0.894	-0.030	0.003	7.05e-22
rs2617802	19	49114479	G	T	0.207	-0.015	0.002	1.59e-10
rs35112940	19	51738917	A	G	0.216	-0.026	0.002	5.21e-31
rs571497	19	7827830	A	G	0.155	-0.036	0.003	6.32e-45
rs34202160	1	101102673	G	T	0.358	-0.015	0.002	8.34e-15
rs6696259	1	101221482	C	G	0.409	0.018	0.002	3.74e-22
rs284317	1	10731625	G	A	0.498	0.017	0.002	3.52e-19
rs9429767	1	110496087	A	G	0.197	0.017	0.002	1.03e-13
rs630505	1	111737916	C	T	0.265	0.012	0.002	3.13e-09

rs2476601	1	114377568	G	A	0.900	0.053	0.003	3.35e-67
rs4970996	1	150506589	C	G	0.751	0.015	0.002	8.97e-13
rs568035	1	156110167	T	C	0.067	-0.025	0.004	9.21e-12
rs34599082	1	159175494	T	C	0.013	-0.151	0.008	1.93e-77
rs11587213	1	161184875	G	A	0.182	-0.020	0.002	7.7e-17
rs61804160	1	161620089	A	T	0.140	-0.030	0.003	8.08e-29
rs12737843	1	167010375	C	T	0.202	0.014	0.002	5.1e-10
rs3795503	1	180905694	T	C	0.315	-0.015	0.002	1.83e-14
rs1779809	1	182145545	T	C	0.334	0.014	0.002	1.36e-13
rs41272536	1	183440531	G	A	0.046	-0.034	0.005	3.65e-13
rs10494783	1	198663661	A	G	0.052	-0.044	0.004	4.43e-26
rs4844565	1	207227138	T	A	0.410	-0.011	0.002	1.01e-09
rs4844390	1	207934849	G	A	0.220	-0.027	0.002	4.97e-33
rs35571080	1	224637790	C	T	0.213	0.025	0.002	1.32e-28
rs2615061	1	225895806	A	G	0.117	-0.028	0.003	3.82e-22
rs6426584	1	227374949	T	A	0.335	-0.012	0.002	3.66e-09
rs533483	1	234765256	A	G	0.243	-0.017	0.002	1.91e-15
rs1886654	1	236105910	C	T	0.892	-0.057	0.003	5.08e-84
rs6684709	1	23850023	C	G	0.620	-0.021	0.002	1.84e-27
rs34298354	1	247588053	T	C	0.124	-0.024	0.003	5.9e-18
rs56188865	1	247606276	C	T	0.374	-0.027	0.002	7e-46
rs4925756	1	248048016	T	C	0.692	-0.015	0.002	3.83e-13
rs35755865	1	27140060	T	C	0.034	-0.041	0.005	3.18e-16
rs7549164	1	31224193	T	C	0.189	0.025	0.002	1.33e-24
rs3917932	1	36943916	G	C	0.577	-0.045	0.002	1.22e-127
rs6667709	1	42201943	T	C	0.370	-0.014	0.002	6.3e-13
rs3754224	1	43423622	C	T	0.271	-0.015	0.002	4.3e-12
rs74076327	1	56979431	C	T	0.045	-0.029	0.004	1.55e-10
rs1933295	1	62107021	G	A	0.777	-0.016	0.002	1.81e-11
rs10889574	1	66149341	A	G	0.357	-0.044	0.002	6.64e-106
rs61780038	1	67487376	A	C	0.565	-0.017	0.002	2.99e-16
rs41313381	1	79411968	A	C	0.030	0.048	0.006	1.47e-18
rs301819	1	8501786	G	A	0.583	-0.016	0.002	3.12e-17
rs10864368	1	8918313	C	T	0.503	0.022	0.002	1.15e-31
rs12138789	1	9192307	C	T	0.238	-0.015	0.002	2.08e-11
rs150649461	1	92925654	C	G	0.015	0.068	0.008	1.33e-17
rs9430574	1	9709072	A	G	0.330	-0.014	0.002	1.55e-12
rs34952318	20	11177055	A	G	0.050	-0.033	0.004	4.24e-14
rs6045615	20	1931582	C	A	0.330	-0.019	0.002	1.99e-22
rs4815552	20	2833578	G	A	0.351	0.012	0.002	7.61e-10
rs6059938	20	33187130	A	G	0.486	0.011	0.002	4.74e-09
rs67139609	20	36888321	G	C	0.145	0.017	0.003	2.47e-10
rs6029234	20	39259278	C	G	0.624	0.024	0.002	7.57e-37
rs763227	20	42205061	A	G	0.786	0.014	0.002	1.93e-09
rs2904270	20	49185272	A	G	0.517	-0.015	0.002	1.03e-16
rs2327028	20	8140925	T	C	0.476	-0.013	0.002	3.36e-13
rs4432538	20	8607393	A	G	0.509	-0.022	0.002	2.22e-33
rs28574812	21	16384940	G	A	0.158	-0.016	0.003	2.66e-10
rs9977672	21	40463283	A	G	0.259	-0.022	0.002	7.08e-25
rs35990176	21	44472118	C	A	0.451	0.012	0.002	6.46e-10
rs2839166	21	47656712	C	T	0.398	-0.016	0.002	2.97e-17
rs5746451	22	18126020	C	T	0.504	0.020	0.002	4.61e-26
rs448184	22	18293967	T	A	0.368	0.015	0.002	1.61e-13
rs34505104	22	24624609	G	A	0.305	-0.020	0.002	3.36e-23
rs9625746	22	29637658	C	G	0.412	-0.018	0.002	8.77e-22
rs2857633	22	30114333	C	T	0.727	-0.013	0.002	2.52e-10

rs2180142	22	31855248	A	T	0.051	-0.025	0.004	3.17e-09
rs713909	22	39532420	C	G	0.432	-0.020	0.002	2.75e-26
rs1033415	22	39888774	G	A	0.650	0.013	0.002	6.01e-10
rs47341	22	43560763	T	C	0.396	0.016	0.002	1.01e-15
rs2076211	22	44329078	T	C	0.158	-0.018	0.003	1.53e-12
rs796056	2	101735276	C	A	0.623	0.021	0.002	2.31e-27
rs6543094	2	102372131	C	T	0.772	0.019	0.002	1.86e-17
rs75726191	2	112914684	T	C	0.136	0.024	0.003	1.22e-16
rs6734238	2	113841030	G	A	0.402	0.036	0.002	2.59e-77
rs11688303	2	128410244	T	C	0.120	0.021	0.003	3.19e-13
rs1863176	2	12931092	C	G	0.497	0.019	0.002	6.27e-24
rs79716587	2	143886819	A	G	0.124	-0.036	0.003	9.22e-37
rs1371045	2	145787050	C	T	0.744	-0.014	0.002	1.26e-11
rs114427331	2	153381036	G	C	0.038	-0.036	0.005	6.36e-14
rs2729707	2	160687231	G	A	0.830	-0.031	0.002	1.28e-36
rs6746368	2	161222998	T	A	0.334	0.011	0.002	4.36e-09
rs2068330	2	163237390	G	C	0.361	0.019	0.002	2.87e-22
rs4632345	2	16702654	A	G	0.678	-0.014	0.002	3.75e-12
rs527393	2	169709757	A	G	0.468	-0.022	0.002	4.34e-33
rs17270882	2	182218046	C	G	0.239	0.031	0.002	4.07e-46
rs7573465	2	182315885	T	G	0.554	0.036	0.002	4.66e-83
rs8176528	2	188345017	A	G	0.297	-0.017	0.002	6.31e-17
rs12997100	2	192522423	C	T	0.071	0.027	0.004	9.38e-14
rs10931934	2	202119789	C	T	0.600	0.015	0.002	1.38e-15
rs1047891	2	211540507	A	C	0.315	-0.021	0.002	1.6e-26
rs10203838	2	213935900	C	T	0.418	0.012	0.002	4.66e-11
rs1250215	2	216253039	C	G	0.641	0.012	0.002	1.08e-09
rs114050631	2	219020958	T	C	0.011	-0.138	0.010	1.38e-45
rs58106596	2	232579379	A	G	0.227	-0.024	0.002	1.85e-25
rs10164769	2	237779229	T	C	0.743	0.027	0.002	7.73e-38
rs1260326	2	27730940	C	T	0.601	-0.033	0.002	9.83e-69
rs4666068	2	28623982	G	A	0.509	-0.012	0.002	1.15e-10
rs633323	2	31463044	T	A	0.698	0.015	0.002	2.87e-13
rs935655	2	46067445	G	T	0.762	0.015	0.002	8.11e-12
rs75475627	2	54787592	G	C	0.077	0.033	0.004	1.92e-20
rs10208769	2	61605614	A	T	0.516	0.022	0.002	6.48e-33
rs6731993	2	65642097	T	A	0.408	0.023	0.002	3.7e-33
rs60190483	2	70385245	C	T	0.057	-0.031	0.004	2.43e-15
rs7639292	3	107295665	T	C	0.168	-0.024	0.002	2.48e-22
rs13321783	3	119615375	C	T	0.394	-0.012	0.002	1.58e-09
rs1822534	3	12266804	G	A	0.394	-0.026	0.002	8.29e-43
rs3773312	3	12946142	A	G	0.134	0.018	0.003	3.67e-10
rs78208917	3	132229708	G	A	0.090	0.026	0.003	7.98e-16
rs2012610	3	140933565	T	G	0.127	0.028	0.003	2.99e-23
rs9819371	3	141206800	T	C	0.065	-0.044	0.004	5.94e-32
rs73028871	3	14277058	T	C	0.134	-0.017	0.003	9.3e-10
rs6440732	3	150990510	A	C	0.828	0.023	0.002	1.35e-21
rs56082403	3	156797225	C	T	0.405	0.015	0.002	5.5e-15
rs10936588	3	169319801	A	G	0.636	-0.014	0.002	6.95e-14
rs4074672	3	183730295	T	C	0.370	0.018	0.002	3.87e-21
rs9867398	3	185912816	T	C	0.094	0.022	0.003	2.8e-12
rs2268829	3	185989567	A	G	0.785	-0.014	0.002	2.66e-10
rs9829114	3	196518623	A	G	0.414	-0.034	0.002	7.45e-72
rs11927257	3	27334294	T	C	0.282	-0.013	0.002	2.97e-10
rs2371108	3	27757018	T	G	0.389	0.019	0.002	3.11e-24
rs11706384	3	39296881	T	G	0.236	0.016	0.002	1.72e-13

rs1366045	3	42909050	C	T	0.384	-0.025	0.002	5.44e-39
rs116781404	3	48730203	T	C	0.043	0.028	0.005	1.46e-09
rs6779340	3	58033701	G	C	0.337	-0.018	0.002	4.51e-19
rs832190	3	63842629	T	C	0.633	-0.013	0.002	3.88e-12
rs35592432	3	71355240	C	G	0.027	0.040	0.006	5.47e-11
rs830623	3	71677746	C	A	0.170	0.014	0.002	3.88e-09
rs4504118	3	98422588	C	G	0.559	0.013	0.002	6.34e-12
rs113473633	4	103449131	G	A	0.026	-0.051	0.006	3.24e-16
rs144317085	4	105806108	T	A	0.034	0.050	0.005	3e-22
rs10006495	4	120263315	G	T	0.354	-0.016	0.002	1.35e-15
rs11735662	4	145026126	T	C	0.033	0.052	0.005	4.74e-24
rs2305980	4	151171269	A	T	0.255	0.014	0.002	6.67e-11
rs6854855	4	152247161	G	A	0.487	0.016	0.002	9.69e-19
rs140311179	4	26295540	G	C	0.051	-0.026	0.004	1.08e-09
rs73191188	4	3105200	A	G	0.353	-0.016	0.002	1.23e-17
rs28530750	4	36312542	A	G	0.043	0.056	0.005	1.83e-33
rs987121	4	38329783	T	A	0.606	0.015	0.002	4.76e-16
rs13132853	4	38680015	G	A	0.355	0.020	0.002	1.88e-25
rs218264	4	55408875	T	A	0.251	0.026	0.002	2.52e-33
rs6554195	4	55500714	T	G	0.484	-0.019	0.002	3.9e-26
rs28695311	4	55992105	C	G	0.506	-0.011	0.002	6.36e-10
rs2412771	4	57761417	C	T	0.417	-0.018	0.002	4.42e-22
rs6831368	4	6969919	G	A	0.362	-0.020	0.002	2.02e-25
rs35734242	4	706700	C	T	0.428	0.022	0.002	6.3e-29
rs16846876	4	72592491	T	A	0.329	-0.021	0.002	7.39e-27
rs16850073	4	74703999	T	C	0.374	0.043	0.002	8.76e-109
rs16850408	4	74932807	A	C	0.369	0.059	0.002	1e-200
rs4640621	4	80946541	C	A	0.632	-0.013	0.002	2.01e-11
rs116746683	4	83471084	T	C	0.063	-0.030	0.004	7.86e-15
rs10028284	4	89752913	T	A	0.177	-0.015	0.002	3.25e-10
rs28378092	4	90228807	T	C	0.458	0.012	0.002	6.64e-10
rs62387565	5	100024839	T	C	0.484	0.014	0.002	1.1e-14
rs4535497	5	1107428	A	C	0.570	-0.013	0.002	2.34e-11
rs79692389	5	111033606	C	G	0.061	0.026	0.004	9.39e-12
rs257063	5	114806819	T	C	0.741	-0.014	0.002	4.91e-11
rs79272926	5	118726637	A	C	0.222	-0.022	0.002	1.04e-23
rs6878780	5	122093740	C	T	0.427	0.013	0.002	1.02e-12
rs7705526	5	1285974	A	C	0.327	0.029	0.002	8.3e-43
rs11242109	5	131677047	T	G	0.477	-0.033	0.002	5.08e-72
rs79237520	5	134712566	T	C	0.023	0.040	0.006	2.13e-10
rs4391200	5	141509537	G	A	0.619	0.023	0.002	7.86e-34
rs72660299	5	150564634	T	C	0.063	-0.024	0.004	7.4e-11
rs4704826	5	156392082	A	C	0.634	-0.016	0.002	9.71e-17
rs9313822	5	159207694	A	G	0.052	0.025	0.004	9.35e-10
rs10054235	5	173087427	C	A	0.349	0.013	0.002	4.9e-11
rs2561758	5	173205282	G	A	0.723	-0.036	0.002	4.2e-68
rs13180726	5	179126457	A	G	0.826	-0.021	0.002	2.71e-18
rs464609	5	34654477	A	G	0.544	0.013	0.002	3e-12
rs987107	5	35875227	A	G	0.261	0.020	0.002	2.52e-22
rs11744663	5	57315635	A	G	0.190	-0.019	0.002	1.72e-15
rs2910580	5	57527886	A	T	0.659	-0.015	0.002	2.83e-15
rs4703881	5	71741851	A	G	0.879	-0.036	0.003	7.28e-38
rs2432142	5	96275201	A	G	0.434	0.013	0.002	1.09e-11
rs1087924	6	10523826	C	G	0.531	-0.014	0.002	4.66e-14
rs9487043	6	109610474	T	C	0.487	0.023	0.002	4.35e-36
rs10872223	6	121814703	T	C	0.183	0.014	0.002	3.57e-09

rs549302	6	122734972	A	G	0.545	0.020	0.002	8.48e-28
rs9375447	6	126874091	G	A	0.495	-0.014	0.002	6.34e-14
rs7776054	6	135418916	G	A	0.261	-0.047	0.002	2.48e-110
rs12214269	6	135846518	A	G	0.546	0.018	0.002	5.06e-23
rs6924387	6	137082948	G	A	0.412	0.017	0.002	1.48e-18
rs72978754	6	138053203	C	T	0.066	-0.028	0.004	2.45e-14
rs9390461	6	147701217	G	A	0.538	0.015	0.002	8.51e-17
rs7751717	6	153005229	A	G	0.200	-0.014	0.002	2.23e-09
rs1738074	6	159465977	C	T	0.568	-0.020	0.002	3.74e-28
rs1322599	6	16758425	T	C	0.174	-0.031	0.002	9.97e-36
rs73735248	6	21809195	T	C	0.159	0.015	0.003	9.08e-10
rs75628831	6	22316185	A	G	0.086	0.031	0.003	4.98e-20
rs707910	6	29910663	A	G	0.187	-0.073	0.002	1e-200
rs114892859	6	31369278	T	G	0.019	-0.085	0.007	1.92e-35
rs9394299	6	35449326	T	C	0.104	-0.028	0.003	1.21e-19
rs12203592	6	396321	T	C	0.210	0.022	0.002	1.61e-21
rs10948036	6	42510305	A	C	0.210	0.023	0.002	1.97e-24
rs17713864	6	43695465	G	A	0.029	0.035	0.005	1.54e-10
rs998584	6	43757896	A	C	0.484	0.016	0.002	9.24e-17
rs729761	6	43804571	G	T	0.713	0.015	0.002	3.38e-13
rs639728	6	44239922	T	C	0.441	-0.012	0.002	5.99e-10
rs1285886	6	7140831	A	G	0.197	0.026	0.002	5.1e-30
rs4707609	6	90946479	C	T	0.360	-0.021	0.002	3.21e-28
rs10808139	7	104709946	A	G	0.119	0.018	0.003	6.06e-10
rs342294	7	106372622	C	T	0.456	-0.017	0.002	4.9e-21
rs38859	7	116379094	T	C	0.449	0.011	0.002	9.71e-10
rs9656395	7	130575884	G	A	0.095	-0.028	0.003	2.97e-18
rs7803075	7	130742066	G	A	0.734	-0.024	0.002	5.47e-31
rs13231262	7	148886058	T	C	0.656	-0.016	0.002	3.99e-16
rs4721659	7	17869147	G	T	0.123	-0.018	0.003	5.3e-11
rs7776857	7	22754768	T	G	0.658	-0.019	0.002	1.89e-21
rs7792934	7	2393016	G	A	0.789	-0.015	0.002	4.69e-11
rs798555	7	2759473	C	T	0.295	0.019	0.002	4.81e-20
rs2158799	7	28277107	G	C	0.612	0.043	0.002	7.86e-114
rs56388170	7	28724374	T	G	0.295	0.059	0.002	2.73e-185
rs2710804	7	36084529	C	T	0.376	0.021	0.002	1.92e-27
rs10260281	7	38270550	C	G	0.237	0.013	0.002	5.33e-10
rs3735485	7	45009341	G	A	0.845	0.041	0.003	2.34e-57
rs2692540	7	47465325	G	C	0.428	-0.011	0.002	1.19e-09
rs6583435	7	50293405	A	C	0.680	0.017	0.002	8.7e-18
rs4948097	7	56056571	G	A	0.758	-0.018	0.002	3.03e-16
rs12540307	7	65662767	T	C	0.049	-0.039	0.004	4.76e-18
rs1474419	7	6692605	C	T	0.580	0.018	0.002	3.11e-22
rs62466318	7	73042085	T	C	0.202	-0.021	0.002	9.8e-19
rs7781268	7	75748183	A	G	0.246	-0.013	0.002	3.66e-09
rs60466842	7	8024840	A	C	0.088	-0.030	0.003	1.04e-19
rs4272	7	92236829	G	A	0.213	0.035	0.002	2.41e-54
rs445	7	92408370	T	C	0.099	-0.095	0.003	1e-200
rs12538820	7	99340049	A	G	0.113	-0.019	0.003	1.62e-10
rs77912140	8	100824826	G	A	0.044	0.027	0.004	1.66e-09
rs3847147	8	105784723	A	G	0.307	-0.012	0.002	2.99e-09
rs17209630	8	106296090	A	G	0.281	0.014	0.002	3.25e-12
rs11250076	8	10647823	G	A	0.575	0.021	0.002	2.44e-30
rs4734879	8	106583124	G	A	0.276	-0.022	0.002	1.34e-26
rs71510325	8	126298640	T	C	0.597	0.014	0.002	3.34e-13
rs10087240	8	129012574	T	C	0.458	0.020	0.002	6.13e-27

rs145209947	8	130694185	A	C	0.014	-0.075	0.009	3.19e-18
rs7018391	8	131243506	C	G	0.140	0.016	0.003	3.26e-09
rs7005996	8	142241681	T	C	0.906	0.024	0.003	4.06e-13
rs6985508	8	142337734	A	G	0.358	-0.027	0.002	1.57e-39
rs12550612	8	22966769	A	G	0.821	-0.033	0.002	1.09e-41
rs755951	8	27226790	C	A	0.403	-0.013	0.002	1.02e-12
rs13248936	8	30316162	G	A	0.498	0.013	0.002	1.17e-12
rs6474514	8	38835269	C	T	0.412	0.011	0.002	3.59e-09
rs28571765	8	55449281	C	T	0.207	-0.022	0.002	1.32e-20
rs1511724	8	56796697	G	C	0.422	-0.017	0.002	1.15e-18
rs2875974	8	61419728	A	G	0.373	0.021	0.002	3.54e-29
rs7846314	8	61650831	T	A	0.187	0.047	0.002	5.71e-88
rs4276676	8	68795618	C	T	0.469	-0.019	0.002	2.22e-25
rs12716647	8	6901304	C	G	0.638	0.012	0.002	1.12e-10
rs1947897	8	78946874	C	G	0.336	-0.014	0.002	1.71e-13
rs62510269	8	79012343	G	A	0.146	-0.036	0.003	1.77e-43
rs10102877	8	79598362	A	G	0.685	-0.013	0.002	3.91e-11
rs7864482	9	102268861	G	A	0.377	0.013	0.002	2e-11
rs12683699	9	112738298	G	A	0.160	0.022	0.003	5.16e-18
rs10980797	9	113912553	G	A	0.488	-0.027	0.002	1.19e-48
rs7855091	9	114655098	A	G	0.307	0.015	0.002	1.11e-13
rs12343532	9	115978022	A	T	0.272	-0.015	0.002	2.49e-13
rs10986338	9	127191232	A	G	0.650	-0.017	0.002	1.93e-17
rs2519093	9	136141870	T	C	0.186	-0.041	0.002	8.55e-68
rs2506699	9	136946799	G	A	0.484	0.014	0.002	4.87e-14
rs4413892	9	139330158	A	G	0.276	0.031	0.002	2.87e-46
rs41317014	9	139929080	T	C	0.248	-0.020	0.002	3.25e-19
rs7036656	9	21990457	T	C	0.722	0.028	0.002	1.25e-42
rs12376511	9	22142756	C	T	0.164	-0.034	0.002	6.16e-43
rs34881325	9	2622134	T	C	0.379	0.017	0.002	1.16e-18
rs13291664	9	282738	G	A	0.187	0.026	0.002	1.57e-28
rs3793537	9	35687556	C	G	0.290	0.020	0.002	1.53e-22
rs10973700	9	38196117	C	G	0.486	-0.013	0.002	1.36e-12
rs447124	9	4746539	T	C	0.491	0.017	0.002	4.86e-20
rs1887428	9	4984530	C	G	0.625	-0.015	0.002	9.58e-15
rs626416	9	79326680	C	G	0.763	-0.017	0.002	2.81e-14
rs796003	9	86595801	T	G	0.258	-0.025	0.002	8.84e-33
rs4877024	9	91520860	G	A	0.381	-0.012	0.002	5.91e-10
rs10992394	9	95433830	A	G	0.230	0.015	0.002	2.14e-12
rs28821528	6	32528913	A	G	0.412	-0.036	0.003	3.23e-45
rs4746522	10	64437684	A	G	0.278	0.013	0.002	7.17e-10
rs71468750	11	120205560	A	G	0.413	0.014	0.002	1.39e-11
rs14408	11	308314	C	T	0.368	0.044	0.002	2.97e-93
rs374039502	13	108960385	A	T	0.022	-0.065	0.007	2.13e-20
rs576566496	17	38140269	A	G	0.026	0.087	0.006	3.22e-42
rs79135050	17	38215203	C	A	0.030	0.068	0.006	1.44e-28
rs76306941	17	61944620	G	T	0.581	-0.012	0.002	1.04e-09
rs55874505	18	43816804	G	T	0.584	-0.019	0.002	5.5e-20
rs74176367	19	1180039	C	T	0.355	0.014	0.002	2.58e-10
rs143998157	1	161561236	T	C	0.392	-0.014	0.002	2.54e-11
rs6676472	1	200294113	T	C	0.712	0.014	0.002	1.96e-11
rs7554039	1	22303188	A	G	0.243	0.016	0.002	1.15e-12
rs140958972	1	46428213	G	A	0.435	-0.020	0.002	6.18e-25
rs12121236	1	56617911	A	C	0.352	0.015	0.002	4.41e-14
rs3016112	22	17517388	T	C	0.750	-0.018	0.002	9.57e-16
rs58270357	2	112236636	T	C	0.081	-0.026	0.004	4.03e-10

rs377110161	2	23963462	G	A	0.128	0.037	0.003	1.56e-36
rs4599108	2	85543222	T	C	0.488	-0.016	0.002	1.31e-16
rs150918492	3	128389659	A	G	0.265	-0.027	0.002	9.95e-36
rs13063578	3	47087837	A	T	0.401	0.029	0.002	1.25e-44
rs11130982	3	64728312	G	T	0.700	0.012	0.002	3.53e-09
rs11960649	5	148203144	C	A	0.553	-0.020	0.002	3.58e-24
rs13165502	5	54972181	A	T	0.652	-0.014	0.002	9.3e-12
rs66514959	5	68611583	A	G	0.444	-0.022	0.002	5.66e-30
rs71536939	6	170486950	G	A	0.783	0.018	0.003	2.38e-11
rs76819034	6	32457814	C	T	0.437	0.070	0.002	1e-200
rs573635286	6	32489556	T	G	0.238	-0.074	0.003	1.61e-144
rs1761678	6	7090640	T	G	0.871	-0.020	0.003	2.25e-11
rs201150852	6	87837608	C	T	0.458	-0.017	0.002	3.78e-18
rs28555390	7	137607217	T	C	0.620	0.012	0.002	2.31e-09
rs10104995	8	103901234	T	C	0.231	-0.022	0.002	4.74e-21
rs59697075	8	130618150	T	C	0.587	-0.039	0.002	1.82e-85
rs10808538	8	87169063	A	G	0.296	0.013	0.002	3.6e-10
rs10815052	9	4648694	T	C	0.453	-0.012	0.002	1.28e-09
rs1288649	9	91426717	G	T	0.612	-0.021	0.002	7.74e-25
rs6502497	17	16102530	D	I	0.464	0.021	0.002	5.67e-24
rs2409752	8	11092685	D	I	0.535	0.017	0.002	4.18e-16

Table S14. 347 SNPs significantly associated with eosinophil percentage of white cells used IVs in forward MR analyses derived from Vuckovic D et al. Chr: Chromosome; EA: Effect allele; NEA: Non-effect allele; EAF: Effect allele frequency.

SNP	Chr	Pos	EA	NEA	EAF	Beta	SE	P-value
rs159963	1	8504421	A	C	0.583	0.025	0.002	1.4e-30
rs4908832	1	9338959	T	C	0.387	0.016	0.002	1.2e-13
rs57959755	1	9358890	A	G	0.162	0.018	0.003	8.3e-10
rs35249183	1	12099345	G	A	0.100	0.038	0.004	1.8e-25
rs10799794	1	23701118	A	C	0.736	-0.014	0.002	4.2e-09
rs2229586	1	24200903	C	G	0.570	0.022	0.002	2.5e-24
rs7416513	1	26647949	C	G	0.762	-0.017	0.002	1.5e-11
rs76022305	1	28631300	T	C	0.069	-0.032	0.004	2.8e-14
rs1004870	1	42370787	T	C	0.589	0.024	0.002	3.3e-29
rs567910619	1	65210790	T	C	0.011	-0.080	0.011	1.5e-13
rs12408934	1	65423447	A	G	0.103	-0.047	0.004	9.3e-41
rs2802469	1	66017386	T	C	0.644	-0.014	0.002	1.4e-10
rs6672038	1	87751670	T	C	0.119	0.039	0.003	1e-32
rs1088318	1	93733057	T	C	0.379	-0.021	0.002	2.1e-22
rs76336747	1	101648318	A	G	0.114	-0.043	0.003	2.8e-38
rs2793925	1	120652158	A	T	0.684	0.017	0.002	7.1e-14
rs2596251	1	145103928	A	G	0.388	0.020	0.003	4.6e-09
rs11204682	1	150595537	T	G	0.223	-0.035	0.003	2.8e-41
rs111548294	1	150911337	T	C	0.013	0.078	0.010	2.6e-16
rs2242193	1	154995503	A	G	0.420	0.015	0.002	2.1e-11
rs6672031	1	172796505	G	C	0.487	0.020	0.002	4.8e-21
rs1099448	1	173231898	T	C	0.467	0.019	0.002	1.1e-19
rs6658698	1	181060791	A	G	0.618	-0.015	0.002	5.5e-12
rs17668272	1	198623842	T	G	0.118	-0.043	0.003	2.8e-39
rs1036332	1	199012478	C	A	0.738	-0.033	0.002	1.5e-42
rs10900595	1	204511602	A	C	0.713	0.016	0.002	3.9e-12
rs3849269	1	206676143	T	C	0.530	-0.013	0.002	2.1e-09
rs59150958	1	221106304	T	C	0.152	-0.026	0.003	1.8e-18
rs12142635	1	226976332	A	C	0.057	-0.030	0.005	1.3e-10
rs4362021	1	234696177	A	T	0.534	0.013	0.002	4.4e-09
rs4660129	1	236094087	G	A	0.892	0.025	0.003	1.9e-13
rs4149909	1	242023898	G	A	0.033	-0.040	0.006	2.9e-11
rs346835	2	8438693	T	C	0.329	-0.027	0.002	3.7e-32
rs62105489	2	8757326	T	C	0.053	-0.030	0.005	5.6e-10
rs780092	2	27743154	G	A	0.161	0.021	0.003	2e-13
rs11127154	2	28685967	T	A	0.492	-0.023	0.002	1.9e-25
rs336039	2	38258827	T	C	0.599	-0.013	0.002	2e-09
rs77059113	2	43672508	G	T	0.073	-0.032	0.004	1.7e-15
rs11126037	2	65663158	A	C	0.327	-0.014	0.002	2.7e-10
rs6546661	2	71277382	C	T	0.304	-0.017	0.002	1.7e-13
rs11126424	2	74262353	C	T	0.306	-0.016	0.002	1.2e-12
rs2366640	2	85810734	C	A	0.319	-0.014	0.002	2.8e-09
rs2579500	2	97201682	A	G	0.390	0.050	0.002	9.7e-116
rs6543250	2	98640981	G	A	0.604	-0.015	0.002	2.1e-11
rs55948744	2	102805911	G	T	0.052	0.030	0.005	9.7e-10
rs12470864	2	102926362	A	G	0.388	0.069	0.002	1e-200
rs66494224	2	112002314	C	T	0.226	0.031	0.003	1e-34
rs4848100	2	112388532	T	C	0.768	0.028	0.003	3.1e-29
rs1257192	2	134991024	G	A	0.812	0.017	0.003	2.3e-10
rs4954388	2	136808142	T	C	0.772	-0.016	0.003	3.2e-10
rs12472034	2	136828273	G	C	0.324	-0.014	0.002	5.3e-10

rs1427508	2	145367024	A	T	0.826	0.021	0.003	4.1e-14
rs10195713	2	158520905	T	C	0.864	0.031	0.003	1.4e-22
rs6734311	2	182139508	A	T	0.630	0.013	0.002	2.5e-09
rs539299744	2	197006644	T	A	0.135	-0.021	0.003	1.5e-11
rs1263595	2	207947633	C	T	0.287	-0.014	0.002	2.9e-09
rs715	2	211543055	C	T	0.311	0.025	0.002	4e-28
rs12619285	2	213824045	G	A	0.265	-0.063	0.002	8.1e-151
rs35409523	2	213908457	A	G	0.076	0.054	0.004	1.1e-40
rs34855578	2	231114990	G	C	0.186	0.026	0.003	1.7e-21
rs1057258	2	234115629	T	C	0.178	-0.037	0.003	1.3e-41
rs34290285	2	242698640	A	G	0.256	-0.046	0.002	4.4e-80
rs76474320	2	242806022	C	T	0.078	0.033	0.004	1.1e-16
rs13084872	3	3068148	T	C	0.359	0.014	0.002	3.5e-10
rs6787336	3	3153194	A	G	0.287	0.037	0.002	5.6e-55
rs73019235	3	3164321	A	T	0.123	0.020	0.003	1.1e-09
rs155266	3	10230728	A	T	0.571	0.018	0.002	1e-16
rs2960422	3	12334991	A	G	0.602	-0.029	0.002	7.3e-41
rs9872485	3	16918405	G	T	0.164	-0.017	0.003	2.4e-09
rs13073683	3	25393837	C	T	0.399	0.015	0.002	3e-12
rs6795177	3	27800734	G	A	0.479	0.015	0.002	1.2e-12
rs7646283	3	33046480	T	C	0.367	0.027	0.002	6e-34
rs2228467	3	42906116	C	T	0.061	0.050	0.004	2.2e-29
rs4072859	3	49032205	C	G	0.659	0.027	0.002	9.7e-31
rs9815974	3	71439472	A	G	0.665	-0.016	0.002	3.4e-12
rs4677079	3	72125863	T	G	0.498	0.015	0.002	6.4e-13
rs7649812	3	98449191	G	C	0.242	0.018	0.002	5.8e-13
rs4618204	3	101281534	C	T	0.444	0.019	0.002	5.4e-18
rs9868582	3	112052927	T	G	0.452	-0.026	0.002	2.8e-33
rs2399441	3	112587012	C	T	0.350	-0.020	0.002	2.2e-19
rs2715275	3	121781689	G	A	0.202	0.021	0.003	5.6e-16
rs9877627	3	128249249	G	A	0.174	-0.085	0.003	1e-200
rs2712399	3	128432953	G	C	0.409	-0.030	0.002	2.8e-42
rs115702896	3	129066698	C	T	0.019	-0.052	0.008	3.4e-11
rs6441306	3	159952278	G	A	0.433	-0.013	0.002	1.4e-09
rs191674933	3	169488691	T	A	0.240	0.025	0.003	3.6e-23
rs73046033	3	171527955	G	T	0.116	-0.022	0.003	7.3e-11
rs9815874	3	188441161	T	C	0.299	0.027	0.002	2.1e-32
rs9818987	3	194402430	T	C	0.306	0.015	0.002	1.2e-10
rs12152276	3	196368501	G	A	0.081	-0.035	0.004	2.9e-19
rs1828803	4	2689449	A	C	0.393	0.015	0.002	2.8e-11
rs13139941	4	7071741	G	A	0.803	-0.017	0.003	2.7e-10
rs76981581	4	38416046	G	C	0.048	-0.032	0.005	2.6e-10
rs13112758	4	38555527	T	C	0.283	0.014	0.002	1.9e-09
rs12331049	4	38655772	T	C	0.212	0.074	0.003	2.6e-175
rs2254075	4	39030183	G	A	0.666	-0.015	0.002	1.4e-10
rs4694639	4	74699054	T	C	0.568	0.017	0.002	2.9e-14
rs13138355	4	83545976	T	C	0.185	-0.040	0.003	1.3e-47
rs13107325	4	103188709	T	C	0.075	-0.030	0.004	2.5e-13
rs6827756	4	123184411	C	T	0.637	-0.023	0.002	8.9e-26
rs2390353	4	123602097	C	T	0.232	0.023	0.003	3.6e-19
rs13120371	4	139092719	G	A	0.327	0.015	0.002	1.4e-11
rs57199846	4	153353956	T	A	0.297	0.019	0.002	2.8e-16
rs2853677	5	1287194	A	G	0.577	0.024	0.002	2.8e-28
rs11737939	5	10622792	T	C	0.165	0.025	0.003	4.3e-18
rs16903574	5	14610309	G	C	0.076	0.036	0.004	2.4e-18
rs4594881	5	35846815	T	G	0.341	-0.034	0.002	3.4e-51

rs4703730	5	76549688	T	C	0.517	-0.016	0.002	2.1e-13
rs34495	5	98265807	T	G	0.304	-0.021	0.002	2.8e-19
rs6884604	5	110084154	C	T	0.089	0.026	0.004	6.3e-12
rs141405779	5	110424932	A	G	0.138	-0.056	0.003	1.5e-74
rs35771899	5	130865179	A	T	0.685	-0.023	0.002	2e-21
rs2188962	5	131770805	T	C	0.428	-0.065	0.002	1e-200
rs244689	5	133422816	G	A	0.877	-0.022	0.003	6.4e-12
rs6580223	5	141489027	T	G	0.617	0.033	0.002	3.8e-50
rs56330463	5	148200011	C	T	0.552	0.049	0.002	5e-114
rs6869605	5	150452866	C	A	0.123	-0.022	0.003	5.5e-12
rs11748149	5	156977393	A	G	0.106	0.021	0.003	5.8e-10
rs114925538	5	169696756	T	C	0.051	-0.037	0.005	4.8e-14
rs35716097	5	176806636	T	C	0.298	0.015	0.002	1.5e-10
rs2666969	6	336410	G	A	0.254	-0.037	0.003	1.3e-30
rs9504361	6	577820	G	A	0.446	-0.029	0.002	5e-39
rs3132682	6	30044388	C	G	0.588	-0.047	0.002	2.7e-104
rs9266321	6	31330360	C	G	0.184	0.045	0.003	1.7e-58
rs192955957	6	32486965	T	C	0.104	0.040	0.005	2.3e-18
rs28383314	6	32587213	C	T	0.625	0.040	0.002	6.3e-74
rs12206050	6	33564296	T	A	0.185	0.033	0.003	3.2e-33
rs9462094	6	35513603	T	C	0.431	-0.024	0.002	5.1e-21
rs11751949	6	42219296	A	G	0.071	-0.034	0.004	5.8e-16
rs76236447	6	42225993	C	G	0.072	0.039	0.004	2e-21
rs62414592	6	42516867	A	G	0.192	-0.019	0.003	1.4e-12
rs9369573	6	45689356	T	C	0.185	0.017	0.003	7e-10
rs2025489	6	87814959	G	A	0.527	0.021	0.002	1.5e-22
rs905670	6	90958502	A	G	0.351	-0.035	0.002	1.2e-55
rs12208103	6	107442431	T	C	0.376	-0.033	0.002	1.2e-50
rs783646	6	117286220	C	G	0.817	-0.022	0.003	4.5e-15
rs9402682	6	135406183	T	G	0.240	0.036	0.003	2.9e-45
rs4548024	6	138165744	C	T	0.237	-0.017	0.003	2.2e-11
rs391875	6	149795136	A	G	0.405	0.016	0.002	5.5e-13
rs3093023	6	167534290	A	G	0.433	0.013	0.002	1.3e-09
rs6955702	7	3157702	G	A	0.524	0.025	0.002	2.3e-30
rs10950642	7	17016646	A	G	0.363	-0.021	0.002	1.4e-20
rs57834782	7	20502828	A	T	0.245	-0.056	0.002	2e-114
rs12537614	7	22789551	C	G	0.592	-0.023	0.002	3.3e-26
rs2158799	7	28277107	G	C	0.610	-0.021	0.002	8.1e-21
rs60600003	7	37382465	G	T	0.101	0.038	0.004	1.2e-26
rs12536178	7	45178748	T	C	0.223	0.015	0.003	4.6e-09
rs61198215	7	50329697	A	G	0.376	0.024	0.002	8.5e-27
rs12718731	7	50436948	G	A	0.399	-0.013	0.002	8e-10
rs80271829	7	50443141	C	T	0.086	-0.030	0.004	2e-15
rs62466318	7	73042085	T	C	0.203	0.019	0.003	2e-12
rs13226583	7	75454152	T	A	0.116	-0.062	0.003	1.3e-76
rs55879743	7	75470364	T	C	0.067	0.077	0.004	7.6e-70
rs4279	7	91764128	A	C	0.395	0.018	0.002	1e-16
rs73187852	7	101878336	T	C	0.275	0.016	0.002	9.8e-12
rs12705849	7	112782556	A	G	0.407	-0.021	0.002	1.5e-22
rs3925331	7	124446137	G	C	0.287	0.014	0.002	4.4e-09
rs6959832	7	135289854	A	G	0.507	-0.015	0.002	1e-11
rs6975036	7	138780350	A	G	0.218	0.023	0.003	4.8e-19
rs12530946	7	148887942	G	A	0.614	0.048	0.002	1.8e-106
rs2979256	8	8871710	T	C	0.450	0.018	0.002	4.3e-17
rs2119690	8	19859539	A	G	0.288	0.014	0.002	2.1e-09
rs4871849	8	22964719	A	G	0.708	-0.027	0.002	2.7e-31

rs73681119	8	27272123	G	C	0.324	-0.015	0.002	1.9e-10		
rs45577137	8	48651633	G	A	0.045	-0.066	0.005	2.7e-33		
rs74915527	8	48806383	T	C	0.097	0.026	0.004	3.5e-13		
rs7846314	8	61650831	T	A	0.187	-0.051	0.003	3e-76		
rs6989099	8	66898262	C	T	0.317	-0.017	0.002	3.9e-14		
rs10957978	8	81285139	T	G	0.647	-0.016	0.002	1.6e-12		
rs13255935	8	95965685	A	C	0.513	0.013	0.002	1.7e-09		
rs1493935	8	119848956	A	G	0.669	0.020	0.002	7e-18		
rs35779726	8	126154899	C	G	0.131	0.020	0.003	1.7e-10		
rs11786536	8	129000416	A	G	0.164	-0.025	0.003	2.6e-17		
rs77959102	8	130602928	C	T	0.143	-0.060	0.003	1.5e-83		
rs4236746	8	130699861	G	A	0.975	0.062	0.007	4.6e-20		
rs2294023	8	134126039	G	A	0.219	0.015	0.003	4.7e-09		
rs10087637	8	142239318	A	C	0.637	-0.015	0.002	3.2e-11		
rs34173062	8	145158607	A	G	0.072	0.058	0.004	4e-40		
rs460631	9	4851440	G	A	0.885	0.023	0.003	8.8e-12		
rs10974980	9	5140671	G	C	0.270	0.029	0.002	1.6e-33		
rs4142528	9	6172296	A	T	0.672	-0.077	0.002	1e-200		
rs1547258	9	6523056	C	T	0.708	-0.021	0.002	3e-19		
rs10810657	9	16884586	A	T	0.625	-0.015	0.002	5.6e-11		
rs11789226	9	34975508	C	T	0.501	-0.014	0.002	4.1e-11		
rs7869321	9	86520782	T	C	0.255	0.031	0.002	4.9e-36		
rs76179738	9	94127317	T	C	0.185	-0.027	0.003	1.6e-23		
rs4742774	9	102562927	C	T	0.378	0.013	0.002	2.6e-09		
rs496475	9	113638236	G	T	0.387	0.033	0.002	8.9e-52		
rs1322054	9	117669299	G	A	0.435	-0.026	0.002	1e-33		
rs7040707	9	117680732	G	A	0.048	-0.034	0.005	3.5e-10		
rs605642	9	135880469	A	C	0.582	0.027	0.002	2.5e-34		
rs671050	9	136001966	C	T	0.674	-0.014	0.002	1.4e-09		
rs2810489	9	136922700	A	G	0.258	-0.028	0.002	4.1e-31		
rs11253517	10	970524	A	G	0.238	0.034	0.003	1.3e-42		
rs11255507	10	8109615	G	T	0.178	0.021	0.003	6e-14		
rs2646438	10	8565675	A	G	0.566	-0.020	0.002	3.3e-20		
rs1775554	10	9054340	C	A	0.423	-0.040	0.002	3.1e-76		
rs2992335	10	26727934	G	C	0.598	-0.030	0.002	5.8e-44		
rs2807740	10	28784483	T	C	0.769	0.027	0.003	6e-27		
rs17482472	10	44859618	A	G	0.099	-0.024	0.004	4e-11		
rs10995251	10	64398466	T	C	0.369	-0.040	0.002	1.7e-74		
rs72834751	10	64559465	T	C	0.013	-0.077	0.010	3.1e-14		
rs61855876	10	65357541	T	C	0.163	-0.017	0.003	2.1e-09		
rs10998535	10	70805373	T	C	0.215	0.018	0.003	2.2e-12		
rs564443	10	89807917	A	G	0.163	0.022	0.003	1.5e-14		
rs7897422	10	90849704	C	T	0.191	0.022	0.003	1.2e-16		
rs7918084	10	94429467	T	C	0.550	0.029	0.002	6.4e-42		
rs3862006	10	111750764	A	G	0.148	-0.018	0.003	4.4e-09		
rs7080536	10	115348046	A	G	0.043	-0.050	0.005	2.6e-21		
rs3781455	10	126347688	C	T	0.305	0.018	0.002	1.6e-15		
rs28475163	11	327143	A	G	0.234	-0.034	0.003	1.7e-42		
rs61871200	11	2959313	T	A	0.769	-0.021	0.003	7.3e-17		
rs360124	11	9802228	C	G	0.904	-0.023	0.004	1.1e-10		
rs34439695	11	33901483	T	C	0.035	-0.039	0.006	4.2e-11		
rs12577643	11	47467172	T	A	0.326	0.016	0.002	7.2e-13		
rs34108746	11	57146225	G	A	0.077	-0.039	0.004	1.9e-22		
rs663743	11	64107735	A	G	0.341	0.015	0.002	5.7e-11		
rs669371	11	65674153	T	G	0.542	0.028	0.002	6.3e-38		
rs7936323	11	76293758	A	G	0.478	0.042	0.002	2.2e-85		

rs11236813	11	76343427	C	G	0.101	-0.028	0.004	3e-15
rs4409785	11	95311422	C	T	0.172	0.018	0.003	4.8e-10
rs637064	11	108140909	T	C	0.555	0.021	0.002	4.4e-23
rs10466533	11	116612128	G	A	0.074	-0.030	0.004	1.1e-13
rs11216183	11	116781545	A	C	0.092	0.022	0.004	1.9e-09
rs73005426	11	118681083	G	A	0.211	0.022	0.003	1.4e-17
rs171270	11	126207745	G	C	0.285	0.019	0.002	1.4e-15
rs10893844	11	128185850	C	G	0.502	0.026	0.002	1.2e-33
rs3016776	11	128606508	T	C	0.472	-0.017	0.002	6.2e-15
rs12820863	12	4318723	T	C	0.351	0.020	0.002	2.9e-18
rs10849448	12	6493351	G	A	0.753	-0.022	0.003	2.6e-18
rs4931002	12	32143169	A	C	0.779	-0.024	0.003	8.7e-21
rs11559982	12	54711574	G	A	0.556	0.014	0.002	3.9e-11
rs1689510	12	56396768	C	G	0.338	0.029	0.002	8.9e-39
rs3024971	12	57493727	G	T	0.107	-0.038	0.003	2.9e-28
rs11105213	12	89624206	G	A	0.139	-0.020	0.003	2.6e-10
rs4760384	12	92579100	C	T	0.171	-0.031	0.003	6.9e-28
rs3858607	12	94536803	C	G	0.378	-0.014	0.002	1.4e-10
rs140526537	12	110553366	T	C	0.020	0.057	0.008	1.4e-12
rs10774624	12	111833788	A	G	0.515	-0.080	0.002	1e-200
rs535850137	12	112018317	A	G	0.407	0.028	0.003	6.2e-25
rs78691875	12	113070264	A	C	0.021	-0.044	0.007	2.5e-09
rs12811109	12	123471094	A	G	0.199	-0.019	0.003	2.4e-12
rs12861824	13	31079184	C	T	0.273	-0.019	0.003	2.8e-13
rs12019493	13	40373190	T	C	0.600	0.014	0.002	4.1e-11
rs2065633	13	41252265	C	T	0.290	0.038	0.002	9.1e-59
rs12876545	13	43057478	G	A	0.300	0.025	0.002	4.6e-27
rs60970458	13	50424159	T	G	0.217	0.018	0.003	2e-12
rs1326125	13	50888893	C	A	0.361	0.021	0.002	2.6e-21
rs9600233	13	74693401	A	G	0.159	-0.027	0.003	5.7e-20
rs9557201	13	99989538	G	A	0.272	0.018	0.002	3.6e-14
rs912131	13	100032346	G	A	0.702	0.028	0.002	6.2e-33
rs9525192	13	114961339	G	A	0.531	-0.017	0.002	8.8e-16
rs1951121	14	23429729	G	T	0.398	-0.015	0.002	3.1e-11
rs2239633	14	23589057	A	G	0.484	0.035	0.002	4.8e-62
rs8020739	14	35882492	T	G	0.643	0.023	0.002	3.7e-24
rs61988304	14	37699544	G	A	0.287	0.016	0.002	3.2e-11
rs6573020	14	55871452	T	C	0.434	0.018	0.002	3.6e-17
rs762810	14	65544367	A	C	0.361	0.016	0.002	2.3e-12
rs11624310	14	69240079	G	A	0.564	0.014	0.002	2.9e-10
rs12882409	14	73425462	A	G	0.176	-0.018	0.003	2.7e-10
rs175705	14	75975648	G	C	0.719	0.037	0.002	6.4e-54
rs3742704	14	88477882	C	A	0.092	0.023	0.004	4.1e-10
rs67856193	14	93024616	G	C	0.308	0.020	0.002	3.6e-18
rs11555542	14	94417531	C	T	0.063	0.064	0.004	1.5e-48
rs8012557	14	103326936	C	G	0.563	-0.014	0.002	1.9e-11
rs62006172	15	38904527	A	G	0.042	-0.038	0.005	4.1e-13
rs1655558	15	41787585	G	T	0.548	0.019	0.002	3.5e-19
rs35311230	15	43633497	C	G	0.200	0.022	0.003	3.2e-16
rs11071528	15	60701797	C	G	0.807	0.019	0.003	1.9e-12
rs34986765	15	61069201	C	T	0.131	-0.023	0.003	4.4e-13
rs11638671	15	63795628	C	T	0.343	-0.015	0.002	7.9e-12
rs17293632	15	67442596	T	C	0.236	0.030	0.003	2.8e-33
rs28362902	15	74712178	A	G	0.133	0.019	0.003	4.3e-09
rs2115535	15	80195199	A	G	0.568	0.030	0.002	6.6e-44
rs1138358	15	80263345	C	A	0.264	-0.026	0.002	1.8e-27

rs13511	15	86284817	C	T	0.577	-0.019	0.002	5.1e-19
rs5011651	15	91166613	A	G	0.759	0.019	0.002	2.2e-14
rs4785903	16	2838274	C	T	0.755	-0.034	0.002	1.7e-43
rs9934736	16	3519252	G	A	0.047	0.033	0.005	1.3e-10
rs9927527	16	11212681	G	A	0.350	-0.045	0.002	1.7e-88
rs2074572	16	27356359	T	C	0.359	0.028	0.002	5.1e-36
rs8060511	16	30101596	A	C	0.480	-0.027	0.002	1.7e-35
rs8044535	16	30464034	A	T	0.568	-0.013	0.002	5.6e-10
rs72800841	16	31014247	A	G	0.250	-0.015	0.002	1.9e-09
rs9924483	16	48646258	T	C	0.310	-0.026	0.002	4.9e-30
rs9925905	16	57080866	A	T	0.473	-0.015	0.002	2.5e-12
rs73595580	16	67636767	A	G	0.083	0.027	0.004	2.4e-12
rs1170439	16	68608511	C	T	0.779	0.024	0.003	3.9e-20
rs73575076	16	79744148	G	A	0.314	-0.015	0.002	1.2e-10
rs301161	16	85810349	A	G	0.850	0.025	0.003	1.6e-17
rs12924774	16	86003804	C	G	0.352	0.018	0.002	2.5e-16
rs17175830	16	88558164	A	G	0.237	0.030	0.003	2.8e-33
rs112036266	17	2834143	T	C	0.176	0.017	0.003	5.7e-10
rs34210653	17	4535314	A	G	0.021	-0.172	0.007	6.5e-118
rs12601936	17	7172609	G	A	0.605	0.013	0.002	8.3e-10
rs74480102	17	7742601	A	G	0.043	-0.068	0.005	3.8e-38
rs1838149	17	33819302	A	G	0.423	-0.020	0.002	4e-19
rs35130019	17	37983142	G	A	0.034	-0.085	0.006	2e-48
rs2109221	17	38830056	A	G	0.578	0.015	0.002	1.6e-11
rs9906974	17	43822772	C	T	0.219	-0.038	0.003	8.4e-49
rs12952581	17	47448346	A	G	0.362	0.029	0.002	3.5e-39
rs11079348	17	56438624	T	G	0.171	0.019	0.003	4.6e-11
rs62086903	17	66016006	C	T	0.231	0.029	0.003	2.6e-30
rs180506	17	68274205	A	G	0.776	-0.024	0.003	2.2e-21
rs11077961	17	81012749	G	A	0.361	0.013	0.002	1.7e-09
rs2847286	18	12817815	G	A	0.352	-0.015	0.002	1.2e-11
rs4578769	18	20395104	T	G	0.749	0.023	0.002	1.1e-20
rs57633475	18	46469650	G	A	0.122	-0.025	0.003	3.4e-14
rs73963711	18	60874413	T	C	0.210	0.023	0.003	1.2e-17
rs954954	18	60902328	C	A	0.105	-0.034	0.003	6.1e-22
rs17758695	18	60920854	T	C	0.029	-0.104	0.006	1.8e-61
rs1395269	18	61377644	G	T	0.301	-0.023	0.002	3.8e-23
rs3848640	19	1037986	G	A	0.329	-0.021	0.002	6.4e-20
rs36084354	19	1079959	A	G	0.092	-0.034	0.004	1.3e-20
rs757230	19	1102131	G	A	0.469	-0.013	0.002	1.9e-09
rs61731111	19	3179517	T	C	0.011	-0.132	0.010	5.1e-38
rs448998	19	16411418	G	A	0.732	0.023	0.002	4e-22
rs410867	19	16427111	G	A	0.216	-0.065	0.003	3.3e-138
rs11669369	19	16654394	C	G	0.107	0.029	0.003	5.3e-17
rs117710327	19	33726578	A	C	0.067	-0.037	0.004	8.8e-18
rs4802399	19	38899999	A	G	0.043	0.045	0.005	2.4e-17
rs412884	19	40219449	C	T	0.672	0.056	0.002	2.4e-135
rs531660643	19	45251806	T	G	0.023	-0.069	0.007	6.9e-21
rs7252363	19	46354793	A	G	0.353	0.026	0.002	1.2e-30
rs11084096	19	52128795	A	G	0.296	-0.017	0.002	1.4e-12
rs11667325	19	52301063	A	G	0.663	-0.019	0.002	8.7e-18
rs6080761	20	17629162	A	G	0.419	0.015	0.002	6.2e-12
rs80054178	20	30294682	C	T	0.023	-0.080	0.007	7.3e-29
rs6141755	20	31163565	T	G	0.236	-0.016	0.003	9.7e-11
rs6031302	20	42656310	G	C	0.728	-0.020	0.002	2.5e-16
rs6126042	20	49162186	T	C	0.085	-0.037	0.004	2.3e-21

rs17728960	20	50129716	C	T	0.068	-0.029	0.004	1.3e-11
rs2223043	21	16438793	G	A	0.305	0.021	0.002	4.2e-18
rs2823327	21	16898807	T	C	0.187	0.017	0.003	4.3e-10
rs1000005	21	34433051	C	G	0.591	-0.021	0.002	3.6e-21
rs34872427	21	36294044	C	T	0.415	-0.018	0.002	2.8e-17
rs2242886	21	36387806	T	C	0.071	-0.099	0.004	1.4e-125
rs9979383	21	36715761	T	C	0.630	0.031	0.002	3.9e-44
rs150229150	21	45641853	T	C	0.125	-0.020	0.003	6.7e-10
rs1811069	22	21982054	G	T	0.191	0.018	0.003	2.7e-11
rs7288670	22	24621826	G	A	0.306	-0.019	0.002	2.8e-16
rs140074	22	31725634	G	C	0.486	0.028	0.002	1.5e-40
rs41280005	22	37316133	A	G	0.167	-0.029	0.003	5e-24
rs228951	22	37529798	G	A	0.363	-0.022	0.002	1.6e-22
rs2413631	22	41359786	A	G	0.250	0.015	0.002	3.2e-09
rs743002	22	41404939	C	T	0.066	-0.063	0.004	1.6e-48
rs78023664	22	46647844	A	G	0.092	-0.029	0.004	6.6e-15

Table S15. 115 SNPs significantly associated with basophil percentage of white cells used IVs in forward MR analyses derived from Vuckovic D et al. Chr: Chromosome; EA: Effect allele; NEA: Non-effect allele; EAF: Effect allele frequency.

SNP	Chr	Pos	EA	NEA	EAF	Beta	SE	P-value
rs35076930	1	17677636	T	C	0.293	0.021	0.003	1.6e-13
rs657624	1	20888348	A	G	0.332	-0.014	0.002	4e-09
rs12097415	1	21506276	A	T	0.547	-0.020	0.002	1.4e-19
rs10788980	1	54892472	G	A	0.447	0.014	0.002	1.4e-10
rs6679677	1	114303808	A	C	0.101	0.030	0.004	4e-16
rs11587409	1	118143444	C	G	0.163	-0.020	0.003	1.3e-11
rs12075	1	159175354	A	G	0.579	0.027	0.002	2.9e-33
rs6671847	1	161478810	A	G	0.510	0.018	0.002	3.9e-16
rs145510048	1	161605454	C	T	0.332	-0.020	0.002	4.6e-17
rs4987358	1	169665551	T	G	0.270	0.015	0.002	1.7e-09
rs12123922	1	205140436	A	G	0.441	-0.032	0.002	5e-48
rs12562618	1	212450399	G	A	0.186	-0.043	0.003	1.6e-50
rs483954	1	212620214	G	A	0.395	0.018	0.002	2.1e-15
rs28434172	1	224614625	T	C	0.208	-0.017	0.003	5.4e-10
rs2208568	1	236090155	C	T	0.893	0.112	0.004	1e-200
rs62105478	2	8735869	A	G	0.052	-0.029	0.005	4.3e-09
rs2384575	2	27387429	C	T	0.291	0.015	0.002	2.6e-09
rs75475627	2	54787592	G	C	0.076	-0.027	0.004	1.8e-10
rs12470883	2	65651851	A	G	0.409	0.014	0.002	8.2e-10
rs3755266	2	103042712	A	G	0.482	0.015	0.002	1.4e-11
rs75490288	2	145377211	A	G	0.175	-0.021	0.003	8.5e-13
rs78862952	2	146506667	C	T	0.092	0.027	0.004	3.2e-12
rs57338137	2	213857250	C	T	0.077	0.029	0.004	1.3e-12
rs4324460	3	3120542	G	T	0.219	0.018	0.003	2.4e-11
rs1669340	3	3198380	T	G	0.839	-0.025	0.003	2.6e-17
rs6766037	3	16908186	G	A	0.218	-0.040	0.003	1.5e-50
rs3181077	3	46250652	T	C	0.718	-0.044	0.002	7.8e-70
rs6782812	3	128317997	A	G	0.893	0.086	0.004	2.9e-127
rs3774298	3	187460099	A	G	0.688	-0.020	0.002	2.6e-16
rs28530750	4	36312542	A	G	0.043	-0.033	0.006	2.4e-09
rs16850073	4	74703999	T	C	0.375	-0.014	0.002	1.6e-09
rs9131	4	74963049	T	C	0.601	0.020	0.002	2.9e-19
rs72667739	4	87877499	C	A	0.168	-0.034	0.003	1.1e-29
rs10013613	4	103423493	T	G	0.402	-0.017	0.002	7e-14
rs12513896	5	10688755	C	G	0.252	0.016	0.003	1.2e-10
rs13188960	5	35853319	T	G	0.280	-0.016	0.002	7.8e-11
rs34500	5	98294885	A	G	0.084	-0.035	0.004	4e-18
rs2271352	5	126091428	C	G	0.226	0.048	0.003	1.5e-71
rs35074529	5	150838293	C	T	0.571	-0.017	0.002	1e-14
rs724745	6	22340396	G	T	0.504	0.021	0.002	6.9e-21
rs915125	6	82463376	T	C	0.279	-0.033	0.002	2.7e-41
rs72895231	6	82679396	A	C	0.596	-0.015	0.002	1.3e-10
rs9376098	6	135499460	A	T	0.349	0.031	0.002	5.6e-41
rs9389286	6	135640965	C	G	0.527	-0.013	0.002	1.7e-09
rs852444	7	5556062	G	A	0.399	-0.015	0.002	3.3e-11
rs73049252	7	8016602	A	G	0.059	-0.035	0.005	1.1e-13
rs62447197	7	50417632	G	A	0.107	0.033	0.004	2.6e-20
rs1186222	7	75247329	T	C	0.462	-0.028	0.002	4.2e-36
rs8179	7	92236164	C	T	0.792	-0.022	0.003	9.3e-16
rs445	7	92408370	T	C	0.096	-0.055	0.004	4.6e-48
rs11772895	7	143081942	C	G	0.277	0.039	0.002	1.5e-55

rs2977799	8	6696927	G	A	0.511	0.031	0.002	8.3e-44
rs2741684	8	6788418	G	C	0.693	0.019	0.002	1.4e-15
rs73523802	8	6878935	G	A	0.271	0.045	0.003	8.3e-60
rs6983894	8	6938131	A	C	0.046	-0.043	0.006	1.4e-14
rs45577137	8	48651633	G	A	0.045	-0.057	0.006	1.4e-23
rs6995515	8	61511366	C	A	0.371	-0.014	0.002	3.4e-09
rs7846314	8	61650831	T	A	0.187	-0.031	0.003	2.5e-28
rs7832357	8	126516197	G	A	0.342	-0.015	0.002	4e-11
rs4836579	9	130528548	T	C	0.573	-0.014	0.002	3.5e-10
rs11253511	10	964832	T	C	0.238	0.050	0.003	6.6e-82
rs11595895	10	26734518	A	C	0.403	0.019	0.002	8.9e-17
rs7082470	10	65277026	A	G	0.471	0.019	0.002	1.9e-17
rs55922628	10	77191441	A	C	0.611	-0.018	0.002	2e-14
rs56014906	10	102103508	G	A	0.368	-0.019	0.002	5.1e-16
rs2256368	10	114186624	A	G	0.904	-0.026	0.004	1.1e-11
rs3781452	10	126355129	T	C	0.633	0.015	0.002	1.4e-10
rs7116520	11	4053377	A	G	0.410	0.016	0.002	6.4e-12
rs74472890	11	72946279	C	T	0.049	0.121	0.005	1.7e-124
rs149709671	11	74382388	A	G	0.034	0.036	0.006	4.5e-09
rs72964941	11	88025009	T	C	0.093	0.024	0.004	7.5e-10
rs4753251	11	89654536	A	T	0.730	-0.016	0.003	5.3e-10
rs10893844	11	128185850	C	G	0.502	-0.015	0.002	4.6e-11
rs146970669	12	27103449	A	G	0.093	0.030	0.004	7.5e-15
rs117053853	12	51720047	A	G	0.011	0.113	0.011	4.3e-26
rs2118138	12	66698144	G	A	0.528	0.015	0.002	3.1e-12
rs75084335	12	76981753	G	C	0.069	0.033	0.004	6.4e-14
rs2952110	12	106661316	G	A	0.169	0.020	0.003	1.8e-11
rs4475963	12	129302255	G	T	0.370	-0.019	0.002	8.6e-17
rs149391212	14	23544999	T	C	0.016	0.068	0.009	3.7e-14
rs2239630	14	23589349	G	A	0.558	0.029	0.002	4e-38
rs11158159	14	57857162	C	G	0.773	0.029	0.003	3.4e-26
rs12878807	14	67964688	A	T	0.478	-0.013	0.002	3.5e-09
rs72721631	14	75807762	A	C	0.238	-0.021	0.003	9e-16
rs2289511	14	88454910	A	G	0.582	-0.019	0.002	1.3e-16
rs12438453	15	42678609	G	A	0.122	0.025	0.003	2.3e-13
rs2009833	15	50193553	A	G	0.360	0.015	0.002	3.4e-10
rs66871053	15	50558793	C	T	0.179	0.018	0.003	2.5e-09
rs7496362	15	65758874	G	C	0.366	-0.017	0.002	8e-14
rs2415042	15	69649385	G	C	0.627	0.017	0.002	1.5e-13
rs7166645	15	83740277	A	G	0.387	0.014	0.002	1.4e-09
rs2074585	15	91009484	A	G	0.514	-0.040	0.002	2.2e-74
rs8026614	15	100051136	T	C	0.472	-0.016	0.002	1.7e-12
rs875740	16	16123048	A	C	0.666	-0.018	0.002	8.7e-14
rs879627	16	88522541	G	C	0.437	0.016	0.002	1.9e-12
rs12941811	17	38159335	C	T	0.578	-0.013	0.002	2.1e-09
rs28730837	17	56355397	A	G	0.016	0.092	0.009	5.2e-26
rs56378716	17	56356502	G	A	0.013	0.060	0.010	1.8e-09
rs17758695	18	60920854	T	C	0.029	-0.086	0.007	2.4e-39
rs62132278	19	836654	T	C	0.256	-0.034	0.003	4.5e-37
rs2336068	19	5813519	C	A	0.276	0.016	0.003	6.1e-10
rs1609712	19	8566634	A	G	0.167	-0.033	0.003	1.2e-28
rs11554159	19	18285944	A	G	0.270	-0.017	0.002	2.8e-12
rs11879838	19	19742751	C	G	0.747	-0.022	0.003	3.3e-18
rs78744187	19	33754548	T	C	0.081	-0.087	0.004	5e-102
rs34158728	19	38903032	A	G	0.029	0.102	0.007	2.3e-53
rs453827	19	40224901	A	G	0.670	-0.020	0.002	2.8e-17

rs56827178	20	31178577	A	G	0.230	-0.017	0.003	1.2e-10
rs6141779	20	31259366	T	C	0.145	0.025	0.003	2.9e-15
rs4931	20	43530234	C	A	0.271	-0.024	0.002	1.3e-21
rs150498232	21	36792624	G	A	0.027	0.043	0.007	2.5e-09
rs11702209	21	38808428	G	C	0.118	-0.024	0.003	4.9e-12
rs2836154	21	39485842	T	C	0.336	0.021	0.002	3.8e-19
rs761702	22	41404970	A	G	0.065	0.037	0.005	2.6e-16
rs5766582	22	45632528	T	C	0.475	-0.021	0.002	6e-21

Table S16. 295 SNPs significantly associated with neutrophil percentage of white cells used IVs in forward MR analyses derived from Vuckovic D et al. Chr: Chromosome; EA: Effect allele; NEA: Non-effect allele; EAF: Effect allele frequency.

SNP	Chr	Pos	EA	NEA	EAF	Beta	SE	P-value
rs11121242	1	8906301	G	A	0.513	0.016	0.002	3.1e-14
rs66731853	1	20916238	A	G	0.325	-0.015	0.002	1.6e-10
rs4543768	1	22329645	C	T	0.321	0.016	0.002	5.5e-13
rs79567479	1	27011533	C	T	0.080	-0.037	0.004	1.9e-20
rs3917932	1	36943916	G	C	0.577	-0.038	0.002	5.2e-67
rs12718444	1	43409179	T	G	0.173	-0.019	0.003	1.6e-11
rs7537229	1	56906274	A	G	0.896	0.045	0.004	2.1e-37
rs2767481	1	66015394	C	T	0.705	0.027	0.002	5.1e-29
rs2755253	1	67470843	T	C	0.707	0.018	0.002	1.1e-13
rs41313381	1	79411968	A	C	0.031	0.052	0.006	1.5e-17
rs115340020	1	92766438	A	G	0.028	0.055	0.006	2.1e-17
rs11166538	1	101513708	G	A	0.220	-0.021	0.003	4.6e-16
rs33967958	1	101701481	C	T	0.318	0.015	0.002	3.6e-10
rs2494015	1	111734122	T	C	0.684	0.021	0.002	5e-20
rs7512697	1	150218242	T	C	0.284	-0.015	0.002	4e-10
rs6694531	1	150683512	A	C	0.514	0.033	0.002	2.1e-52
rs2479868	1	158580069	T	C	0.266	-0.021	0.002	3.1e-18
rs3026940	1	159130696	T	A	0.008	-0.120	0.014	8.1e-19
rs12131588	1	197666111	A	G	0.217	0.017	0.003	3.5e-11
rs7530375	1	212371305	G	T	0.452	-0.015	0.002	1e-11
rs6540754	1	212878652	T	A	0.664	-0.015	0.002	4.4e-11
rs5011752	1	221044762	A	G	0.675	-0.019	0.002	1e-16
rs34791963	1	224662703	C	T	0.252	0.015	0.003	2.4e-09
rs7552783	1	227174145	C	T	0.512	-0.025	0.002	3.6e-26
rs822829	1	234962529	C	A	0.879	0.024	0.003	1.6e-13
rs9970896	1	236104981	T	A	0.888	-0.027	0.003	3.1e-15
rs61838753	1	247569300	C	A	0.672	-0.014	0.002	4.5e-10
rs56188865	1	247606276	C	T	0.372	-0.016	0.002	1.9e-13
rs12105729	2	7614263	G	A	0.159	0.021	0.003	1.7e-12
rs10779936	2	23950200	A	G	0.715	-0.017	0.002	2.6e-12
rs7578575	2	25488819	A	T	0.293	-0.027	0.002	8.7e-29
rs12988231	2	25593537	C	T	0.315	-0.014	0.002	2.4e-09
rs6721395	2	27280127	A	G	0.700	-0.019	0.002	9.8e-16
rs62149448	2	40697617	C	T	0.189	0.019	0.003	1.2e-11
rs6705577	2	43359275	C	G	0.271	-0.021	0.002	1.4e-18
rs113542380	2	43464818	A	G	0.075	0.086	0.004	4.3e-96
rs62137602	2	53963242	C	A	0.184	0.020	0.003	2e-13
rs74625883	2	65663381	A	G	0.176	-0.025	0.003	3.2e-18
rs4599108	2	85543222	T	C	0.487	-0.015	0.002	1.2e-11
rs1921622	2	102966067	A	G	0.542	-0.019	0.002	3.1e-18
rs1969232	2	111597636	G	A	0.683	-0.014	0.002	6.4e-10
rs3789087	2	111791653	T	C	0.163	0.028	0.003	1.1e-21
rs55706446	2	112239082	T	C	0.075	0.041	0.004	3.5e-21
rs13409371	2	113838145	A	G	0.396	0.028	0.002	1.9e-36
rs10188023	2	136981135	A	G	0.907	0.023	0.004	6.2e-10
rs354719	2	143892139	T	A	0.825	0.026	0.003	2.2e-20
rs12611922	2	160462749	G	T	0.374	0.023	0.002	1.2e-24
rs6718526	2	161214175	C	T	0.798	-0.019	0.003	1.9e-12
rs55954384	2	182017630	G	A	0.315	0.024	0.002	2.9e-25
rs17365418	2	182173530	G	A	0.442	-0.018	0.002	4.4e-17
rs3185378	2	202151439	C	G	0.641	-0.019	0.002	4.6e-17

rs114050631	2	219020958	T	C	0.011	-0.121	0.011	3.4e-28
rs113414093	2	219859171	A	G	0.047	-0.043	0.006	6.1e-14
rs11676298	2	227291731	G	C	0.192	-0.025	0.003	1.5e-20
rs6741554	2	232272500	G	A	0.248	0.016	0.003	4.2e-10
rs2568569	2	237796392	G	A	0.739	0.015	0.002	1.8e-09
rs13066322	3	12269266	T	C	0.417	0.021	0.002	3.1e-21
rs1563656	3	28520417	A	T	0.414	-0.017	0.002	1.4e-15
rs11706384	3	39296881	T	G	0.236	-0.022	0.003	2.5e-18
rs13063578	3	47087837	A	T	0.401	-0.030	0.002	2.6e-39
rs71298382	3	71382067	G	A	0.027	-0.076	0.007	5.6e-28
rs1456117	3	123078908	T	G	0.217	0.023	0.003	5.8e-18
rs115697464	3	128175328	C	T	0.015	0.054	0.009	1.9e-09
rs76937118	3	128316589	T	C	0.050	-0.039	0.005	2.4e-14
rs6782812	3	128317997	A	G	0.893	-0.079	0.003	8.8e-114
rs143699489	3	140927121	G	A	0.127	0.022	0.003	2.7e-11
rs2871960	3	141121814	C	A	0.445	-0.017	0.002	2e-15
rs28465436	3	169489082	C	A	0.248	-0.016	0.003	4.4e-10
rs1523475	3	187444210	C	T	0.806	0.017	0.003	6.6e-10
rs789858	3	194405966	T	C	0.403	0.014	0.002	5.8e-10
rs73221129	4	729603	G	A	0.359	0.019	0.002	1.1e-17
rs6831368	4	6969919	G	A	0.362	-0.015	0.002	1.8e-11
rs28530750	4	36312542	A	G	0.043	0.038	0.005	1.7e-12
rs6531601	4	38015513	A	T	0.422	-0.013	0.002	2e-09
rs6841652	4	38363262	C	T	0.680	-0.044	0.002	3.2e-81
rs6827279	4	38566132	T	C	0.539	0.024	0.002	6.7e-26
rs218265	4	55408999	C	T	0.155	0.032	0.003	5.6e-27
rs13121174	4	55497446	A	T	0.381	-0.016	0.002	1e-12
rs16850073	4	74703999	T	C	0.375	0.045	0.002	7.4e-90
rs146149115	4	74768492	G	A	0.010	-0.085	0.011	5.5e-14
rs546829	4	74956372	T	A	0.626	-0.056	0.002	1.1e-140
rs36114828	4	80923829	A	C	0.739	0.020	0.002	1.4e-15
rs11726195	4	103538911	G	C	0.521	-0.027	0.002	4.5e-35
rs2189234	4	106075498	G	T	0.618	-0.034	0.002	4.7e-53
rs309375	4	123681156	A	C	0.572	-0.019	0.002	2e-17
rs4696314	4	153005716	C	T	0.512	-0.017	0.002	2.6e-15
rs2853677	5	1287194	A	G	0.577	-0.025	0.002	2.1e-31
rs11744391	5	33241655	A	C	0.392	-0.013	0.002	1.5e-09
rs11567701	5	35859863	T	G	0.260	-0.035	0.002	8.8e-47
rs4865744	5	52082774	G	A	0.921	0.026	0.004	9.5e-11
rs11741775	5	68590395	T	C	0.443	-0.020	0.002	2.7e-20
rs10942300	5	71745412	T	C	0.880	-0.033	0.003	6.6e-24
rs27643	5	87994702	G	T	0.721	0.020	0.002	4.7e-17
rs2927608	5	96252432	A	G	0.446	0.013	0.002	4.4e-09
rs62385260	5	131967540	A	G	0.188	-0.027	0.003	1.3e-22
rs75925066	5	134722833	A	C	0.023	-0.049	0.007	9.4e-12
rs6580229	5	141510754	G	A	0.617	0.015	0.002	3e-11
rs56330463	5	148200011	C	T	0.552	-0.035	0.002	5.4e-57
rs7720470	5	149398480	G	A	0.625	-0.018	0.002	1e-15
rs6878978	5	157950125	T	G	0.278	0.018	0.002	1.2e-13
rs12659737	5	176742506	C	T	0.840	-0.028	0.003	9.3e-22
rs61408162	5	179042400	T	C	0.357	0.017	0.002	9.4e-15
rs27474	5	179342289	C	A	0.395	0.015	0.002	3.4e-11
rs3777755	6	12159699	T	C	0.310	0.019	0.002	1.5e-15
rs2560775	6	13525365	A	G	0.692	-0.017	0.002	6.7e-14
rs1267500	6	14715825	T	C	0.810	0.021	0.003	9e-14
rs6911302	6	24820052	C	A	0.551	0.025	0.002	2.7e-31

rs3128900	6	29833057	T	G	0.845	0.046	0.003	2.8e-53
rs2071596	6	31506691	A	G	0.163	0.027	0.003	4e-20
rs116787876	6	32491139	G	A	0.422	-0.028	0.003	1.7e-26
rs2185798	6	41773576	C	G	0.479	-0.016	0.002	3e-14
rs11755487	6	42506099	A	G	0.210	0.028	0.003	1.3e-25
rs1533584	6	53221183	C	T	0.301	-0.014	0.002	2.2e-09
rs915125	6	82463376	T	C	0.279	-0.027	0.002	2.4e-29
rs9362417	6	87983690	G	A	0.490	-0.029	0.002	3e-40
rs12526696	6	108053364	A	G	0.161	-0.024	0.003	8e-16
rs12664455	6	109590739	T	C	0.293	0.016	0.002	2e-11
rs13203252	6	109672736	G	A	0.300	0.025	0.002	4.8e-26
rs9398642	6	121883200	T	C	0.144	0.018	0.003	1.7e-09
rs72959041	6	127454893	A	G	0.049	-0.033	0.005	5.3e-11
rs928722	6	137973832	T	C	0.221	0.017	0.003	4.3e-11
rs149110519	6	144385777	T	C	0.036	-0.072	0.006	2.4e-34
rs212409	6	159470058	A	G	0.553	-0.014	0.002	1.7e-10
rs1012657	6	167525142	C	A	0.473	0.013	0.002	1.4e-09
rs6796	7	6502367	C	T	0.278	-0.040	0.002	5.4e-61
rs73049270	7	8021646	T	C	0.058	-0.034	0.005	2.1e-13
rs212837	7	26695215	C	T	0.621	-0.013	0.002	2.2e-09
rs2158799	7	28277107	G	C	0.610	0.043	0.002	7.3e-83
rs56388170	7	28724374	T	G	0.292	0.055	0.002	3.7e-117
rs60600003	7	37382465	G	T	0.101	-0.025	0.004	7.6e-12
rs10259698	7	40069862	G	A	0.421	0.013	0.002	3.5e-09
rs876036	7	50307710	C	T	0.320	0.027	0.002	5.3e-32
rs149007767	7	50370254	T	C	0.162	0.044	0.003	3e-48
rs4416724	7	64982809	C	A	0.101	0.029	0.004	2.6e-15
rs42033	7	92237533	T	A	0.208	0.028	0.003	1.4e-25
rs445	7	92408370	T	C	0.096	-0.045	0.004	2.3e-34
rs6969234	7	97987986	C	T	0.493	-0.018	0.002	4.3e-17
rs6956344	7	99359151	T	C	0.086	-0.026	0.004	6.3e-12
rs62621812	7	127015083	A	G	0.020	-0.062	0.008	3.1e-15
rs12531590	7	148888461	C	A	0.624	-0.017	0.002	1e-14
rs3735080	7	150217309	T	C	0.230	-0.026	0.003	1.7e-23
rs4840639	8	6698413	A	T	0.510	0.013	0.002	6.9e-10
rs4841133	8	9183664	G	A	0.909	0.039	0.004	9.6e-25
rs1864585	8	10732905	G	A	0.742	-0.022	0.002	7.8e-19
rs34215892	8	21767240	A	G	0.028	0.044	0.007	3.4e-11
rs13272050	8	23406241	T	C	0.171	0.017	0.003	4.9e-09
rs6468341	8	30279355	C	T	0.742	0.016	0.002	1.1e-10
rs4737009	8	41630405	A	G	0.236	-0.025	0.003	1.6e-23
rs45577137	8	48651633	G	A	0.045	0.066	0.006	3.6e-32
rs7846314	8	61650831	T	A	0.187	0.067	0.003	4.8e-128
rs2953475	8	79638138	A	C	0.250	-0.031	0.002	2.1e-35
rs667927	8	103912414	T	A	0.366	-0.015	0.002	8.6e-12
rs2954027	8	126485294	A	T	0.468	0.027	0.002	1e-34
rs4871622	8	126618736	T	C	0.666	0.014	0.002	1.1e-09
rs16904123	8	130607775	T	G	0.187	0.034	0.003	5.2e-34
rs13276309	8	142338552	G	A	0.359	-0.014	0.002	3.9e-10
rs385893	9	4763176	C	T	0.524	0.026	0.002	1.4e-33
rs2066361	9	6218960	A	T	0.258	-0.017	0.002	2.7e-11
rs3731211	9	21986847	A	T	0.722	-0.027	0.002	4.7e-30
rs2065500	9	22145694	G	A	0.158	0.032	0.003	4.4e-27
rs10971930	9	34116083	C	T	0.127	-0.027	0.003	3.3e-17
rs2585668	9	38200670	C	T	0.505	0.013	0.002	1.2e-09
rs10867416	9	82324152	C	T	0.656	0.013	0.002	3.9e-09

rs61750929	9	91495135	T	C	0.056	0.060	0.005	8.4e-37
rs10980797	9	113912553	G	A	0.489	0.023	0.002	1.8e-26
rs10817483	9	116097941	A	C	0.357	-0.014	0.002	8.4e-10
rs72759286	9	126985858	C	T	0.199	-0.022	0.003	2.5e-16
rs455381	9	136905577	A	C	0.266	0.020	0.002	3.4e-16
rs3812565	9	139272502	C	T	0.397	0.020	0.002	8.6e-20
rs61839660	10	6094697	T	C	0.098	0.032	0.004	8.4e-19
rs7916672	10	13534234	T	C	0.600	0.013	0.002	1.1e-09
rs2807742	10	28781367	A	G	0.770	0.018	0.003	1.4e-12
rs2288619	10	45939818	T	C	0.070	0.027	0.004	3.1e-10
rs7084861	10	50303536	T	C	0.236	0.017	0.003	4.5e-11
rs10740054	10	63702586	A	T	0.680	0.015	0.002	6.7e-11
rs4469770	10	65013912	G	A	0.784	-0.021	0.003	4.9e-16
rs182049218	10	72332726	T	C	0.035	-0.037	0.006	4.2e-10
rs2802372	10	81047575	C	A	0.436	-0.016	0.002	8.1e-13
rs1539330	10	94502738	T	C	0.134	-0.022	0.003	3.3e-11
rs10882881	10	99010067	A	T	0.600	0.020	0.002	1.8e-19
rs7912643	10	112027936	G	A	0.358	-0.013	0.002	3e-09
rs10885079	10	112797803	A	G	0.704	0.015	0.002	3.4e-10
rs11198861	10	121049819	A	G	0.512	-0.015	0.002	2e-11
rs11245331	10	126382353	A	G	0.110	0.032	0.003	1.2e-20
rs11603142	11	300228	G	C	0.342	0.014	0.002	2.4e-09
rs14408	11	308314	C	T	0.368	0.037	0.002	6.2e-59
rs11599958	11	10358095	T	C	0.289	-0.014	0.002	3.8e-09
rs11033388	11	36074218	T	A	0.418	-0.014	0.002	5.4e-11
rs11039216	11	47406592	T	C	0.533	-0.013	0.002	3.3e-09
rs7396753	11	60021508	C	T	0.592	0.016	0.002	3.2e-13
rs61897795	11	61618169	G	A	0.164	-0.022	0.003	1.7e-14
rs7927894	11	76301316	T	C	0.392	-0.014	0.002	9.3e-11
rs72996108	11	100452869	A	C	0.097	0.023	0.004	3.6e-10
rs2606724	11	113957880	A	G	0.455	0.019	0.002	2.4e-17
rs11243	11	119178640	G	A	0.045	0.033	0.005	5.1e-10
rs58432776	11	122514281	A	C	0.380	-0.032	0.002	4.4e-46
rs10790962	11	128385169	A	G	0.469	-0.019	0.002	2.6e-17
rs34038797	12	740009	G	C	0.479	0.015	0.002	3e-12
rs2364482	12	6502131	G	T	0.197	-0.035	0.003	1.4e-37
rs117266223	12	10543436	T	A	0.075	-0.035	0.004	7.4e-17
rs11045886	12	21386493	C	A	0.166	0.020	0.003	1.4e-11
rs113736796	12	48213720	G	C	0.039	0.047	0.006	2.9e-16
rs1793937	12	48375475	G	C	0.401	-0.015	0.002	3.4e-12
rs190801344	12	53735778	G	C	0.019	-0.051	0.008	3.2e-10
rs4761234	12	69732105	C	T	0.485	0.024	0.002	2.1e-29
rs4842610	12	88811689	T	C	0.705	-0.014	0.002	1.2e-09
rs35700167	12	108165223	T	G	0.347	-0.014	0.002	3.8e-10
rs653178	12	112007756	T	C	0.518	0.050	0.002	1.2e-120
rs28413626	12	123861452	A	G	0.214	-0.026	0.003	1.8e-22
rs12428172	13	28645757	G	A	0.544	0.013	0.002	2.4e-09
rs9549201	13	40977348	C	T	0.634	0.015	0.002	1.2e-11
rs9532679	13	41522338	C	A	0.150	0.032	0.003	4.9e-25
rs2044732	13	42833400	G	A	0.214	0.022	0.003	1.8e-16
rs1570621	13	47170118	C	T	0.848	0.019	0.003	9.6e-10
rs9530281	13	74679161	A	G	0.716	0.022	0.002	1.5e-19
rs2997995	13	114160506	T	C	0.295	0.018	0.002	1.1e-13
rs7981577	13	114835802	C	T	0.437	0.014	0.002	2.7e-11
rs2239630	14	23589349	G	A	0.558	-0.021	0.002	1.4e-21
rs2038700	14	25461989	C	T	0.394	0.017	0.002	4.5e-15

rs17091786	14	35565856	T	A	0.200	-0.021	0.003	1.9e-14
rs73267743	14	64978142	T	G	0.230	-0.021	0.003	1.1e-15
rs34765661	14	69852940	C	T	0.065	0.040	0.004	7.5e-20
rs175702	14	75971133	C	A	0.532	-0.018	0.002	8.6e-17
rs10146346	14	93083572	A	G	0.408	-0.013	0.002	1.2e-09
rs8008502	14	103359492	A	G	0.202	-0.024	0.003	1.6e-18
rs4990175	14	103838509	A	G	0.534	-0.014	0.002	3.6e-10
rs10851372	15	39246754	G	A	0.409	-0.013	0.002	1.2e-09
rs12440045	15	41782684	C	A	0.529	-0.018	0.002	4.7e-17
rs4924590	15	42233673	A	T	0.244	0.020	0.003	2.3e-15
rs28885693	15	51278413	A	G	0.459	-0.013	0.002	1.3e-09
rs28727558	15	66016711	A	T	0.170	0.024	0.003	1.5e-16
rs4480762	15	75139426	G	A	0.581	0.020	0.002	1.7e-19
rs168108	15	86183873	G	C	0.651	0.016	0.002	6.2e-13
rs28539372	15	91021412	A	T	0.319	-0.025	0.002	3.2e-27
rs67175901	15	101748227	T	C	0.108	0.039	0.003	6.1e-29
rs12935657	16	11219041	A	G	0.246	0.017	0.003	3.1e-11
rs61739285	16	27480797	T	C	0.034	-0.035	0.006	2.9e-09
rs11150589	16	30482494	C	T	0.517	-0.023	0.002	2.3e-25
rs74250734	16	50212589	G	T	0.095	-0.023	0.004	3.2e-10
rs36026517	16	74597758	C	G	0.198	0.022	0.003	1e-16
rs391855	16	85928621	T	A	0.423	0.043	0.002	6.1e-86
rs305064	16	85974142	C	T	0.648	-0.022	0.002	2.4e-22
rs7202624	16	86016175	C	G	0.811	-0.023	0.003	8.2e-17
rs9905106	17	1373518	C	T	0.735	0.020	0.002	8e-17
rs9674744	17	2729980	T	C	0.277	-0.016	0.002	1.7e-10
rs75921867	17	7241277	T	C	0.163	0.019	0.003	8.8e-10
rs9910148	17	16169009	A	G	0.476	-0.038	0.002	5.7e-70
rs8070995	17	17167492	T	C	0.137	-0.020	0.003	3.2e-10
rs113865680	17	29278821	C	T	0.204	-0.020	0.003	1.1e-12
rs4580194	17	38126477	G	A	0.435	0.071	0.002	1e-200
rs11654706	17	38147293	G	A	0.168	-0.021	0.003	4.2e-13
rs8067341	17	41079374	C	T	0.476	-0.017	0.002	2.8e-14
rs71375338	17	44332793	A	G	0.269	0.028	0.002	1.3e-30
rs8178414	17	56345363	T	C	0.013	0.100	0.010	2e-25
rs34097845	17	56358429	T	C	0.057	0.054	0.005	1.9e-29
rs8079549	17	73066982	T	G	0.458	0.015	0.002	1.1e-11
rs4789294	17	74419177	G	A	0.217	-0.020	0.003	5.7e-15
rs4969405	17	79122996	T	G	0.420	0.014	0.002	7.2e-11
rs7231555	18	19699122	G	A	0.088	0.026	0.004	2.8e-11
rs75763843	18	42062380	C	A	0.129	0.031	0.003	1.2e-21
rs66836460	18	60837508	A	C	0.136	-0.023	0.003	8e-13
rs3177609	18	74071078	C	T	0.077	-0.028	0.004	2.6e-12
rs454854	18	77253866	C	G	0.679	0.015	0.002	4.5e-10
rs35140707	19	1078297	T	C	0.100	0.038	0.004	2.5e-25
rs6510834	19	5008673	C	T	0.657	0.025	0.002	1.2e-27
rs35251378	19	10459969	A	G	0.293	-0.028	0.002	1.6e-32
rs345627	19	13917264	T	C	0.349	-0.021	0.002	7.5e-21
rs10423726	19	16444697	A	C	0.580	0.033	0.002	3.9e-48
rs7251806	19	16499569	T	C	0.841	0.065	0.003	1.1e-108
rs79355165	19	18522031	G	A	0.120	0.027	0.003	2.2e-16
rs867616	19	19757682	T	C	0.177	-0.018	0.003	3.7e-10
rs12151289	19	33751852	C	G	0.030	0.059	0.006	8e-20
rs56898944	19	36662925	A	T	0.114	0.022	0.004	2.8e-09
rs4760	19	44153100	G	A	0.156	-0.050	0.003	7.5e-63
rs349048	19	44300937	T	C	0.476	0.013	0.002	8.6e-10

rs7255933	19	45766729	A	G	0.258	-0.015	0.002	1.7e-09
rs466477	19	47679798	C	T	0.743	0.018	0.002	4e-13
rs8102492	19	49140809	C	A	0.796	0.034	0.003	2.4e-36
rs4630833	20	1930798	C	T	0.263	0.032	0.002	5.3e-38
rs8114815	20	3757520	C	T	0.267	-0.018	0.002	2.5e-13
rs7261425	20	20068635	G	C	0.279	-0.018	0.002	2.8e-13
rs8114375	20	31196471	G	A	0.394	-0.015	0.002	1e-10
rs6071837	20	38496424	G	A	0.247	-0.015	0.003	1.7e-09
rs112111275	20	39198317	A	G	0.064	-0.029	0.004	1e-10
rs2143606	20	42838550	G	A	0.455	-0.022	0.002	9.9e-24
rs259967	20	57816060	C	T	0.528	0.025	0.002	1.5e-29
rs6011066	20	62364180	A	G	0.674	0.018	0.002	1.4e-14
rs1736148	21	16813212	C	T	0.430	-0.013	0.002	1e-09
rs2836884	21	40467643	C	T	0.276	-0.017	0.002	9.4e-12
rs5747308	22	18133500	C	A	0.505	0.016	0.002	3.6e-13
rs5754104	22	21916361	A	G	0.187	0.026	0.003	4.3e-21
rs118839	22	28380770	C	T	0.482	0.014	0.002	1.4e-10
rs4823076	22	30519414	A	G	0.606	-0.022	0.002	1.6e-23
rs5757886	22	40636076	C	T	0.748	0.018	0.002	1.2e-13
rs5758307	22	41750219	G	A	0.229	-0.026	0.003	3.4e-23

Table S17. 371 SNPs significantly associated with monocyte percentage of white cells used IVs in forward MR analyses derived from Vuckovic D et al. Chr: Chromosome; EA: Effect allele; NEA: Non-effect allele; EAF: Effect allele frequency.

SNP	Chr	Pos	EA	NEA	EAF	Beta	SE	P-value
rs67289919	1	2954208	A	G	0.183	0.017	0.003	9.3e-10
rs12124851	1	8903931	G	A	0.316	0.016	0.002	1.2e-11
rs79412885	1	9241839	A	G	0.073	0.046	0.004	6.7e-29
rs6703602	1	10378451	G	C	0.127	0.025	0.003	9.2e-15
rs2501299	1	22345647	T	C	0.661	0.019	0.002	5.8e-17
rs12140788	1	23659214	A	C	0.182	-0.022	0.003	7.5e-16
rs7416513	1	26647949	C	G	0.762	0.034	0.002	7.5e-44
rs78169804	1	27155747	T	G	0.081	0.038	0.004	1.4e-22
rs3917932	1	36943916	G	C	0.577	0.025	0.002	6e-31
rs11800162	1	36970512	T	C	0.020	-0.052	0.008	1.6e-11
rs11209092	1	41346344	C	T	0.599	-0.015	0.002	3.8e-12
rs11206004	1	53210958	A	G	0.470	-0.013	0.002	1.2e-09
rs7516441	1	60140247	A	G	0.124	0.027	0.003	3.8e-16
rs11579758	1	65445542	A	G	0.757	0.017	0.002	3.7e-12
rs1892534	1	66105944	T	C	0.375	0.021	0.002	3.8e-22
rs17391694	1	78623626	T	C	0.138	-0.018	0.003	4.6e-09
rs12118443	1	90038588	A	G	0.489	-0.015	0.002	3.6e-12
rs17575497	1	92315896	T	C	0.013	0.365	0.010	1e-200
rs143705965	1	93055538	T	C	0.029	-0.125	0.006	1.5e-84
rs4847398	1	93578402	T	C	0.620	0.018	0.002	3.3e-17
rs11166469	1	100991634	C	G	0.756	0.017	0.002	5.2e-12
rs663045	1	108743059	C	G	0.534	-0.018	0.002	1.1e-16
rs11102144	1	111195493	G	A	0.169	-0.023	0.003	5e-16
rs11464707	1	118161020	G	T	0.164	0.027	0.003	3.6e-21
rs11204682	1	150595537	T	G	0.223	-0.064	0.003	1e-140
rs3014880	1	153344374	G	C	0.116	0.038	0.003	7.3e-31
rs12741203	1	161139951	T	C	0.300	0.024	0.002	8.7e-25
rs5030738	1	161599654	T	G	0.018	-0.064	0.008	1.3e-15
rs12725126	1	179427632	A	G	0.188	-0.016	0.003	4.1e-09
rs2281415	1	185392845	T	C	0.321	-0.023	0.002	3.4e-23
rs79273924	1	198639132	T	C	0.044	-0.033	0.005	5.8e-10
rs11582805	1	205653056	T	C	0.460	0.019	0.002	3.1e-19
rs1663626	1	212422567	A	C	0.399	0.026	0.002	8e-34
rs2738752	1	221052765	G	A	0.331	-0.027	0.002	4.6e-33
rs113292043	1	223231657	T	C	0.067	-0.028	0.004	3e-11
rs34791963	1	224662703	C	T	0.252	-0.016	0.002	1e-10
rs12593	1	227172290	T	C	0.465	0.017	0.002	3.2e-13
rs72749964	1	234740975	A	G	0.189	0.023	0.003	6.5e-17
rs4626924	1	234909298	T	C	0.551	-0.036	0.002	1.7e-63
rs6429432	1	236107241	C	A	0.892	-0.053	0.003	5.8e-56
rs113566032	1	247495385	C	T	0.376	0.021	0.002	2e-21
rs3811444	1	248039451	T	C	0.334	-0.015	0.002	5.6e-12
rs186975	2	8442736	C	G	0.394	0.017	0.002	1.7e-15
rs138730630	2	24250088	A	G	0.133	-0.038	0.003	4.6e-34
rs56180058	2	25337155	T	C	0.160	0.018	0.003	1.4e-09
rs1260326	2	27730940	C	T	0.606	0.040	0.002	5e-78
rs6715862	2	42328575	G	A	0.505	0.013	0.002	1.9e-10
rs28498283	2	43360065	T	A	0.256	0.029	0.002	1.5e-31
rs6750285	2	64920032	A	G	0.244	0.024	0.002	3.7e-22
rs79709502	2	65087232	G	C	0.222	0.015	0.003	3e-09
rs11694714	2	65663936	T	C	0.328	-0.019	0.002	1.2e-17

rs72844410	2	85723006	C	T	0.204	0.018	0.003	2.3e-11
rs7593080	2	86145787	T	C	0.572	0.016	0.002	6.5e-14
rs62153700	2	96581338	T	C	0.305	0.018	0.002	8.3e-15
rs3789098	2	111738371	A	G	0.159	-0.048	0.003	1.2e-60
rs150449635	2	111752151	C	T	0.022	0.148	0.008	1.6e-86
rs140796214	2	111861072	T	A	0.017	0.071	0.008	6.4e-17
rs72836346	2	111876613	C	G	0.078	0.051	0.004	4.5e-37
rs7604081	2	127866535	C	T	0.056	-0.036	0.005	4.8e-15
rs7604460	2	136773638	C	T	0.115	-0.023	0.003	5.9e-12
rs2056295	2	136886897	G	A	0.737	-0.044	0.002	1.2e-74
rs146048066	2	145277924	G	A	0.036	0.040	0.006	2.4e-10
rs13000983	2	148561383	C	T	0.295	0.017	0.002	6.3e-13
rs10185155	2	160553228	A	G	0.374	-0.032	0.002	4.6e-50
rs4664330	2	161333128	C	G	0.316	-0.020	0.002	1e-18
rs13023380	2	163154363	A	G	0.518	0.014	0.002	2.8e-11
rs11680095	2	181825956	T	C	0.593	0.026	0.002	1.9e-32
rs6740847	2	182308352	G	A	0.564	0.114	0.002	1e-200
rs12693280	2	182424718	G	A	0.689	-0.022	0.002	3.3e-22
rs190303734	2	203491226	T	A	0.295	0.019	0.002	1.3e-16
rs114050631	2	219020958	T	C	0.011	0.117	0.011	1e-27
rs10206184	2	225748532	A	T	0.157	-0.035	0.003	1.4e-32
rs6742228	2	232267116	G	A	0.327	-0.013	0.002	3e-09
rs11693819	2	232566206	A	G	0.186	0.018	0.003	2.6e-11
rs3749171	2	241569692	T	C	0.182	-0.032	0.003	1.5e-31
rs1584063	3	12266855	A	G	0.456	-0.014	0.002	3.2e-11
rs11721007	3	14260417	A	G	0.335	0.020	0.002	4.5e-18
rs12485694	3	24346109	G	A	0.583	-0.017	0.002	7.6e-15
rs10510607	3	28286261	T	C	0.174	-0.017	0.003	4.1e-10
rs73058593	3	39281374	C	G	0.217	0.033	0.003	4.1e-37
rs2228467	3	42906116	C	T	0.061	0.149	0.004	1e-200
rs34847985	3	45949753	G	A	0.260	-0.020	0.002	1.2e-15
rs2213290	3	46406367	T	C	0.409	0.046	0.002	1.4e-96
rs9853113	3	57397738	A	T	0.462	-0.015	0.002	2.4e-12
rs13079034	3	71813219	T	C	0.112	-0.027	0.003	1.7e-15
rs55890339	3	72386266	T	C	0.222	0.024	0.003	4e-20
rs73133996	3	98429219	C	G	0.440	0.015	0.002	7.9e-13
rs1456117	3	123078908	T	G	0.217	-0.017	0.003	3.4e-11
rs2939820	3	128127643	A	G	0.294	-0.016	0.002	8e-12
rs72983394	3	128226451	C	T	0.023	-0.068	0.007	1.1e-21
rs2712421	3	128287743	T	G	0.654	0.069	0.002	1e-200
rs116026278	3	128323904	T	C	0.014	0.064	0.009	4.1e-13
rs9828891	3	140967436	T	C	0.568	0.013	0.002	6.5e-10
rs11916906	3	141314133	T	A	0.401	-0.029	0.002	1.1e-39
rs115379740	3	152031848	C	T	0.021	0.044	0.007	4e-09
rs9290357	3	168819643	G	T	0.889	0.021	0.003	7.5e-10
rs12490581	3	169565030	A	G	0.318	0.014	0.002	8.9e-10
rs79287178	3	172294500	A	G	0.031	-0.057	0.006	4.7e-19
rs939334	3	183749731	C	T	0.328	-0.019	0.002	1.4e-16
rs12485444	3	188135783	A	T	0.545	-0.029	0.002	1.1e-40
rs114503621	4	730272	C	T	0.314	-0.028	0.002	1e-33
rs871134	4	7044380	T	C	0.574	0.029	0.002	2.9e-41
rs79804860	4	26306658	T	C	0.049	0.030	0.005	1.4e-09
rs28478131	4	38039008	C	A	0.314	-0.013	0.002	3.4e-09
rs10213458	4	38366390	G	A	0.682	-0.018	0.002	4.7e-15
rs7686818	4	38682612	G	A	0.809	-0.019	0.003	4.1e-12
rs2711981	4	39039258	T	C	0.663	-0.018	0.002	5.7e-15

rs16850073	4	74703999	T	C	0.375	-0.027	0.002	6.6e-34
rs518867	4	74955050	C	T	0.623	0.036	0.002	1.1e-59
rs4693593	4	84143528	C	T	0.354	-0.028	0.002	2.6e-35
rs34333163	4	103283117	G	A	0.071	-0.027	0.004	4.5e-11
rs11723120	4	103502551	A	G	0.375	-0.025	0.002	3.7e-29
rs62343137	4	145885961	C	G	0.371	-0.016	0.002	4.4e-13
rs10069690	5	1279790	T	C	0.258	-0.016	0.002	6.9e-11
rs11960511	5	35883241	G	A	0.259	-0.016	0.002	1.3e-10
rs28722705	5	55453942	T	A	0.148	0.031	0.003	9.4e-26
rs11741775	5	68590395	T	C	0.443	0.013	0.002	9.5e-10
rs6865582	5	78201833	G	C	0.462	-0.014	0.002	6.8e-11
rs73187140	5	90320859	C	T	0.036	0.035	0.006	1.6e-09
rs327806	5	98280018	T	G	0.083	-0.026	0.004	3.2e-11
rs12521723	5	132273808	T	A	0.163	-0.020	0.003	9.6e-12
rs620498	5	133869270	G	A	0.160	-0.024	0.003	7.3e-17
rs2082382	5	148200553	A	G	0.548	0.026	0.002	3.6e-33
rs6579771	5	149482262	T	C	0.267	0.035	0.002	4.8e-49
rs193549	5	149500236	T	C	0.323	-0.054	0.002	4.6e-123
rs1081071	5	158381663	C	T	0.390	-0.015	0.002	8.1e-12
rs56998314	5	179242418	G	A	0.438	0.026	0.002	3.5e-34
rs12203592	6	396321	T	C	0.220	-0.017	0.003	1.3e-10
rs3748059	6	6900951	G	A	0.799	-0.016	0.003	1e-09
rs509227	6	10535591	C	A	0.522	-0.022	0.002	3.2e-25
rs6906830	6	16711060	A	C	0.380	-0.014	0.002	1e-10
rs9264400	6	31229413	T	C	0.808	-0.061	0.003	6.4e-115
rs11751074	6	31518157	A	G	0.033	0.053	0.006	3.1e-19
rs140932965	6	31561913	T	C	0.011	-0.069	0.011	1.2e-10
rs190452998	6	31606528	A	G	0.020	-0.113	0.011	2.8e-26
rs28746843	6	32634137	C	A	0.240	-0.035	0.003	1.2e-35
rs45482297	6	35265535	A	G	0.030	0.063	0.006	8.8e-24
rs72664259	6	41986269	G	A	0.244	0.041	0.002	1.9e-61
rs10948036	6	42510305	A	C	0.210	-0.028	0.003	9.8e-27
rs79894332	6	44591721	A	G	0.101	-0.045	0.004	1.8e-37
rs809942	6	53443269	A	G	0.198	-0.021	0.003	1.9e-15
rs915125	6	82463376	T	C	0.279	0.051	0.002	2.4e-102
rs4706287	6	87806496	G	A	0.571	0.021	0.002	5.4e-22
rs62414606	6	89836176	A	G	0.304	-0.015	0.002	5.2e-11
rs60066732	6	90935383	G	A	0.362	0.027	0.002	2e-34
rs2783019	6	107422239	C	G	0.336	-0.020	0.002	5.5e-19
rs77791617	6	114083374	T	C	0.060	0.039	0.004	3.6e-18
rs12190916	6	131045693	A	C	0.153	-0.023	0.003	7.6e-15
rs12525521	6	144371265	A	G	0.095	-0.027	0.004	7.9e-14
rs149110519	6	144385777	T	C	0.036	0.107	0.006	1.6e-76
rs2292334	6	160858188	A	G	0.390	-0.015	0.002	1e-11
rs2273215	6	170586082	A	G	0.459	-0.013	0.002	3.5e-09
rs7786990	7	5499748	G	T	0.207	0.016	0.003	7.5e-10
rs6796	7	6502367	C	T	0.278	0.050	0.002	4e-100
rs56195338	7	8107922	A	G	0.058	0.042	0.005	4.5e-20
rs10259620	7	27202289	G	A	0.787	0.017	0.003	7e-11
rs498475	7	28256240	A	G	0.632	-0.018	0.002	1.2e-15
rs2158799	7	28277107	G	C	0.610	-0.019	0.002	7.8e-19
rs56388170	7	28724374	T	G	0.292	-0.039	0.002	1.2e-62
rs73109480	7	44811221	T	C	0.071	0.029	0.004	4.9e-12
rs1870027	7	50258234	A	G	0.312	-0.079	0.002	1e-200
rs149007767	7	50370254	T	C	0.162	-0.092	0.003	1e-200
rs11766800	7	50435617	T	A	0.322	0.026	0.002	4.1e-31

rs41430449	7	50798525	G	C	0.067	-0.034	0.004	1.7e-15
rs2690418	7	65181112	C	G	0.544	-0.014	0.002	2.4e-11
rs33951980	7	73029437	T	C	0.130	0.038	0.003	3.3e-33
rs2282979	7	92264993	C	T	0.269	-0.041	0.002	2.8e-66
rs12532293	7	106886032	C	T	0.518	0.015	0.002	4.4e-12
rs3778754	7	128575552	G	C	0.467	0.013	0.002	1.7e-09
rs8180748	7	134740199	A	C	0.485	-0.014	0.002	1.2e-10
rs7785014	7	137881690	C	T	0.672	-0.026	0.002	9.5e-31
rs9640381	7	143082027	C	T	0.488	0.014	0.002	1.2e-11
rs4840638	8	6698248	G	T	0.536	-0.033	0.002	6.8e-53
rs17078436	8	6827165	C	T	0.016	0.100	0.009	2e-31
rs7826487	8	6880925	G	A	0.116	-0.061	0.003	8.3e-76
rs6988319	8	6915673	G	C	0.608	0.021	0.002	1.7e-21
rs11774798	8	21758823	G	C	0.222	0.020	0.003	7.6e-15
rs72607398	8	22941873	G	C	0.200	0.023	0.003	3.1e-18
rs55650056	8	23080829	G	C	0.185	0.017	0.003	5.9e-10
rs7007639	8	38820363	T	C	0.447	-0.013	0.002	4.5e-09
rs145718079	8	48641192	A	G	0.010	-0.097	0.011	8.1e-18
rs45577137	8	48651633	G	A	0.045	-0.082	0.005	1.8e-50
rs2719233	8	56886156	G	A	0.193	0.020	0.003	9.1e-14
rs10100873	8	59464803	G	T	0.305	0.014	0.002	6.7e-10
rs7846314	8	61650831	T	A	0.187	-0.049	0.003	1.4e-73
rs6990105	8	79601887	C	T	0.814	0.020	0.003	2.4e-13
rs10103921	8	82041714	G	A	0.419	-0.017	0.002	3.1e-15
rs6469603	8	116596188	T	C	0.563	-0.033	0.002	7.5e-54
rs28393360	8	126619477	A	T	0.477	-0.019	0.002	4.7e-19
rs10094748	8	130454047	C	G	0.084	-0.026	0.004	1.4e-11
rs7843207	8	130570063	A	C	0.506	-0.065	0.002	1e-200
rs837224	8	130971985	A	G	0.770	-0.016	0.003	1.2e-10
rs11136301	8	144650510	A	G	0.346	-0.013	0.002	2.5e-09
rs4741775	9	307266	G	A	0.702	0.021	0.002	4.2e-20
rs12005199	9	4763491	A	G	0.311	-0.017	0.002	2.4e-12
rs1887428	9	4984530	C	G	0.625	0.015	0.002	4.1e-11
rs3731246	9	21971989	G	C	0.105	-0.024	0.003	1.9e-12
rs10965258	9	22153663	G	A	0.073	0.043	0.004	1.2e-25
rs11557154	9	34107505	T	C	0.127	0.032	0.003	2.8e-23
rs10780209	9	91472127	A	G	0.477	0.040	0.002	2.3e-81
rs7029413	9	91558264	A	G	0.322	-0.019	0.002	3.6e-17
rs34374605	9	113338631	A	G	0.050	-0.033	0.005	2.7e-11
rs118012336	9	113731641	C	T	0.013	0.064	0.010	2.5e-11
rs10126013	9	113838318	A	G	0.596	0.068	0.002	1e-200
rs1930303	9	114071879	A	G	0.663	-0.061	0.002	2.7e-160
rs75943501	9	114162372	G	A	0.052	0.056	0.005	1.4e-31
rs4979025	9	114421035	G	T	0.559	0.022	0.002	1.1e-24
rs72759286	9	126985858	C	T	0.199	0.044	0.003	5.8e-62
rs10739694	9	130526605	G	A	0.588	-0.017	0.002	5.2e-15
rs12216891	9	136127366	T	C	0.059	-0.037	0.005	3.4e-16
rs1038193	9	137855206	G	C	0.264	-0.016	0.002	2.9e-11
rs3812591	9	139341612	C	T	0.275	-0.026	0.002	6.8e-29
rs71477838	10	3827920	T	C	0.148	-0.019	0.003	1.7e-10
rs11598318	10	7544309	A	C	0.051	-0.030	0.005	1.4e-09
rs2646424	10	8463115	C	G	0.326	-0.026	0.002	1.3e-29
rs2094285	10	22418702	A	G	0.225	0.023	0.003	4.5e-20
rs2807740	10	28784483	T	C	0.769	-0.019	0.003	6.9e-14
rs1184089	10	61645590	G	A	0.673	-0.017	0.002	2e-14
rs4595427	10	65012944	C	A	0.472	0.020	0.002	2.7e-21

rs3747866	10	73521578	A	G	0.061	0.028	0.004	4.5e-10
rs10824722	10	80905542	T	C	0.246	0.021	0.003	8.7e-17
rs113452214	10	89786760	T	C	0.026	0.040	0.007	1.7e-09
rs370527799	10	94036630	A	T	0.304	0.014	0.002	3.4e-09
rs11189155	10	99108947	A	G	0.257	0.031	0.002	1.6e-37
rs11817777	10	101264592	T	C	0.107	0.029	0.003	1.6e-17
rs11190141	10	101292390	T	C	0.372	-0.038	0.002	1.3e-68
rs10885396	10	114711755	T	C	0.541	-0.019	0.002	1.8e-19
rs41287142	10	121353054	T	C	0.023	-0.044	0.007	5.7e-10
rs3781454	10	126348565	A	G	0.681	-0.019	0.002	4.1e-17
rs7115703	11	306920	A	T	0.513	-0.053	0.002	6.8e-139
rs907612	11	1874221	T	C	0.380	-0.029	0.002	5.2e-39
rs2633082	11	47248050	G	C	0.808	-0.027	0.003	2.7e-24
rs11246680	11	51584437	A	G	0.947	0.032	0.005	3.1e-10
rs1373064	11	60168200	T	C	0.637	0.016	0.002	1.7e-12
rs28456	11	61589481	G	A	0.314	0.027	0.002	8.7e-32
rs74472890	11	72946279	C	T	0.049	-0.040	0.005	2.5e-16
rs2606724	11	113957880	A	G	0.455	-0.033	0.002	4.9e-51
rs77669868	11	114040394	G	A	0.170	-0.026	0.003	1.1e-19
rs6589695	11	118644323	G	C	0.957	0.036	0.005	4.8e-12
rs6589940	11	122518609	G	A	0.387	0.024	0.002	7.1e-29
rs548116	11	128538015	C	T	0.190	0.022	0.003	3.2e-16
rs2159599	12	710441	G	A	0.258	0.016	0.003	1.6e-10
rs10849448	12	6493351	G	A	0.753	-0.063	0.003	5.8e-138
rs10772280	12	10581040	A	C	0.875	-0.046	0.003	1.2e-45
rs4149083	12	21380630	T	A	0.161	-0.019	0.003	1.5e-11
rs4082413	12	30783184	G	C	0.511	0.018	0.002	5.8e-17
rs190801344	12	53735778	G	C	0.019	0.080	0.008	5.4e-24
rs11170652	12	54099076	A	G	0.194	-0.022	0.003	3.4e-16
rs35979828	12	54685880	T	C	0.070	-0.048	0.004	1.6e-30
rs1800973	12	69744014	A	C	0.061	0.115	0.004	2.1e-149
rs8181612	12	89904698	A	T	0.707	0.014	0.002	4.2e-09
rs4964677	12	108720446	C	T	0.389	0.032	0.002	6e-48
rs7314538	12	110050110	G	A	0.441	0.013	0.002	2.7e-09
rs10849930	12	111739236	C	T	0.161	0.018	0.003	2.8e-10
rs11065286	12	121271734	C	T	0.555	0.015	0.002	2.9e-12
rs7485656	12	125315647	G	A	0.141	0.032	0.003	6.7e-26
rs7326855	13	28555260	A	G	0.180	-0.020	0.003	9.4e-13
rs188175496	13	28595940	T	C	0.024	-0.075	0.008	1.9e-23
rs76428106	13	28604007	C	T	0.013	0.483	0.010	1e-200
rs17086242	13	28611221	C	T	0.041	-0.057	0.005	1.9e-26
rs138028125	13	28712689	G	C	0.035	0.109	0.006	4.5e-74
rs1892548	13	41002641	C	T	0.657	-0.064	0.002	2.2e-175
rs9532580	13	41244260	C	T	0.264	-0.036	0.002	6.9e-51
rs809845	13	50724055	T	C	0.443	0.015	0.002	5e-13
rs73217470	13	72513420	G	A	0.047	0.070	0.005	2.1e-43
rs111371822	13	92005134	G	A	0.117	0.030	0.003	5.2e-19
rs9521345	13	110039905	A	G	0.354	-0.026	0.002	6.7e-32
rs76760541	13	114191214	G	A	0.169	-0.020	0.003	4.8e-12
rs6560956	13	114816003	A	G	0.414	0.014	0.002	2.1e-11
rs2239630	14	23589349	G	A	0.558	0.051	0.002	5.1e-126
rs2038700	14	25461989	C	T	0.394	0.017	0.002	4.7e-15
rs8015119	14	35568788	T	C	0.109	-0.032	0.003	1.9e-21
rs7146939	14	65583581	C	T	0.581	0.013	0.002	9.7e-10
rs150362244	14	69884941	G	A	0.065	-0.035	0.004	3.1e-16
rs4903311	14	75827841	T	C	0.621	-0.015	0.002	2.8e-12

rs8016326	14	103846716	A	G	0.737	0.055	0.002	3.2e-113
rs55633823	14	105954705	T	C	0.251	0.016	0.002	2.3e-10
rs484943	15	40398754	C	T	0.662	0.022	0.002	3.9e-21
rs149698681	15	42651900	C	G	0.021	0.103	0.008	8.6e-42
rs150844304	15	43726625	C	A	0.025	-0.059	0.007	4.9e-18
rs3743044	15	50773787	G	A	0.028	0.046	0.006	1.7e-12
rs1533378	15	64646777	T	C	0.879	0.039	0.003	2.2e-32
rs12594547	15	65784751	A	G	0.185	-0.035	0.003	1.6e-37
rs3765066	15	75140854	A	G	0.672	-0.022	0.002	2.5e-22
rs11854390	15	80224817	T	C	0.564	0.038	0.002	1.6e-69
rs7180804	15	80259074	A	G	0.258	-0.078	0.002	1e-200
rs338555	15	86236101	A	G	0.636	-0.020	0.002	2.4e-20
rs11856737	15	99094203	G	C	0.183	0.023	0.003	2.6e-17
rs3784526	15	101716797	A	G	0.107	-0.044	0.003	6.8e-37
rs8047143	16	1349659	G	T	0.277	-0.015	0.002	3.1e-10
rs12599911	16	4062436	T	G	0.567	-0.019	0.002	2.8e-19
rs8049116	16	4150530	T	C	0.117	0.028	0.003	5.1e-17
rs12325238	16	10981518	A	C	0.265	0.019	0.002	2e-15
rs28449958	16	28528781	A	G	0.343	-0.013	0.002	4.7e-09
rs9783783	16	30078492	C	G	0.502	-0.013	0.002	1.7e-09
rs12598049	16	53316854	G	A	0.299	-0.020	0.002	1.4e-17
rs12924604	16	74601486	A	G	0.198	-0.032	0.003	3.4e-34
rs374029	16	85725953	A	G	0.297	0.018	0.002	1.2e-14
rs428578	16	85917944	G	A	0.348	-0.077	0.002	1e-200
rs305071	16	85949271	A	G	0.120	-0.108	0.003	1e-200
rs2176777	16	86012521	C	G	0.813	0.056	0.003	3.2e-95
rs16940328	16	86076108	A	G	0.113	0.035	0.003	6.6e-26
rs3743860	16	89818491	C	T	0.416	0.013	0.002	4.3e-09
rs9900036	17	4739161	G	T	0.344	-0.014	0.002	1.2e-09
rs61759532	17	7240391	T	C	0.247	0.022	0.003	5.5e-18
rs74480102	17	7742601	A	G	0.043	0.068	0.005	4.4e-39
rs541313781	17	16749390	G	A	0.028	-0.048	0.008	2e-10
rs2948542	17	25856486	G	A	0.587	-0.018	0.002	2.6e-17
rs894606	17	27909352	T	C	0.420	0.015	0.002	9.3e-13
rs7218453	17	32535460	T	A	0.221	-0.028	0.003	1.3e-27
rs57968500	17	38145828	G	A	0.388	-0.066	0.002	1e-200
rs79364306	17	41627966	T	C	0.060	-0.027	0.004	2.2e-09
rs4997213	17	44186267	A	G	0.180	-0.020	0.003	1.2e-13
rs11656379	17	47273515	G	A	0.162	-0.019	0.003	4.3e-11
rs8178414	17	56345363	T	C	0.013	-0.070	0.009	1.1e-13
rs34097845	17	56358429	T	C	0.057	-0.137	0.005	4.3e-184
rs7216675	17	57882674	C	T	0.079	-0.058	0.004	6.3e-49
rs734232	17	72765731	A	G	0.463	-0.020	0.002	1.7e-21
rs79647649	17	80295875	C	T	0.042	-0.047	0.005	3.1e-19
rs12946146	17	81079703	T	C	0.486	0.055	0.002	1.2e-125
rs149519071	17	81152877	C	T	0.331	0.020	0.003	3.9e-10
rs238135	18	3448979	C	T	0.290	-0.020	0.002	1.5e-16
rs303752	18	21074255	A	G	0.408	0.016	0.002	3.5e-13
rs4800173	18	21619931	G	A	0.804	0.021	0.003	2.7e-14
rs8098694	18	60780199	A	G	0.441	-0.016	0.002	2e-13
rs954954	18	60902328	C	A	0.105	-0.028	0.003	2.5e-16
rs17758695	18	60920854	T	C	0.029	-0.073	0.006	1.6e-31
rs3177609	18	74071078	C	T	0.077	0.058	0.004	9.9e-49
rs3786228	18	77464170	C	T	0.383	-0.022	0.002	3.1e-23
rs138303849	19	837174	C	G	0.254	0.028	0.003	2.7e-28
rs376882860	19	3174514	T	C	0.080	-0.033	0.004	1.7e-13

rs57900491	19	4995295	T	A	0.341	-0.018	0.002	5.4e-15
rs413141	19	6675989	G	A	0.863	0.033	0.003	1.5e-26
rs3786602	19	16730196	C	T	0.346	-0.015	0.002	4e-12
rs11666267	19	17660300	A	G	0.566	-0.014	0.002	1.9e-10
rs11086102	19	18398628	C	G	0.633	0.035	0.002	3.2e-57
rs3859570	19	18510925	C	T	0.425	-0.045	0.002	4.1e-96
rs7256148	19	30345526	G	A	0.498	-0.014	0.002	7.5e-11
rs62126615	19	33749710	T	C	0.167	0.028	0.003	3.4e-22
rs12151289	19	33751852	C	G	0.030	-0.094	0.006	7.9e-50
rs8103728	19	33900257	G	C	0.670	0.015	0.002	9e-12
rs28528757	19	36732215	A	G	0.226	-0.016	0.003	8.9e-11
rs4760	19	44153100	G	A	0.156	0.045	0.003	8.8e-54
rs62116961	19	44283843	C	G	0.376	-0.043	0.002	1.1e-83
rs12982781	19	45786555	C	T	0.339	-0.024	0.002	1.1e-26
rs73052804	19	52155401	T	C	0.072	-0.025	0.004	1.9e-09
rs62143206	19	54326212	T	G	0.211	-0.078	0.003	1.6e-196
rs602444	19	55246731	A	C	0.614	-0.017	0.002	3.7e-12
rs1883932	20	8609588	T	A	0.508	-0.015	0.002	6e-12
rs6121172	20	30296095	C	A	0.288	-0.020	0.002	2.9e-17
rs4911236	20	31082056	A	G	0.308	-0.034	0.002	2e-46
rs61114499	20	31192357	A	G	0.259	0.060	0.002	1.4e-132
rs4142441	20	42839620	G	A	0.151	0.044	0.003	1.6e-47
rs932905	20	48796131	A	G	0.281	0.022	0.002	2.3e-20
rs17196752	20	48887268	T	C	0.189	-0.079	0.003	1.9e-182
rs6020468	20	48967103	A	G	0.195	-0.051	0.003	7.9e-80
rs2254953	20	61574660	G	A	0.722	0.014	0.002	3.1e-09
rs2823002	21	16373836	T	A	0.158	-0.022	0.003	1.8e-14
rs11088296	21	36237546	T	C	0.262	0.014	0.002	2.2e-09
rs2834754	21	36463774	G	C	0.706	0.015	0.002	2.4e-10
rs80109907	21	39850955	A	C	0.009	-0.107	0.011	1.1e-21
rs4817987	21	40465901	T	C	0.266	0.019	0.002	6.2e-15
rs17176611	21	47676948	C	A	0.172	0.019	0.003	5.6e-12
rs41409548	22	17579495	A	G	0.033	-0.096	0.006	1.9e-50
rs5748937	22	17675324	T	C	0.054	-0.060	0.005	1.3e-32
rs9605235	22	17702355	A	G	0.094	-0.023	0.004	2.6e-10
rs143283354	22	24646573	T	C	0.275	-0.016	0.002	4.6e-11
rs2015580	22	32326021	T	C	0.312	-0.017	0.002	5.8e-13
rs62234274	22	43549646	T	C	0.573	-0.023	0.002	4.5e-26
rs738408	22	44324730	T	C	0.216	0.023	0.003	1.2e-19
rs8138399	22	50044824	T	C	0.669	0.014	0.002	1.4e-10

Table S18. 307 SNPs significantly associated with lymphocyte percentage of white cells used IVs in forward MR analyses derived from Vuckovic D et al. Chr: Chromosome; EA: Effect allele; NEA: Non-effect allele; EAF: Effect allele frequency.

SNP	Chr	Pos	EA	NEA	EAF	Beta	SE	P-value
rs1569419	1	2996602	C	T	0.767	-0.016	0.003	2.2e-10
rs11121242	1	8906301	G	A	0.513	-0.015	0.002	2.7e-12
rs589942	1	20916080	G	C	0.338	0.017	0.002	3e-14
rs79567479	1	27011533	C	T	0.080	0.029	0.004	1.9e-13
rs3917932	1	36943916	G	C	0.577	0.032	0.002	9.7e-48
rs80200046	1	43423932	A	G	0.172	0.024	0.003	3.3e-17
rs6703960	1	46232723	T	C	0.457	0.013	0.002	7.1e-10
rs10789009	1	56331635	G	A	0.562	0.013	0.002	1.1e-09
rs7537229	1	56906274	A	G	0.896	-0.045	0.004	1e-37
rs2767481	1	66015394	C	T	0.705	-0.022	0.002	7.1e-20
rs1925408	1	67365034	T	C	0.735	-0.022	0.002	6.8e-19
rs41313381	1	79411968	A	C	0.031	-0.069	0.006	4.1e-30
rs150649461	1	92925654	C	G	0.015	-0.114	0.009	1.5e-35
rs2148404	1	101225444	A	G	0.590	-0.022	0.002	2e-23
rs9433774	1	101755299	T	C	0.617	-0.016	0.002	7.3e-13
rs325927	1	111698348	C	T	0.654	-0.019	0.002	1.7e-17
rs7555995	1	150212707	G	C	0.286	0.017	0.002	3.7e-12
rs3894771	1	150789961	T	A	0.448	0.022	0.002	2.4e-23
rs34599082	1	159175494	T	C	0.014	0.121	0.009	5.2e-39
rs571841	1	160791411	T	C	0.426	0.021	0.002	1.4e-21
rs10489481	1	185391167	A	G	0.321	0.016	0.002	1.6e-12
rs16841904	1	197701992	T	C	0.213	-0.022	0.003	5.6e-17
rs7555082	1	198598663	A	G	0.118	0.027	0.003	1.5e-15
rs1434282	1	199010721	T	C	0.725	0.015	0.002	7e-10
rs55734382	1	201019059	T	C	0.333	0.016	0.002	9.3e-12
rs4311947	1	212880752	T	G	0.711	0.016	0.002	6.5e-12
rs7552783	1	227174145	C	T	0.512	0.021	0.002	7e-19
rs6697464	1	235007116	T	A	0.291	-0.014	0.002	4.5e-09
rs9970896	1	236104981	T	A	0.888	0.030	0.003	2e-18
rs61838753	1	247569300	C	A	0.672	0.014	0.002	9.3e-10
rs12105729	2	7614263	G	A	0.159	-0.023	0.003	2.3e-14
rs10779936	2	23950200	A	G	0.715	0.022	0.002	1.4e-20
rs7578575	2	25488819	A	T	0.293	0.036	0.002	7.3e-51
rs1124649	2	27260469	A	G	0.299	-0.019	0.002	4e-16
rs4952073	2	31457981	C	T	0.703	-0.017	0.002	3.2e-12
rs11688289	2	40698811	C	T	0.189	-0.019	0.003	7.1e-12
rs6705577	2	43359275	C	G	0.271	0.018	0.002	1.9e-13
rs113542380	2	43464818	A	G	0.075	-0.087	0.004	5e-99
rs698853	2	54053872	G	A	0.276	-0.017	0.002	4e-12
rs2860773	2	65665641	T	G	0.408	-0.020	0.002	1.3e-19
rs10048745	2	68962137	A	G	0.256	-0.021	0.002	8.2e-18
rs4599108	2	85543222	T	C	0.486	0.014	0.002	2.5e-10
rs6419573	2	103027103	C	T	0.780	0.026	0.003	2.2e-23
rs10210955	2	111599282	C	G	0.490	0.025	0.002	1.6e-31
rs55706446	2	112239082	T	C	0.075	-0.061	0.004	5e-45
rs13409371	2	113838145	A	G	0.396	-0.025	0.002	1.1e-29
rs80018307	2	129030338	C	T	0.050	0.033	0.005	5.1e-11
rs6750955	2	136982502	A	C	0.907	-0.029	0.004	3.6e-15
rs354719	2	143892139	T	A	0.825	-0.025	0.003	6.3e-18
rs34030812	2	144248905	C	T	0.367	-0.014	0.002	3.8e-10
rs12052715	2	160677375	G	C	0.726	0.022	0.002	2e-19

rs6746841	2	161280888	A	G	0.747	0.027	0.003	1.4e-27
rs199514773	2	181925838	A	T	0.641	0.024	0.002	3.3e-24
rs6760805	2	182157839	G	A	0.388	0.028	0.002	2.6e-37
rs13029501	2	182334753	T	G	0.563	-0.019	0.002	9.7e-19
rs75507031	2	202160762	T	C	0.732	0.022	0.002	2.3e-20
rs114050631	2	219020958	T	C	0.011	0.098	0.011	3.4e-19
rs78058190	2	219699999	A	G	0.050	0.046	0.006	1.5e-16
rs6713887	2	220026369	T	G	0.638	-0.015	0.002	1.9e-11
rs11676298	2	227291731	G	C	0.192	0.020	0.003	5.5e-14
rs57003673	2	231123460	C	A	0.108	0.023	0.003	2.8e-11
rs6741554	2	232272500	G	A	0.248	-0.015	0.002	1e-09
rs2568569	2	237796392	G	A	0.739	-0.015	0.002	2.7e-09
rs9826367	3	12294202	G	A	0.449	-0.016	0.002	3.7e-14
rs75831154	3	28284622	G	C	0.175	0.032	0.003	2.2e-29
rs11706384	3	39296881	T	G	0.236	0.016	0.003	7.2e-10
rs7433284	3	42872590	A	G	0.383	0.021	0.002	5.1e-22
rs13063578	3	47087837	A	T	0.401	0.036	0.002	2.4e-55
rs71298382	3	71382067	G	A	0.027	0.091	0.007	6.6e-39
rs13314303	3	71421314	T	C	0.138	-0.019	0.003	2.5e-09
rs1584335	3	98394871	C	T	0.606	-0.015	0.002	2.7e-11
rs6781942	3	119758049	G	A	0.372	0.015	0.002	3.2e-12
rs4474982	3	123068820	A	G	0.177	-0.019	0.003	8.5e-12
rs6782228	3	128323424	C	G	0.268	0.038	0.002	1.5e-53
rs7625643	3	141150026	G	A	0.446	0.021	0.002	1.6e-21
rs6440859	3	152977859	A	T	0.574	0.013	0.002	1.1e-09
rs1565568	3	171176050	A	T	0.196	-0.018	0.003	1.1e-10
rs68062772	3	195171795	G	A	0.183	0.019	0.003	1.2e-11
rs6831368	4	6969919	G	A	0.362	0.021	0.002	1.6e-20
rs6531603	4	38031267	C	T	0.668	0.019	0.002	1.9e-16
rs12649942	4	38261175	C	T	0.352	0.014	0.002	1.6e-09
rs6841652	4	38363262	C	T	0.680	0.060	0.002	5.5e-148
rs6827279	4	38566132	T	C	0.539	-0.036	0.002	8.7e-59
rs218265	4	55408999	C	T	0.155	-0.032	0.003	1.5e-26
rs6554196	4	55501109	G	C	0.382	0.020	0.002	2.3e-19
rs16850073	4	74703999	T	C	0.375	-0.039	0.002	1.7e-68
rs146149115	4	74768492	G	A	0.010	0.079	0.011	2.2e-12
rs546829	4	74956372	T	A	0.626	0.047	0.002	7.1e-103
rs28830602	4	80908833	G	A	0.629	-0.020	0.002	4e-20
rs11726195	4	103538911	G	C	0.522	0.033	0.002	1e-53
rs6533181	4	106074826	G	T	0.632	0.038	0.002	8.1e-64
rs309375	4	123681156	A	C	0.572	0.020	0.002	1.2e-19
rs60428870	4	144896336	A	G	0.616	-0.015	0.002	1.3e-10
rs4696314	4	153005716	C	T	0.512	0.022	0.002	3.9e-24
rs2853677	5	1287194	A	G	0.577	0.020	0.002	2.7e-20
rs3822412	5	10689768	G	A	0.369	-0.015	0.002	4e-11
rs11567701	5	35859863	T	G	0.260	0.042	0.002	6.1e-67
rs4865744	5	52082774	G	A	0.921	-0.028	0.004	1.5e-12
rs13154469	5	68607707	C	T	0.441	0.016	0.002	5.6e-14
rs10942300	5	71745412	T	C	0.880	0.038	0.003	9.4e-30
rs304151	5	88125853	G	T	0.268	0.020	0.002	3e-16
rs2910687	5	96276877	T	C	0.435	-0.014	0.002	1.4e-10
rs114378220	5	110566360	T	C	0.074	-0.033	0.004	2.4e-14
rs2250127	5	127388844	A	G	0.247	-0.018	0.002	9.9e-13
rs3846730	5	131806780	T	C	0.237	-0.022	0.003	2.4e-18
rs7700687	5	141491985	T	C	0.617	-0.020	0.002	3e-20
rs56330463	5	148200011	C	T	0.552	0.022	0.002	6.7e-23

rs9313777	5	157945599	G	T	0.281	-0.023	0.002	1.6e-22
rs535833686	5	158347179	G	A	0.024	-0.047	0.008	8.8e-10
rs55775466	5	172006296	G	A	0.120	-0.020	0.003	8.4e-10
rs12659737	5	176742506	C	T	0.840	0.032	0.003	4e-27
rs55785724	5	176817583	T	A	0.312	-0.015	0.002	8.7e-11
rs72835315	6	231878	C	T	0.058	0.031	0.005	3.2e-11
rs1050979	6	410417	G	A	0.528	0.014	0.002	1.7e-10
rs3777755	6	12159699	T	C	0.310	-0.019	0.002	7.8e-17
rs2560775	6	13525365	A	G	0.692	0.020	0.002	4.6e-18
rs1267498	6	14715910	C	T	0.807	-0.024	0.003	5.9e-19
rs4713208	6	29283579	C	T	0.616	0.013	0.002	2.9e-09
rs28883698	6	29846390	A	G	0.200	-0.043	0.003	3.7e-34
rs73390981	6	31243578	A	G	0.078	0.065	0.004	3.4e-57
rs7758790	6	31552850	C	T	0.226	0.023	0.003	1.2e-18
rs113571892	6	32489037	C	T	0.251	-0.029	0.003	2.2e-24
rs17880292	6	32552000	T	C	0.043	-0.055	0.007	4.7e-16
rs11758943	6	42501162	C	G	0.208	-0.018	0.003	5.5e-12
rs1533584	6	53221183	C	T	0.301	0.017	0.002	8.2e-13
rs915125	6	82463376	T	C	0.279	0.018	0.002	4.9e-14
rs3734187	6	87969737	T	C	0.490	0.022	0.002	2.3e-25
rs12526696	6	108053364	A	G	0.161	0.033	0.003	3.9e-29
rs9487023	6	109590004	G	A	0.448	-0.024	0.002	4.2e-28
rs577721086	6	127440047	C	T	0.050	0.030	0.005	1.2e-09
rs12203816	6	135529561	T	C	0.401	0.017	0.002	1.1e-14
rs1012515	6	137216847	T	A	0.494	0.015	0.002	5.3e-12
rs142761146	6	138004508	A	T	0.181	0.018	0.003	2.1e-10
rs566028	6	147520965	C	T	0.452	0.016	0.002	4e-13
rs6956301	7	971124	A	G	0.667	-0.016	0.002	2.9e-12
rs6955702	7	3157702	G	A	0.524	-0.021	0.002	7.3e-23
rs2347784	7	6524843	G	C	0.271	0.031	0.002	2.5e-36
rs11763992	7	22725522	T	A	0.305	-0.014	0.002	1.7e-09
rs6978504	7	26695880	G	C	0.344	-0.016	0.002	4.4e-13
rs498475	7	28256240	A	G	0.633	0.016	0.002	1.5e-12
rs2158799	7	28277107	G	C	0.610	-0.038	0.002	7.1e-67
rs56388170	7	28724374	T	G	0.292	-0.049	0.002	1.6e-95
rs532636967	7	40060707	C	T	0.461	-0.015	0.002	3.6e-11
rs149007767	7	50370254	T	C	0.162	-0.023	0.003	2.8e-14
rs2943379	7	65174354	T	C	0.098	-0.021	0.004	3.7e-09
rs2108321	7	74297551	A	T	0.285	0.019	0.002	1e-14
rs42035	7	92239531	G	A	0.245	-0.023	0.003	1.5e-19
rs445	7	92408370	T	C	0.096	0.068	0.004	7.2e-77
rs56229643	7	97865936	C	T	0.491	0.022	0.002	1.4e-23
rs776739	7	99283696	C	T	0.082	0.031	0.004	1.6e-15
rs9920	7	116200092	C	T	0.105	0.026	0.004	1.1e-13
rs62621812	7	127015083	A	G	0.020	0.071	0.008	1e-19
rs62485905	7	138741586	T	C	0.551	-0.013	0.002	2.2e-09
rs3735080	7	150217309	T	C	0.230	0.024	0.003	1.1e-21
rs7457817	7	156596400	C	T	0.418	-0.013	0.002	9.5e-10
rs17453815	8	5549619	G	A	0.231	-0.020	0.003	1.8e-14
rs28494016	8	9187961	C	A	0.792	-0.018	0.003	1.2e-11
rs4840514	8	10612925	A	G	0.312	-0.022	0.002	2.2e-21
rs12550367	8	22793451	C	T	0.347	0.015	0.002	1.3e-11
rs2979489	8	30280833	A	G	0.742	-0.021	0.002	2.3e-18
rs66593272	8	41548911	T	A	0.037	-0.033	0.006	4.1e-09
rs4737009	8	41630405	A	G	0.236	0.025	0.003	1.7e-23
rs45577137	8	48651633	G	A	0.045	-0.033	0.006	1.9e-09

rs7846314	8	61650831	T	A	0.187	-0.047	0.003	3.8e-66
rs10111413	8	72891748	A	G	0.231	-0.016	0.003	1.2e-10
rs75653581	8	78363128	T	C	0.013	0.068	0.010	5.5e-12
rs2919917	8	79657263	C	T	0.250	0.041	0.002	8.5e-62
rs4521730	8	101180650	A	G	0.185	0.019	0.003	1.8e-11
rs6988147	8	101459320	T	C	0.863	-0.020	0.003	1.1e-10
rs6988140	8	126480543	T	C	0.543	-0.024	0.002	1e-24
rs1865223	8	130606476	T	G	0.568	0.040	0.002	5e-74
rs16904181	8	130976074	A	G	0.301	0.016	0.002	3.3e-11
rs2219143	9	2622278	A	G	0.393	-0.016	0.002	3e-12
rs385893	9	4763176	C	T	0.524	-0.026	0.002	8.9e-34
rs3731211	9	21986847	A	T	0.722	0.026	0.002	4.7e-27
rs2065500	9	22145694	G	A	0.158	-0.027	0.003	1.8e-19
rs10971930	9	34116083	C	T	0.127	0.020	0.003	8.2e-10
rs10867416	9	82324152	C	T	0.656	-0.016	0.002	1.4e-12
rs61750929	9	91495135	T	C	0.056	-0.055	0.005	2.5e-31
rs2120229	9	123440875	G	T	0.491	0.014	0.002	2.5e-10
rs2519093	9	136141870	T	C	0.184	0.027	0.003	1.1e-22
rs28545822	9	139217805	C	T	0.467	0.014	0.002	2.1e-10
rs11145986	9	139319847	G	A	0.290	-0.019	0.002	9.3e-16
rs61839660	10	6094697	T	C	0.098	-0.046	0.004	1.1e-36
rs2388486	10	8099021	C	T	0.830	0.024	0.003	1.1e-16
rs2992327	10	26711899	T	A	0.273	-0.016	0.002	1.1e-10
rs2807742	10	28781367	A	G	0.770	-0.022	0.003	2.9e-17
rs17156360	10	44922040	T	C	0.132	0.020	0.003	3.6e-10
rs56197478	10	50301892	G	A	0.221	-0.017	0.003	8.5e-11
rs7090445	10	63721176	T	C	0.660	-0.022	0.002	5.1e-23
rs142239370	10	72382869	C	A	0.040	0.053	0.005	3.8e-22
rs1250546	10	81032532	G	A	0.437	0.019	0.002	7.2e-19
rs1539330	10	94502738	T	C	0.134	0.025	0.003	6.2e-15
rs10882827	10	98371790	T	C	0.179	-0.018	0.003	1.4e-10
rs11189132	10	99086195	C	G	0.396	0.025	0.002	5.9e-29
rs11198861	10	121049819	A	G	0.512	0.016	0.002	2.7e-13
rs2885521	10	126373313	T	C	0.110	-0.029	0.003	7.3e-17
rs14408	11	308314	C	T	0.368	-0.031	0.002	3.8e-44
rs12289940	11	4224589	A	T	0.378	-0.013	0.002	1.4e-09
rs4756265	11	36078171	G	A	0.418	0.016	0.002	1.8e-13
rs4647725	11	47245389	C	T	0.157	-0.023	0.003	1.9e-14
rs7232	11	59940599	A	T	0.378	0.019	0.002	1.3e-17
rs61897795	11	61618169	G	A	0.164	0.021	0.003	8.4e-13
rs11231693	11	63862612	A	G	0.055	-0.028	0.005	3.2e-09
rs554169857	11	65686473	A	C	0.020	-0.048	0.008	1.8e-09
rs7930161	11	78268878	A	C	0.826	0.020	0.003	2.9e-12
rs72992361	11	100427512	A	C	0.082	-0.025	0.004	9.8e-11
rs680113	11	108165406	A	G	0.555	-0.016	0.002	3.7e-14
rs73000965	11	113982321	A	T	0.317	-0.014	0.002	4e-09
rs73564493	11	119056273	A	G	0.073	-0.026	0.004	1.5e-10
rs11218725	11	122509237	G	A	0.377	0.029	0.002	9e-40
rs4520607	11	128406775	C	T	0.525	0.021	0.002	1.6e-21
rs2364482	12	6502131	G	T	0.197	0.041	0.003	4.1e-51
rs7960048	12	12554861	G	T	0.466	0.013	0.002	1.3e-09
rs35071163	12	12883507	C	G	0.479	0.017	0.002	2e-15
rs113736796	12	48213720	G	C	0.039	-0.047	0.006	1.7e-16
rs1793937	12	48375475	G	C	0.401	0.015	0.002	2.3e-11
rs75639074	12	56367901	G	C	0.063	0.026	0.004	4.8e-09
rs4761234	12	69732105	C	T	0.485	-0.020	0.002	7.4e-21

rs4842610	12	88811689	T	C	0.705	0.015	0.002	2.8e-10
rs653178	12	112007756	T	C	0.518	-0.041	0.002	2e-81
rs11064881	12	120146925	A	G	0.073	0.026	0.004	4.5e-10
rs28413626	12	123861452	A	G	0.214	0.032	0.003	2.1e-34
rs117808697	13	28513538	T	A	0.073	-0.027	0.005	4.4e-09
rs2504235	13	28612886	G	A	0.638	-0.024	0.002	2.8e-27
rs9532679	13	41522338	C	A	0.150	-0.034	0.003	2.2e-29
rs9533100	13	42996548	T	G	0.536	-0.021	0.002	3.8e-23
rs9316484	13	50645164	C	T	0.220	-0.018	0.003	2.9e-12
rs67483792	13	72503638	T	C	0.043	-0.040	0.005	2.1e-13
rs9530281	13	74679161	A	G	0.716	-0.031	0.002	2.7e-39
rs7981577	13	114835802	C	T	0.437	-0.020	0.002	5e-20
rs2004925	14	25445362	A	G	0.393	-0.026	0.002	6.7e-31
rs10131567	14	35864878	T	G	0.285	-0.020	0.002	4.6e-17
rs11848179	14	64977053	G	A	0.318	0.025	0.002	9.9e-27
rs112506826	14	69975724	T	G	0.030	-0.042	0.006	5.7e-11
rs9323612	14	75968608	G	A	0.331	-0.021	0.002	8.5e-20
rs61975764	14	93014929	A	G	0.465	-0.019	0.002	2.9e-18
rs72699870	14	93116351	C	T	0.195	0.022	0.003	2.6e-16
rs11625841	14	99787017	C	T	0.619	-0.014	0.002	7.1e-10
rs8008502	14	103359492	A	G	0.202	0.031	0.003	5.8e-30
rs1132639	15	41597437	A	T	0.295	0.018	0.002	1.4e-14
rs4924590	15	42233673	A	T	0.244	-0.027	0.003	9.1e-28
rs60820695	15	48584883	T	C	0.134	-0.022	0.003	8.6e-12
rs6494476	15	64657496	T	C	0.879	-0.022	0.003	2.5e-11
rs77365378	15	69972289	T	C	0.046	0.032	0.005	8.5e-10
rs12908814	15	75132490	G	C	0.629	-0.017	0.002	4.9e-15
rs17832339	15	90557494	T	G	0.039	0.037	0.006	2.1e-11
rs28539372	15	91021412	A	T	0.319	0.035	0.002	1.3e-50
rs67175901	15	101748227	T	C	0.108	-0.031	0.003	3.2e-19
rs447361	16	4148240	G	T	0.669	0.015	0.002	3.2e-11
rs61739285	16	27480797	T	C	0.034	0.044	0.006	7.8e-14
rs11150589	16	30482494	C	T	0.517	0.027	0.002	1.5e-36
rs7192652	16	57075180	G	A	0.479	0.023	0.002	2.3e-25
rs11640346	16	81603848	T	G	0.537	-0.014	0.002	2.7e-10
rs1519978	16	85935211	T	A	0.168	-0.022	0.003	3.2e-14
rs72803323	16	89373324	T	C	0.040	-0.038	0.005	6.8e-12
rs9905106	17	1373518	C	T	0.735	-0.024	0.002	3.4e-23
rs9674692	17	2730030	T	G	0.277	0.018	0.002	6.3e-14
rs28662192	17	4604497	C	T	0.294	0.019	0.002	1.8e-15
rs9895848	17	4738958	T	C	0.345	-0.019	0.002	1.3e-16
rs9910148	17	16169009	A	G	0.476	0.038	0.002	5.9e-69
rs4924997	17	19422725	T	A	0.460	-0.015	0.002	4.9e-12
rs4239225	17	38127112	A	G	0.472	-0.069	0.002	1e-200
rs11654706	17	38147293	G	A	0.168	0.021	0.003	7.9e-13
rs146346285	17	38897220	T	C	0.014	-0.074	0.010	1.8e-14
rs8081535	17	41080195	A	G	0.436	0.018	0.002	1.2e-14
rs1706725	17	44324185	G	A	0.236	-0.028	0.003	1.1e-27
rs28730837	17	56355397	A	G	0.016	-0.060	0.008	1.9e-12
rs138284624	17	56385573	T	C	0.013	-0.103	0.010	6.8e-27
rs13342015	17	73067482	A	G	0.304	-0.015	0.002	4.8e-11
rs4789294	17	74419177	G	A	0.217	0.026	0.003	6.4e-23
rs11659109	17	75375859	T	C	0.483	-0.016	0.002	3.5e-14
rs7231555	18	19699122	G	A	0.088	-0.034	0.004	1e-18
rs75763843	18	42062380	C	A	0.129	-0.032	0.003	6.2e-23
rs9947316	18	43777831	G	T	0.559	0.013	0.002	5.8e-10

rs66836460	18	60837508	A	C	0.136	0.021	0.003	9.9e-12
rs56249713	18	67533332	C	T	0.420	0.016	0.002	4.1e-13
rs454854	18	77253866	C	G	0.679	-0.015	0.002	6.1e-11
rs36084354	19	1079959	A	G	0.092	-0.038	0.004	2.4e-24
rs28841391	19	1193965	G	T	0.759	0.024	0.003	9.1e-19
rs2569703	19	10404227	G	C	0.544	-0.032	0.002	4.2e-49
rs537835324	19	12994335	T	C	0.191	-0.019	0.003	6.4e-11
rs345627	19	13917264	T	C	0.349	0.023	0.002	7.6e-25
rs10423726	19	16444697	A	C	0.580	-0.036	0.002	3.1e-54
rs7251806	19	16499569	T	C	0.841	-0.088	0.003	1e-194
rs4530278	19	33752994	T	G	0.601	0.018	0.002	6.9e-16
rs59058760	19	40811757	C	T	0.068	0.026	0.004	7.9e-10
rs4760	19	44153100	G	A	0.156	0.039	0.003	3e-39
rs11673093	19	45742094	A	G	0.260	0.027	0.002	1.5e-28
rs8102492	19	49140809	C	A	0.796	-0.036	0.003	3e-40
rs1028397	20	1929662	A	G	0.331	-0.033	0.002	1.3e-47
rs1010608	20	3784593	A	G	0.236	0.018	0.003	4.3e-12
rs73125628	20	20066701	T	C	0.278	0.015	0.002	1.3e-10
rs910083	20	31378690	C	A	0.447	0.016	0.002	5.6e-14
rs6073369	20	42832789	T	C	0.314	0.019	0.002	2.4e-16
rs2230604	20	49196284	T	C	0.084	0.029	0.004	7.3e-14
rs259981	20	57748369	A	T	0.359	-0.026	0.002	3.7e-31
rs4809330	20	62349586	G	A	0.668	-0.020	0.002	2.3e-18
rs1736023	21	16812882	T	C	0.430	0.014	0.002	1.9e-10
rs2212931	21	39859190	G	A	0.417	-0.014	0.002	1.8e-10
rs220262	21	43482046	A	T	0.482	-0.016	0.002	2.2e-13
rs5747308	22	18133500	C	A	0.505	-0.019	0.002	7.9e-18
rs5998509	22	21917479	T	C	0.188	-0.033	0.003	2.5e-33
rs2144922	22	28923317	A	G	0.878	0.027	0.003	2.7e-16
rs2412971	22	30494371	A	G	0.446	0.028	0.002	3e-37
rs5757886	22	40636076	C	T	0.747	-0.017	0.002	2e-12
rs5758307	22	41750219	G	A	0.229	0.028	0.003	1.6e-27

Table S19. 9 SNPs significantly associated with COVID-19 severity (very severe respiratory confirmed covid vs. population) used IVs in reverse MR analyses derived from the COVID-19 Host Genetics Initiative. Chr: Chromosome; EA: Effect allele; NEA: Non-effect allele; EAF: Effect allele frequency.

SNP	Chr	Pos	EA	NEA	EAF	Beta	SE	P-value
rs35081325	3	45889921	T	A	0.075	0.626	0.045	5.754e-45
rs111837807	6	31121232	C	T	0.100	0.295	0.043	5.656e-12
rs622568	7	54647894	C	A	0.146	0.226	0.037	1.044e-09
rs2237698	7	107607902	T	C	0.090	0.237	0.040	2.414e-09
rs10860891	12	103014757	A	C	0.885	-0.239	0.040	1.636e-09
rs2384074	12	113382977	T	C	0.676	0.198	0.028	2.104e-12
rs77534576	17	47940666	T	C	0.035	0.460	0.075	8.525e-10
rs2109069	19	4719443	A	G	0.329	0.257	0.028	6.115e-20
rs13050728	21	34615210	C	T	0.663	-0.200	0.029	2.437e-12

Table S20. 5 SNPs significantly associated with COVID-19 severity (hospitalized covid vs. population) used IVs in reverse MR analyses derived from the COVID-19 Host Genetics Initiative. Chr: Chromosome; EA: Effect allele; NEA: Non-effect allele; EAF: Effect allele frequency.

SNP	Chr	Pos	EA	NEA	EAF	Beta	SE	P-value
rs35081325	3	45889921	T	A	0.081	0.488	0.032	3.684e-54
rs622568	7	54647894	C	A	0.151	0.154	0.026	3.644e-09
rs2660	12	113357442	A	G	0.690	0.116	0.019	2.005e-09
rs2109069	19	4719443	A	G	0.323	0.151	0.020	2.937e-14
rs13050728	21	34615210	C	T	0.653	-0.168	0.020	7.437e-17

Table S21. 7 SNPs significantly associated with COVID-19 susceptibility (covid vs. population) used IVs in reverse MR analyses derived from the COVID-19 Host Genetics Initiative. Chr: Chromosome; EA: Effect allele; NEA: Non-effect allele; EAF: Effect allele frequency.

SNP	Chr	Pos	EA	NEA	EAF	Beta	SE	P-value
rs4971066	1	155105882	G	T	0.178	-0.077	0.013	1.015e-08
rs2271616	3	45838013	T	G	0.118	0.156	0.015	3.608e-25
rs17078348	3	45847241	G	A	0.100	0.092	0.016	1.196e-08
rs10936744	3	101433440	T	C	0.359	-0.063	0.010	3.511e-10
rs579459	9	136154168	T	C	0.790	-0.094	0.011	2.183e-16
rs757405	12	113406945	A	T	0.709	0.069	0.011	1.639e-10
rs12482060	21	34611571	G	C	0.338	0.062	0.011	3.958e-09
